# Enzymatic epimerization of monoterpene indole alkaloids in kratom

**DOI:** 10.1038/s41589-025-01970-9

**Published:** 2025-07-16

**Authors:** Allwin McDonald, Yoko Nakamura, Carsten Schotte, Gabriel Titchiner, Kin Lau, Ryan Alam, Adriana A. Lopes, C. Robin Buell, Sarah E. O’Connor

**Affiliations:** 1 Department of Natural Product Biosynthesis, Max Planck Institute for Chemical Ecology, Jena, Germany; 2 Research Group Biosynthesis and NMR, Max Planck Institute for Chemical Ecology, Jena, Germany; 3 Department of Plant Biology, Michigan State University, East Lansing, MI USA; 4 Biotechnology Unit, University of Ribeirão Preto (UNAERP), Ribeirão Preto, Brazil; 5 Department of Crop & Soil Sciences, University of Georgia, Athens, GA USA; 6 Center for Applied Genetic Technologies, University of Georgia, Athens, GA USA; 7 Institute of Plant Breeding, Genetics & Genomics, University of Georgia, Athens, GA USA; 8 The Plant Center, University of Georgia, Athens, GA USA

**Keywords:** Enzymes, Plant sciences, Natural products

## Abstract

Monoterpene indole alkaloids (MIAs) are a large, structurally diverse class of bioactive natural products. These compounds are biosynthetically derived from a stereoselective Pictet–Spengler condensation that generates a tetrahydro-β-carboline scaffold characterized by a 3*S* stereocenter. However, a subset of MIAs contains a noncanonical 3*R* stereocenter. Here we report the basis for 3*R*-MIA biosynthesis in *Mitragyna speciosa* (kratom). We discover the presence of the iminium species (20*S*)-3-dehydrocorynantheidine, which supports isomerization of 3*S* to 3*R* via oxidation and stereoselective reduction downstream of the initial Pictet–Spengler condensation. Isotopologue feeding experiments identify the sites for downstream MIA pathway biosynthesis as well as the oxidase/reductase pair that catalyzes this epimerization. This oxidase/reductase pair has broad substrate specificity, suggesting that this pathway may be responsible for the formation of many 3*R*-MIAs and downstream spirooxindole alkaloids in kratom. The elucidation of this epimerization mechanism allows biocatalytic access to a range of pharmacologically active spirooxindole alkaloid compounds.

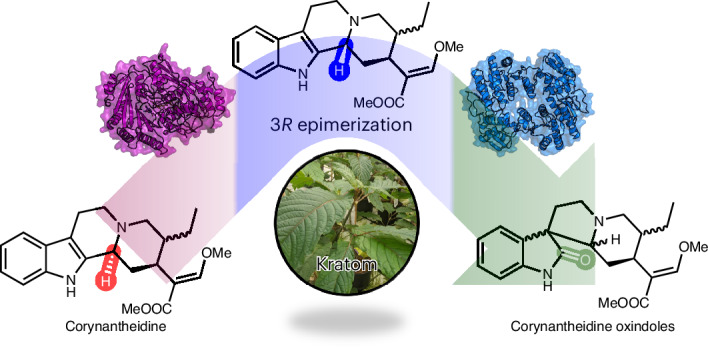

## Abstract (web summary)

Monoterpene indole alkaloids are formed via a 3*S* stereoselective condensation between secologanin and tryptamine. Here the authors uncover the mechanism of epimerization behind uncommon 3*R*-containing alkaloids in *Mitragyna speciosa* (kratom) and study their inclusion in downstream biosynthesis.

## Main

**Fig. 1:**
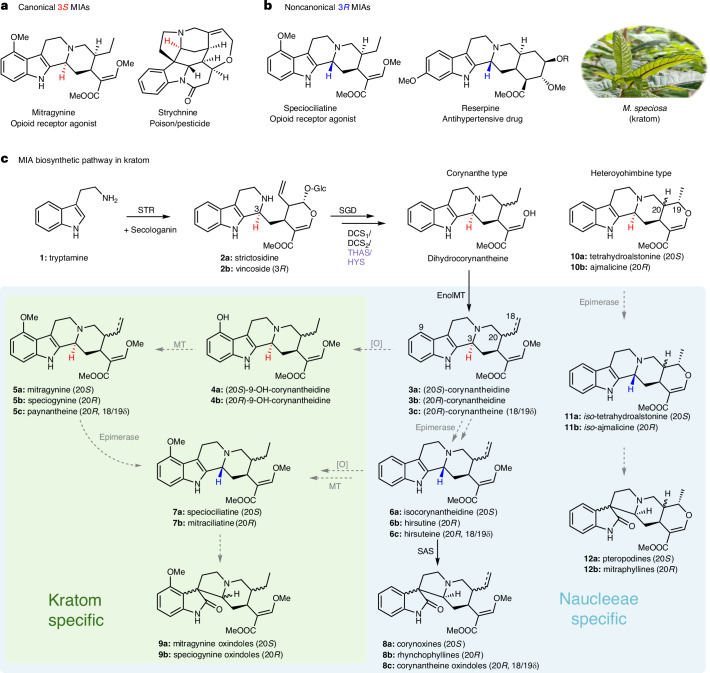
MIAs and their biosynthesis in kratom. **a**, Examples of MIAs with the canonical 3*S* stereocenter (highlighted in red). **b**, MIAs possessing the noncanonical 3*R* stereocenter (highlighted in blue). Reserpine, *R* = –(O)C–Ph(OMe)_3_. **c**, Kratom MIA biosynthetic network. Known transformations are depicted with black arrows; unknown or unverified transformations are depicted with gray dashed arrows. SGD, strictosidine β-d-glucosidase; DCS, dihydrocorynantheine synthase; THAS, tetrahydroalstonine synthase; HYS, heteroyohimbine synthase; EnolMT, enol-methyltransferase; SAS, spirooxindole alkaloid synthase; MT, unknown methyltransferase; [O], unknown oxidase.

Monoterpene indole alkaloids (MIAs) are a class of structurally diverse and pharmacologically important plant-derived natural products. This natural product family includes vinblastine (anticancer), ajmalicine (antihypertensive)^1–3^, strychnine (infamous toxin and pesticide)^4^, mitragynine (pain relief/psychedelic)^5^ and ibogaine^6^^,^^7^ (candidate for opioid withdrawal treatment; Fig. 1a). A highly conserved feature of MIA biosynthesis^4,8–10^ is the stereoselective Pictet–Spengler condensation between the aldehyde secologanin and tryptamine, catalyzed by the enzyme strictosidine synthase (STR). Strictosidine, the resulting tetrahydro-β-carboline product, is characterized by a 3*S* stereocenter, the stereochemical configuration observed in the vast majority of MIAs (Fig. 1a). More rarely, however, MIAs possess a 3*R* stereocenter (Fig. 1b), a subset that includes reserpine (an adrenergic blocking agent previously approved to treat high blood pressure)^11^, hirsutine (a potent antiarrhythmic and vasodilator)^12,13^ and speciociliatine (a more potent µ-opioid receptor agonist than the corresponding 3*S* epimer, mitragynine)^14^. 3*R*-MIAs are concentrated within the Naucleeae tribe of the Rubiaceae plant family^15^, in species such as *Mitragyna speciosa*^16^, *Uncaria* sp.^17–19^, *Pausinystalia yohimbe*^20^ and *Cephalanthus occidentalis*^21^. The mechanism by which this 3*R* stereocenter is formed is unknown.

Kratom (*M. speciosa*) has attracted wide interest for its use in managing chronic pain and its potential in treating opiate withdrawal. Its pharmacological properties are derived from both the 3*S*- (for example, mitragynine (**5a**)) and 3*R*- (for example, speciociliatine (**7a**)) MIAs found in this plant (Supplementary Figs. 1 and 2)^5,14^. The biosynthetic pathway of one kratom MIA, (20*S*)-corynantheidine (**3a**, 3*S*), has been elucidated (Fig. 1c)^9,22^. Briefly, strictosidine (**2a**) is formed via Pictet–Spengler condensation of tryptamine (**1**) and secologanin by *Ms*STR, deglucosylation occurs via the action of strictosidine β-d-glucosidase and the resulting aglycone is reduced via *Ms*DCS isoforms to produce dihydrocorynantheine (both 20*S* and 20*R* isomers). *Ms*EnolMT catalyzes an unusual enol methylation, resulting in corynantheidine (**3a/3b**), which is hypothesized to then be derivatized to form a variety of downstream alkaloids (Fig. 1c). Kratom additionally accumulates a range of corynanthe- and heteroyohimbine-type 3*R*-MIAs, which, in addition to having important pharmacological properties, are postulated to serve as precursors for a plethora of biologically active spirooxindole alkaloids, such as mitraphylline (**12b**_ii_, a potential treatment for Parkinson’s disease)^18,21,23–25^. A cytochrome P450 enzyme, spirooxindole alkaloid synthase (*Ms*SAS, also known as *Ms*3eCIS), is reported to produce spirooxindole alkaloids from 3*R*-MIAs **6b** and **6c**^26^.

Here we report the elucidation of the enzymatic steps responsible for the biosynthesis of 3*R*-MIAs in kratom. Through isolation of a previously unreported kratom biosynthetic intermediate, we hypothesize that epimerization of the 3*S* center occurs on corynantheidine (**3a/3b**). Feeding of isotopically labeled substrates to kratom tissues reveals that epimerization occurs in only specific tissues, leading to the identification of an oxidase/reductase (*Ms*CO/*Ms*DCR1) pair responsible for epimerization of corynantheidine (**3a/3b**) to isocorynantheidine (**6a/6b**). Additionally, we demonstrate that the resulting 3*R*-MIAs can be enzymatically converted into spirooxindole alkaloids by the cytochrome P450 *Ms*SAS. Furthermore, we establish that *Ms*CO1, *Ms*DCR1 and *Ms*SAS each have broad substrate specificity, suggesting that these three enzymes are collectively responsible for the biosynthesis of many 3*R*-MIAs and spirooxindole alkaloids in kratom.

## Results

### Discovery of the kratom MIA iminium species

**Fig. 2:**
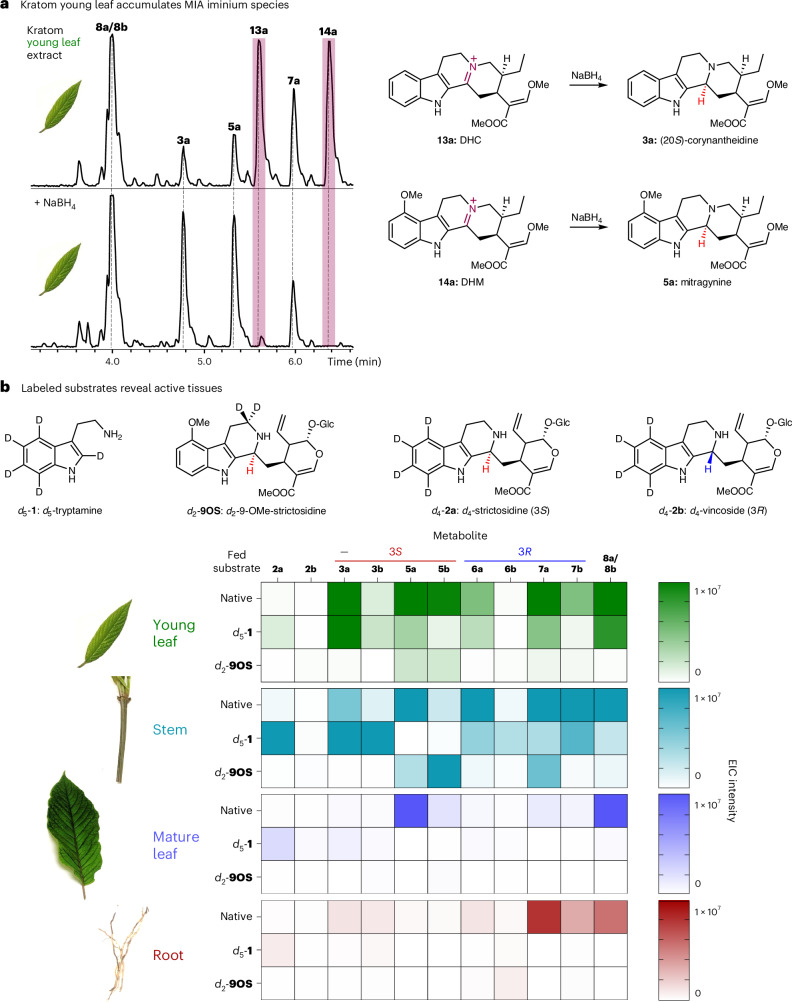
Discovery of kratom metabolites and isotopologue feeding. **a**, Comparison of kratom leaf extract with and without added NaBH_4_. The spectrum is a representative example from biological triplicates. Peaks corresponding to (20*S*)-3-DHC (**13a**) and DHM (**14a**) are highlighted. These compounds were isolated and characterized by NMR (Supplementary Figs. 62 and 65–68 and Supplementary Table 4). **b**, Native metabolite distribution and metabolite incorporation from fed labeled precursors. Values are derived from EIC from expected *m*/*z* values and are shown as the average of biological triplicates. Please note that values for fed tissues are scaled 2× (*d*_5_-**1**) or 5× (*d*_2_-**9OS**) in the above heat map for better visualization (raw values and standard error provided in Supplementary Tables 1 and 2). *d*_4_-Strictosidine (*d*_4_-**2a**) and *d*_4_-vincoside (*d*_4_-**2b**) feeding results can be found in Supplementary Fig. 11. EIC, extracted ion chromatogram.

Kratom tissues were subjected to metabolic profiling. A diversity of alkaloids, including 3*S-*MIAs (for example, (20*S*)-corynantheidine (**3a**) and mitragynine (**5a**)) as well as 3*R*-MIAs (for example, speciociliatine (**7a**)), were found in young leaves (Supplementary Figs. 3 and 4). In contrast, mature leaves contained primarily 3*S*-MIAs (Supplementary Fig. 5). Stems and roots contained greater amounts of 3*R*-MIAs and spirooxindole alkaloids (Supplementary Figs. 6 and 7). In young leaf extracts, we noticed two especially abundant peaks (*m*/*z* (M)^+^ = 367 and *m*/*z* (M)^+^ = 397) that did not match any available MIA standard (Fig. 2a). Because these masses were consistent with an oxidized MIA derivative, we incubated the young leaf extract with the reducing agent NaBH_4_, which resulted in efficient conversion to either corynantheidine (**3a**) or mitragynine (**5a**). Compounds purified from young leaf extracts were shown to be the iminium species (20*S*)-3-dehydrocorynantheidine (DHC) (**13a**) and 3-dehydromitragynine (DHM) (**14a**) by NMR analysis (Fig. 2a and Supplementary Fig. 8). Both iminium species showed minimal decomposition during purification and were stereoselectively reduced by NaBH_4_ to the respective 3*S* epimers (Supplementary Fig. 8). This stereoselective NaBH_4_ reduction of corynanthe-type iminium species to the 3*S* isomers was previously observed in ref. ^27^. DHM (**14a**) had been previously reported to be present in kratom leaves^28^, but DHC (**13a**) has not been previously reported. The presence of these compounds led us to hypothesize that 3*R*-MIAs could be formed via stereoselective reduction of one or both of these iminium species.

### Isotopologue feeding of metabolically active kratom tissue

Kratom MIA biosynthesis appears to involve the transport of MIA intermediates between tissues^23^, a scenario that complicates the discovery of biosynthetic genes. Known pathway enzymes are preferentially expressed in roots^9^, although mitragynine (**5a**) and related isomers accumulate predominantly in leaves and stems. To deconvolute metabolite and gene locations, we fed labeled MIA isotopologues to cuttings of kratom tissues—young/mature leaf disks, green stem cuttings and cut roots (Fig. 2b and Supplementary Tables 1 and 2). Young leaves and stems produced substantial levels of labeled MIAs from *d*_5_-tryptamine (*d*_5_-1; Fig. 2b and Supplementary Figs. 9 and 10), most notably labeled mitragynine (**5a**, 3*S*) and speciociliatine (**7a**, 3*R*), demonstrating that these tissues harbor the genes responsible for establishing the 3*R* stereochemistry. Interestingly, some metabolites, such as hirsutine (**6b**, 3*R*), were not detected in untreated stem tissue but were observed after isotopologue feeding, highlighting potential discrepancies between gene expression and native metabolite profiles. Mature leaves contained large amounts of mitragynine (**5a**, 3*S*) and corynantheidine oxindoles (**8a/8b**, 3*R* derived), but labeled substrates were not incorporated to significant levels in this tissue. Roots primarily contained speciociliatine (**7a**, 3*R*) and likewise showed minimal isotopologue incorporation. Feeding of *d*_4_-strictosidine (*d*_4_-**2a**, 3*S*) resulted in similar incorporation as observed for *d*_*5*_-tryptamine (*d*_5_-**1**), but with lowered efficiency (Supplementary Fig. 11). The 3*R* epimer of strictosidine, *d*_4_-vincoside (*d*_4_-**2b**, 3*R*), was not incorporated in any tissue, suggesting that epimerization happens at a later biosynthetic stage. Feeding of *d*_2_-9-OMe-strictosidine (*d*_2_-**9OS**, 3*S*) produced labeled mitragynine (**5a**, 3*S*) and speciogynine (**5b**, 3*S*) in both stem and young leaf (Fig. 2b). However, only small amounts of labeled speciociliatine (**7a**, 3*R*) were observed (stems), suggesting that epimerization does not occur efficiently on C9 methoxylated intermediates. Altogether, these data suggest that epimerization occurs in young leaves after the formation of strictosidine (**2a**) but before methoxylation at C9. Along with the identification of the iminium moiety DHC (**13a**), these observations led us to hypothesize that corynantheidine (**3a**) could be a primary substrate for epimerization.

### Identification and characterization of epimerase genes

**Fig. 3:**
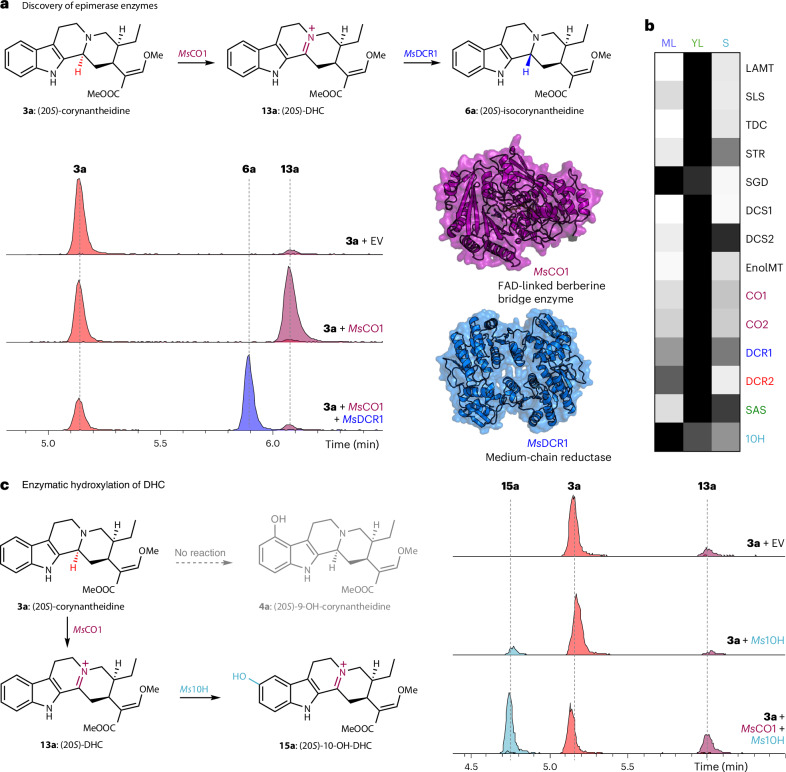
Identification of kratom biosynthetic enzymes. **a**, *Ms*CO1 and *Ms*DCR1 epimerize (20*S*)-corynantheidine (**3a**, 3*S*) to (20*S*)-isocorynantheidine (**6a**, 3*R*) via iminium intermediate **13a**. Enzyme activities were assessed via agroinfiltration in *N. benthamiana*. Assay conditions: 16 h 100 μM **3a** incubation in *N. benthamiana* leaves infiltrated with indicated gene mixes. The shown data are representative spectra from biological replicates (*n* = 3). Compound peaks were determined via EIC from expected *m*/*z* values. Protein structures generated using AlphaFold3. **b**, Coexpression matrix of kratom pathway enzymes in ML, YL and S. **c**, Activity of a herein-discovered CYP81, *Ms*10H, hydroxylates (20*S*)*-*DHC to form **15a**. Assay conditions: 16 h 100 μM **3a** incubation in *N. benthamiana* leaves infiltrated with indicated gene mixes. The shown data are representative spectra from biological replicates (*n* = 3). ML, mature leaves; YL, young leaves; S, stems.

Using a coupled feeding/RNA extraction procedure, we extracted RNA from young leaf and green stem tissue that showed robust conversion of *d*_5_-tryptamine (*d*_5_-**1**) to speciociliatine (**7a**, 3*R*). For comparison, we also isolated RNA from mature leaf tissue that did not incorporate *d*_5_-tryptamine (*d*_5_-**1**). From the resultant RNA-sequencing (RNA-seq) data, we searched for gene candidates that could be responsible for the oxidation of the proposed substrate, (20*S*)-corynantheidine (**3a**), to the iminium species DHC (**13a**). We noted that in the biosynthetically unrelated alkaloid morphine, isomerization of a key stereocenter of the tetrahydroisoquinoline scaffold also occurs via formation and reduction of an iminium intermediate (from *S*-reticuline via dehydroreticuline to *R*-reticuline). In this case, a fusion protein consisting of a cytochrome P450 linked to an aldo-keto reductase performs both the oxidation and reduction^29,30^. Taking inspiration from this transformation, we targeted genes encoding cytochromes P450 that were coexpressed with the gene encoding *Ms*EnolMT in young leaves, although gene candidates annotated as polyphenol oxidases and berberine bridge enzyme (BBE)-like enzymes were also considered. Oxidase gene candidates were agroinfiltrated into *Nicotiana benthamiana* along with (20*S*)-corynantheidine (**3a**). This screening approach resulted in the identification of two BBE-like enzymes that catalyzed the oxidation of (20*S*)-corynantheidine (**3a**) to DHC (**13a**) (Fig. 3a and Supplementary Fig. 12a). No activity was observed when mitragynine was used as a substrate (Supplementary Fig. 13), suggesting an alternate route to DHM (**14a**) production in leaves (Fig. 3b). Thus, these enzymes were termed corynantheidine oxidases 1–2 (CO1 and CO2). *Ms*CO1 and *Ms*CO2 share 94% sequence identity and were coexpressed with *Ms*EnolMT in young leaf tissue (Fig. 3b and Supplementary Fig. 12b). No differences in substrate specificity were noted among these isoforms from in planta assays; therefore, *Ms*CO1 was used for all subsequent assays. Negative (empty vector (EV)) controls indicated that native *N. benthamiana* enzymes could oxidize **3a** to **13a**, although with low efficiency (Fig. 3a). Expression and purification of *Ms*CO1 from *N. benthamiana* leaves additionally allowed for verification of oxidase activity in vitro (Supplementary Figs. 14 and 15).

Reductase candidates were initially chosen based on coexpression with *Ms*CO1 and/or homology to known iminium reductases (aldo-keto reductases and alcohol dehydrogenases—*Ms*DCS1, *Cr*THAS1 and *Cr*DPAS^31^), but no enzyme that reduced **13a** to (20*S*)-isocorynantheidine (**6a**) could be identified. We then compared the reductase genes that are conserved among the known 3*R*-MIA-producing species of the Naucleeae tribe of Rubiaceae (kratom, *Uncaria guianensis*, and *Uncaria rhynchophylla*) with the reductases that are found in nonproducers outside of the Naucleeae (Rubiaceae—*Cinchona pubescens* and *Coffea arabica*; Apocynaceae—*Catharanthus roseus*). Sequence similarity networks (SSNs) are a useful visualization tool for analysis of related sequences, and we hypothesized that Naucleeae-specific clusters (NSCs) could contain the reductase responsible for stereoselectively reducing DHC (**13a**). To this aim, we built an SSN with every annotated kratom reductase/dehydrogenase/methyltransferase and the closest homolog found in each of the aforementioned plant transcriptomes. Through increasing the stringency of the node alignment threshold, we observed the emergence of NSCs containing kratom gene candidates. As a control, we tested this approach using the known Naucleeae pathway-specific methyltransferase gene, *Ms*EnolMT, which was observed to indeed be localized in an NSC (Supplementary Fig. 16). Encouraged, we identified eight reductase candidates that appeared in NSCs (Supplementary Fig. 17). Two of these candidates were found to be active on **13a** and were annotated as isoflavone reductases. One reduced DHC (**13a**) to the 3*R* isomer (20*S*)-isocorynantheidine (**6a**) and was designated as DHC reductase 1 (*Ms*DCR1), while the other (93% sequence identity) reduced **13a** to the 3*S* isomer (20*S*)-corynantheidine (**3a**) and was designated as *Ms*DCR2 (Fig. 3a and Supplementary Fig. 18). These enzymes belong to the class of lignan aromatic alcohol dehydrogenases^32^ and share high sequence identity (~70%) to isoflavone reductase in *C. arabica* (Supplementary Fig. 18b). Notably, these enzymes are not related to the other known kratom MIA iminium reductases, *Ms*DCS1 (<15% amino acid sequence identity). Although *Ms*DCR1 and *Ms*DCR2 were only identified after SSN analysis, these genes have a similar expression pattern with the upstream pathway (Fig. 3b). Both *Ms*CO1 and *Ms*DCR1/*Ms*DCR2 could be heterologously expressed and purified, and we verified activities on both **13a** and **14a** from in vitro assays (Supplementary Figs. 19–21).

### Mutagenesis and docking studies of *Ms*CO1 and *Ms*DCR isoforms

**Fig. 4:**
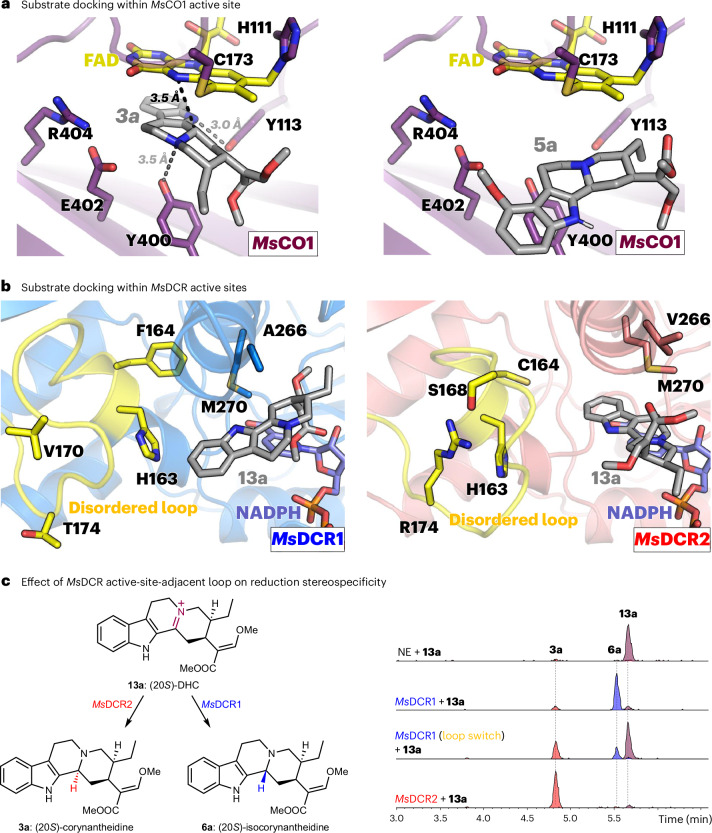
Structural analyses of *Ms*CO1 and *Ms*DCRs. **a**, Results from docked **3a** or **5a** substrates within the *Ms*CO1 active site (model generated via AlphaFold3). **b**, Docking results of *Ms*DCR1 and *Ms*DCR2 with substrate **13a** (models generated via AlphaFold3). The disordered loop region adjacent to the active site is highlighted in yellow. **c**, In vitro assay detailing the effect of replacing the loop region of *Ms*DCR1 with that from *Ms*DCR2 on reduction stereoselectivity. Reaction conditions: 100 μM **13a**, 100 μM NADPH, 1 μM *Ms*DCR isoform, 50 mM HEPES (pH = 7.5), 25 °C for 4 h. Shown data are representative spectra from biological replicates (*n* = 3).

We used AlphaFold^33^ modeling to investigate the molecular basis of substrate specificity of *Ms*CO1, which accepts (20*S*)-corynantheidine (**3a**) but not mitragynine (**5a**) (Fig. 4a). *Ms*CO1 is predicted to be a BBE-like flavin adenine dinucleotide (FAD)-linked oxidase, and modeling implicated His111 and Cys173 in the covalent tethering of the cofactor within the enzyme active site. Docking of **3a** close to the FAD (Fig. 4a) resulted in a close (3.5 Å) distance of C3 to the FAD nitrogen. Nearby residues, such as Y400, E402 or Y113, are likely involved in substrate binding. However, alanine scanning of five active site residues failed to significantly impact the activity of *Ms*CO1 (Supplementary Fig. 22). We note that docking of **5a** revealed that this substrate appears to be unable to bind productively in the active site, presumably due to the added steric bulk of the methoxy group (Fig. 4a). The 9-hydroxylated analog, **4a**, was found to bind similarly to **3a**, but with close hydrogen bonds to E402 (3.3 Å) and R404 (3.6 Å; Supplementary Fig. 22). These results support experimental evidence that *Ms*CO1 has no detectable activity on **5a**.

AlphaFold3 modeling of *Ms*DCR isoforms revealed near-complete conservation of the predicted active site residues between *Ms*DCR1 and *Ms*DCR2 (Supplementary Fig. 18c). However, docking of **13a** within the respective active sites revealed key differences in substrate binding (Fig. 4b). *Ms*DCR1 was predicted to bind **13a** with a pro-R orientation with a 7.1 Å distance between C3 and NADPH, while *Ms*DCR2 was predicted to bind 13a with a pro-S orientation 4.0 Å away from the NADPH. Mutagenesis of active-site residues of MsDCR1 revealed mutation of two residues, E116 and Y131, diminished enzyme activity (Supplementary Fig. 23). Focusing on differences between *Ms*DCR1 and *Ms*DCR2, we noticed one loop that is situated adjacent to the active site that contains almost a third of the residues that are mutated between the two reductases. We used mutagenesis to interchange the loop region of *Ms*DCR1 with that from *Ms*DCR2 and tested the activity of this protein in vitro (Fig. 4c and Supplementary Fig. 24). *Ms*DCR1 with the loop region of *Ms*DCR2 indeed showed altered stereoselectivity, preferentially forming the 3*S* isomer **3a** over the 3*R* isomer **6a**, albeit with decreased overall activity. These results highlight the importance of the loop structure in substrate orientation.

### Iminium intermediate 13a is a substrate for 10-hydroxylation

We envisioned that SSN analysis for all annotated kratom oxidases/P450s and the closest homologs in related Rubiaceae and Apocynaceae species would be a viable strategy to uncover the hydroxylase hypothesized to be involved in downstream MIA biosynthesis. Because 9- and 10-hydroxylation has been reported in kratom and *Uncaria* species^34^, we targeted candidates in NSCs along with kratom-specific clusters (Supplementary Fig. 25). Although no candidate gene hydroxylated (20*S*)-corynantheidine (**3a**), one CYP81, identified from a NSC, catalyzed an unexpected 10-hydroxylation on **13a** to form **15a**, as verified by an authentic 10-hydroxycorynantheidine (**16a**) standard (Supplementary Fig. 26). This enzyme showed no activity on other kratom metabolites. The gene encoding this enzyme, here termed *Ms*10H, was expressed primarily in mature leaves. No 10-hydroxylated MIAs have been observed in kratom, and the corresponding **15a** and **16a** peaks do not correspond to any observed compounds from kratom extracts. The in planta role of *Ms*10H in kratom MIA biosynthesis, therefore, remains unclear. Nevertheless, this discovery highlights that the iminium intermediate **13a** can serve as a substrate for downstream pathway steps.

### Substrate activity assays for *Ms*CO1, *Ms*DCR1 and *Ms*SAS

**Fig. 5:**
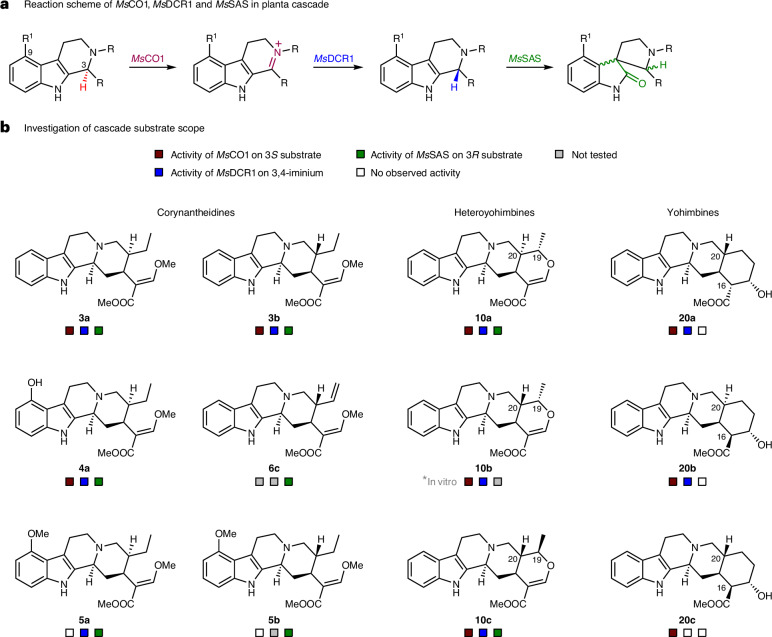
Cascade substrate scope of the late-stage kratom biosynthetic pathway. **a**, Reaction scheme for *Ms*CO1, *Ms*DCR1 and *Ms*SAS cascades. **b**, Colored squares indicate observed transformation for a given substrate, an empty square indicates that no reaction was observed and a gray square indicates that a given transformation was not tested. Assay conditions: 36 h incubation of 100 μM of the shown substrates in *N. benthamiana* leaves infiltrated with indicated gene mixes. An asterisk indicates that for ajmalicine (**10b**), activity was observed in vitro but not in planta (Supplementary Fig. 27). See Supplementary Figs. 27–38 and 41 for detailed analyses of all tested reactions.

In kratom and related plants, it is hypothesized that only MIAs with 3*R* stereochemistry can be converted to spirooxindole alkaloids. A recently discovered P450, *Ms*SAS (also known as *Ms*3eCIS), was shown to convert hirsutine (**6b**, 3*R*, 20*R*) and hirsuteine (**6c**, 3*R*, 20*R*) to spirooxindole alkaloids **8b** and **8c**^26^, although additional substrates were not tested. We sought to determine the substrate scope of 3*S*-MIA to 3*R*-MIA epimerization, as well as the spirocyclization of the formed 3*R*-MIAs. To this aim, we assayed a variety of substrates with *Ms*CO1, *Ms*DCR1 and *Ms*SAS in *N. benthamiana* (Fig. 5 and Supplementary Figs. 27–38 and 41) to assess how these enzymes functioned in planta.

Hirsuteine (**6c**) has previously been reported to be a substrate for *Ms*SAS^26^, and reaction in planta with this substrate resulted in the following two 3*S* spirocyclic products: isocorynoxeine (**8c**_**i**_) and corynoxeine (**8c**_**ii**_) (Fig. 5b and Supplementary Fig. 28). We then tested the activity of *Ms*SAS in the context of the upstream enzymes *Ms*CO1 and *Ms*DCR1 on (20*S*)-corynantheidine (**3a**) and found that this substrate was converted to a mixture of three corynoxine isomers (**8a**), including corynoxine A (**8a**_**i**_) and corynoxine B (**8a**_**ii**_) (Supplementary Fig. 29). A compound putatively assigned as 3-epicorynoxine (**8a**_**iii**_**/8a**_**iv**_**)** was also observed as a major product. Consistent with previous reports of spirooxindole alkaloid interconversion, we observed that produced corynantheidine oxindoles (and standards) spontaneously isomerize^35^. Lack of availability of (20*R*)-corynantheidine (**3b**) precluded direct assay of this substrate, although activity on this compound was verified via pathway reconstitution (see below; Supplementary Fig. 41).

(20*S*)-9-Hydroxycorynantheidine (**4a**), a presumed intermediate en route to mitragynine (**5a**), appeared to be readily oxidized by *Ms*CO1, but only traces of reduced product were observed with the subsequent reaction with *Ms*DCR1 (Supplementary Fig. 30). Neither mitragynine (**5a**, 20*S*) nor speciogynine (**5b**, 20*R*) were oxidized by *Ms*CO1, but DHM (**14a**) was reduced via *Ms*DCR1 to speciociliatine (**7a** (3*R*, 20*S*); Supplementary Fig. 31). Both speciociliatine (**7a**) and mitraciliatine (**7b**) act as substrates for *Ms*SAS, forming compounds with mass fragmentation patterns consistent with mitragynine oxindoles (**9a**) and speciogynine oxindoles (**9b**), respectively (Supplementary Figs. 31 and 32). Peaks co-eluting with these observed methoxylated spirooxindole alkaloid products were highly abundant in stem and root tissue.

Heteroyohimbine-type MIAs such as tetrahydroalstonine (THA) (**10a**) and ajmalicine (**10b**) are also observed in kratom (Fig. 2). When *Ms*CO1, *Ms*DCR1 and *Ms*SAS were assayed with THA (**10a**), we observed the formation of minor amounts of isopteropodine (**12a**_**i**_) and pteropodine (**12a**_**ii**_), with the major peak likely corresponding to one of the two possible 3*R* **12a** isomers (3-epipteropodine; Supplementary Fig. 33). Additional compounds assigned as 3-dehydro-THA (**19a**) and iso-THA (**11a**) were also observed. In contrast, *Ms*CO1 incubation with ajmalicine (**10b**) did not result in observable product formation in planta (Supplementary Fig. 34a), although *Ms*CO1 and *Ms*DCR1 were shown to be active on ajmalicine (**10b**) in vitro, forming isoajmalicine (**11b**) (Supplementary Fig. 34b). The related heteroyohimbine mayumbine (**10c**) was also carried through the cascade, resulting in the formation of compounds that could be putatively assigned as mayumbine oxindoles (**12c**), alongside intermediates 3-dehydromayumbine (**19c**) and isomayumbine (**11c**) (Supplementary Fig. 35).

Finally, we tested the activity of *Ms*CO1, *Ms*DCR1 and *Ms*SAS on yohimbine-type alkaloids, although yohimbanes are not present in kratom. Yohimbine (**20a**) was readily converted by both *Ms*CO1 and *Ms*DCR1 to form pseudoyohimbine (**22a**) (Supplementary Fig. 36). Rauwolscine (**20b**) was similarly isomerized to isorauhimbine (**22b**) (Supplementary Fig. 37), but corynanthine (**20c**) could only be converted to 3-dehydrocorynanthine (**21c**), and no subsequent reduction to isocorynanthine (**22c**) was observed (Supplementary Fig. 38). Interestingly, *Ms*SAS showed no activity on any of these yohimbine-type substrates, highlighting the greater specificity of this P450 for conversion to the spirooxindole alkaloids.

### Pathway construction in *N. benthamiana* from tryptamine

Finally, we reconstituted the full pathway from tryptamine (**1**) to spirooxindole alkaloids (**8a/8b**) by coexpressing kratom pathway enzymes in *N. benthamiana* (*Ms*STR, *Cr*SGD, *Ms*DCS1/*Cp*DCS, *Ms*EnolMT, *Ms*CO1, *Ms*DCR1 and *Ms*SAS), along with infiltration of the starting substrates tryptamine (**1**) and secologanin. Successful stepwise formation of all 20*S* and 20*R* isomers leading to the respective corynantheidine oxindoles (**8a/8b**) was observed (Supplementary Fig. 39). When *Ms*DCS1, which is selective for 20*S* products, was used, corynoxine (**8a**) isomers were observed (Supplementary Fig. 40). To obtain the 20*R* series, we used the dihydrocorynantheine synthase reductase from *C. pubescens*, *Cp*DCS, which selectively produces this epimer^36^. In this cascade, only two 20*R* products were noted, both with 3*S* stereochemistry corresponding to standards of isorhynchophylline (**8b**_**i**_) and rhynchophylline (**8b**_**ii**_) (Supplementary Fig. 41). Therefore, (20*R*)-corynantheidine (**3b**) also acts as a substrate for *Ms*CO1, *Ms*DCR1 and *Ms*SAS (Fig. 5b).

## Discussion

The mechanisms by which the 3*R* stereocenter is generated in MIA biosynthesis were unknown at the outset of this study. Here we show that kratom (Rubiaceae family, Naucleeae tribe) evolved an oxidase/reductase pair to epimerize a variety of corynanthe-, heteroyohimbine-, and yohimbine-type *3S*-MIAs to the corresponding 3*R-*MIAs. An analogous strategy of epimerization following a stereoselective Pictet–Spengler reaction has been previously characterized for biosynthesis of the tetrahydroisoquinoline alkaloid morphine. This morphine biosynthetic enzyme, reticuline epimerase, a fused P450-AKR enzyme, carries out both oxidation and reduction. Fusing the epimerase in this way is proposed to limit the reactivity of the dehydroreticuline iminium intermediate and increase pathway flux^29,30^. Additionally, two recent preprints have shown that in *Rauvolfia serpentina* (Apocynaceae family), the biosynthetic intermediate rauwolscine **20b** is racemized to isorauhimbine **22b** by a similar redox mechanism^37,38^. Although the *Rauvolfia* oxidase is distantly related to *Ms*CO1, the corresponding reductase belongs to a different class of reductases than *Ms*DCR1/*Ms*DCR2. In kratom, we show that the intermediate iminium species are surprisingly long-lived and decoupled from subsequent reduction, accumulating to a high degree in growing leaf tissue. *Ms*DCR1 and *Ms*SAS are expressed at higher levels in stem than in other pathway genes, suggesting that iminium species could be synthesized in young leaves and then transported to the stems/roots for further biosynthetic transformation. In support of this hypothesis, both roots and stems primarily accumulate 3*R*-MIAs and downstream 3*R*-derived spirooxindole MIAs, while leaves contain primarily 3*S*-MIAs and iminium MIAs.

**Fig. 6:**
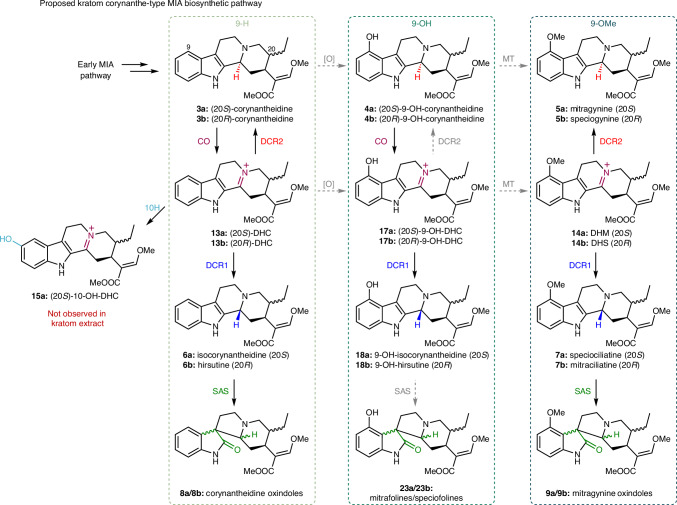
Proposed kratom corynanthe-type MIA biosynthetic pathway from corynantheidine. Unknown or hypothesized transformations are depicted in gray.

Iminium formation from BBE-like enzymes has been previously observed. In a related MIA biosynthetic pathway in *C. roseus*, the BBE-like enzyme *Cr*PAS (54% identity) produces the conjugated iminium precondylocarpine acetate^39^. The herein-reported hydroxylase *Ms*10H has substrate specificity for DHC (**13a**), acting only on this iminium species (**13a**) and not on (20*S*)-corynantheidine (**3a**). Alongside the high abundance of DHC (**13a**) and DHM (**14a**) in leaf tissue (and presence of hydroxylated DHC (**17a**) (Supplementary Fig. 42)), these data raise the possibility that DHC (**13a**) acts as a central kratom metabolite and branchpoint of kratom MIA biosynthesis (Fig. 6). We hypothesize that **13a** could be converted to DHM (**14a**), which would then be reduced by *Ms*DCR2 to mitragynine (**5a**, 3*S*), although despite extensive screening, we have not yet identified an enzyme that catalyzes 9-hydroxylation of **13a**.

*Ms*DCR1 and *Ms*DCR2 catalyze well-precedented 1,2-iminium reductions but are not related to other known MIA-associated iminium reductases, *Cr*THAS1, *Ms*DCS1, *Cp*DCS or *Cr*DPAS (<15% sequence identity). The most closely related sequences to *Ms*DCR isoforms are isoflavone reductase-like enzymes, known to catalyze the reduction of alkenes in isoflavonoid biosynthesis (~70% sequence identity)^32^. This class of reductase has not been previously shown to reduce iminium species. The relatively broad substrate scope of the *Ms*CO1/*Ms*DCR1 pair will be useful for the formation of intermediates of other known 3*R*-MIA biosynthetic pathways. Notably, yohimbine-type alkaloids could also be epimerized by these enzymes, although these pharmacologically important alkaloids are not produced by kratom. Along with the recently reported yohimbine synthase from *Rauvolfia tetraphylla*^40^, biocatalytic production of 3*R* yohimbanes, such as pseudoyohimbine (**22a**) and isorauhimbine (**22b**), is now possible.

Finally, the establishment of enzymatic production of numerous 3*R*-MIAs allowed the assessment of the activity of spirocyclase CYP71, *Ms*SAS^26^. We showed that *Ms*SAS turns over all tested 3*R* corynanthe- and heteroyohimbine-type MIAs but remains inactive on yohimbane scaffolds. We reconstituted the biosyntheses of spirooxindoles corynoxine (**8a**) and rhynchophylline (**8b**) from tryptamine. Additionally, both speciociliatine (**7a**) and mitraciliatine (**7b**) are spirocyclized via this enzyme, indicating that the presence of the 9-methoxy group does not inhibit catalytic activity. When 3*R*-MIAs with 20*R* stereochemistry (hirsutine (**6b**), hirsuteine (**6c**) and isomayumbine (**11c**)) are used as substrates, two 3*S* spirooxindole alkaloid products are formed (Supplementary Fig. 43). Conversely, substrates with 20*S* stereochemistry, such as (20*S*)-corynantheidine (**3a**) or THA (**10a**), are converted into a more diverse product mixture comprising both 3*R* and 3*S* geometries (Supplementary Fig. 44). This is consistent with previous studies and energy calculations that have confirmed the effect of ring stereochemistry on product distribution resulting from isomerization (Supplementary Table 3)^35,41^. Notably, for both 20*S* and 20*R* geometries, the thermodynamic equilibrium favors 3*S* spirocyclic products, meaning the formation of (3*R*, 20*S*) spirooxindole alkaloids is due to the stereochemical control of *Ms*SAS. Our data, therefore, show that the *Ms*SAS active site is able to promote the formation of otherwise thermodynamically unfavorable spirocyclic products with 3*R* geometries.

3*R*-MIAs such as hirsutine (**6b**) and speciociliatine (**7a**) have long been recognized as having valuable bioactivities, but the biosynthetic pathways for these compounds have remained cryptic. Labeled substrate feeding, isolation of intermediates and bioinformatic analysis of RNA-seq data led to the discovery of two enzymes, a BBE-like FAD-linked oxidase, *Ms*CO1, and an iminium reductase, *Ms*DCR1, that act together to epimerize 3*S*-MIAs to 3*R*-MIAs in *M. speciosa* (kratom). The resulting 3R-MIAs can also be combined with the previously discovered *Ms*SAS to form spiroxindole alkaloids. The wide substrate specificity of this two-enzyme cascade expands the available stereochemical space of MIAs, thereby substantially improving access to these medicinally relevant compounds. Moreover, the discovery of reductases having both 3*S* (*Ms*DCR1) and 3*R* (*Ms*DCR2) stereoselectivity suggests that iminium species DHC (**13a**) could serve as an intermediate for a wide range of alkaloids in kratom.

## Methods

### Plants and plant growth

*M. speciosa ‘*Green Thai*’* plants were a generous gift from S.S. Nadakuduti (University of Florida) and were originally purchased from a supplier in Thailand. Plants were kept on a standard soil mix in the greenhouse (Jena, Germany) at 24–32 °C during the day and 22–24 °C during the night (summer) and at 18–23 °C during the day and 18–20 °C during the night (winter). Relative humidity was kept between 60% and 80%. Plants were propagated via cuttings.

*M. speciosa ‘*Rifat*’* plants were grown on a standard soil mix in the greenhouse (Athens, GA). Culture conditions were set to 28 °C (day) and 19 °C (night) following a 15-h light/9-h dark photoperiod with 23 daily light intervals.

*N. benthamiana* plants were grown on a standard soil mix in the greenhouse. Culture conditions were set to 22 °C, 60% relative humidity and followed a 16-h light/8-h dark photoperiod. Tobacco plants were usually grown for at least 3 weeks but no longer than 4 weeks before infiltration with *Agrobacterium tumefaciens* GV3101. Plant watering was performed as needed.

### Chemicals

All chemicals used in this study were purchased as molecular biology grade or higher from commercial vendors (Sigma-Aldrich, Thermo Fisher Scientific, etc.) unless denoted differently. Kratom alkaloid standards were obtained from the following sources: mitragynine (**5a**) was obtained from Biosynth; speciogynine (**5b**), paynantheine (**5c**), hirsutine (**6b**), hirsuteine (**6c**), speciociliatine (**7a**), corynoxine A (**8a**_i_), corynoxine B (**8a**_ii_), rhynchophylline (**8b**_ii_) and isorhynchophylline (**8b**_i_) were obtained from Cayman Chemical. Yohimbine (**20a**), rauwolscine (**20b**) and corynanthine (**20c**) were obtained from Extrasynthese. Isopteropodine (**12a**_i_) and pteropodine (**12a**_ii_) were purchased from Phytolab. Corynoxeine (**8c**_ii_) and isocorynoxeine (**8c**_i_) were purchased from MedChemExpress. Mitraphylline (**12b**_ii_), (20*S*)-9-hydroxycorynantheidine (**4a**) and (20*S*)-corynantheidine (**3a**) were kindly gifted to us by C. McCurdy, and isoajmalicine (**11b**) and isomitraphylline (**12b**_i_) were provided by A. Lopez. (20*R*)-corynantheidine (**3b**), mitraciliatine (**7b**) and (20*S*)-isocorynantheidine (**3b**) standards were isolated from Rifat kratom leaf tissue provided by C.R. Buell. Unless otherwise noted, all reagents were obtained from commercial sources and used without any further purification. Thin-layer chromatography and preparative thin-layer chromatography were carried out on aluminum-backed silica gel 60 F254 plates (Merck Millipore) and visualized using UV254 nm light detection.

### Isotopologue feeding of *M. speciosa* tissue

Tissue samples of *M. speciosa* were collected from recently pruned, 4-year-old plants during winter (pruning greatly increased the availability of fresh young leaf tissue). Samples for metabolomic analysis were freshly extracted in MeOH before liquid chromatography–mass spectrometry (LC–MS) analysis. Samples for isotopologue feeding were prepared in the following way: 5 mg leaf disks, 5 mm cut stem disks or 5 mg cut roots were obtained fresh from plants. These samples were incubated with 200 μl H_2_O, 1 mM *d*_5_-trypamine (*d*_5_-**1**, *d*_4_-strictosidine (*d*_4_-**2a**), *d*_4_-vincoside (*d*_4_-**2b**), *d*_2_-9-methoxystrictosidine (*d*_2_-**9OS**) or *d*_4_-serotonin for 24 h at 25 °C. Any remaining liquid was removed, and the samples were extracted for 1 h with 500 ml MeOH, filtered and then analyzed via LC–MS. The remaining issue was immediately snap-frozen in liquid nitrogen and stored at −80 °C for indefinite periods. For samples with positive feeding results (or negative for mature leaves and roots), the noncut material was used for subsequent RNA extraction and RNA-seq.

### RNA purification and sequencing

Total RNA of *M. speciosa* (roots, stem, young leaves and mature leaves) was extracted using the RNeasy Mini Kit (Qiagen) according to the manufacturer’s instructions with additional on-column DNAse incubation in biological triplicate. The quality of the obtained RNA was analyzed using an Implen NanoPhotometer N60. All samples except roots satisfied the necessary requirements for total RNA-seq (≥100 ng; A260/280 = 1.8–2.2; A260/230 ≥ 1.8) and were submitted to Novogene (https://en.novogene.com/) for total RNA-seq using the company’s standard protocols for library preparation and RNA-Seq. In total, ≥30 million raw sequencing reads (Illumina; 150 bp paired-end) were acquired per sample. PacBio de novo transcriptome assembly was prepared from a pooling of the abovementioned RNA samples using the PacBio SMRT platform (PacBio Sequel II CSS mode, single end).

### Coexpression/homology analysis for gene discovery

The abovementioned PacBio assembled transcriptome was used for transcript analysis with predicted functions annotated via BLASTing to the SwissProt database. Fragments per kilobase of transcript per million counts were used to evaluate transcript expression abundances. Pearson correlation coefficients were calculated using Microsoft Excel using the expression profile of *Ms*EnolMT or *Ms*CO1. For reductase candidates, annotated reductases/dehydrogenases with high homology to *Cr*THAS1 and *Ms*DCS1 were chosen as candidates for screening.

### Identification of closest homologs to kratom genes in available transcriptomes and SSN analysis

#### Methyltransferases

Based on SwissProt gene annotation, the amino acid sequences of all kratom transcripts in the PacBio assembly that were annotated as a ‘methyltransferase’ were obtained (205 transcripts). Using an in-house Python script, for each of five additional transcriptomes (*U*. *rhynchophylla*, *U*. *guianensis*, *C*. *pubescens*, *C*. *arabica* and *C. roseus*), the open reading frame of every transcript (starting with ATG) with a minimum amino acid length of 100 residues was translated. The peptides were then aligned to the kratom methyltransferase list, and the gene from each transcriptome with the highest scoring alignment (using Python Bio.Align’s scoring metric) was obtained as a peptide sequence. These genes, along with the list of kratom genes, were aligned using the enzyme function initiative-enzyme similarity tool (EFI-EST) server (https://efi.igb.illinois.edu/efi-est/)^42^. Clusters were generated with an initial alignment score threshold of 50. Using CytoScape, clusters were visualized, and the alignment score threshold increased until clusters not containing *C. roseus*, *C. arabica* or *C. pubescens* genes emerged (NSCs). We observed a clear NSC cluster containing *Ms*EnolMT emerging with an alignment score threshold of 100.

#### Reductases

As detailed above, the amino acid sequences of all kratom transcripts in the PacBio assembly that were annotated as either a ‘reductase’ or ‘dehydrogenase’ were obtained (605 transcripts). Kratom reductase candidates and their closest homologs from the other transcriptomes (see above for details) were then aligned using EFI-EST, and clusters were generated with an initial alignment score threshold of 50. Using CytoScape, clusters were visualized, and the alignment score threshold was increased until NSCs emerged. We started observing NSCs with an alignment score threshold of 145 and increased this threshold at increments of five until eight suitable candidates emerged (see sequences below for additional information). We identified the kratom genes within these clusters as candidates for screening.

#### P450s/oxidases

As detailed above, the amino acid sequences of all kratom transcripts in the PacBio assembly that included keywords ‘P450’, ‘oxidase’, ‘hydroxylase’ or ‘monooxygenase’ were obtained (215 transcripts). Kratom oxidase candidates and their closest homologs from the other transcriptomes (see above for details) were then aligned using EFI-EST, and clusters were generated with an initial alignment score threshold of 50. Using CytoScape, clusters were visualized, and the alignment score threshold was increased until NSCs emerged. We started observing NSCs with an alignment score threshold of 170 and identified the kratom genes within these clusters as candidates for screening. In addition, genes in kratom-specific clusters and unclustered kratom genes were also possible candidates for screening.

### Cloning of gene candidates

Primers containing overhanging ends homologous to a modified 3Ω1 vector^43^ were used to amplify full-length genes via PCR. *M. speciosa* cDNA from either the stem or the young leaf was used as the template. Resultant amplicons were purified via gel electrophoresis. Empty 3Ω1 vector was digested via BsaI (Thermo Fisher Scientific) and purified via the DNA Clean & Concentrator-5 (Zymo) kit. In-Fusion cloning (Clontech Takara; manufacturer’s instructions) was used to fuse the gene of interest with the assembled final plasmid. The in-fusion crude reaction was transformed into chemically competent *Escherichia coli* TOP10 cells (Thermo Fisher Scientific) and plated onto LB agar supplemented with spectinomycin (200 μg ml^−1^) and incubated at 37 °C for 16 h. Plasmids of positive transformants were isolated from overnight cultures (37 °C, 225 rpm, 2 ml LB + spectinomycin) and correct cloning was confirmed by Sanger sequencing (Azenta Life Sciences).

### Transformation of *A. tumefaciens* GV3101

Electrocompetent cells of *A. tumefaciens* GV3101 (GoldBio) were thawed on ice and mixed with plasmid DNA (~500 ng) that had been verified by Sanger sequencing. Immediately post-thawing, the cell suspension was transferred to a prechilled electroporation cuvette, and cells were electroporated using a MicroPulser (Bio-Rad) at 2.2 kV. Cells were mixed with 0.6 ml LB medium and recovered at 28 °C at 225 rpm for 3 h before plating on selective LB agar plates (supplemented with 20 μg ml^−1^ rifampicin, 50 μg ml^−1^ gentamycin and 200 μg ml^−1^ spectinomycin). Plates were kept at 28 °C for 2 days. Single colonies were used to inoculate liquid cultures. Liquid cultures were prepared as 5 ml cultures (supplemented with 20 μg ml^−1^ rifampicin, 50 μg ml^−1^ gentamycin and 200 μg ml^−1^ spectinomycin) and cultivated at 28 °C and 250 rpm for up to 24 h. In total, 50% glycerol stocks were prepared thereof, snap-frozen in liquid nitrogen and stored at −80 °C indefinitely. The remaining culture was then used for transient expression in *N. benthamiana*.

### Transient expression of gene candidates in *N. benthamiana*

Transient expression of gene candidates in *N. benthamiana* was performed as previously reported in ref. ^44^. The cells containing the gene of interest in the abovementioned cultures were collected by centrifugation (4,000*g* for 5 min). Cells were resuspended in 5 ml infiltration buffer (27.8 mM glucose, 100 μM acetosyringone, 50 mM MES and 2 mM Na_3_PO_4_ (pH = 6.0)) to an optical density (OD)_600_ of ~0.6. Upon infiltration of multiple Agrobacterium strains, the strains were diluted so that the final OD_600_ was <1 (equal concentration for each strain). Resulting suspensions were incubated at 25 °C for 1 h and then infiltrated into the underside of 3–4-week-old *N. benthamiana* leaves using a needleless 1 ml syringe. After 2 days, the substrate(s) were infiltrated (50–100 μl) into the underside of the same leaves previously infiltrated with the Agrobacterium strains of choice in a predemarcated area. Substrate concentrations were 100 μM or 500 μM (tryptamines + secologanin). Each individual infiltration experiment was tested at least twice for candidate screening and thrice for activity characterization, with biological replicates consisting of leaves from different tobacco plants. At 2 days postinfiltration, 5 mg leaf disks were excised from the sites of substrate injection. Leaf disks were extracted in 400 μl MeOH for 1 h at 300 rpm. The MeOH supernatant was subsequently filtered through 0.45 μm low-binding hydrophilic polytetrafluoroethylene spin-filter plates (Millipore). Filtered samples were directly analyzed by high-resolution LC–MS, and individual metabolites were identified based on comparison of retention times and MS2 spectra with authentic standards. DataAnalysis version 5.3 (Bruker) was used to analyze LC–MS data.

### Reduction of kratom iminiums using NaBH_4_

Kratom young leaf disks (5 mg) were extracted with 500 μl MeOH for 16 h at room temperature. The extract was then divided into two equal portions, with 10 mM NaBH_4_ (dissolved in MeOH) added to one. Both extract portions were incubated at 40 °C for 1 h and then quenched via the addition of 20 mM HCl. The extracts were subsequently filtered and analyzed via high-performance LC (HPLC)–MS.

Reduction of isolated iminium standards was performed as follows: 10 mM NaBH_4_ or H_2_O was added to 100 μM (20*S*)-3-DHC or 500 μM DHM and incubated for 1 h at 40 °C and then quenched via the addition of 20 mM HCl. The reactions were then filtered and analyzed via HPLC–MS.

### Isolation of (20*S*)-3-DHC (13a) and DHM (14a) from kratom leaves

Young leaves mixed with some mature leaves (62.2 g) were harvested from multiple kratom plants. The leaves were blended in 500 ml of MeOH, and the resultant slurry was filtered and washed with additional MeOH. The solution was evaporated down to ~50 ml, turning an orange color as the chlorophyll precipitated. This mixture was diluted 1:4 in H_2_O and run through a 10 g C18 SPE column. The column was washed with 10% MeOH until the flow-through became clear (originally pinkish). Alkaloids were eluted with 50% MeOH, resulting in an orange eluent. The MeOH was evaporated, and the resulting aqueous solution was dried via lyophilization.

This oily mass was dissolved in 5 ml of 25% MeOH and injected onto a preparative HPLC for compound isolation with 300 μl injections. Peaks corresponding to (20*S*)-3-DHC (**13a**) and DHM (**14a**) were identified, and the fractions were pooled. These solutions were then dried to obtain 12 mg of crude DHC (**13a**) and 62 mg of crude DHM (**14a**). Because the compounds were not pure, some of these crude mixtures were repurified using semipreparative HPLC, resulting in 0.63 mg of DHC (**13a**) and 2.5 mg of DHM (**14a**). The identities and structures were verified using NMR (DHM (Supplementary Fig. 62) and DHC (Supplementary Figs. 65–68 and Supplementary Table 4)).

### Isolation of alkaloid standards from Rifat kratom tissue

A mixture of dried young and mature leaves (5 g) from Rifat kratom was blended in 200 ml of MeOH, and the resultant slurry was filtered and washed with additional MeOH. The solution was evaporated down to ~5 ml. This mixture was diluted 1:4 in H_2_O and run through a 1 g C18 SPE column. The column was washed with 10% MeOH until the flow-through became clear, and then alkaloids were eluted with 50% MeOH, resulting in an orange flow-through. The MeOH was evaporated, and the resulting aqueous solution was dried via lyophilization. The resulting solution was resuspended in 10% MeOH, and the compounds were purified using semipreparative HPLC. Fractions containing compounds of interest were pooled and dried via lyophilization. The following isolated compounds were obtained: **3a** (0.11 mg), **3b** (0.09 mg), **6a** (0.17 mg), **6b** (0.12 mg), **5b** (0.31 mg), **7a** (0.10 mg), **7b** (0.08 mg) and **7c** (0.12 mg).

### Structure optimization and energy calculations of spirooxindole alkaloids

All calculations were performed using GaussView ver. 6 (Semichem) and Gaussian ver. 16 (Gaussian). Structures were optimized using the semi-empirical method PM6; the resulting structures were used for conformer variation with the GMMX processor of the Gaussian program package. Resulting structures were density functional theory-optimized with Gaussian ver. 16 (B3LYP/6-31G(d), gas phase). The energy of each lowest energy conformer from the density functional theory calculations is shown in Supplementary Table 3.

### Purification of *Ms*CO1 from *N. benthamiana* leaves

For the purification of *Ms*CO1, expression in *E. coli* or *Saccharomyces cerevisiae* failed to yield active enzyme. Therefore, the procedure from ref. ^39^ was adapted for expression and purification from *N. benthamiana*. Using the previously mentioned modified 3Ω1 plasmid backbone, an N-terminal His_6_-tag (see sequence below) was appended to *Ms*CO1. Because native enzymes present in *N. benthamiana* are able to catalyze corynantheidine oxidation (albeit with low efficiency), non-*Ms*CO1 transformed tissue was also investigated as a control (designated EV). Using our standard agroinfiltration/expression strategy, 25 *N. benthamiana* plants were infiltrated with an *A. tumefaciens* GV3101 strain containing this plasmid, and another 25 *N. benthamiana* plants were infiltrated with an *A. tumefaciens* GV3101 strain containing an EV plasmid. After 4 days postinfiltration, infected leaves were harvested, comprising 21.2 g (*Ms*CO1) and 22.5 g (EV). These leaves were homogenized in 100 ml of 50 mM Tris–HCl buffer (pH = 7.5) containing EDTA-free protease inhibitors and 1% insoluble polyvinylpolypyrrolidone (PVPP) using a blender. The homogenates were then filtered, washed with additional buffer and centrifuged at 4000*g* for 10 min to pellet the insoluble PVPP and tissue debris. The supernatants were further clarified by centrifugation at 35,000*g* for 20 min. *Ms*CO1 and the EV lysate were treated identically, with both lysates poured over a 1 ml Ni-NTA column, washed with 20 ml of 50 mM Tris–HCl buffer (pH = 7.5) containing 20 mM imidazole, and eluted with 2.5 ml of 50 mM Tris–HCl buffer (pH = 7.5) buffer containing 250 mM imidazole. The eluted fractions were spin-concentrated/buffer-exchanged using centrifuge filtration with a 30 kDa cutoff (Amicon; Merck Millipore) into 50 mM Tris–HCl buffer (pH = 7.5). *Ms*CO1 and the EV control were then snap-frozen at −70 °C. Protein concentration was estimated using the Bradford assay (no protein was detected for EV control).

### In vitro reactions

*Ms*CO1 variants, *Ms*DCR isoforms/variants and/or the EV control for *Ms*CO1 were thawed at −70 °C and centrifuged for 5 min at 10,000*g* to pellet any aggregated enzyme. The supernatants were then removed and used for the following assays: 25 μl total volume containing 100 μM substrate (20*S*-corynantheidine (**3a**), ajmalicine (**10b**) or DHC (**13a**)), 1 mM NADPH, 0 or 1 μM *Ms*CO1/EV control and/or 1 μM *Ms*DCR variant and 50 mM HEPES (pH = 7.5). Reactions were allowed to proceed at 25 °C for 16 h before quenching via 20:1 addition of MeOH. Solutions were then filtered and analyzed via LC–MS.

### Molecular docking

The structures of *Ms*CO1, *Ms*DCR1 and *Ms*DCR2 were predicted using AlphaFold^33^. Small molecule structures were optimized using the semi-empirical method PM6; the resulting structures were used for conformer variation with the GMMX processor of the Gaussian program package. Resulting structures were density functional theory-optimized with Gaussian ver. 16 (B3LYP/6-31G(d), gas phase). AutoDock Vina^45,46^ was used to predict substrate docking conformations. The docking outputs are visualized using PyMol.

### Reporting summary

Further information on research design is available in the Nature Portfolio Reporting Summary linked to this article.

## Online content

Any methods, additional references, Nature Portfolio reporting summaries, source data, extended data, supplementary information, acknowledgements, peer review information; details of author contributions and competing interests; and statements of data and code availability are available at 10.1038/s41589-025-01970-9.

## Supplementary information

Supplementary Information Supplementary Figs. 1–75, Tables 1–5 and methods.

Reporting Summary

## Data Availability

All studied gene/enzyme sequence data are available in Supplementary Information. Kratom Sequence Read Archive data are deposited under GenBank BioProject PRJNA1244102. *U. guianensis* Sequence Read Archive data are deposited under GenBank BioProject PRJNA1167310. *Ms*CO1 (PV711318), *Ms*CO2 (PV711319), *Ms*DCR1 (PV711320), *Ms*DCR2 (PV711321) and *Ms*10H (PV711322) have been deposited in the National Center for Biotechnology Information. Data are available from the corresponding author upon request.

## Code availability

Custom code used for alignment of transcriptome sequences is available on GitHub (https://github.com/Allwin-McDonald/Cross-Transcriptome-Homology-Analysis.git).
