# Supplementary material for: Enzymatic epimerization of monoterpene indole alkaloids in kratom
Source: Nat Chem Biol. 2025 Jul 16;22(2):229–38. doi: 10.1038/s41589-025-01970-9 (PMC12441186; doi:10.1038/s41589-025-01970-9)
Supplement: Supplementary file 1 — Supplementary Figs. 1–75, Tables 1–5 and methods. [file 41589_2025_1970_MOESM1_ESM.pdf]

nature chemical biology

Supplementary information https://doi.org/10.1038/s41589-025-01970-9

Enzymatic epimerization of monoterpene indole alkaloids in kratom

In the format provided by the authors and unedited

Table of Contents:

Supplementary Figures 2 – 41

Supplementary Tables 42 – 46

Supplementary Methods 47 – 50

Coding Sequences 51 – 60

Synthetic Methods 61 – 69

Structural Data 70 – 105

Supplementary References 106 – 107

Supplementary Figures

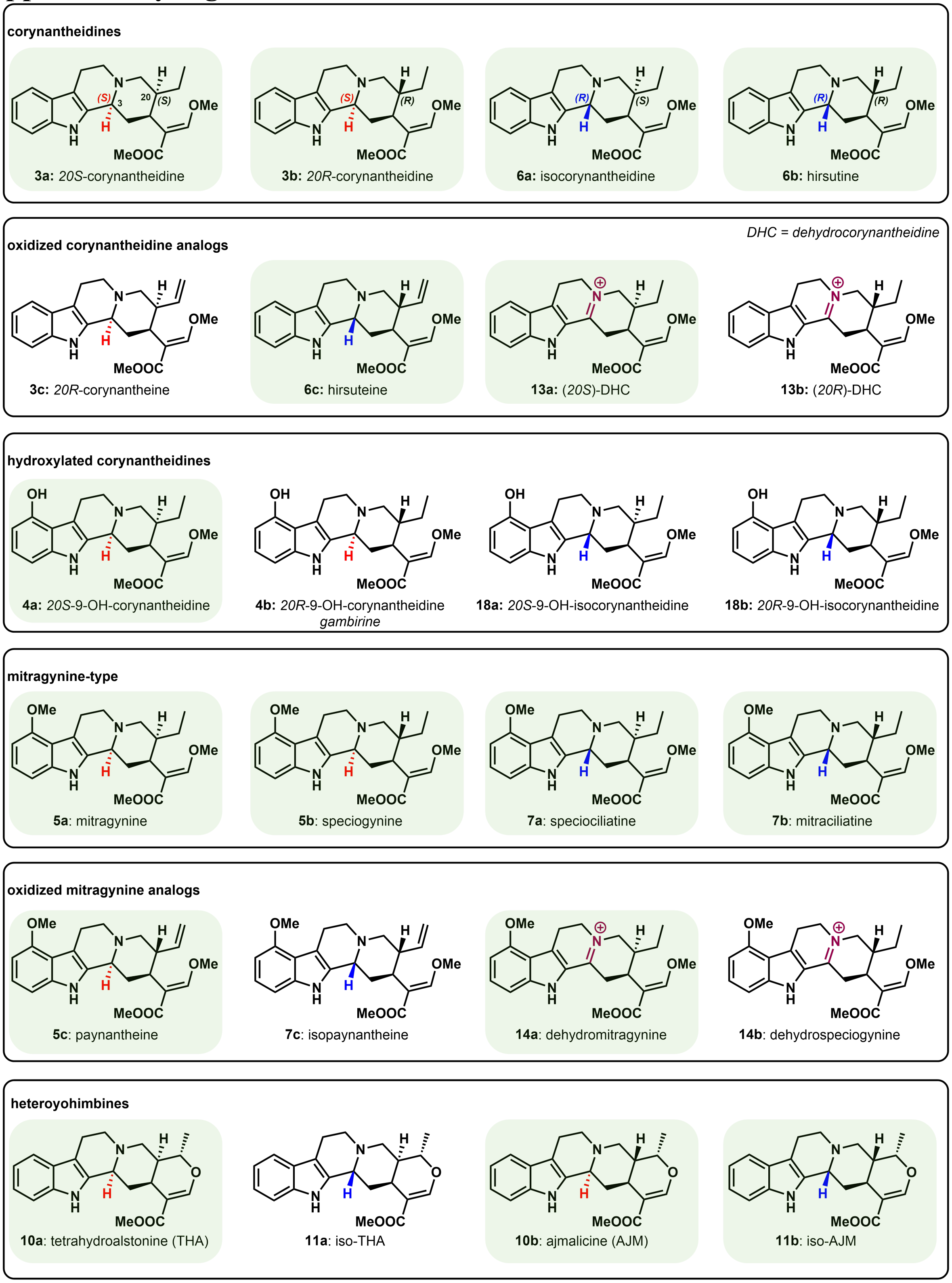

Supplementary Figure 1: Structures and names of select Kratom alkaloids. Compounds with verified standards are highlighted in green.

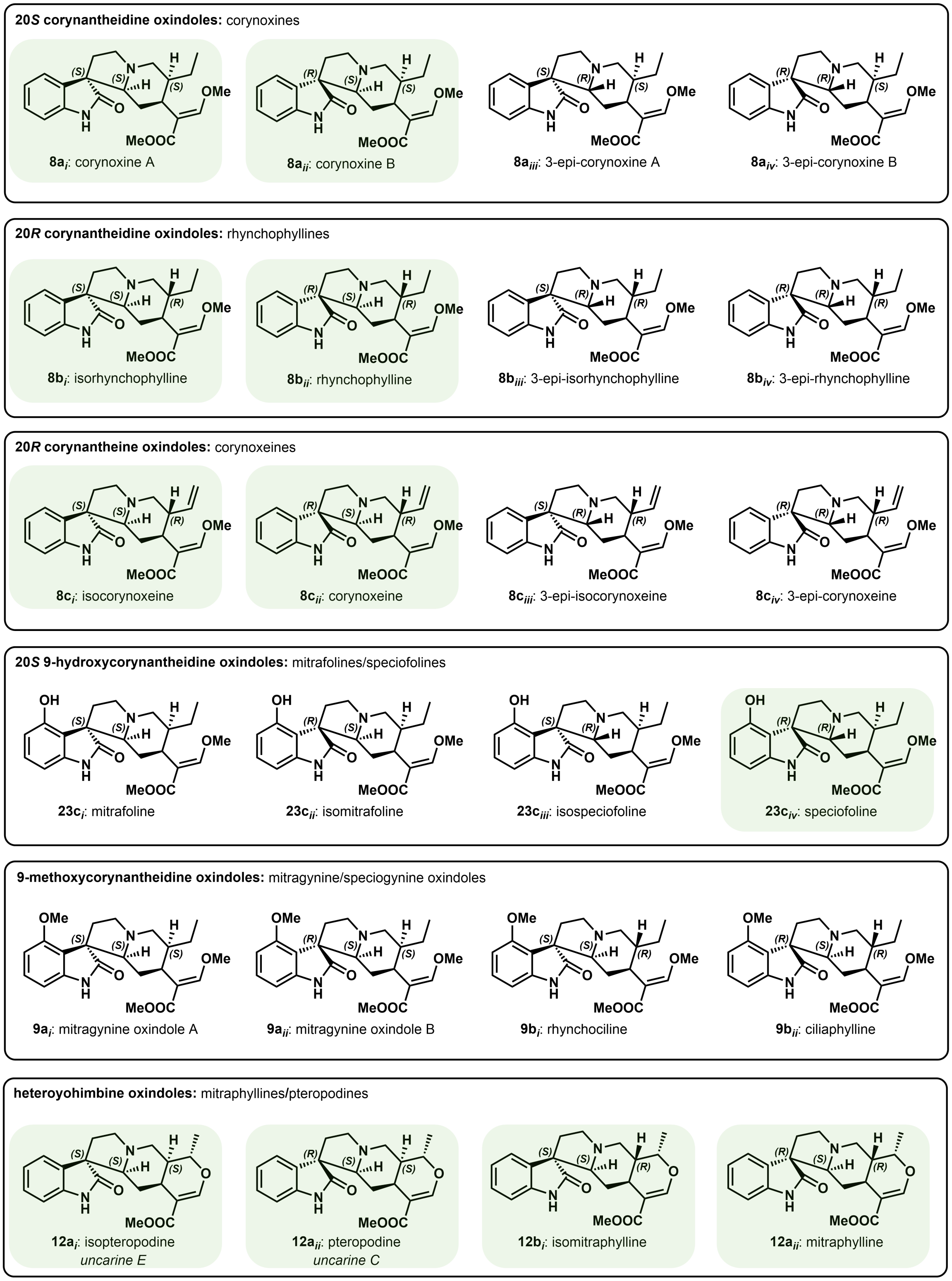

Supplementary Figure 2: Structures and names of select Kratom spirooxindole alkaloids. Compounds with verified standards are highlighted in green. **Note:** different spirocylized isomers (*i.e.* **8a**) are differentiated via subscript lettering (i.e. $8a_i$ corresponds to the (3*S*, 7*S*) isomer). We adopted this numbering scheme to avoid confusion since these isomers interconvert under our assay conditions.

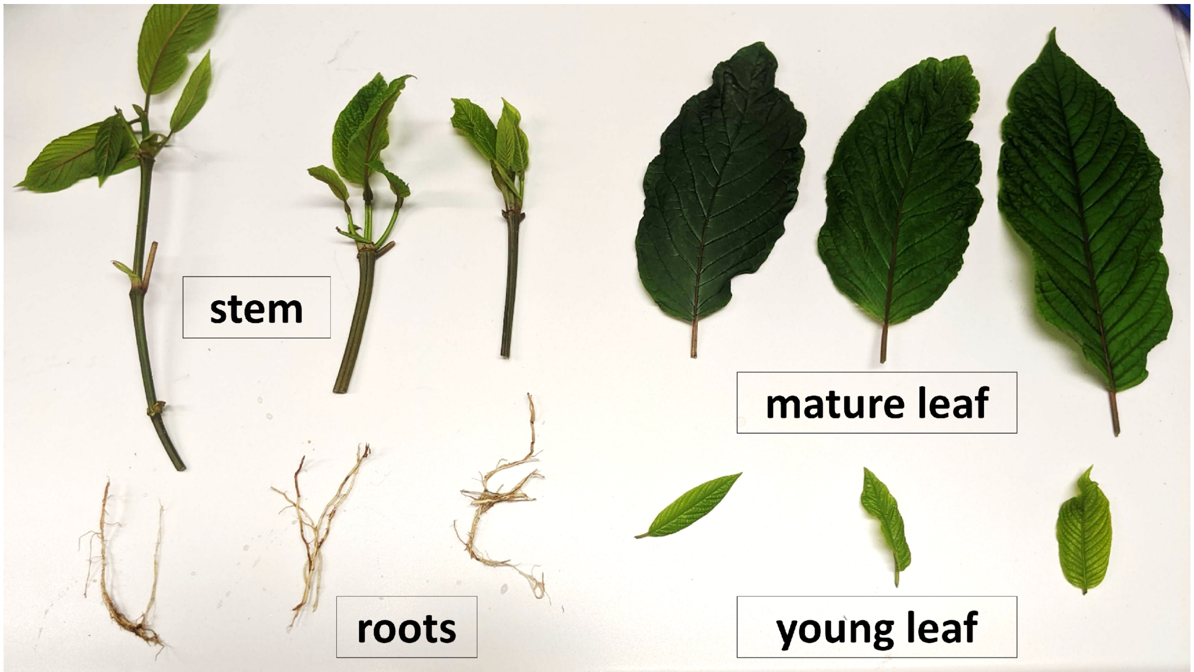

Supplementary Figure 3: Kratom tissues used for alkaloid analysis and feeding studies. Note that for stems, cut stem disks were acquired from the stem internode preceding young leaf petioles. Roots were cut into small 2 mm fragments, while 5 mm leaf disks were cut out from leaf tissue.

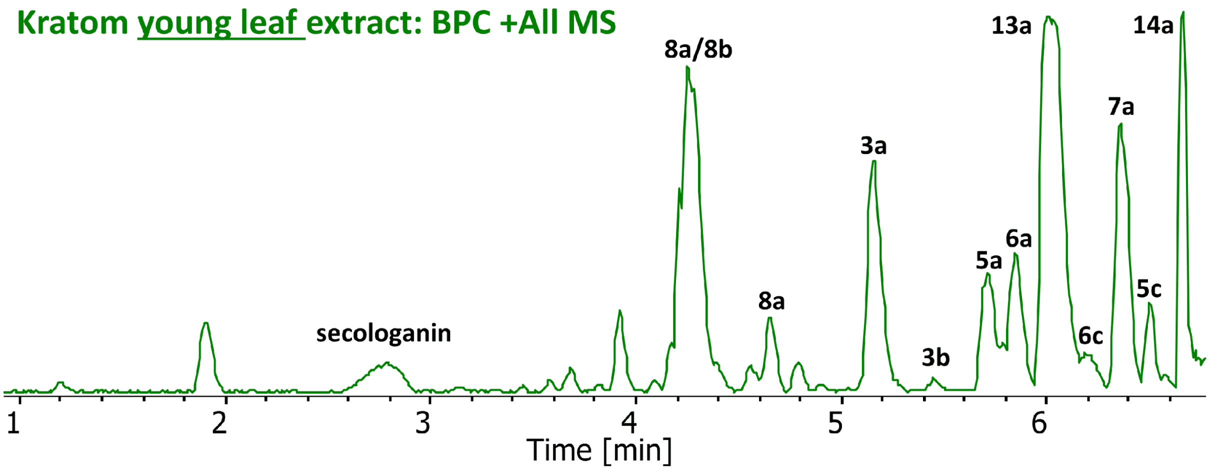

Supplementary Figure 4: Kratom young leaf base peak chromatogram (BPC) with identified alkaloids. Designation of **8a/8b** represents mixtures of corynoxine (**8a**)/rhynchophylline (**8b**) isomers.

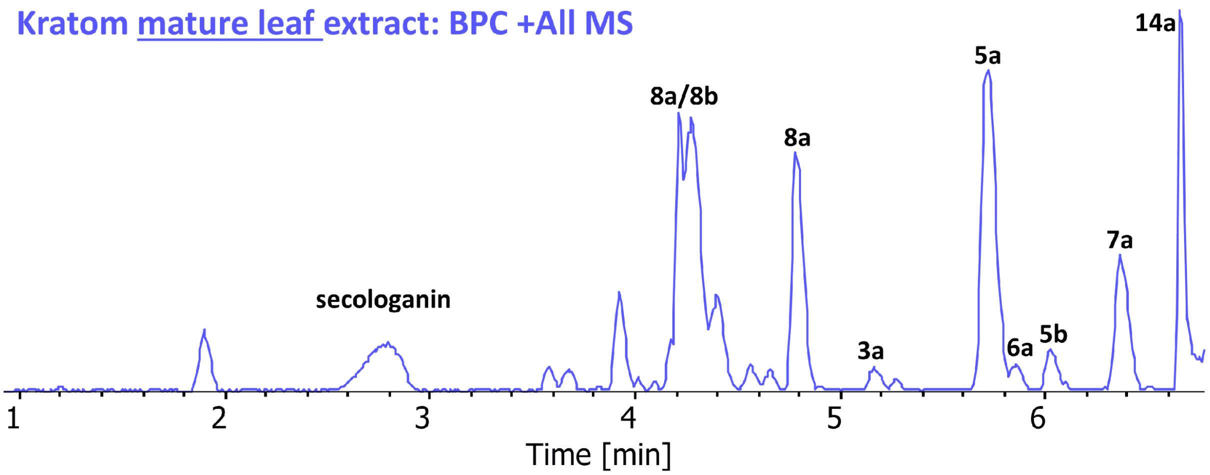

Supplementary Figure 5: Kratom stem base peak chromatogram (BPC) with identified alkaloids. Designation of **8a/8b** represents mixtures of corynoxine (**8a**)/rhynchophylline (**8b**) isomers.

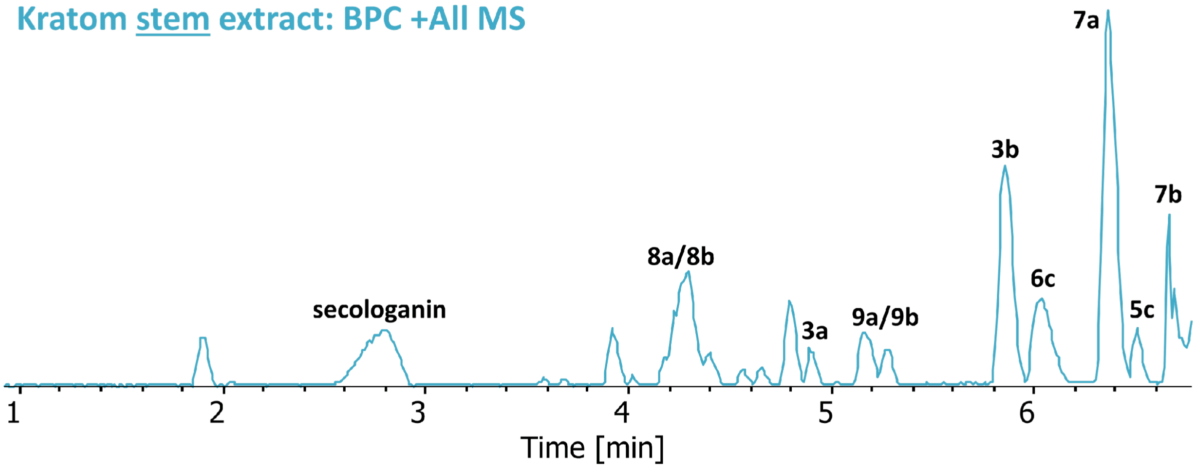

Supplementary Figure 6: Kratom ML base peak chromatogram (BPC) with identified alkaloids. Designation of **8a/8b** represents mixtures of corynoxine (**8a**)/rhynchophylline (**8b**) isomers, and designation of **9a/9b** represents unknown peaks corresponding to mitragynine oxindole (**9a**)/speciogynine oxindole (**9b**) isomers.

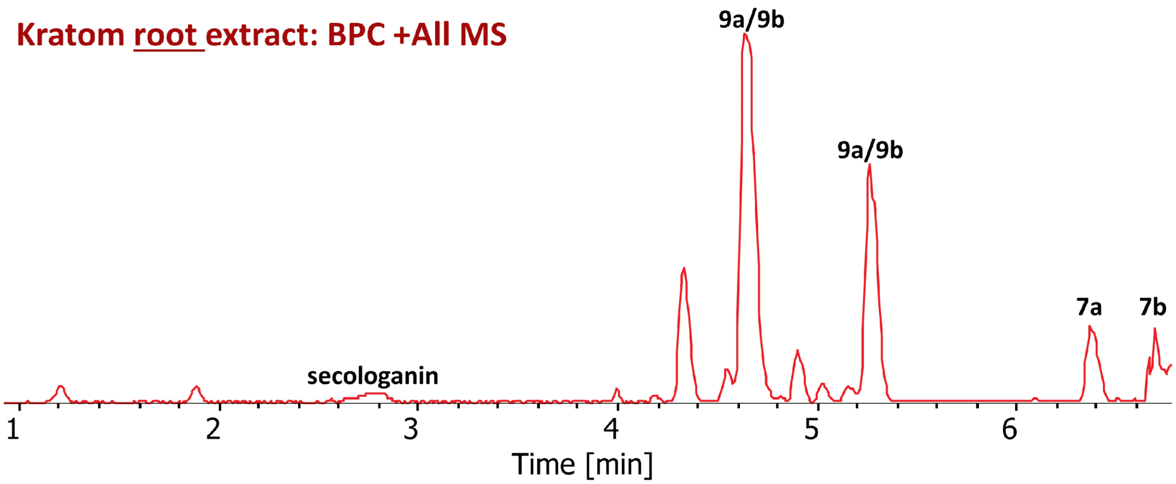

Supplementary Figure 7: Kratom root base peak chromatogram (BPC) with identified alkaloids. Designation of **9a/9b** represents unknown peaks corresponding to mitragynine oxindole (**9a**)/speciogynine oxindole (**9b**) isomers.

a) Reduction of DHC (**13a**) via $NaBH_4$

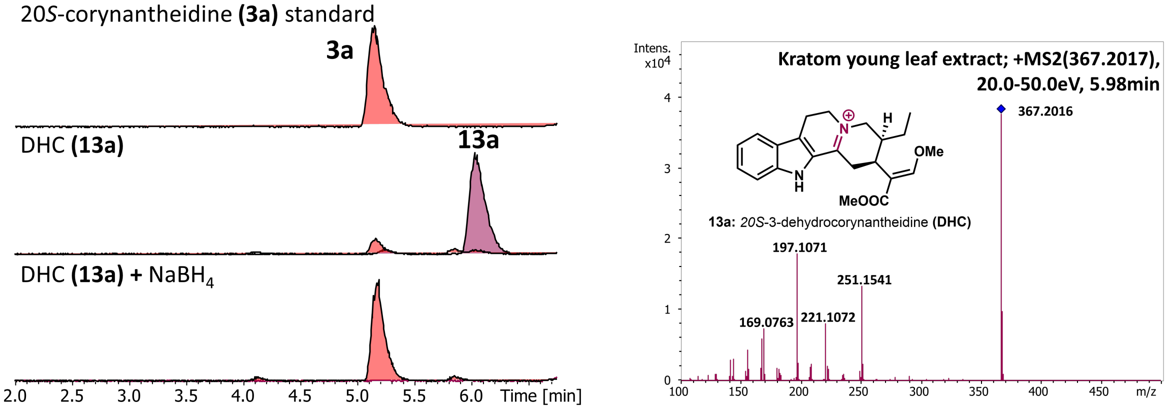

b) Reduction of DHM (**14a**) via $NaBH_4$

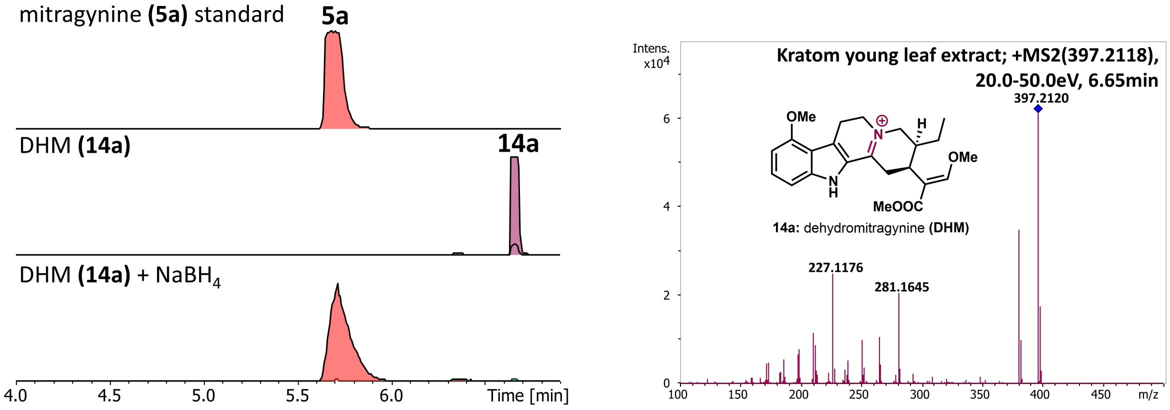

c) Stereoselective $NaBH_4$ reduction

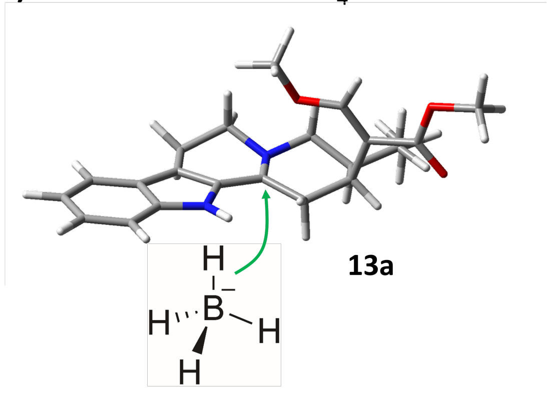

d) Observed fluorescence of DHC

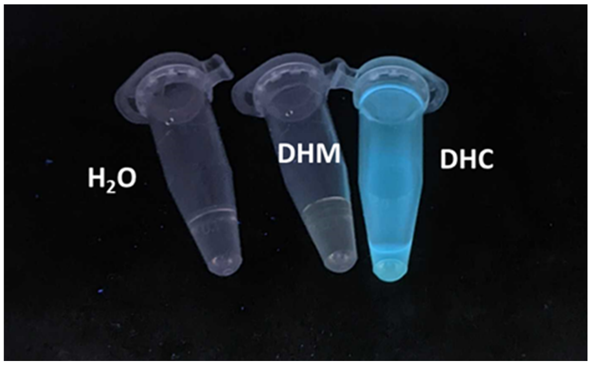

Supplementary Figure 8: a) Reduction of 100 μM isolated DHC (**13a**) with 10 mM $NaBH_4$ at r.t. for 1 h. Full conversion to (20*S*)-corynantheidine is observed with no observable formation of other products. MS/MS analysis of isolated DHC. NMR structural characterization of DHC is detailed in Supplementary Figures 65-68 and Supplementary Table 4. **b)** Reduction of 500 μM DHM with 10 mM $NaBH_4$ at r.t. for 1 h. Full conversion to mitragynine is observed with no observable formation of other products. MS/MS analysis of isolated DHM. NMR structural characterization of DHM is detailed in Supplementary Figure 62. **c)** Depiction of $NaBH_4$ reduction of **13a** showing the facial selectivity of hydride addition. **d)** Observed fluorescence of 100 μM DHC under UV light.

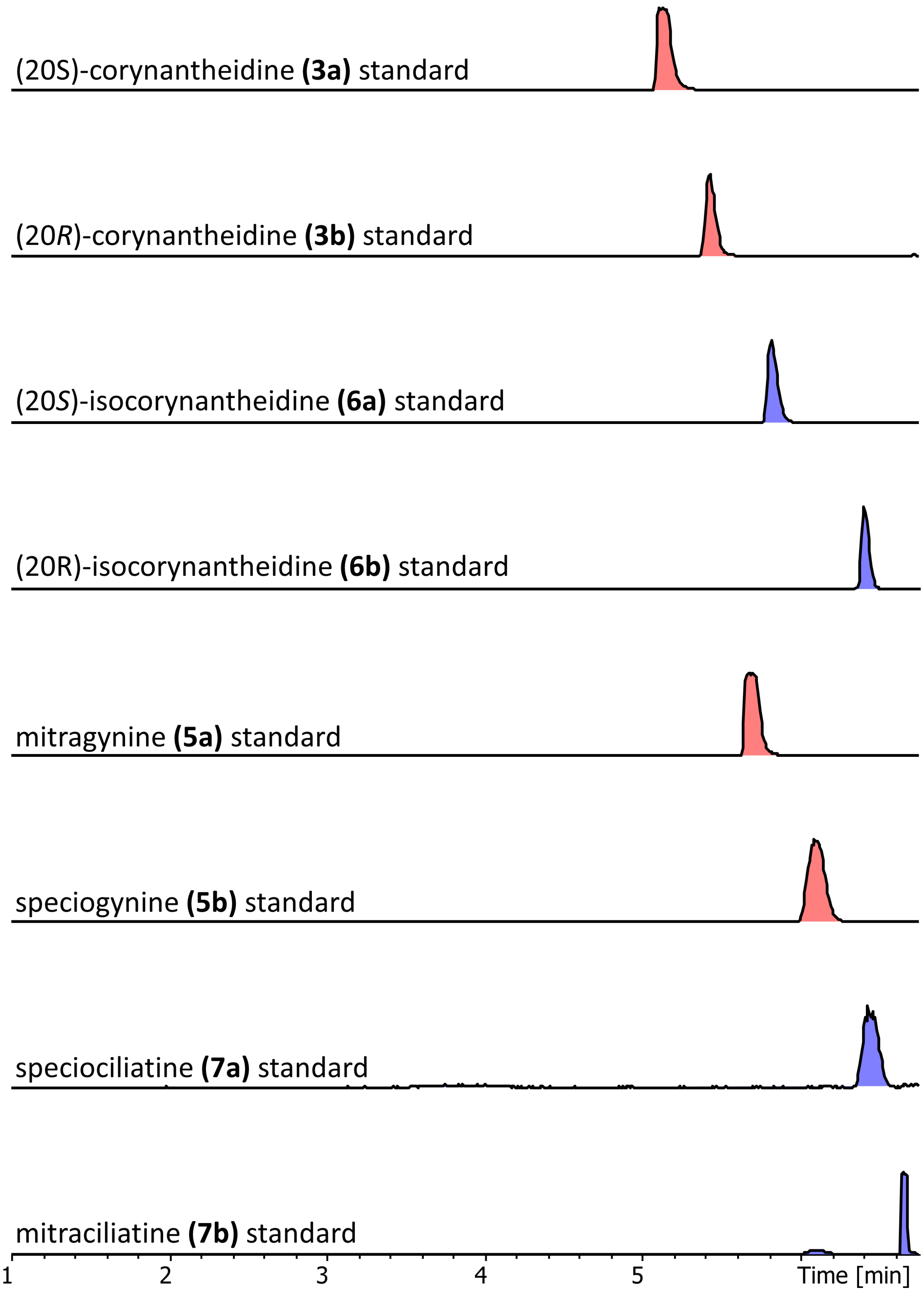

Supplementary Figure 9: Retention times and elution order of corynantheidine and mitragynine stereoisomers with y-axis denoting ionization intensity. We observed a consistent elution order that corresponded to relative stereochemistry at C3 and C20 for both methoxylated and non-methoxylated corynantheidines. In this way, relative retention times assisted in identification of corynantheidine/mitragynine stereoisomers. NMR structural characterization for each isomer is detailed in Supplementary Figures 54-61.

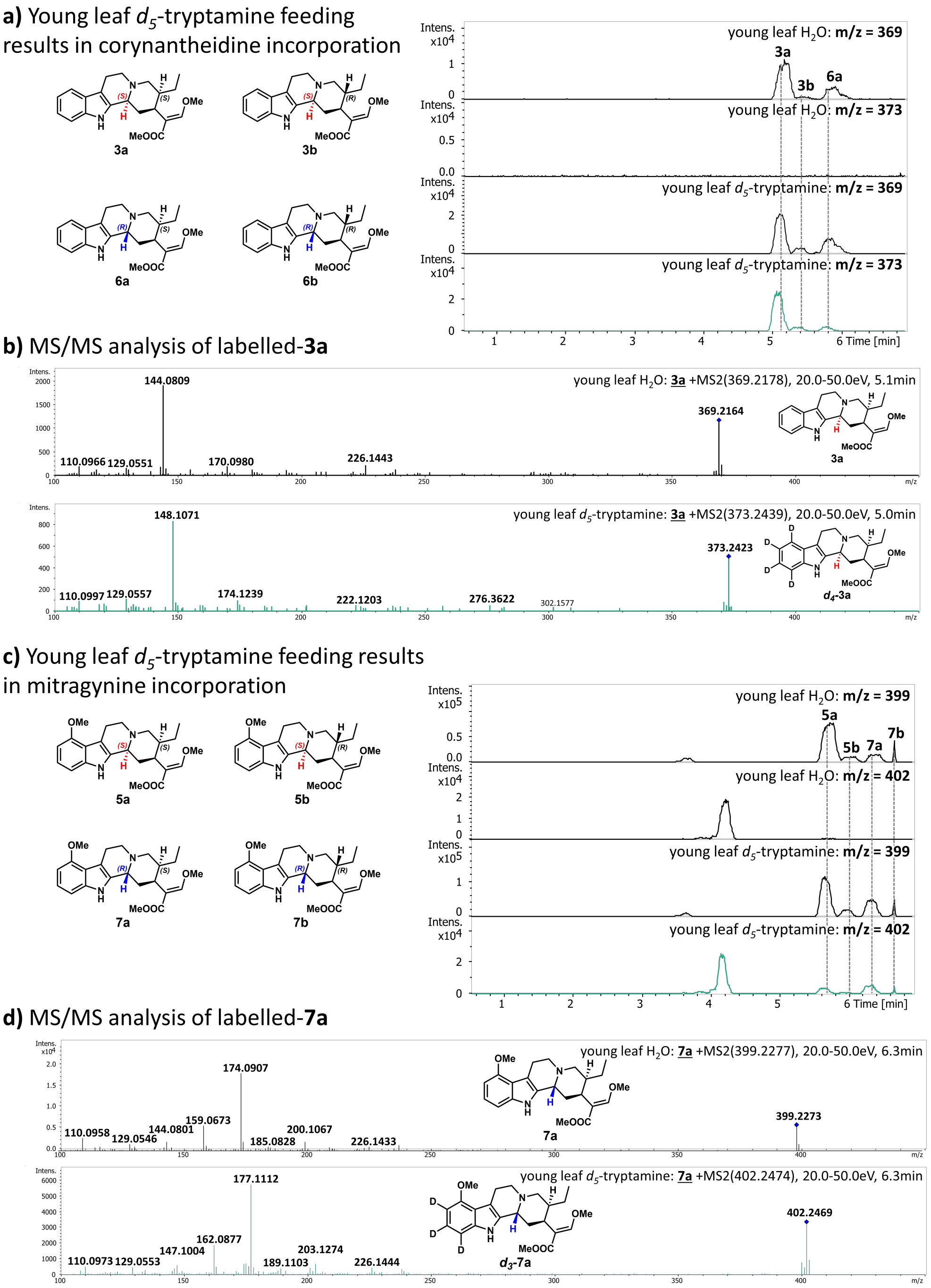

Supplementary Figure 10: Feeding of d_5-tryptamine to young leaf tissue. Shown data are representative spectra from biological replicates (n = 3). All products were determined via comparison with authentic standards as detailed in Supplementary Figure 9. **a)** LC-MS traces of control (H_2O) *m/z*+0 and *m/z*+4 and of fed (*d_5*-tryptamine) *m/z*+0 and *m/z*+4. **b)** MS2 analysis showing a *m/z*+4 shift relative to (20*S*)-corynantheidine (**3a**). **c)** LC-MS traces of control (H_2O) *m/z*+0 and *m/z*+3 and of fed (*d_5*-tryptamine) *m/z*+0 and *m/z*+3. **d)** MS2 analysis showing a *m/z*+3 shift relative to speciociliatine (**7a**).

Kratom tissue feeding results for d_4-strictosidine/d_4-vincoside

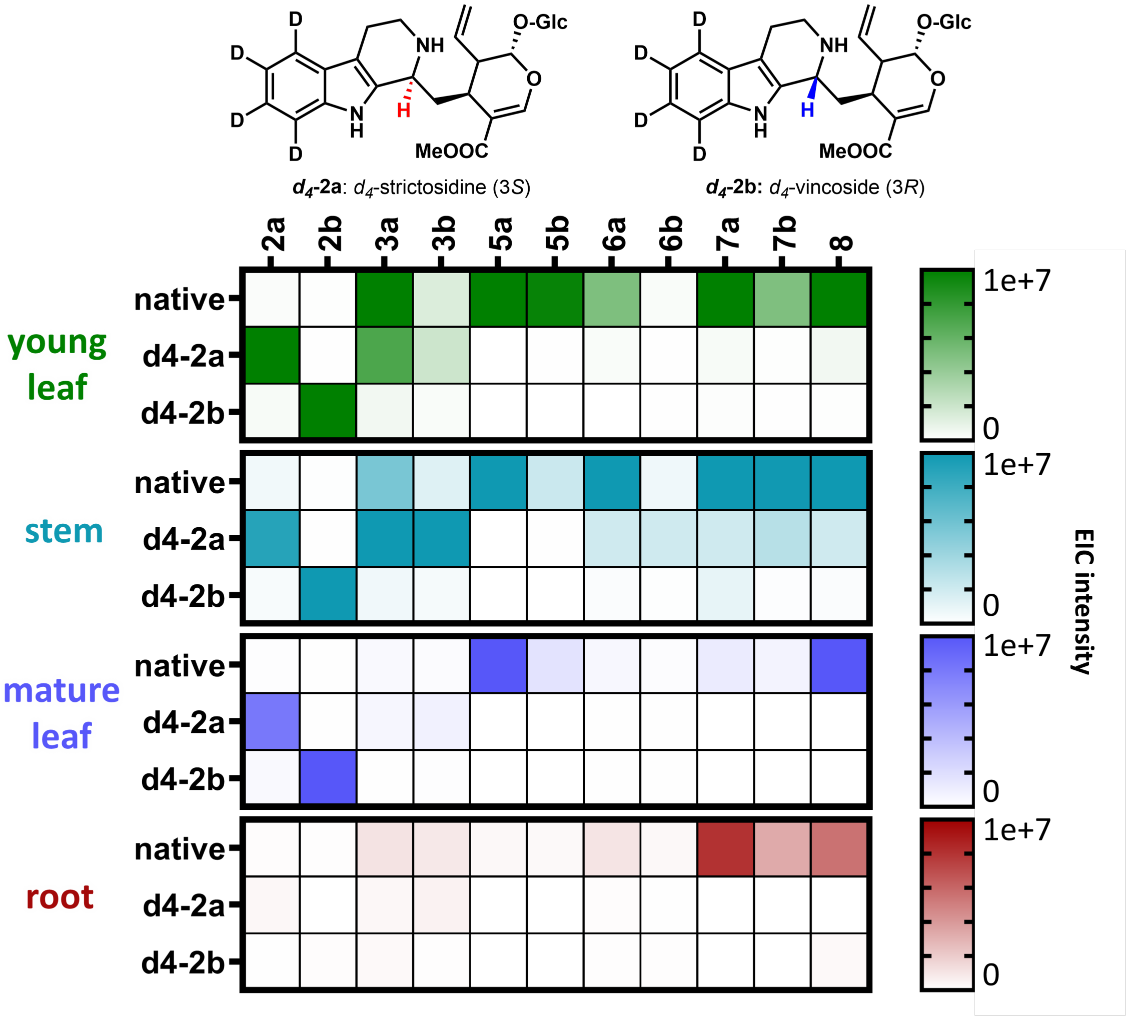

Supplementary Figure 11: Native metabolite distribution of Kratom tissues and incorporation from labeled d_4-strictosidine (***d_4*-2a**) and d_4-vincoside (***d_4*-2b**). Values are derived from extracted ion chromatograms (EIC) from expected *m/z* values and are shown as the average of biological triplicates. Note that values for fed tissues are scaled 5x in the above heat map for better visualization (raw values in Supplementary Tables 1-2).

a) Screening results from BBE candidates

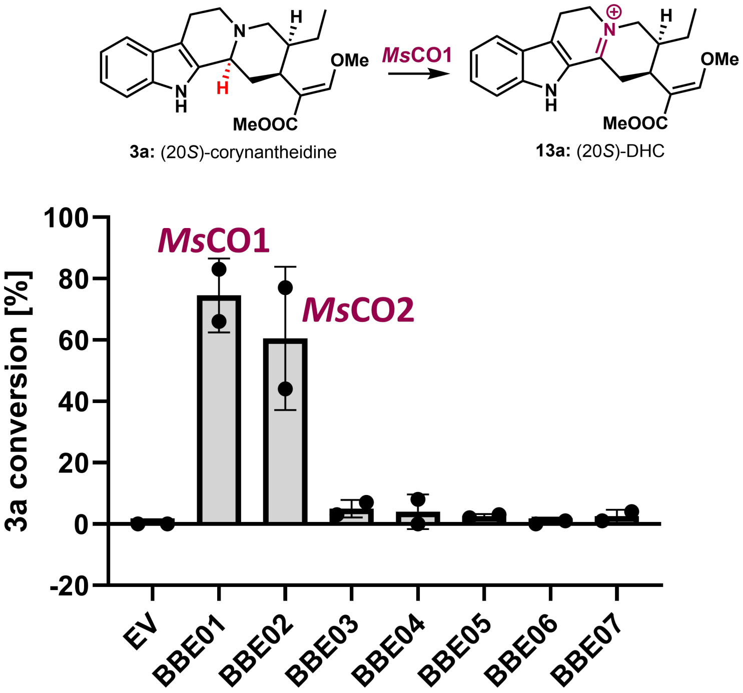

b) Identity matrix of BBEs and *Cr*PAS

	*Ms*CO1	*Ms*CO2	BBE03	BBE04	BBE05	BBE06	BBE07	CrPAS
*Ms*CO1		**93.7**	**81.6**	**46.5**	**46.4**	**42.2**	**42.4**	**53.4**
*Ms*CO2	**93.7**		**81.3**	**47.3**	**46.9**	**42.9**	**43.2**	**53.5**
BBE03	**81.6**	**81.3**		**47.7**	**45.9**	**42.6**	**42.0**	**55.7**
BBE04	**46.5**	**47.3**	**47.7**		**58.6**	**44.9**	**44.1**	**42.3**
BBE05	**46.4**	**46.9**	**45.9**	**58.6**		**42.0**	**42.5**	**42.3**
BBE06	**42.2**	**42.9**	**42.6**	**44.9**	**42.0**		**73.9**	**39.9**
BBE07	**42.4**	**43.2**	**42.0**	**44.1**	**42.5**	**73.9**		**42.1**
CrPAS	**53.4**	**53.5**	**55.7**	**42.3**	**42.3**	**39.9**	**42.1**	

Supplementary Figure 12: Newly discovered Kratom epimerase enzymes. **a)** Activity of berberine bridge-like enzyme (BBE) candidates on corynantheidine (**3a**). Assay conditions: 16 h 100 µM **3a** incubation into *N. benthamiana* leaves infiltrated with the indicated gene. Background activity from EV control is subtracted from final conversion values. Shown data are representative spectra from biological replicates (n = 2) with standard deviations depicted. **b)** Sequence alignment matrix of BBE candidates with each other and *Catharanthus roseus* precondylocarpine acetate synthase (*Cr*PAS).

a) Activity of select BBE candidates on **3a**

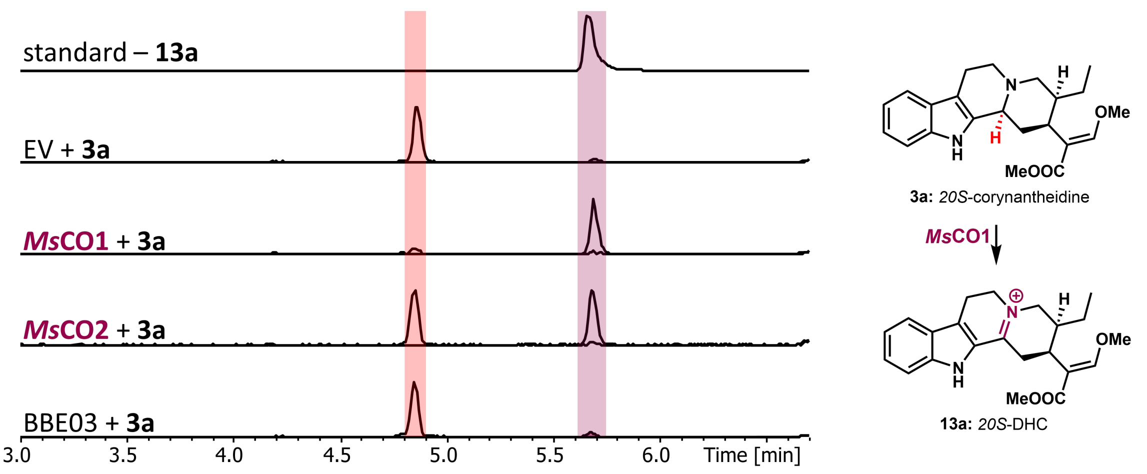

b) Lack of activity of select BBE candidates on **5a**

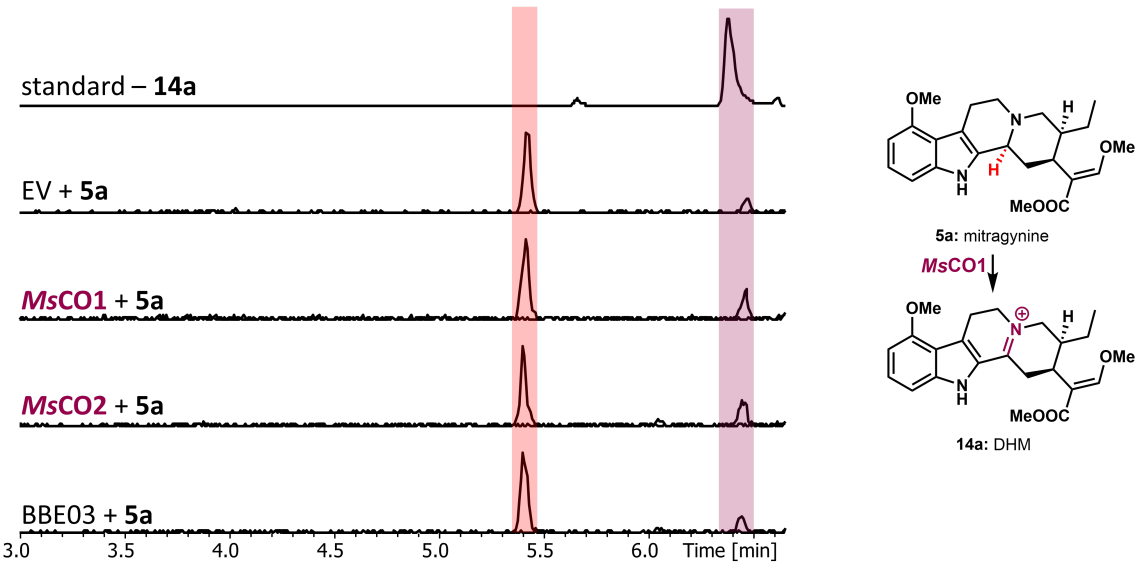

Supplementary Figure 13: Activity differences of *Ms*CO1 and *Ms*CO2 with **a) 3a** or **b) 5a**. BBE03 is included as an example of a candidate with no activity on **3a**. Assays were performed as described in Supplementary Figure 12. Shown data are representative spectra from biological replicates (n = 2).

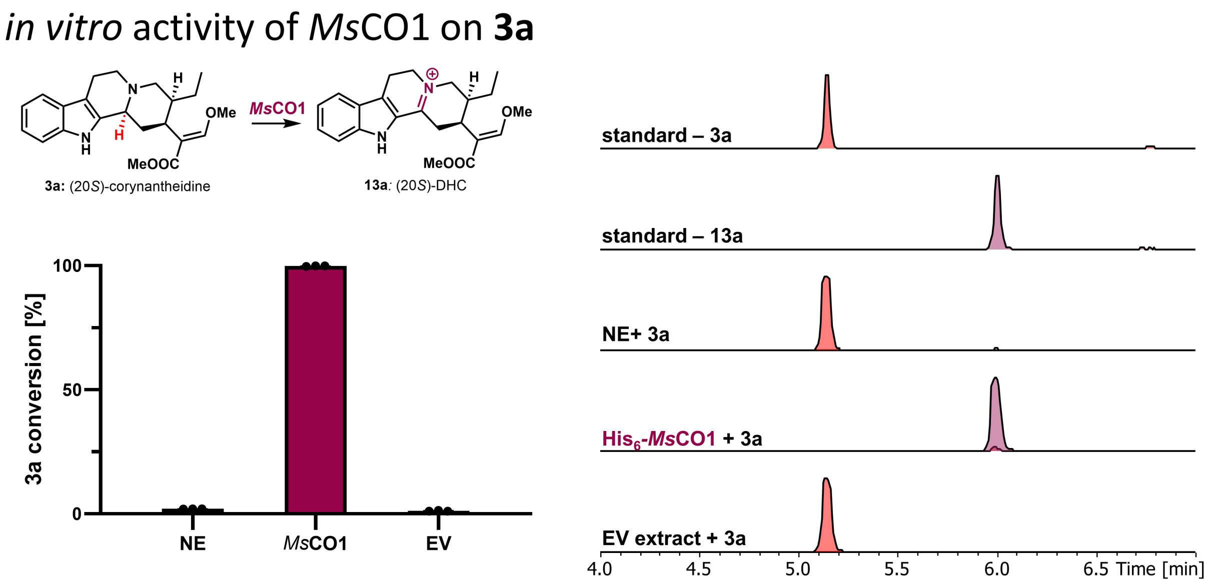

Supplementary Figure 14: Reaction of *Ms*CO1 converting **3a** to **13a**. EV = empty vector control; NE = no enzyme control. Reaction conditions: 100 µM **3a**, 0.5 µM *Ms*CO1/EV control, 50 mM Tris-HCl pH = 7.5, 25 ˚C for 16 h. Shown data are representative spectra from technical replicates (n = 3) with standard deviations depicted.

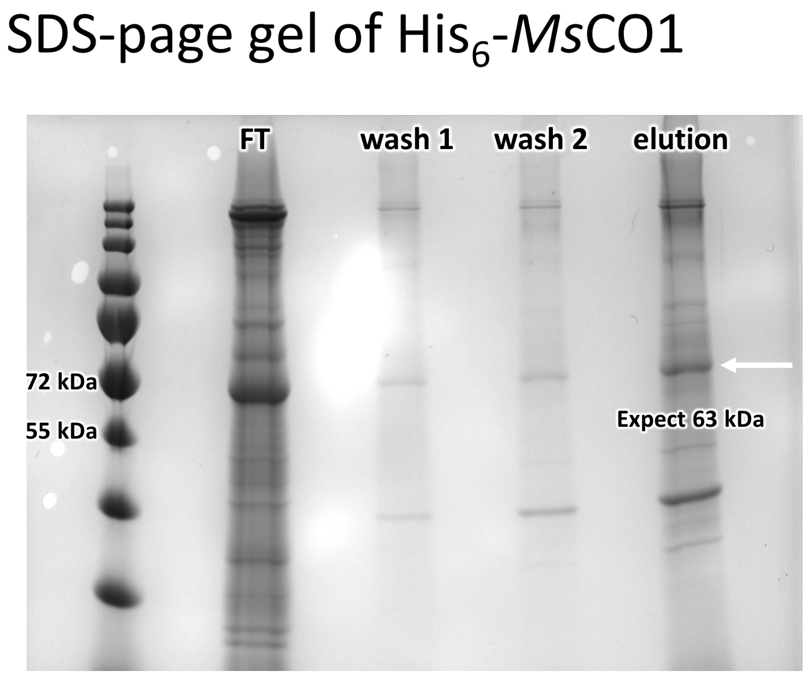

Supplementary Figure 15: SDS-page gel of expressed His$_6$-*Ms*CO1 from *N. benthamiana.* FT = column flow-through.

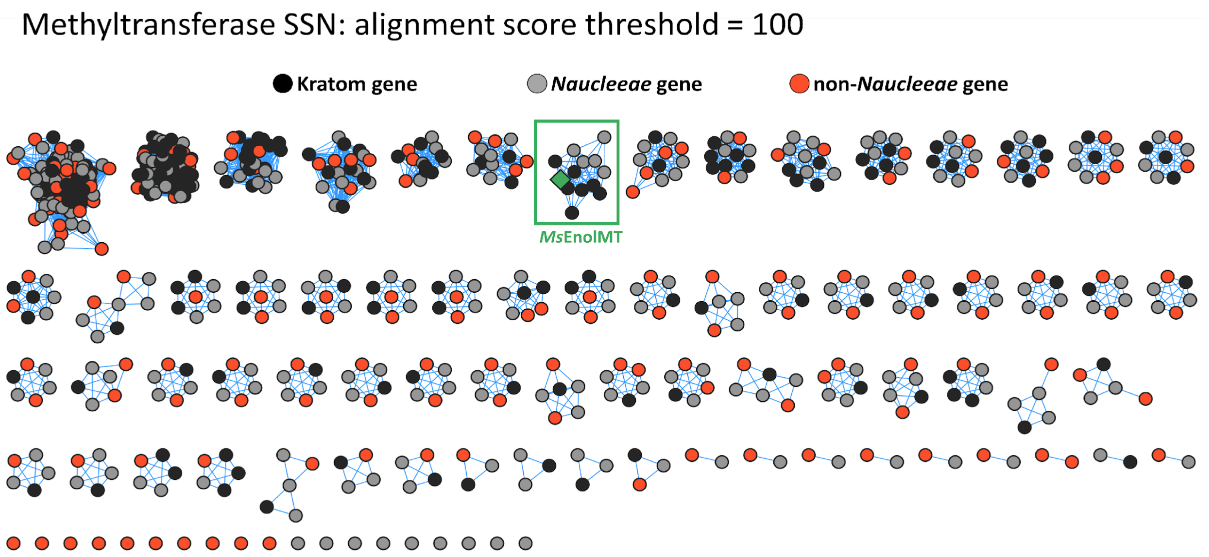

Supplementary Figure 16: Sequence similarity network (SSN) to identify *Naucleeae*-specific methyltransferases with alignment score threshold of 100. Kratom genes are shown in black, *Naucleeae* genes in gray, and non-*Naucleeae* genes in orange. The *Naucleeae*-specific cluster containing the previously identified *Ms*EnolMT is highlighted in green.

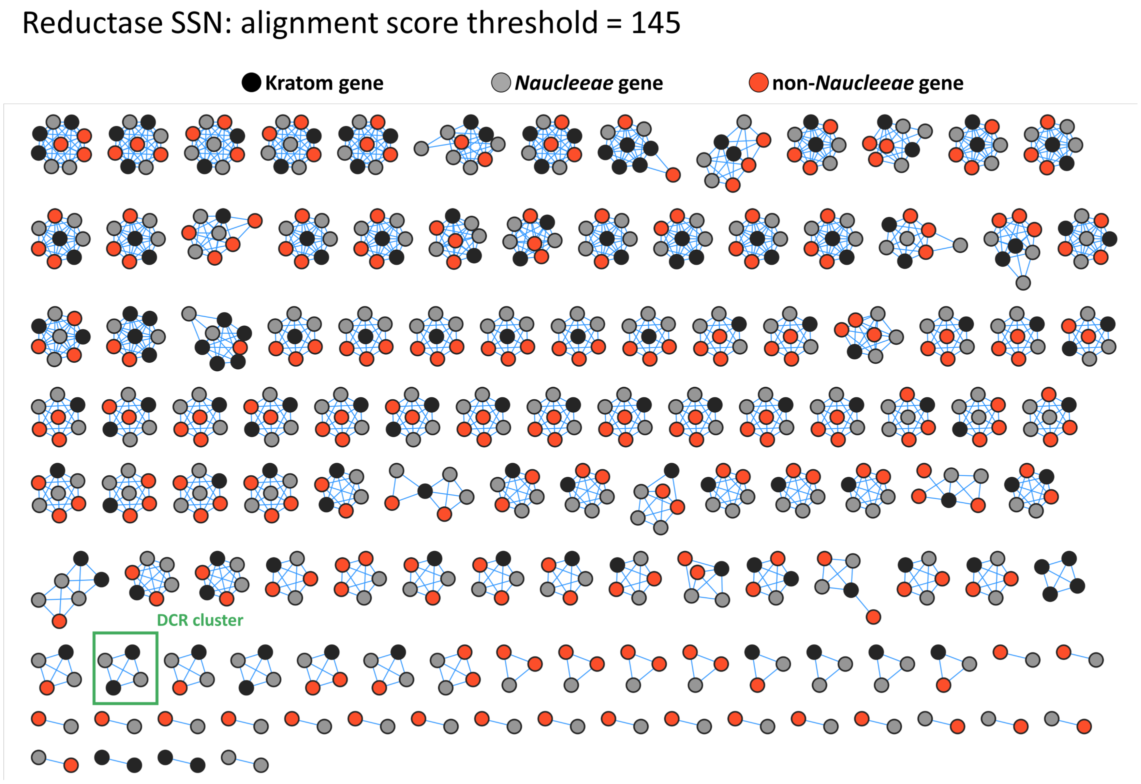

Supplementary Figure 17: Sequence similarity network (SSN) to identify *Naucleeae*-specific reductases/dehydrogenases with alignment score threshold of 145. Kratom genes are shown in black, *Naucleeae* genes in gray, and non-*Naucleeae* genes in orange. The *Naucleeae*-specific cluster containing *Ms*DCR isoforms is highlighted with a box (green).

a) *Investigated reductase candidates from SSN network analysis*

Gene ID	FPKM_ML	FPKM_YL	FPKM_S	P_{CO1}	Annotation
Red1	15	380	25	**1.00**	Glucose 1-dehydrogenase
Red2	39	156	19	**0.97**	Tropinone reductase homolog
MsDCR2	313	500	34	**0.74**	Isoflavone reductase-like protein
Red4	5	9	6	**0.97**	Putative oxidoreductase GLYR1
Red5	25	21	10	**0.17**	5'-adenylylsulfate reductase-like 5
MsDCR1	141	348	181	**1.00**	Isoflavone reductase-like protein
Red7	11	15	4	**0.72**	Probable mannitol dehydrogenase
Red8	129	72	249	**-0.67**	2-methylene-furan-3-one reductase

b) *Reductase sequence identity matrix*

	***Ms*DCR1**	***Ms*DCR2**	*Ca*IFR	*Ms*DCS1	*Cr*THAS1	*Cr*DPAS
***Ms*DCR1**		**93.6**	**73.3**	**13.4**	**12.2**	**14.4**
***Ms*DCR2**	**93.6**		**73.2**	**12.9**	**11.6**	**14.4**
*Ca*IFR	**73.3**	**73.2**		**16.0**	**14.6**	**15.3**
*Ms*DCS1	**13.4**	**12.9**	**16.0**		**63.4**	**56.5**
*Cr*THAS1	**12.2**	**11.6**	**14.6**	**63.4**		**53.3**
*Cr*DPAS	**14.4**	**14.4**	**15.3**	**56.5**	**53.3**	

c) *Protein sequence alignment of MsDCR1 and MsDCR2*

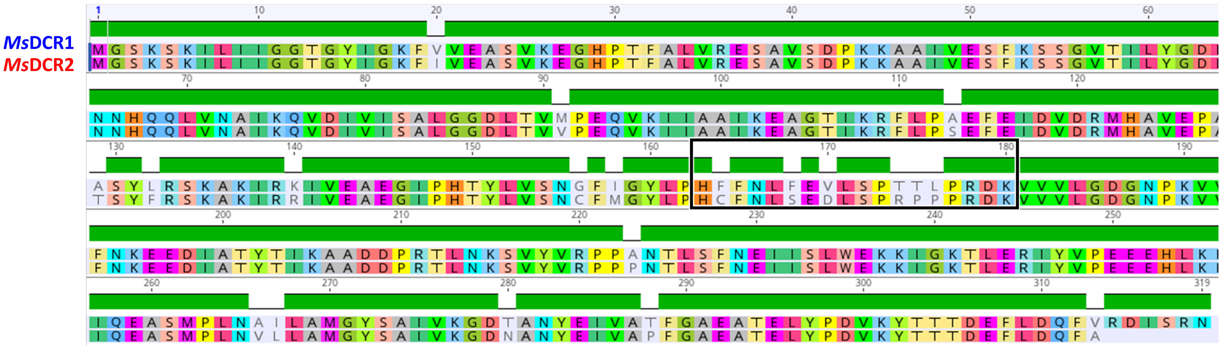

Supplementary Figure 18: a) Reductase candidates identified via SSN analysis (Supplementary Figure 17) with Fragments Per Kilobase Million (FPKM) counts and Pearson correlations with *Ms*CO1. Pearson values were calculated using Microsoft Excel. **b)** Identity matrix of *Ms*DCR isoforms, *Coffea arabica* isoflavone reductase (*Ca*IFR), and reported MIA iminium dehydrogenases: *Ms*DCS1, *Cr*THAS1, and *Cr*DPAS. **c)** Sequence alignment of *Ms*DCR1 and *Ms*DCR2 with the loop region adjacent to the active site highlighted with a black box.

*In vitro activity verification of MsDCRs – **13a***

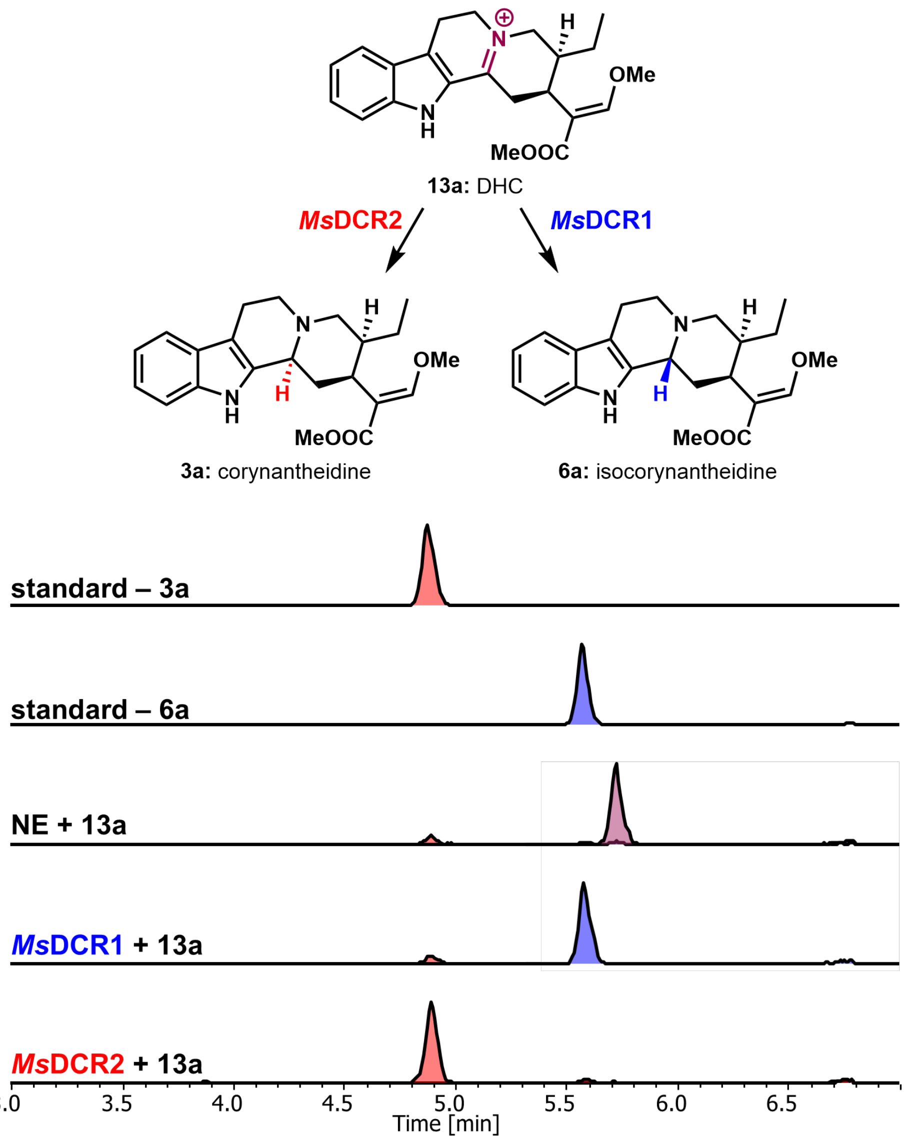

Supplementary Figure 19: Reaction of *Ms*DCR1 converting **13a** to **6a**. NE = no enzyme control. Reaction conditions: 100 µM **13a**, 100 µM NADPH, 1 µM *Ms*DCR1, 50 mM HEPES pH = 7.5, 25 ˚C for 16 h. Shown data are representative spectra from biological replicates (n = 3).

In vitro activity verification of MsDCRs – ***14a***

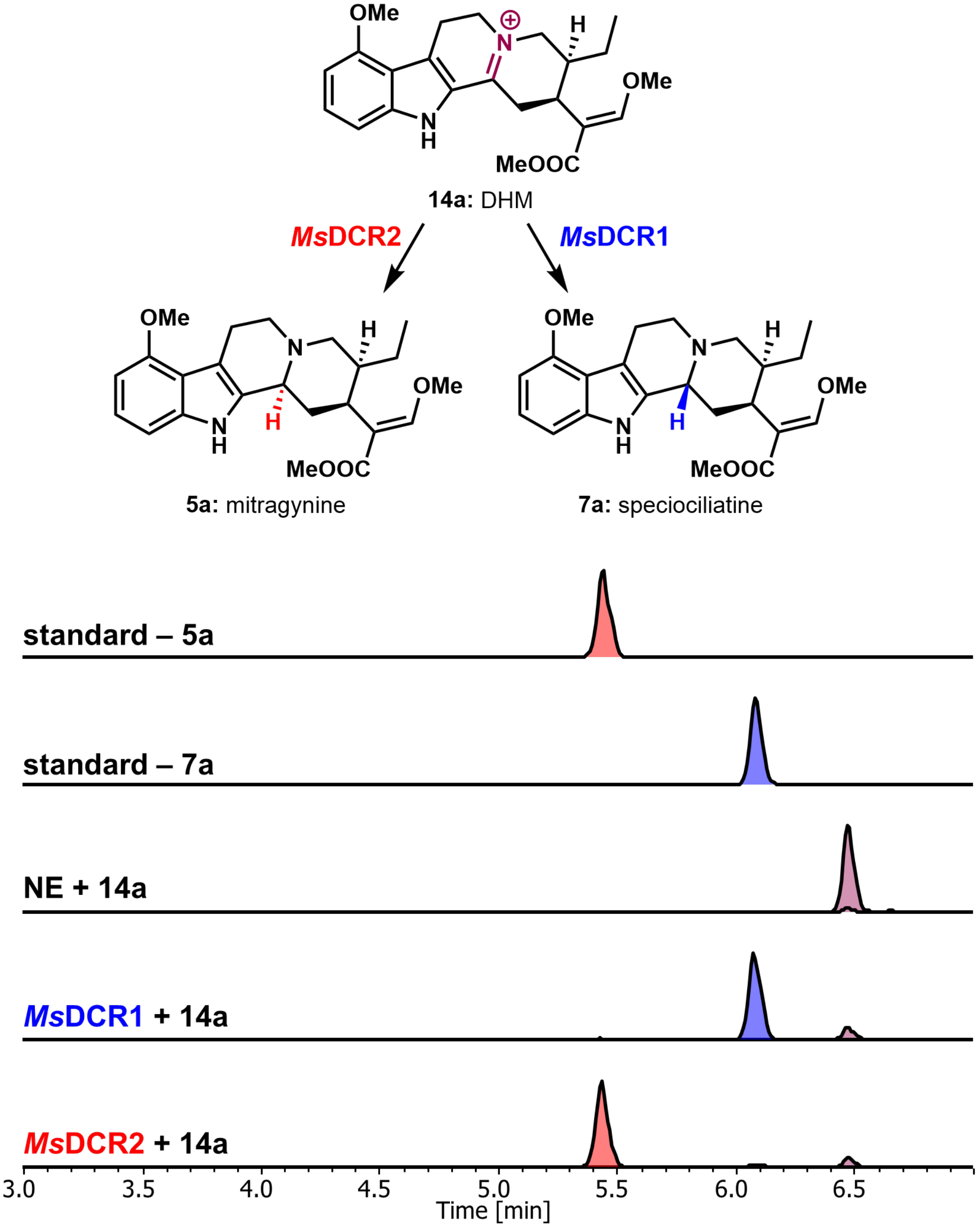

Supplementary Figure 20: Reaction of *Ms*DCR1 converting **13a** to **6a**. NE = no enzyme control. Reaction conditions: 100 µM **14a**, 100 µM NADPH, 1 µM *Ms*DCR1, 50 mM HEPES pH = 7.5, 25 ˚C for 16 h. Shown data are representative spectra from biological replicates (n = 3).

SDS-page gel of *Ms*DCRs

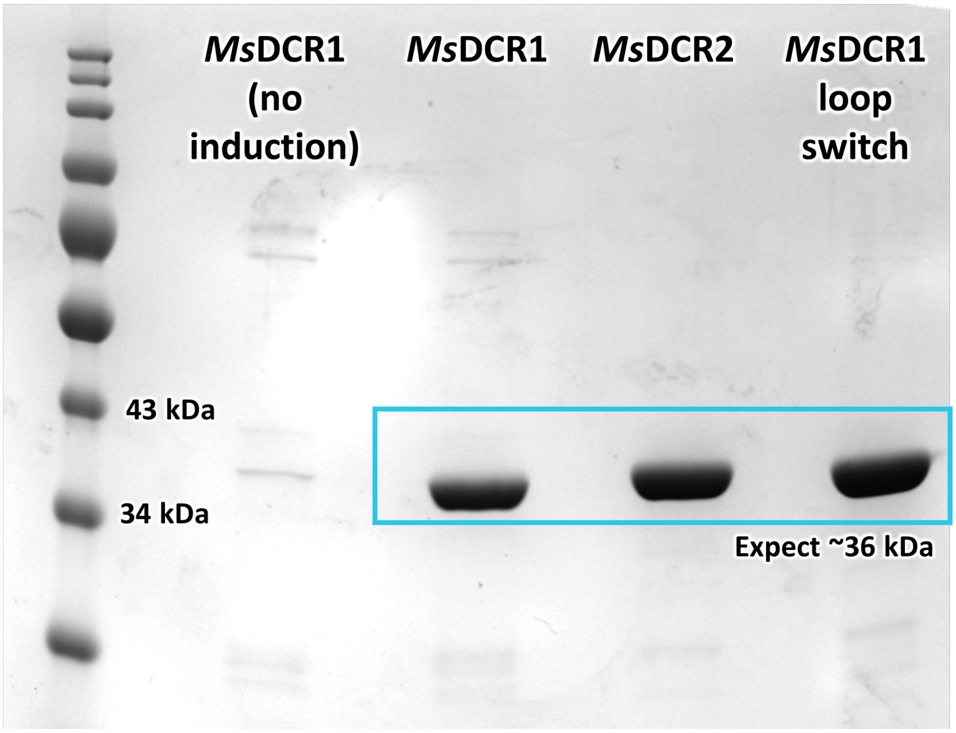

Supplementary Figure 21: SDS-page gel of purified DCR isoforms/variants from *E. coli*.

a) Results from *Ms*CO1 variants

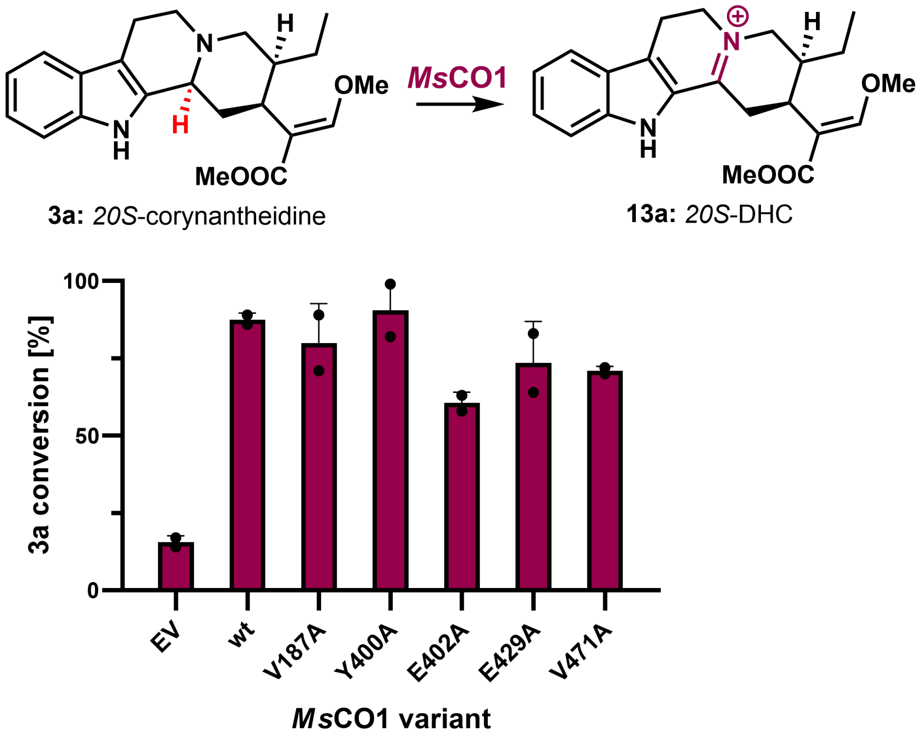

b) Docking of 4a in *Ms*CO1

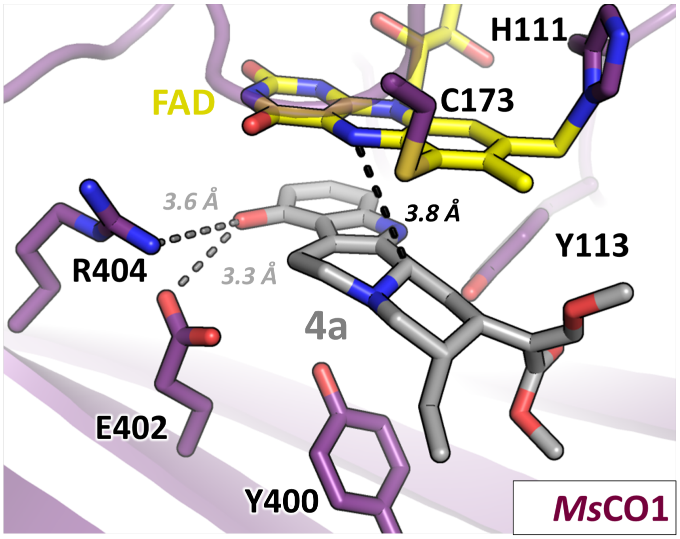

Supplementary Figure 22: a) Activity of *Ms*CO1 mutants. *Assay conditions:* 16 h 100 µM **3a** incubation into *N. benthamiana* leaves infiltrated with *Ms*CO1 mutated at the indicated position. Shown data are representative spectra from biological replicates (n = 2) with standard deviations depicted. **b)** Docking outcome of fitting **4a** within the active site of *Ms*CO1. Hydrogen bond contacts between the phenolic oxygen and E402/R404 are noted.

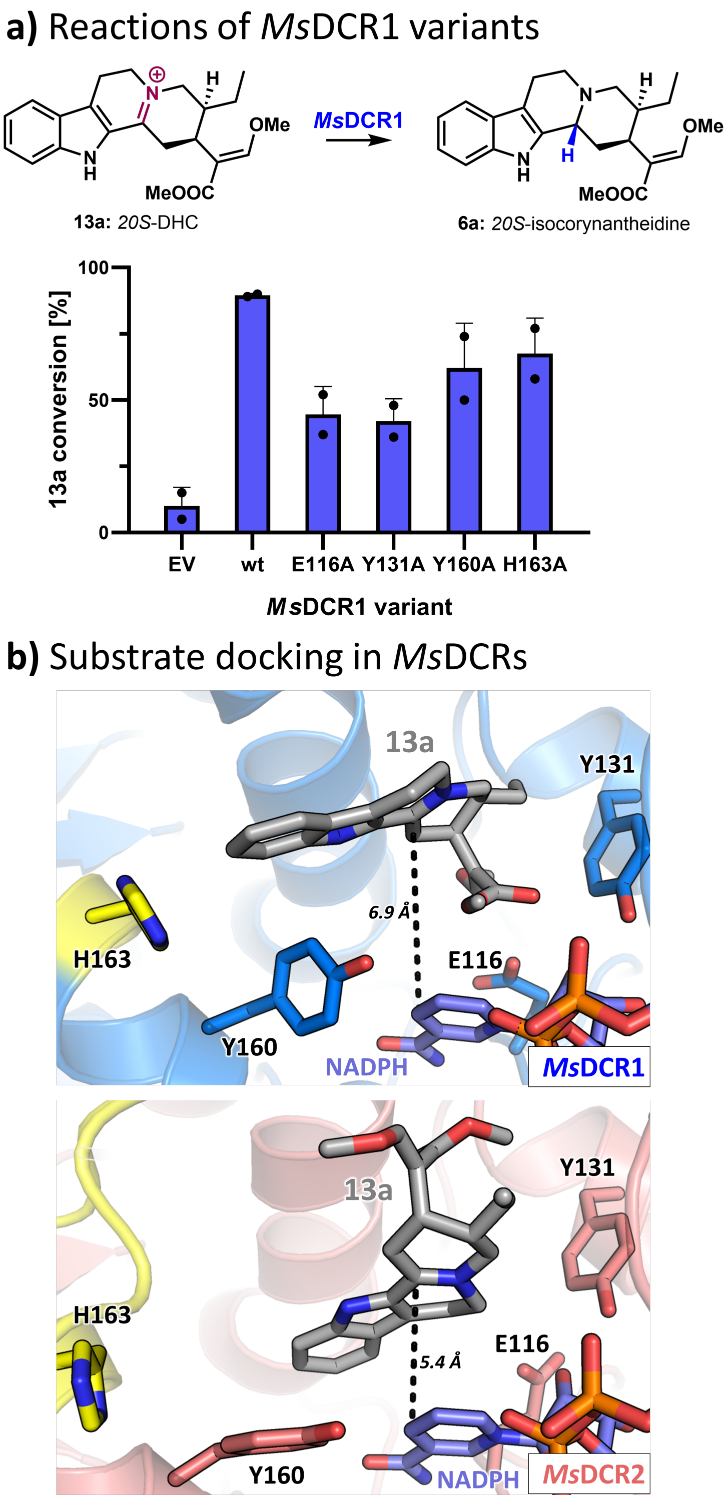

Supplementary Figure 23: Activity of *Ms*DCR1 mutants. **a)** *Assay conditions:* 16 h 100 µM **13a** incubation into *N. benthamiana* leaves infiltrated with *Ms*DCR1 mutated at the indicated position. Shown data are representative spectra from biological replicates (n = 2) with standard deviations depicted. **b)** The active site of *Ms*DCR2 is shown for comparison.

Results from *Ms*DCR1 loop switch variants

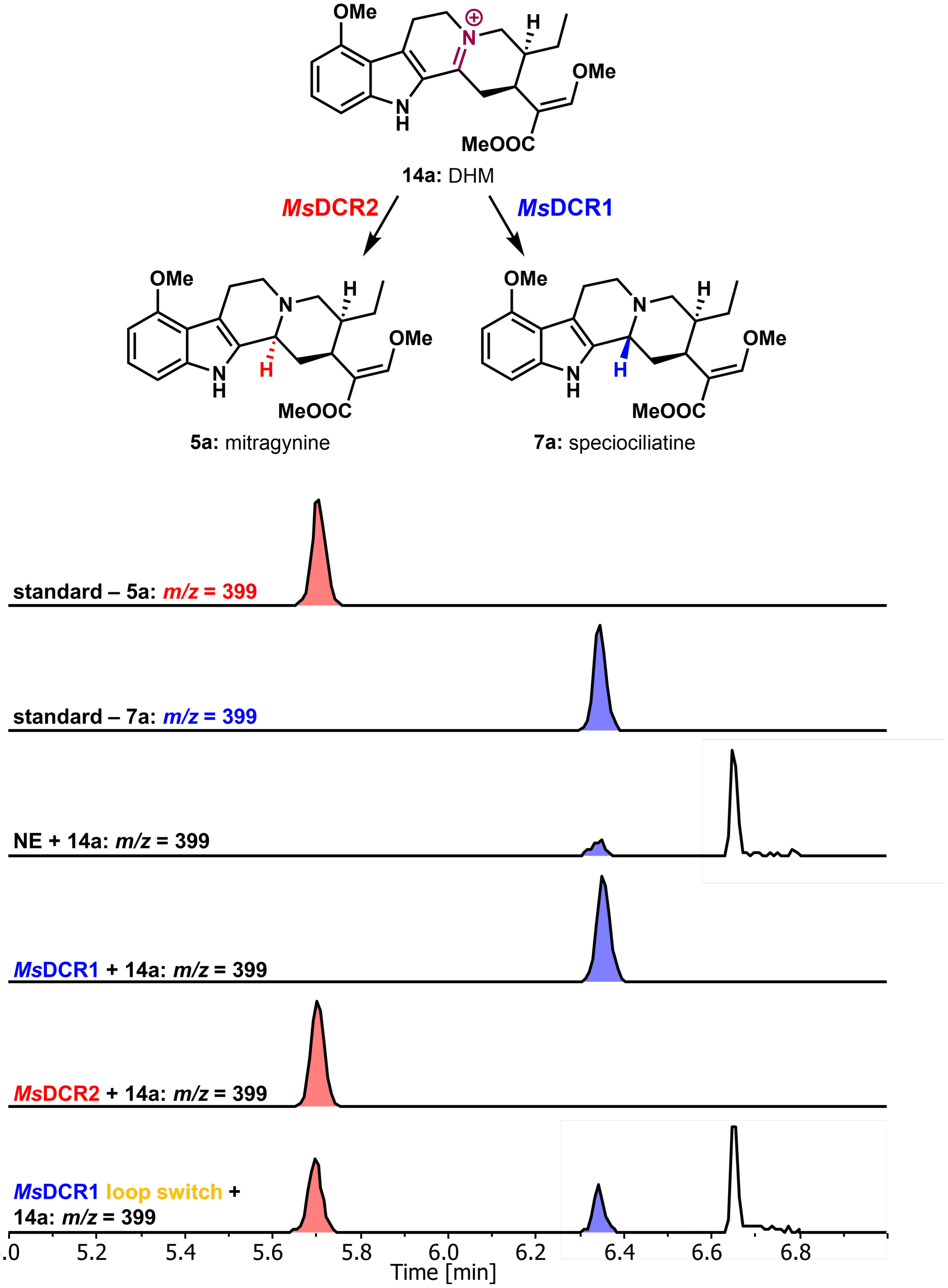

Supplementary Figure 24: Activity of *Ms*DCR1 loop switch mutant on **14a**. NE = no enzyme control. *Reaction conditions:* 100 µM **14a**, 100 µM NADPH, 1 µM *Ms*DCR isoform, 50 mM HEPES pH = 7.5, 25 ˚C for 4 h. Shown data are representative spectra from biological replicates (n = 3).

P450/oxidase SSN: alignment score threshold = 170

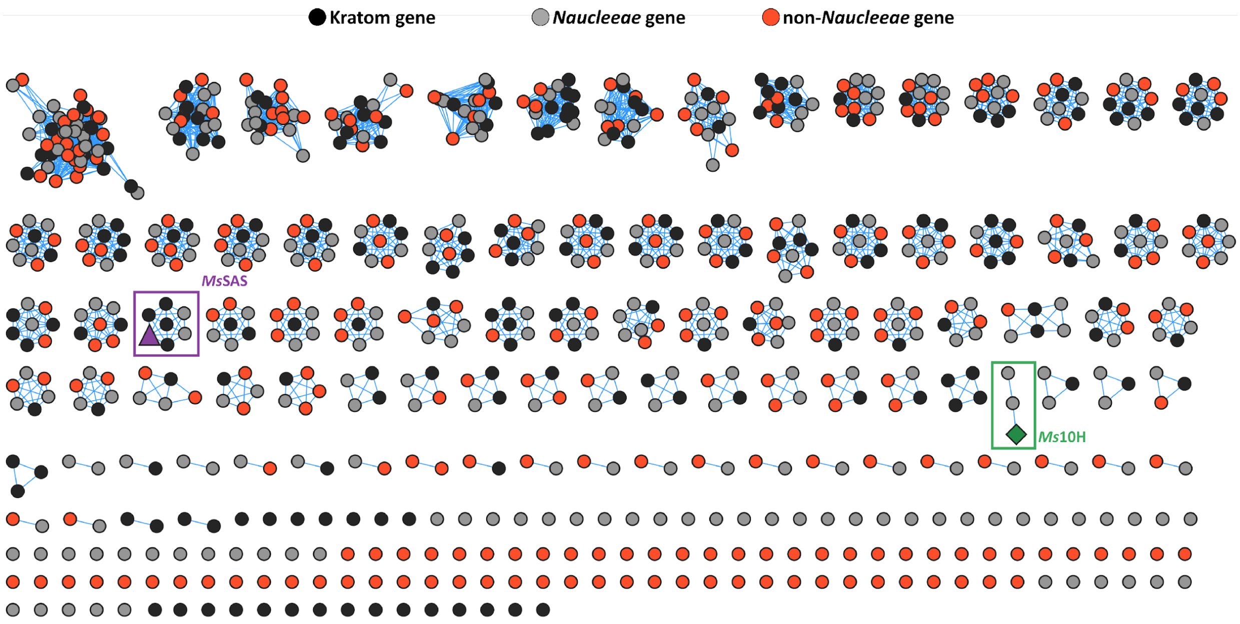

Supplementary Figure 25: Sequence similarity network (SSN) to identify *Naucleeae*-specific P450s/oxidases with alignment score threshold of 170. Kratom genes are shown in black, *Naucleeae* genes in gray, and non-*Naucleeae* genes in orange. The *Naucleeae*-specific cluster containing *Ms*10H is highlighted with a green box and the Naucleeae-specific cluster containing *Ms*SAS is highlighted with a purple box.

Discovery of *Ms*10H activity on DHC (**13a**)

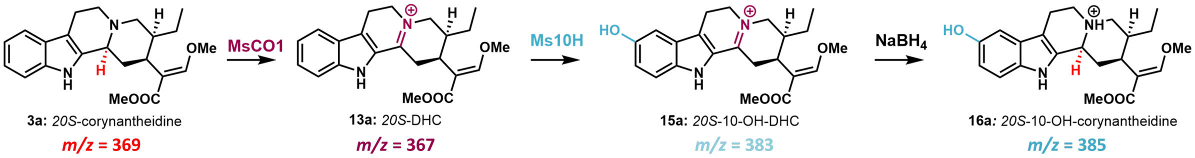

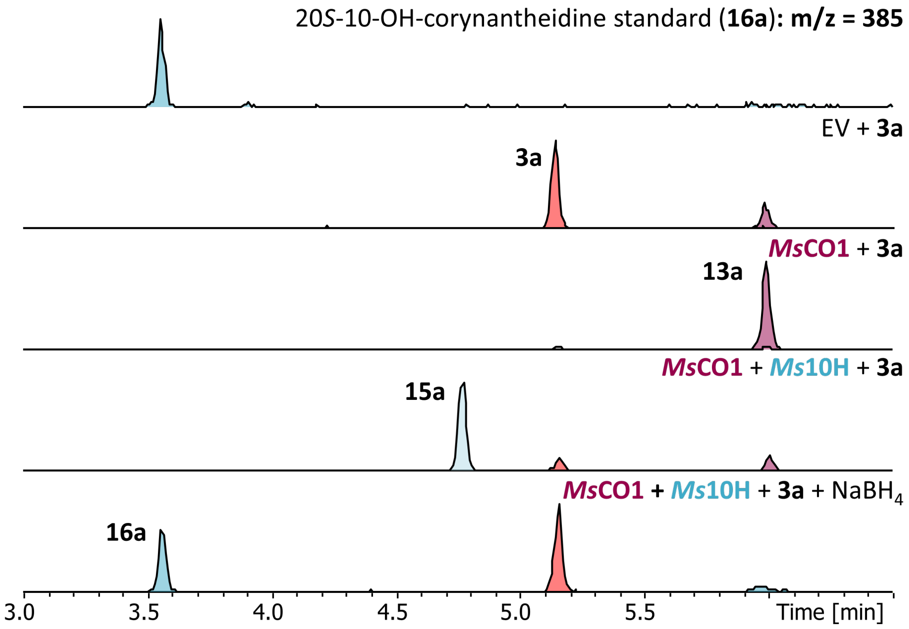

Supplementary Figure 26: *In planta* activity of *Ms*10H. LC-MS traces showing *Ms*10H is active on **13a** and is reduced via $NaBH_4$ to **16a**. *Assay conditions:* 16 h 100 µM **3a** incubation into *N. benthamiana* leaves infiltrated with indicated gene mixes. Shown data are representative spectra from biological replicates (n = 3). *$NaBH_4$ reduction:* 1 h incubation of half of methanol extract of infiltrated leaf disks with 10 mM $NaBH_4$ at r.t.

Overview of enzymatically produced spirooxindole compounds

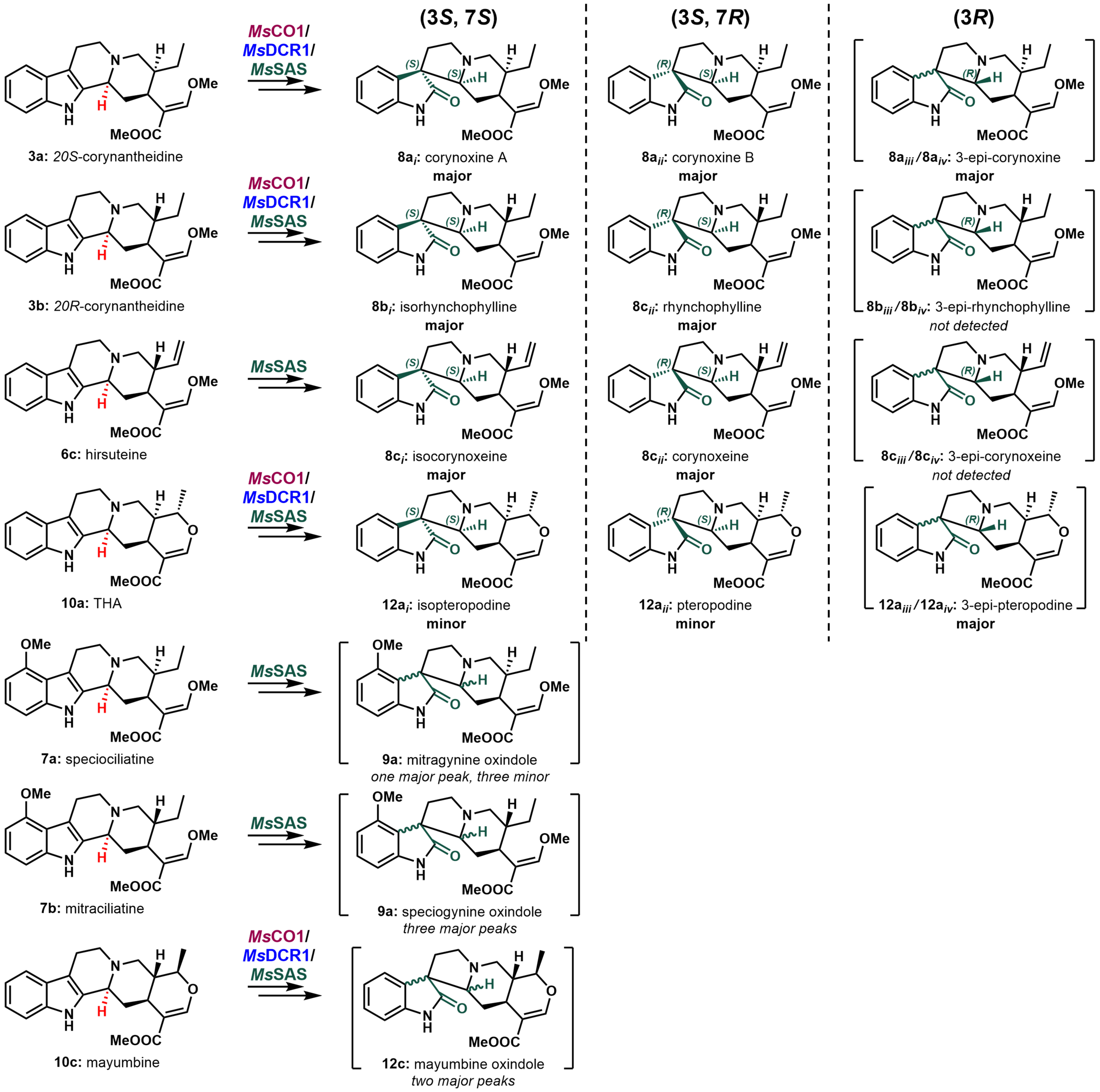

Supplementary Figure 27: Summative figure of substrate scope results (Supplementary Figures 28-38) with observed spirooxindole formation via *Ms*SAS and available standards. Compounds in brackets represent putatively identified compounds based on mass fragmentation and chemical reasoning. No standards for 3*R* spirooxindole compounds were available; identification between 3*R* isomers was not possible. **Major/minor**/*not detected* modifiers indicate relative peak abundance observed via LC-MS.

Activity of *Ms*SAS on hirsuteine (**6c**)

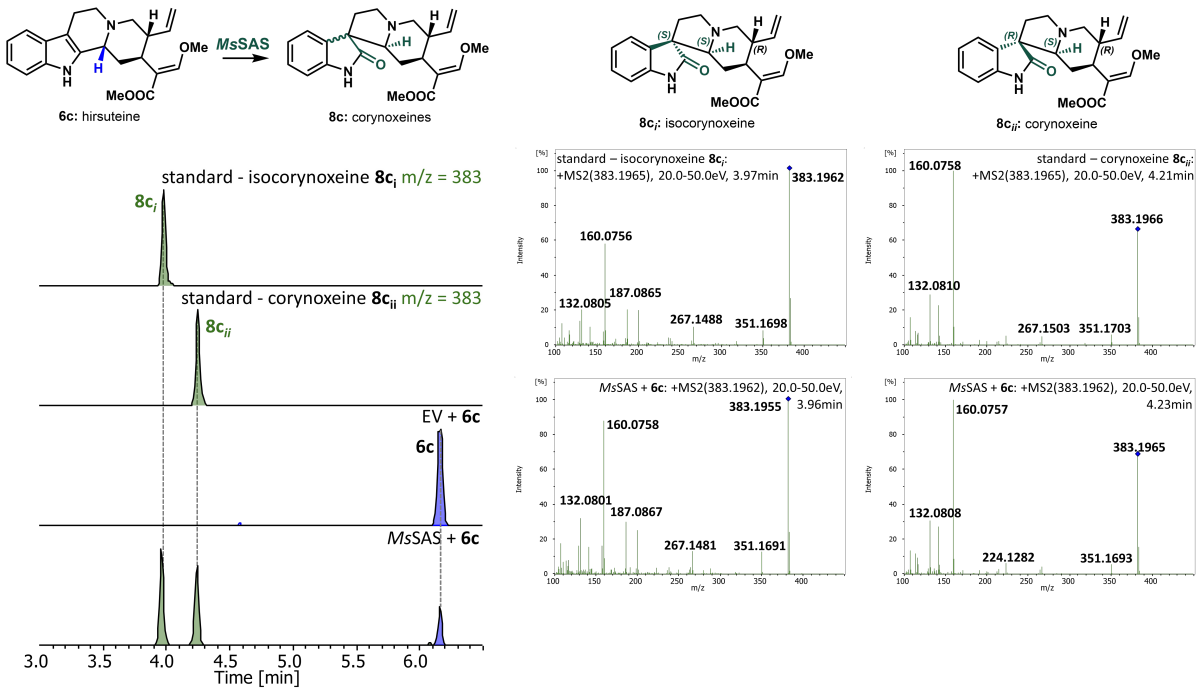

Supplementary Figure 28: Representative UPLC-MS traces and analysis of compounds produced from hirsuteine (**6c**) following reaction with *Ms*SAS in *N. benthamiana*. We note coelution and comparable MS2 fragmentation with authentic standards of isocorynoxeine **8c*i*** and corynoxeine **8c*ii***. In contrast to the previous report,[1] we assigned the products as corynoxeine and isocorynoxeine, not epi-corynoxeine (3*R*) based on NMR data. Assays were performed in biological triplicate (n = 3) for all reaction conditions.

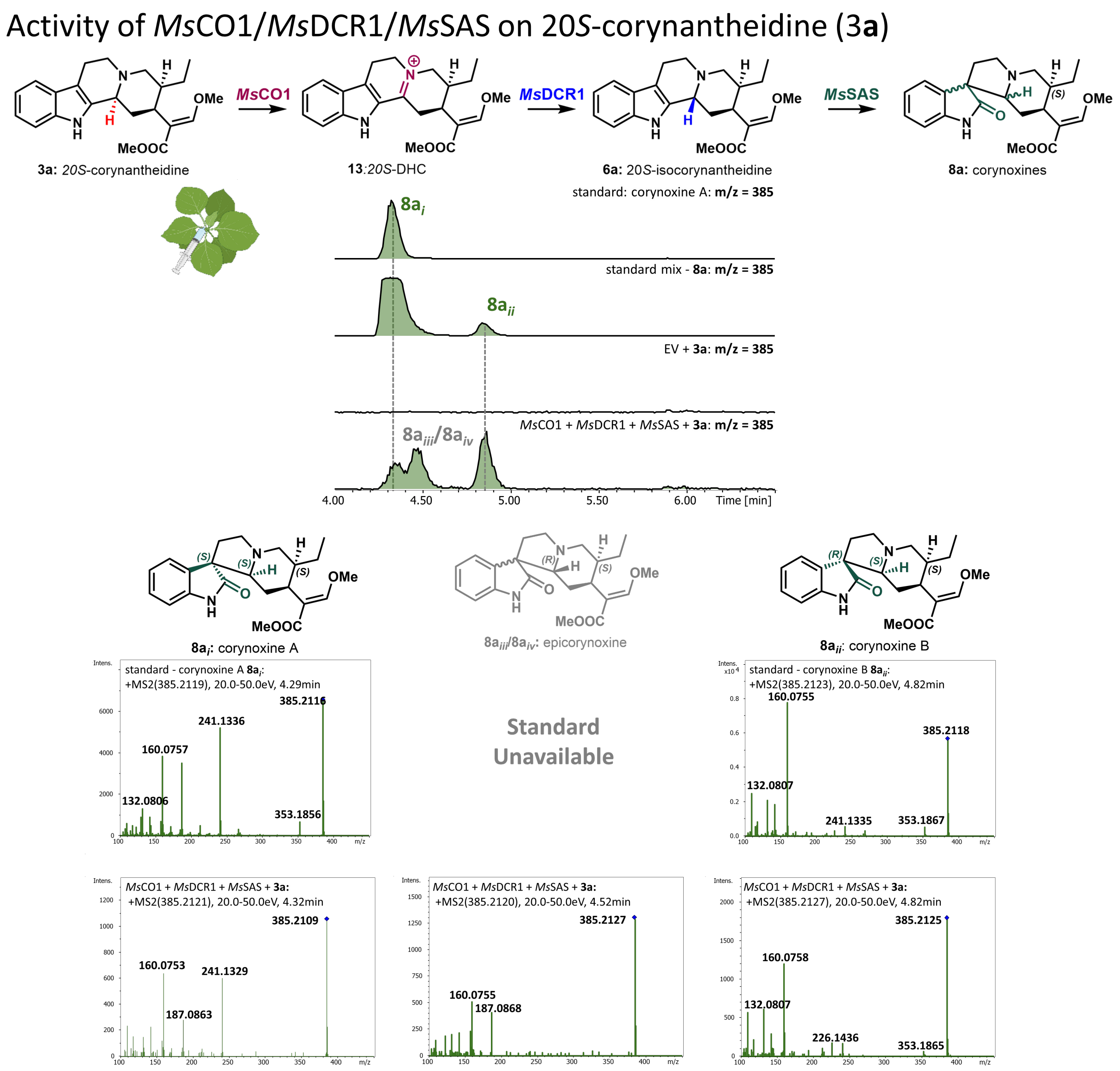

Supplementary Figure 29: Evaluation of spirooxindole alkaloids produced via *Ms*CO1/*Ms*DCR1/*Ms*SAS cascade reaction. Assays were performed in biological triplicate for all reaction conditions (n = 3). The 8a standard mix was overloaded to be able to visualize the minor isomer **8a$_{ii}$**.

Activity of *Ms*CO1/*Ms*DCR1/*Ms*SAS on 20*S*-9-hydroxycorynantheidine (**4a**)

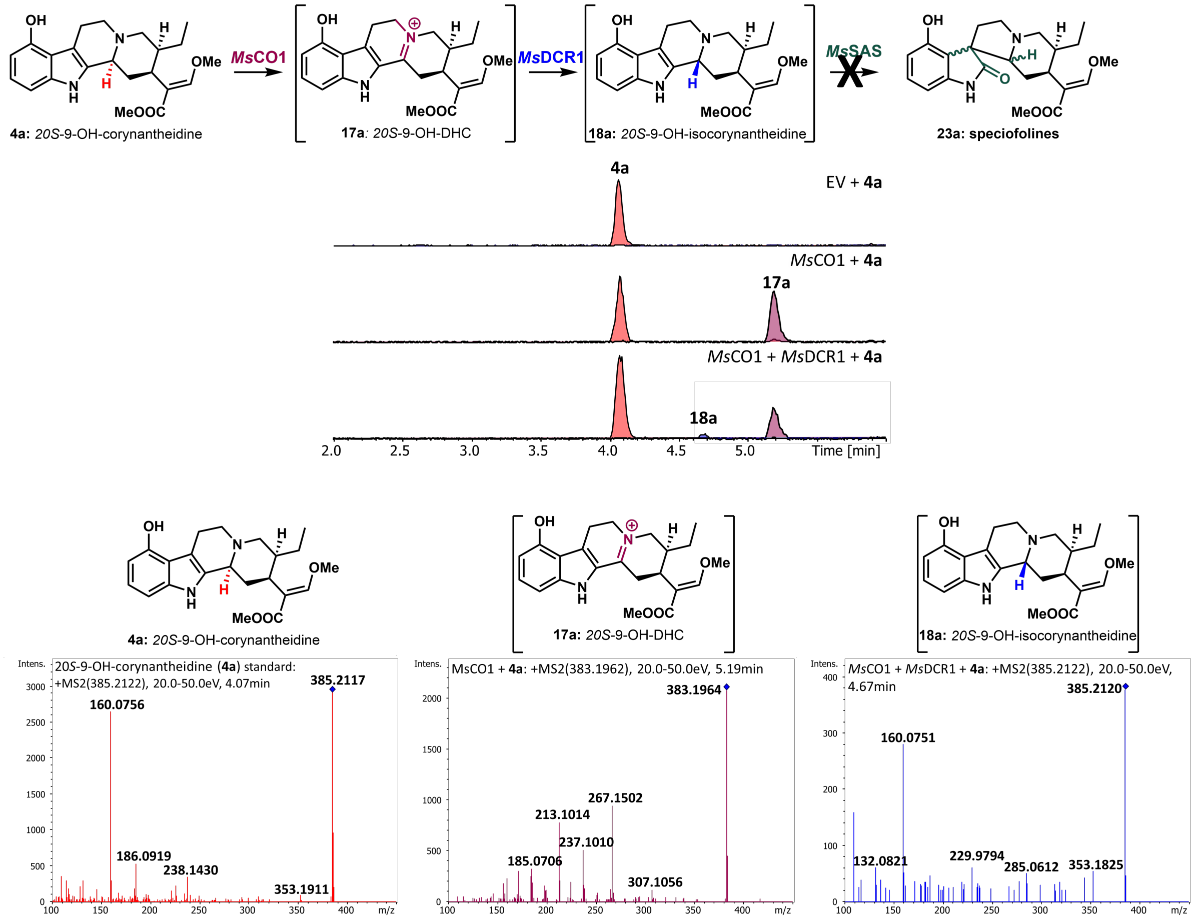

Supplementary Figure 30: Representative UPLC-MS traces and analysis of compounds produced from a (20*S*)-9-hydroxycorynantheidine (**4a**) standard following reaction with *Ms*CO1-*Ms*DCR-*Ms*SAS in *N. benthamiana*. A minor peak for a compound with a mass corresponding to (20*S*)-9-OH-isocorynantheidine was detected, with similar fragmentation to the (20*S*)-9-OH-corynantheidine standard. Assays were performed in biological triplicate for all reaction conditions (n = 3). Compounds in brackets were putatively assigned without an authentic standard based on mass fragmentation.

Activity of *Ms*CO1/*Ms*DCR1/*Ms*SAS on DHM (**14a**)

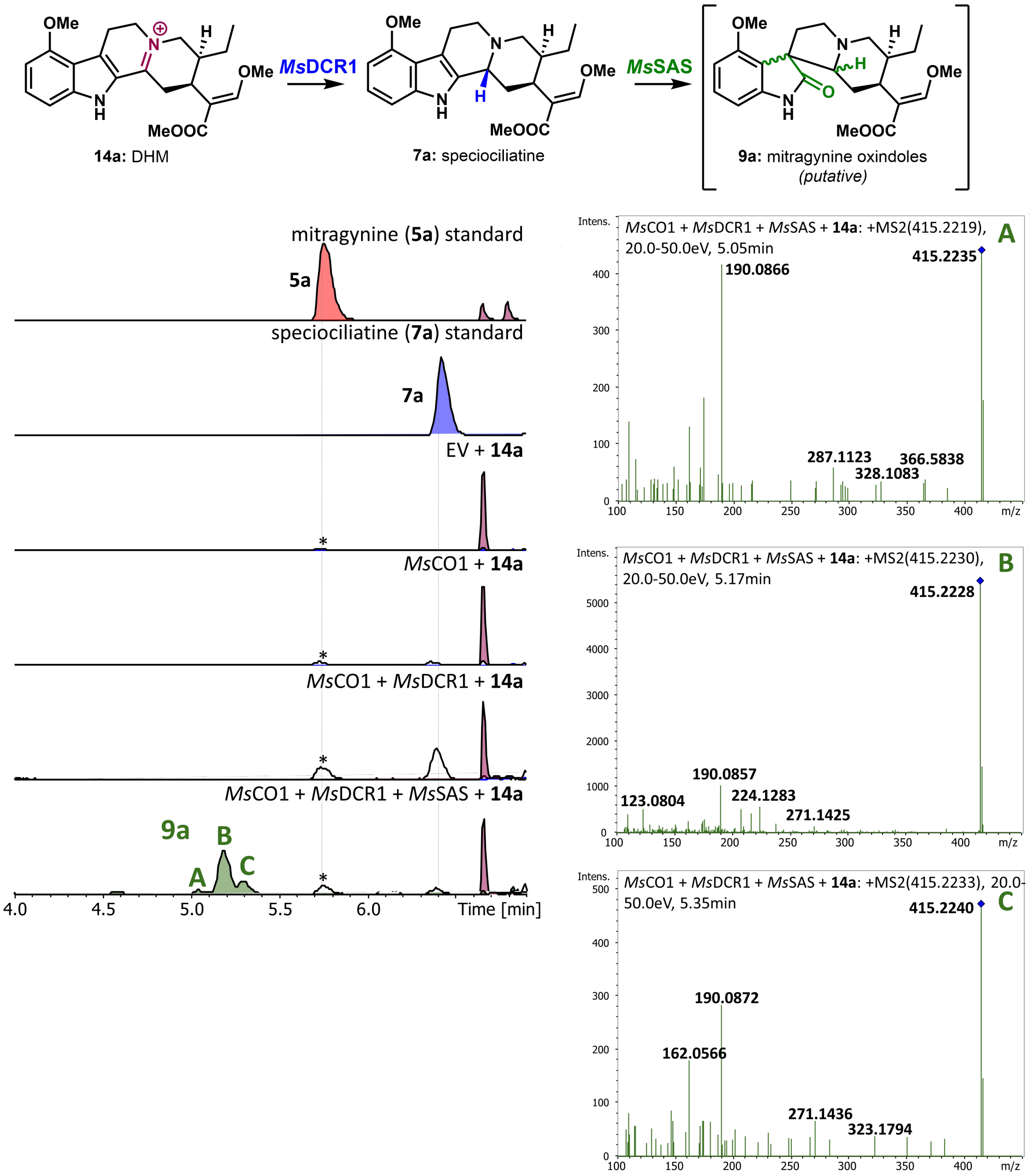

Supplementary Figure 31: Representative UPLC-MS traces and analysis of compounds produced from synthesized 3-dehydromitragynine (DHM, **14a**) following reaction with *Ms*CO1-*Ms*DCR-*Ms*SAS in *N. benthamiana*. Due to lack of standards, we did not attempt to assign structures to the observed mitragynine oxindoles (**9a**), but mass fragmentation is consistent with methoxylated oxindoles. *A small mitragynine (**5a**) impurity was present in the original DHM substrate. Assays were performed in biological triplicate for all reaction conditions (n = 3).

a) Activity of *Ms*SAS on speciociliatine (**7a**)

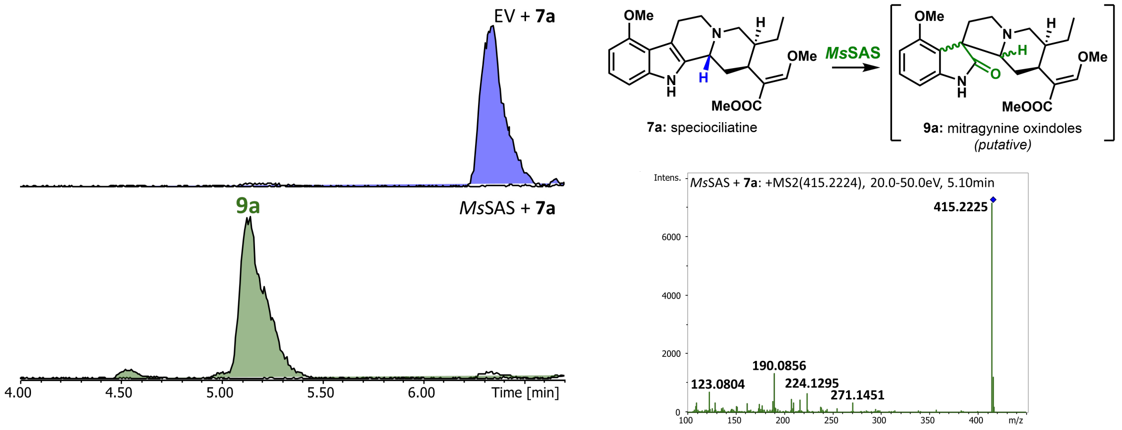

b) Activity of *Ms*SAS on mitraciliatine (**7b**)

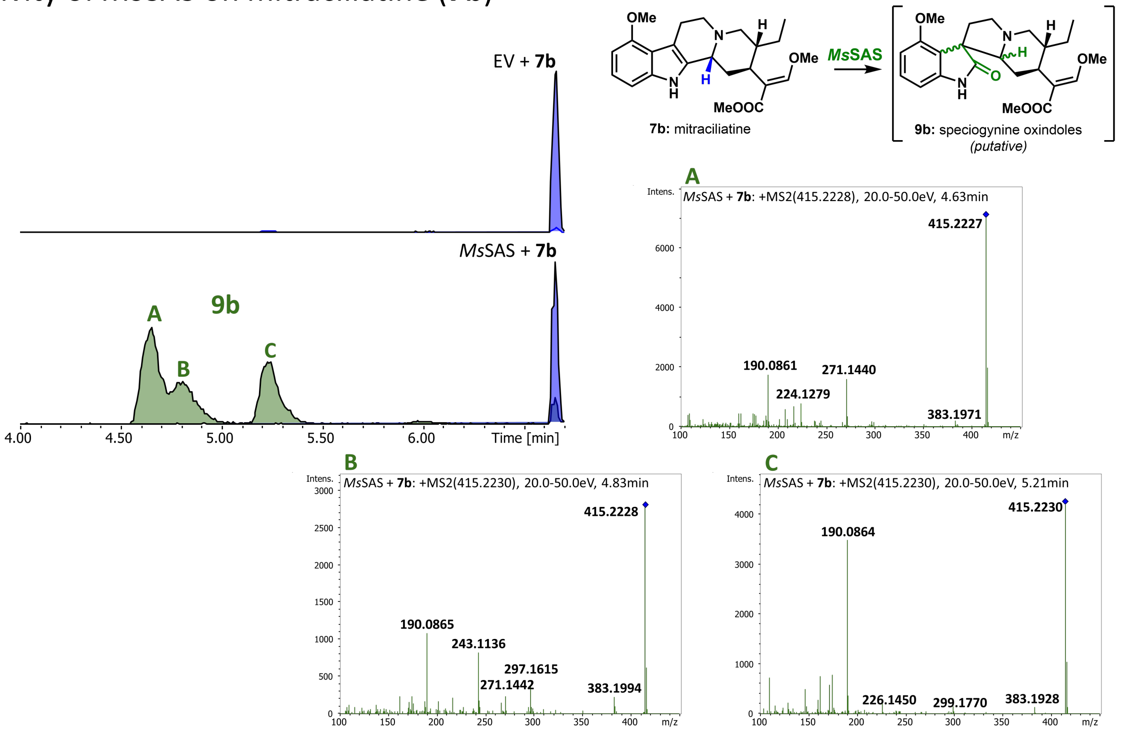

Supplementary Figure 32: a) Representative UPLC-MS traces and analysis of compounds produced from speciociliatine (**7a**) following reaction with *Ms*SAS in *N. benthamiana*. We note the same major putative mitragynine oxindole product (**9a**) (designated in brackets) as from reaction with DHM (**14a**) with matching mass fragmentation patterns (see **Supplementary Figure 31**). In contrast to *Ms*SAS products from non-methoxylated 20*S* substrates, reaction with speciociliatine (**7a**) produces primarily only a single major peak, although minor peaks are also observed. **b)** Representative UPLC-MS traces and analysis of compound produced from mitraciliatine (**7b**) following reaction with *Ms*CO1-*Ms*DCR-*Ms*SAS in *N. benthamiana*. Due to lack of standards, we did not attempt to assign structures to the observed speciogynine oxindoles (**9b**) (designated in brackets), but mass fragmentation is consistent with methoxylated oxindoles. Again, in contrast to *Ms*SAS products from non-methoxylated 20*R* substrates, reaction with mitraciliatine (**7b**) produces three major peaks, producing a mixture of 3*S*/3*R* isomers. Assays were performed in biological triplicate for all reaction conditions (n = 3).

Activity of *Ms*CO1/*Ms*DCR1/*Ms*SAS on tetrahydroalstonine (**10a**)

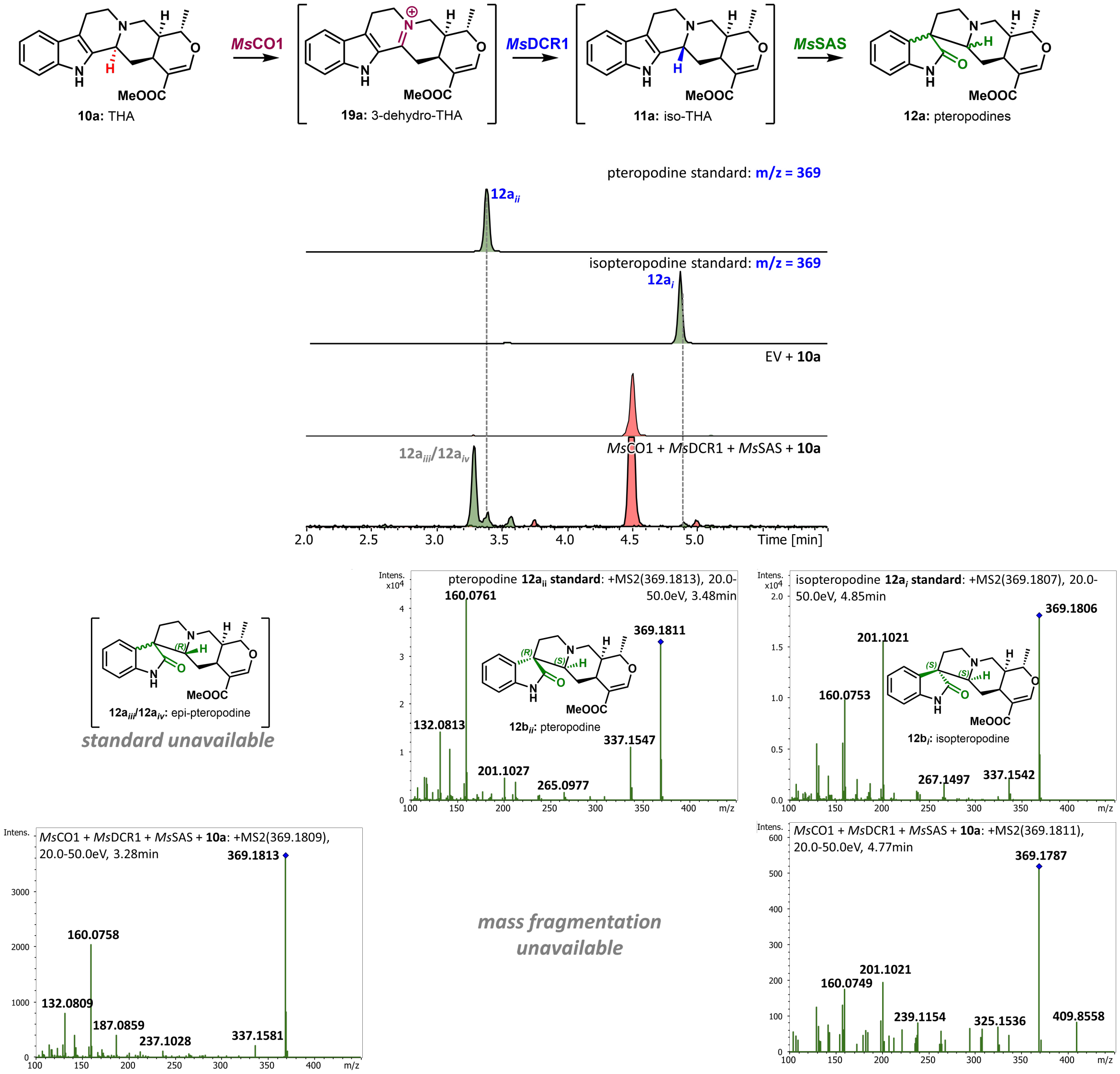

Supplementary Figure 33: Representative UPLC-MS traces and analysis of pteropodine **12a** isomers produced from commercially available tetrahydroalstonine (THA) following reaction with *Ms*CO1-*Ms*DCR1-*Ms*SAS in *N. benthamiana*. Compounds in brackets were putatively assigned without an authentic standard based on mass fragmentation. Assays were performed in biological triplicate for all reaction conditions (n = 3).

Activity of *Ms*CO1/*Ms*DCR1/*Ms*SAS on ajmalicine (**10b**)

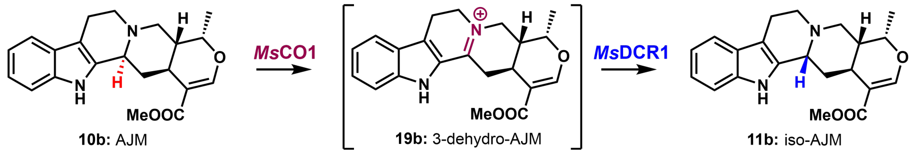

a) *in planta* assay results

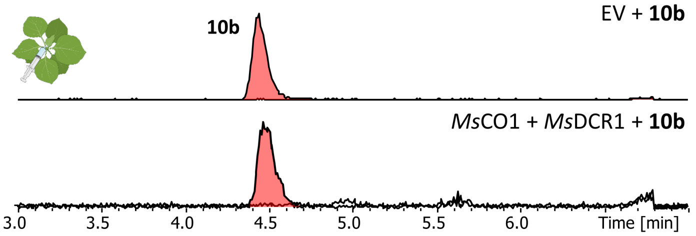

b) *in intro* assay results

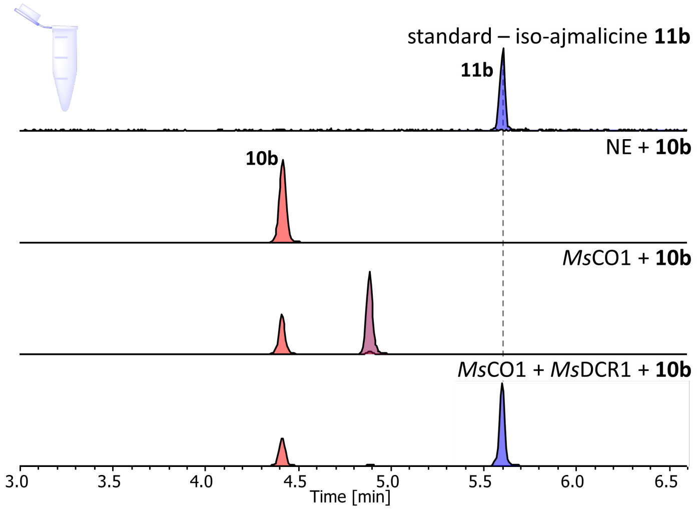

Supplementary Figure 34: Representative UPLC-MS traces and analysis of reaction of *Ms*CO1 and *Ms*DCR1 with commercially available ajmalicine (**10b**, AJM). **a)** *In planta* reactions in *N. benthamiana*. No products were observed. Shown data are representative spectra from biological replicates (n = 3). This experiment was repeated several times with identical results. **b)** Formation of isoajmalicine (**11b**, iso-AJM) was conversely observed from *in vitro* reactions. Compounds in brackets were putatively assigned without an authentic standard based on mass fragmentation. *In vitro* reaction conditions: 25 µL total volume containing 100 µM ajmalicine (**10b**), 1 mM NADPH, 1 µM *Ms*CO1 and 1 µM *Ms*DCR, and 50 mM HEPES pH = 7.5. Reactions were allowed to proceed at 25 ˚C for 4 h. Shown data are representative spectra from technical replicates (n = 3).

Activity of *Ms*CO1/*Ms*DCR1/*Ms*SAS on mayumbine (**10c**)

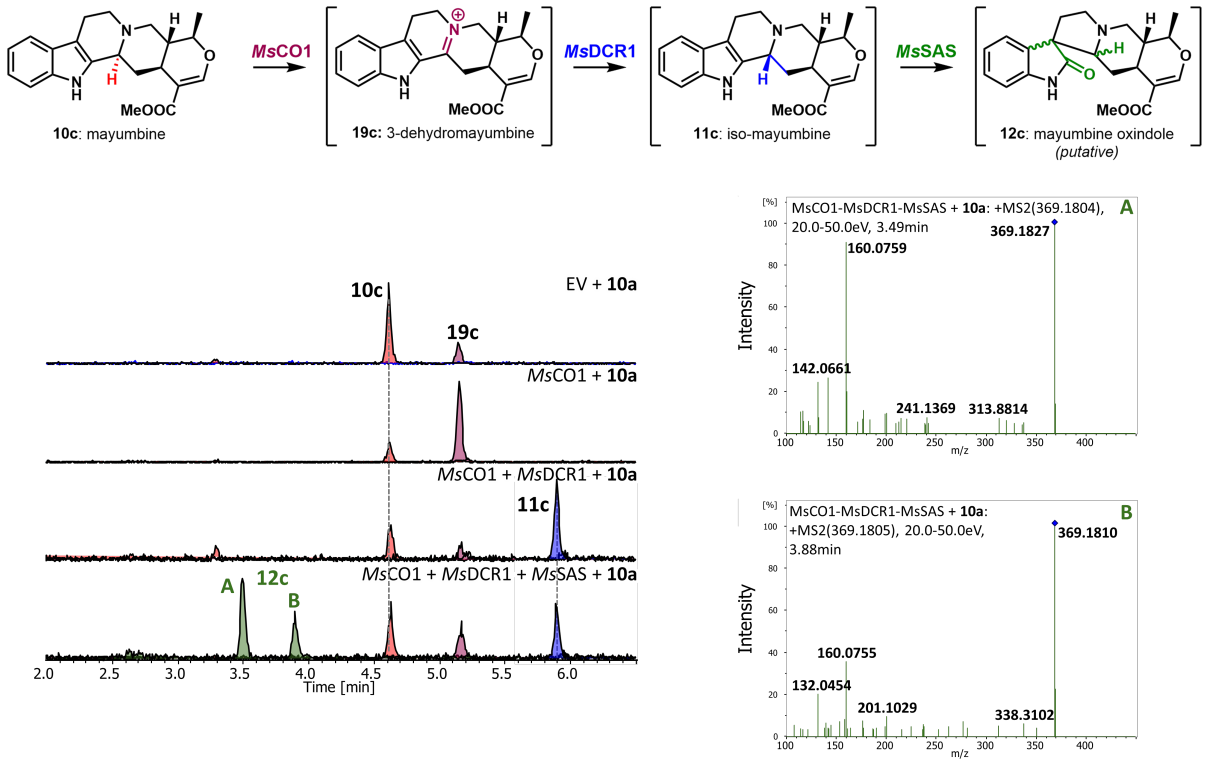

Supplementary Figure 35: Representative UPLC-MS traces and analysis of compounds produced from commercially available mayumbine (**10c**) following reaction with *Ms*CO1-*Ms*DCR1-*Ms*SAS in *N. benthamiana*. Only two putative mayumbine oxindole products (**12c**) were observed, but mass fragmentation is consistent with oxindoles: (*m/z* $[M+H]^+ = 160$). Compounds in brackets were putatively assigned without an authentic standard based on mass fragmentation. Assays were performed in biological triplicate for all reaction conditions (n = 3).

Activity of *Ms*CO1/*Ms*DCR1/*Ms*SAS on yohimbine (**20a**)

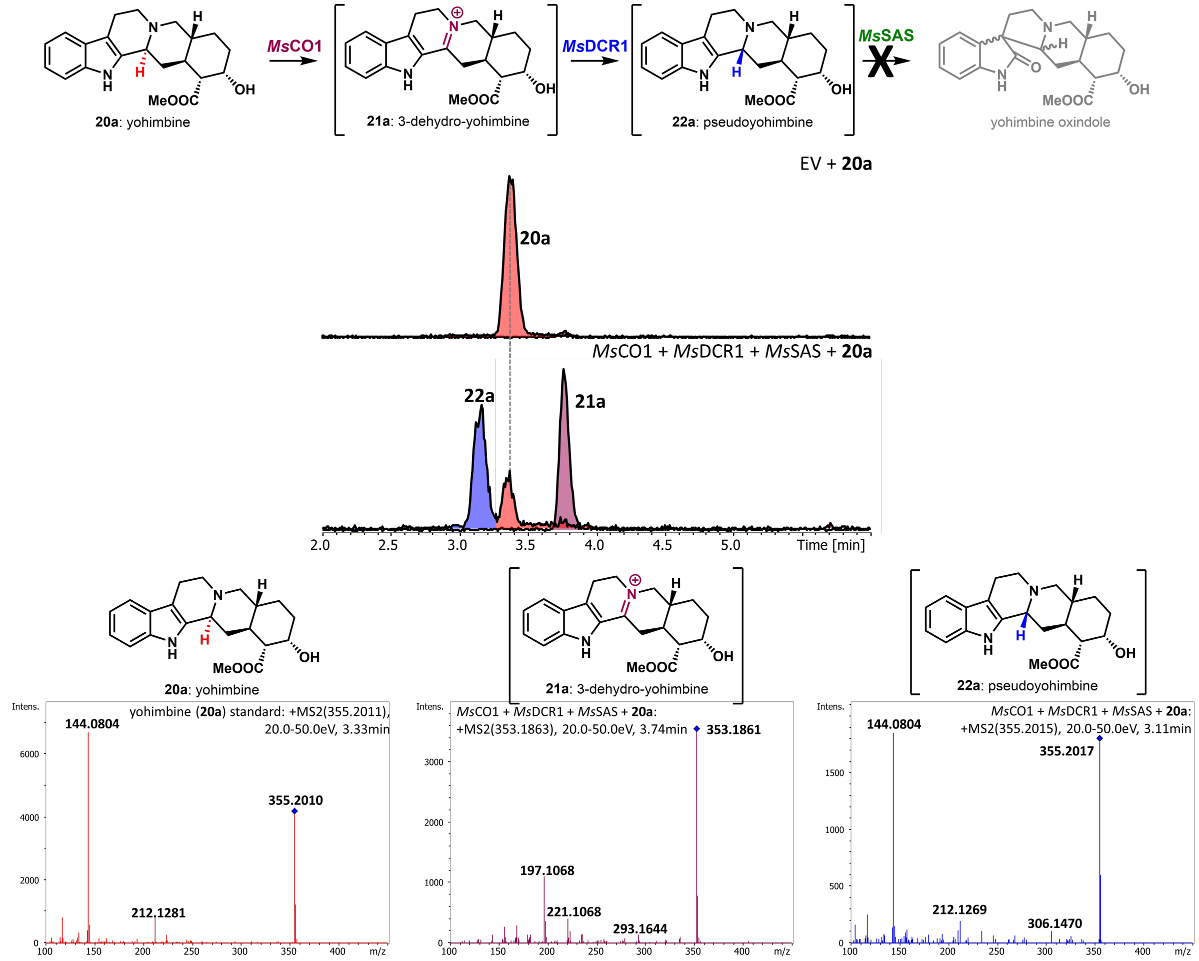

Supplementary Figure 36: Representative UPLC-MS traces and analysis of compounds produced from commercially available yohimbine (**20a**) following reaction with *Ms*CO1-*Ms*DCR1-*Ms*SAS in *N. benthamiana*. We note formation of a putative 3*R* epimer of yohimbine, pseudoyohimbine (**22a**) with comparable mass fragmentation to **20a**. Compounds in brackets were putatively assigned without an authentic standard based on mass fragmentation. Assays were performed in biological triplicate for all reaction conditions (n = 3).

Activity of *Ms*CO1/*Ms*DCR/*Ms*SAS on rauwolscine (**20b**)

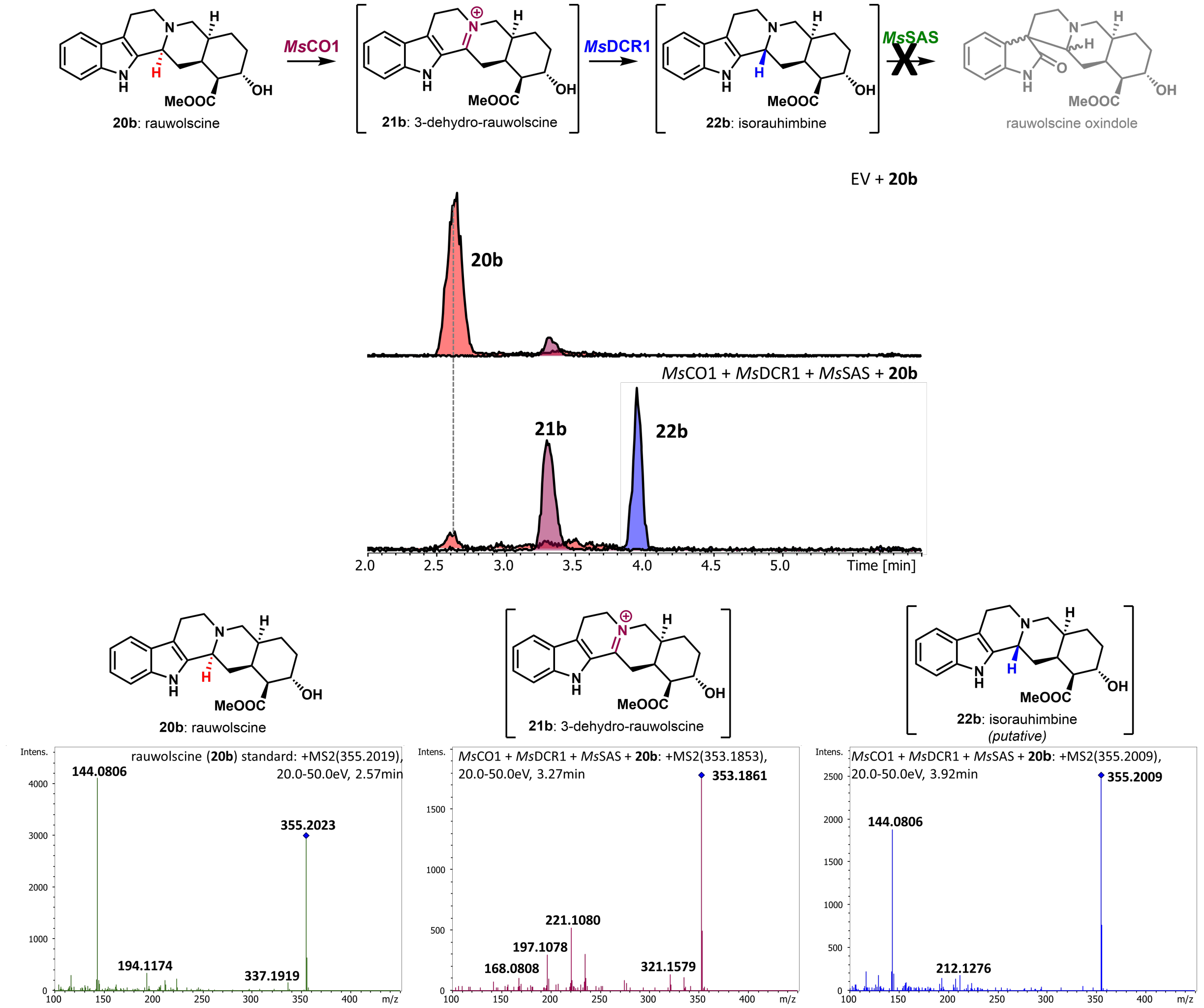

Supplementary Figure 37: Representative UPLC-MS traces and analysis of compounds produced from commercially available rauwolscine (**20b**) following reaction with *Ms*CO1-*Ms*DCR1-*Ms*SAS in *N. benthamiana*. We note formation of a putative 3*R* epimer of rauwolscine, isorauhimbine (**22b**). Compounds in brackets were putatively assigned without an authentic standard based on mass fragmentation. Assays were performed in biological triplicate for all reaction conditions (n = 3).

Activity of *Ms*CO1/*Ms*DCR1/*Ms*SAS on corynanthine (**20c**)

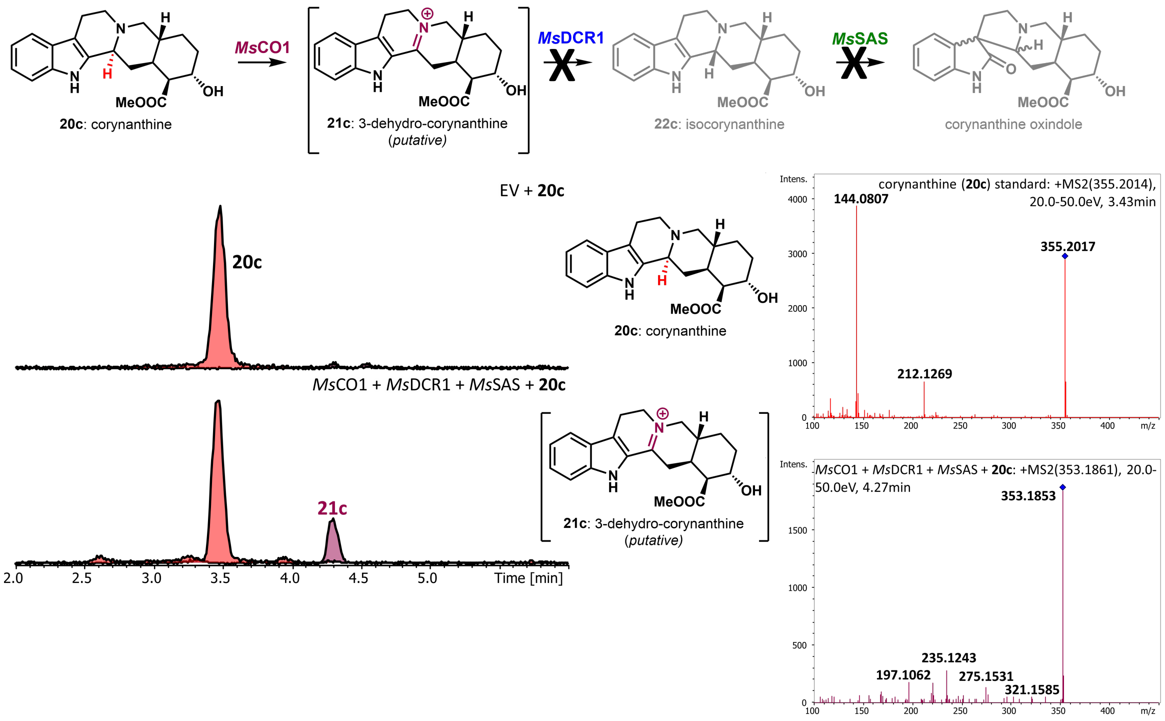

Supplementary Figure 38: Representative UPLC-MS traces and analysis of compounds produced from commercially available corynanthine (**19c**) following reaction with *Ms*CO1-*Ms*DCR1-*Ms*SAS in *N. benthamiana*. We note formation of a compound corresponding to a putative 3-dehydrocorynanthine (**21c**) but no significant formation of isocorynanthine (**22c**). Compounds in brackets were putatively assigned without an authentic standard based on mass fragmentation. Assays were performed in biological triplicate for all reaction conditions (n = 3).

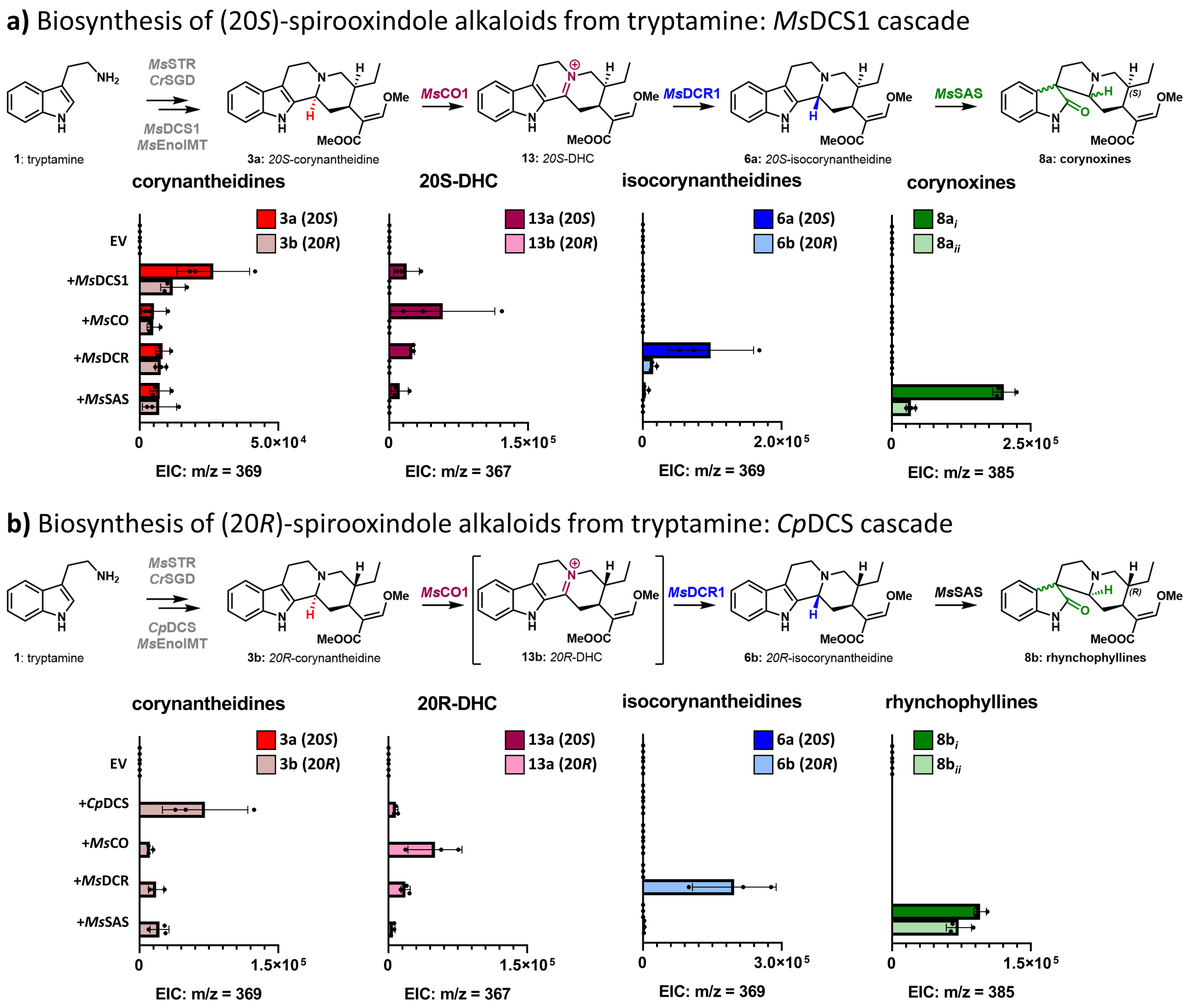

Supplementary Figure 39: Pathway reconstruction in *N. benthamiana* from tryptamine to spirooxindole alkaloids with peak areas from extracted ion chromatograms (EICs) for provided *m/z* values. A) 20*S* biosynthetic pathway utilizing *Ms*DCS1. B) 20*R* biosynthetic pathway utilizing *Cp*DCS. Compounds in brackets were putatively assigned without an authentic standard based on mass fragmentation. Assays were performed in biological triplicate for all reaction conditions (n = 3).

Biosynthesis of spirooxindole alkaloids from tryptamine: *Ms*DCS1 cascade

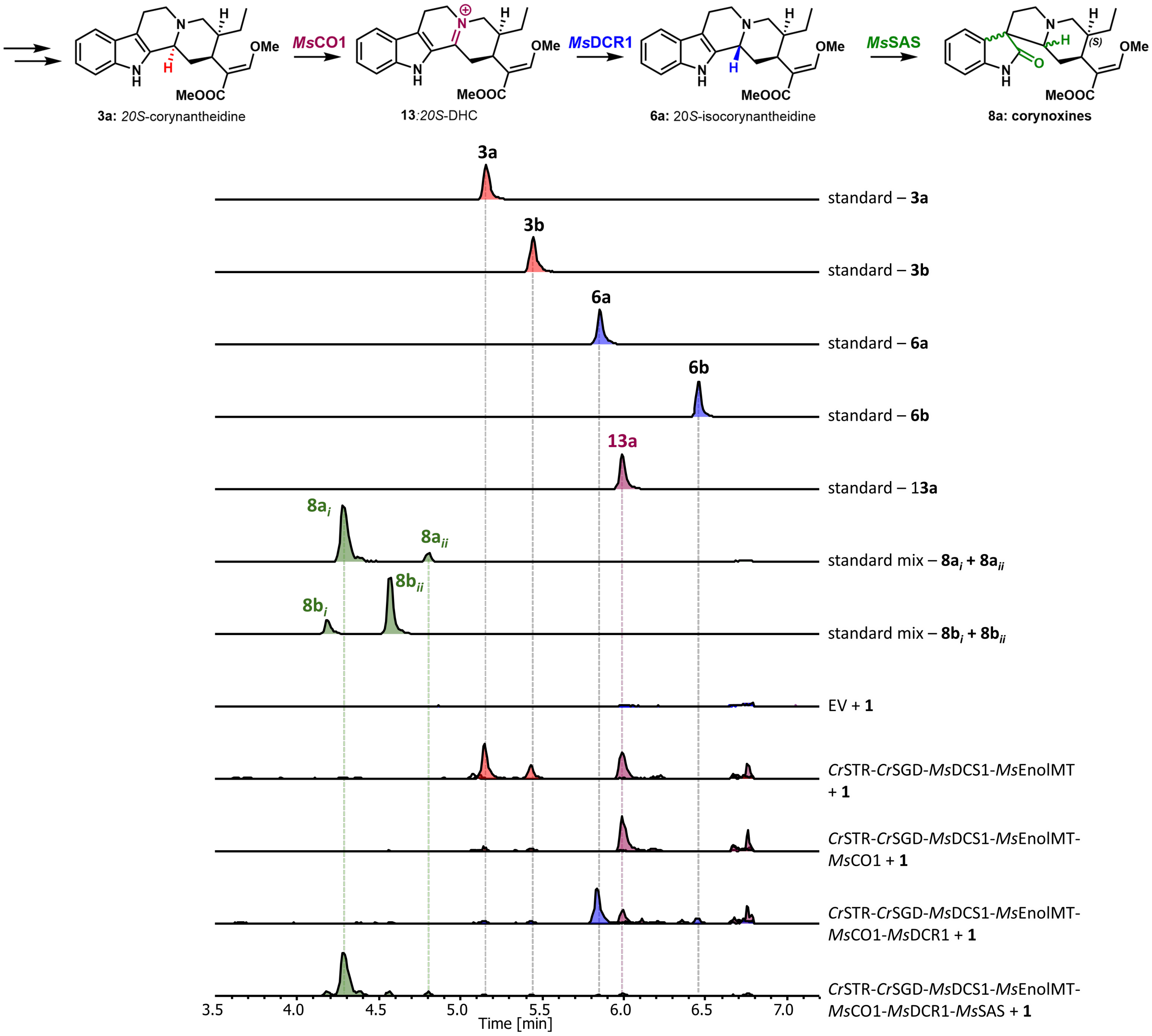

Supplementary Figure 40: Representative UPLC-MS traces from pathway reconstruction for *Ms*DCS1 cascade corresponding to **Supplementary Figure 39**. The major identified peak corresponded to corynoxine A **8a*i***. Peaks corresponding to **8a*ii***, **8b*i***, and **8b*ii*** were also observed. Additionally, the **8a*ii*** standard was found to isomerize relatively quickly to **8a*i***, as previously described.[2] Reported data are representative spectra from biological replicates (n = 3). See **Supplementary Figure 29** for additional spectra detailing *Ms*CO1/*Ms*DCR1/*Ms*SAS activity on (20S)-corynantheidine (**3a**).

Biosynthesis of spirooxindole alkaloids from tryptamine: *Cp*DCS cascade

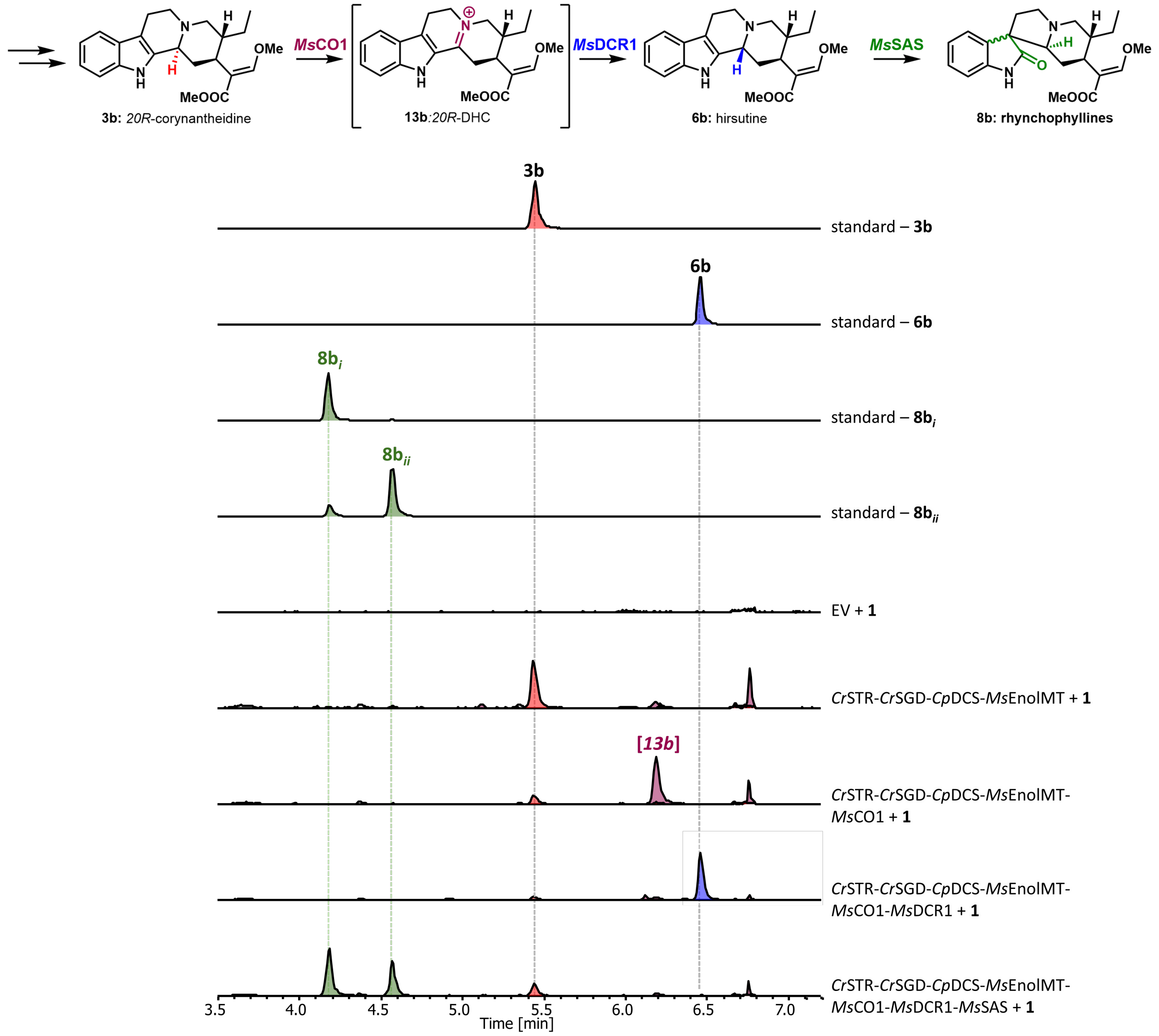

Supplementary Figure 41: Representative UPLC-MS traces from pathway reconstruction for *Cp*DCS cascade corresponding to **Supplementary Figure 39**. Two major spirooxindole peaks could be observed, corresponding to isorhynchophylline **8b*i*** and rhynchophylline **8b*ii*** standards. The **8b*ii*** standard was found to isomerize to **8b*i***. Reported data are representative spectra from biological replicates (n = 3).Compounds in brackets were putatively assigned without an authentic standard based on mass fragmentation.

20*S*-9-hydroxy-dehydrocorynantheidine (**17a**) identified in Kratom young leaves

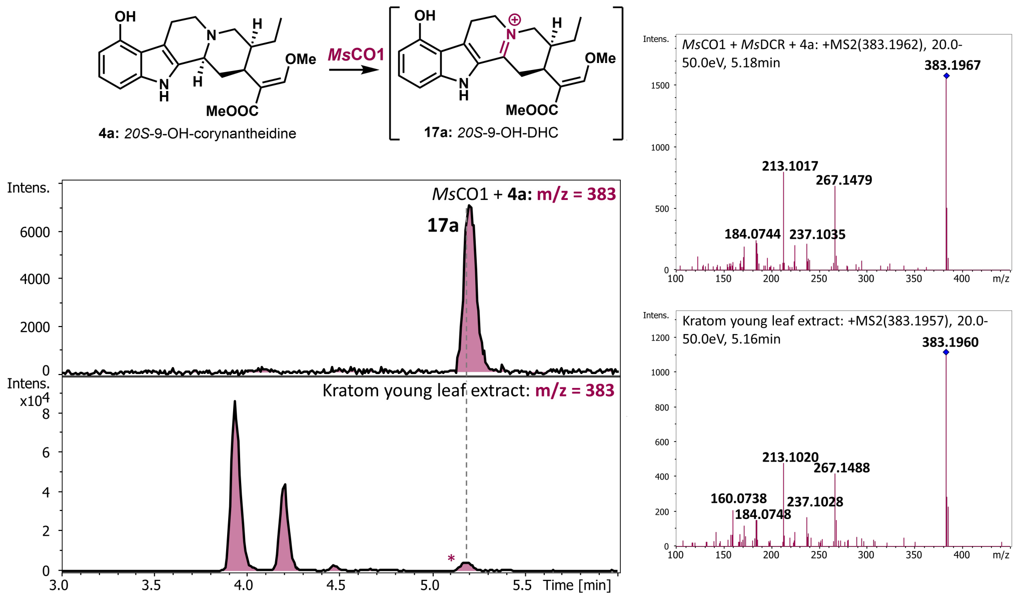

Supplementary Figure 42: Comparison of enzymatically produced (20*S*)-3-dehydro-9-hydroxycorynantheidine (**17a**) with Kratom young leaf extract.

Isomerization mechanism of rhynchophyllines: **8b**

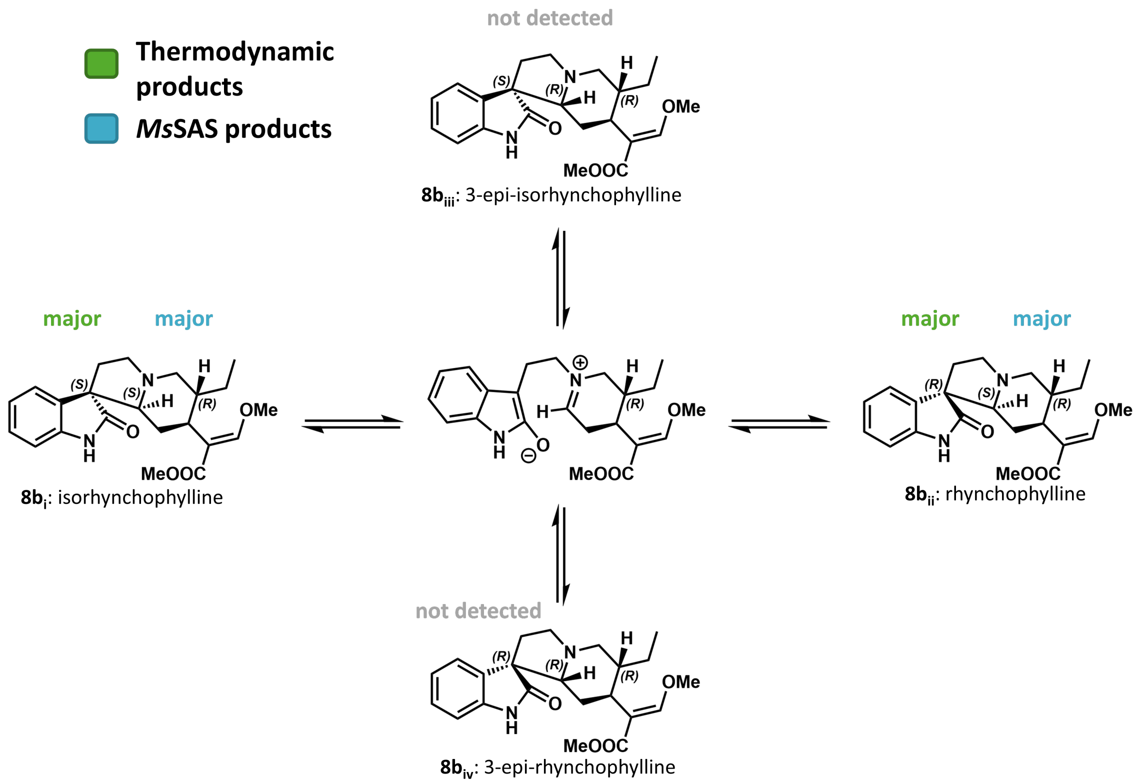

Supplementary Figure 43: Comparison of observed products from *Ms*SAS originating from hirsutine (**6b**) and proposed thermodynamic distribution of product isomerization (see Laus 1996).[2]

Isomerization mechanism of corynoxines: **8a**

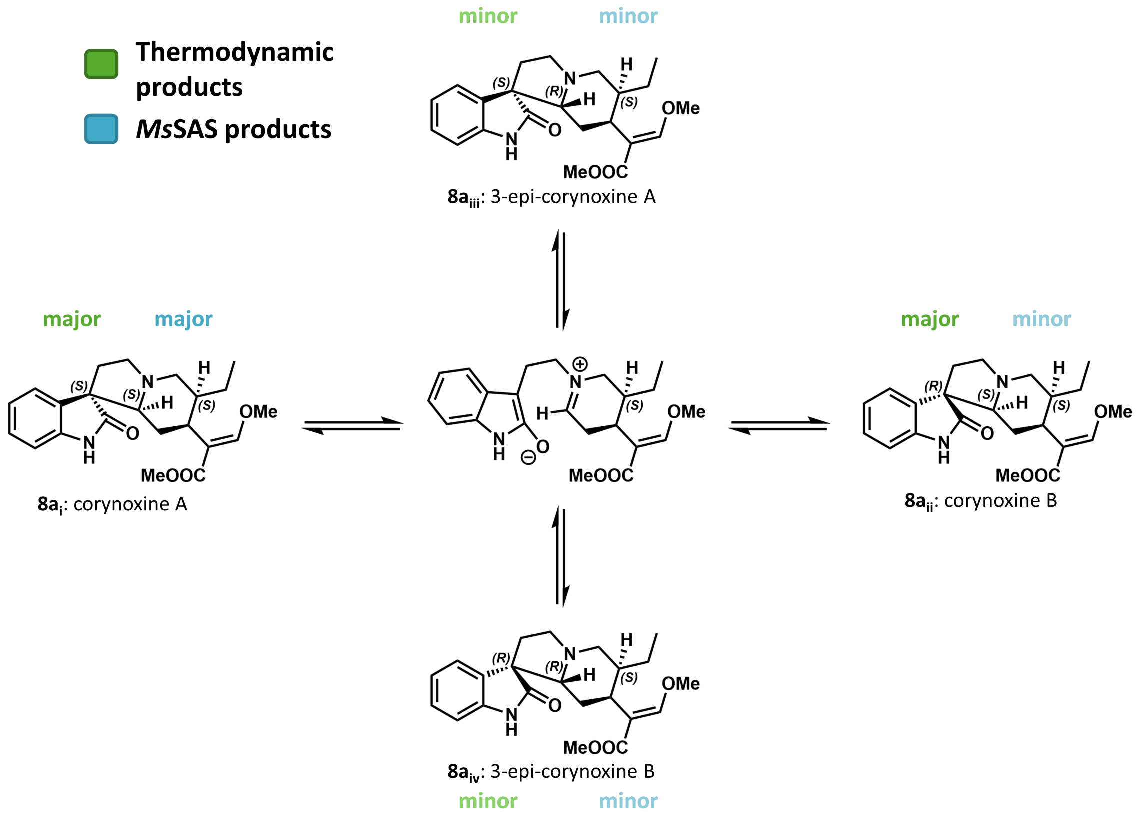

Supplementary Figure 44: Comparison of observed products from *Ms*SAS originating from (20*S*)-isocorynantheidine (**6a**) and proposed thermodynamic distribution of product isomerization (see Laus 1996).[2] *Since standards of **8c**$_{iii}$ and **8c**$_{iv}$ could not be obtained, the precise identity of the remaining major product(s) in the enzymatic reaction could not be conclusively assigned.*

Supplementary Tables

Supplementary Table 1: Peak areas of identified Kratom metabolites from labelled substrate feeding. All values are averages of biological triplicates. *NA = not measured, ND = not detected.*

**METABOLITE AREAS**		**2a**	**2b**	**3a**	**3b**	**6a**	**6b**	**13**	**14**	**5a**	**5b**	**7a**	**7b**	**8**
**Young leaf**	**native**	**2E+05**	**5E+04**	**1E+07**	**1E+06**	**5E+06**	**2E+05**	**4E+07**	**6E+07**	**6E+07**	**1E+07**	**2E+07**	**5E+06**	**1E+08**
	d5-1	7E+05	3E+04	1E+07	1E+06	1E+06	2E+04	8E+04	ND	2E+06	4E+05	2E+06	3E+05	4E+06
	d4-2a	1E+07	9E+03	7E+06	2E+06	2E+05	3E+04	NA	NA	ND	3E+04	3E+05	7E+04	5E+05
	d4-2b	3E+05	1E+07	5E+05	2E+05	ND	3E+04	NA	NA	ND	ND	1E+05	2E+04	5E+04
	d2-9OS	2E+02	2E+04	4E+04	ND	2E+04	3E+04	NA	NA	4E+05	4E+05	1E+05	8E+04	3E+04
**Stem**	**native**	**6E+05**	**7E+04**	**6E+06**	**1E+06**	**6E+07**	**6E+05**	**2E+07**	**5E+07**	**1E+07**	**2E+06**	**2E+08**	**1E+07**	**2E+08**
	d5-1	3E+07	8E+04	2E+07	1E+07	2E+06	2E+06	9E+03	ND	ND	9E+04	2E+06	4E+06	1E+06
	d4-2a	9E+06	2E+04	1E+07	2E+07	2E+06	2E+06	NA	NA	ND	3E+04	2E+06	3E+06	2E+06
	d4-2b	3E+05	3E+07	6E+05	4E+05	2E+05	6E+04	NA	NA	ND	ND	1E+06	1E+05	2E+05
	d2-9OS	9E+03	3E+04	ND	ND	1E+05	4E+04	NA	NA	6E+05	2E+06	1E+06	7E+04	2E+05
**Mature leaf**	**native**	**9E+04**	**5E+04**	**3E+05**	**2E+05**	**4E+05**	**2E+05**	**ND**	**4E+06**	**2E+07**	**2E+06**	**1E+06**	**7E+05**	**3E+07**
	d5-1	1E+06	1E+05	4E+05	2E+05	1E+05	7E+04	ND	ND	ND	6E+03	ND	3E+04	1E+05
	d4-2a	8E+06	2E+04	5E+05	8E+05	2E+04	5E+04	NA	NA	ND	ND	ND	ND	ND
	d4-2b	3E+05	1E+07	3E+04	7E+04	9E+03	5E+04	NA	NA	ND	ND	ND	ND	ND
	d2-9OS	9E+03	2E+03	ND	6E+04	ND	3E+04	NA	NA	ND	9E+04	ND	ND	ND
**Root**	**native**	**1E+05**	**5E+04**	**1E+06**	**9E+05**	**1E+06**	**3E+05**	**ND**	**1E+06**	**3E+05**	**2E+05**	**8E+06**	**3E+06**	**6E+06**
	d5-1	4E+05	1E+04	4E+04	1E+05	2E+04	8E+04	ND	ND	ND	ND	ND	2E+04	ND
	d4-2a	3E+05	ND	3E+05	5E+05	8E+04	3E+04	NA	NA	ND	ND	ND	ND	ND
	d4-2b	2E+04	1E+05	2E+05	4E+04	6E+04	4E+04	NA	NA	ND	ND	ND	ND	2E+05
	d2-9OS	2E+04	8E+03	1E+04	3E+04	6E+04	3E+05	NA	NA	ND	ND	ND	6E+04	ND

Supplementary Table 2: Corresponding standard deviations for above observed metabolite areas from Supplementary Table 1. *NA = not measured, ND = not detected.*

STDEV		2a	2b	3a	3b	6a	6b	13	14	5a	5b	7a	7b	8
**Young leaf**	**native**	**3E+04**	**2E+04**	**3E+06**	**3E+05**	**1E+06**	**2E+04**	3E+05	1E+05	**1E+07**	**2E+06**	**8E+06**	**6E+05**	**1E+07**
	d5-1	3E+05	1E+04	4E+06	7E+05	5E+05	6E+04	1E+06	NA	6E+05	2E+05	1E+06	6E+04	1E+06
	d4-2a	4E+06	8E+03	2E+06	3E+05	1E+05	2E+04	NA	NA	ND	5E+04	1E+05	8E+03	3E+05
	d4-2b	6E+04	6E+06	3E+04	1E+04	ND	4E+04	NA	NA	ND	ND	1E+04	9E+03	9E+04
	d2-9OS	3E+04	2E+04	4E+04	ND	2E+04	4E+04	NA	NA	3E+05	2E+05	7E+04	3E+04	4E+04
**Stem**	**native**	**3E+05**	**2E+04**	**6E+05**	**2E+05**	**5E+06**	**7E+04**	**2E+05**	**1E+05**	**1E+06**	**1E+06**	**9E+06**	**8E+05**	**1E+07**
	d5-1	1E+07	4E+04	7E+06	5E+06	1E+06	6E+05	2E+06	ND	ND	7E+04	3E+05	1E+06	4E+05
	d4-2a	9E+05	9E+03	6E+06	6E+06	2E+06	7E+05	NA	NA	ND	4E+04	4E+05	6E+05	2E+06
	d4-2b	7E+04	1E+06	1E+05	6E+04	9E+04	4E+03	NA	NA	ND	ND	4E+04	2E+04	5E+04
	d2-9OS	2E+04	2E+04	2E+04	4E+04	9E+04	2E+04	NA	NA	7E+05	7E+05	1E+05	3E+04	1E+05
**Mature leaf**	**native**	**9E+03**	**1E+04**	**ND**	**ND**	**4E+04**	**8E+03**	**ND**	**4E+04**	**2E+06**	**2E+05**	**1E+05**	**7E+04**	**3E+06**
	d5-1	5E+05	8E+04	5E+04	1E+05	1E+05	2E+04	ND	ND	ND	7E+04	ND	2E+04	1E+05
	d4-2a	3E+06	2E+04	5E+05	5E+05	3E+04	3E+04	NA	NA	ND	ND	ND	ND	ND
	d4-2b	8E+04	6E+05	6E+04	3E+04	1E+04	6E+04	NA	NA	ND	ND	ND	ND	ND
	d2-9OS	3E+04	2E+04	ND	3E+04	ND	2E+04	NA	NA	ND	2E+04	ND	ND	ND
**Root**	**native**	**1E+04**	**2E+04**	**3E+05**	**3E+05**	**2E+05**	**1E+05**	**ND**	**2E+04**	**7E+04**	**3E+04**	**5E+05**	**2E+05**	**8E+05**
	d5-1	5E+05	2E+04	7E+04	2E+05	4E+04	8E+04	ND	ND	ND	ND	ND	1E+05	ND
	d4-2a	9E+04	ND	2E+05	1E+04	5E+04	5E+04	NA	NA	ND	ND	ND	ND	ND
	d4-2b	3E+04	5E+04	1E+05	5E+04	6E+04	2E+04	NA	NA	ND	ND	ND	ND	2E+05
	d2-9OS	1E+04	5E+03	4E+04	2E+04	3E+04	1E+05	NA	NA	ND	ND	ND	4E+04	ND

Supplementary Table 3: Calculated relative stabilities of spirooxindole alkaloid isomers.

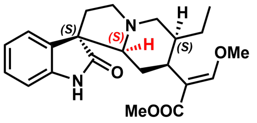

corynoxine A **8a**$_i$

20*S*

Compound	Δkcal/mol	Thermodynamic product	*Ms*SAS products
(3S,7S)-corynoxine A **8a**$_i$	0	major	major
(3S,7R)-corynoxine B **8a**$_{ii}$	1.1	minor	major
(3R,7R)-3-epi-corynoxine B **8a**$_{iv}$	1.6	minor	major
(3R,7S)-3-epi-corynoxine A **8a**$_{iii}$	2.0	minor	*ND*

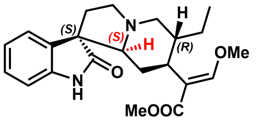

isorhynchophylline **8b**$_i$

20*R*

Compound	Δkcal/mol	Thermodynamic product	*Ms*SAS products
(3S,7S)-isorhynchophylline **8b**$_i$	0	major	major
(3S,7R)-rhynchophylline **8b**$_{ii}$	2.0	major	major
(3R,7R)-3-epi-rhynchophylline **8b**$_{iv}$	3.7	*ND*	*ND*
(3R,7S)-3-epi-isorhynchophylline **8b**$_{iii}$	10.8	*ND*	*ND*

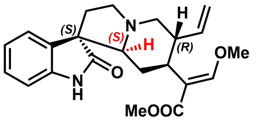

isocorynoxeine **8c**$_i$

20*R*

Compound	Δkcal/mol	Thermodynamic product	*Ms*SAS products
(3S,7S)-isocorynoxeine **8c**$_i$	0	major	major
(3S,7R)-corynoxeine **8c**$_{ii}$	2.2	major	major
(3R,7R)-3-epi-corynoxeine **8c**$_{iv}$	8.9	*ND*	*ND*
(3R,7S)-3-epi-isocorynoxeine **8c**$_{iii}$	10.8	*ND*	*ND*

lowlevel_DFT: B3LYP/6-31G(d), gas phase

Supplementary Table 4: NMR data for **13a**: (20*S*)-3-dehydrocorynantheidine in $CDCl_3$ **overlapped signal J unresolved, measured by 500 MHz NMR*

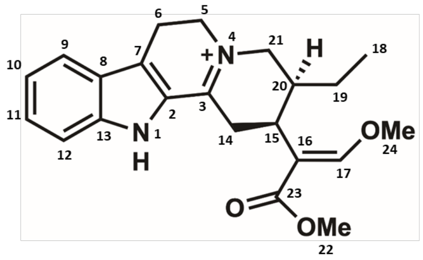

pos.	δ_H	mult.	J_{HH}	δ_C
2	-	-	-	126.7
3	-	-	-	168.5
5a	3.92	m*	-	53.2
5b	3.92	m*	-	53.2
6a	3.28	m*	-	20.2
6b	3.28	m*	-	20.2
7	-	-	-	121.1
8	-	-	-	123.9
9	7.54	d	8.1	120.6
10	7.13	dd	8.1/7.6	121.7
11	7.37	brdd	7.9/7.6	128.5
12	7.80	brd	7.9	115.5
13	-	-	-	142.6
14a	3.88	m*	-	32.8
14b	3.74	dd	16.6/10.7	32.8
15α	3.60	m*	-	28.1
16	-	-	-	108.1
17	7.50	s	-	162.6
18	0.99	t	7.3	11.8
19a	1.49	m	-	23.3
19b	1.17	m	-	23.3
20α	2.10	m	-	38.8
21a	3.58	m*	-	54.7
21b	3.32	m*	-	54.7
22	-	-	-	168.5
23 (CO_2Me)	3.61	s	-	51.7
24(OMe)	3.77	s	-	62.2

Supplementary Table 5: NMR data for **8c$_{ii}$**: corynoxeine in $CDCl_3$
**overlapped signal J unresolved, measured by 700 MHz NMR*

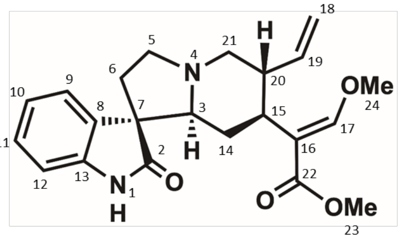

pos.	δ_H	mult.	J_{HH}	δ_C
1 (NH)	8.13	brs	-	-
2	-	-	-	181.3
3α	2.30	dd	11.4/2.4	75.2
5α	2.44	m*	-	55.0
5β	3.39	dd	8.2/8.2	55.0
6α	2.51	m	-	34.9
6β	2.03	ddd	13.3/7.7/1.2	34.9
7	-	-	-	56.1
8	-	-	-	133.8
9	7.21	brd	7.4	123.4
10	7.04	ddd	7.5/7.4/0.9	122.7
11	7.18	ddd	7.7/7.5/1.1	128.0
12	6.87	brd	7.7	109.4
13	-	-	-	141.0
14α	1.23	m	-	29.0
14β	1.99	m	-	29.0
15α	2.43	m*	-	38.7
16	-	-	-	111.7
17	7.23	s	-	159.9
18	4.95	ddd	17.1/2.0/0.6	115.6
18b	4.91	brdd	10.2/2.0	115.6
19	5.51	ddd	17.1/10.2/8.3	139.6
20β	3.01	dddd	11.2/11.0/8.3/4.0	42.2
21α	1.89	dd	11.0/10.9	58.9
21β	3.27	dd	10.9/4.0	58.9
22	-	-	-	168.9
23 (CO_2Me)	3.61	s	-	51.4
24(OMe)	3.72	s	-	61.7

Supplementary Methods

Molecular biology kits

All molecular biology kits were used according to the manufacturer´s instructions, unless specified. The RNeasy Mini Kit (*Qiagen*) was used for RNA extraction (*vide infra*). cDNA was subsequently prepared using iScript reverse transcriptase (*Bio-Rad*), following manufacturer's instructions. For genes or gene fragments destined for downstream applications the SuperFi polymerase was used for amplification. Gene fragments were purified by agarose gel electrophoresis (1% agarose; 120 V, 40 min) and extracted from the gel using a Zymoclean™ Gel DNA Recovery Kit (*Zymo*). All oligonucleotide primers were synthesized by and obtained from *Sigma Aldrich*. Gene cloning was routinely performed using an In-Fusion kit (Clontech *Takara*) or via Gibson assembly. Plasmid DNA was isolated from bacterial cultures using the Wizard® *Plus* SV Minipreps DNA Purification System kit (*Promega*).

Cross-species transcriptome analysis

Three Naucleeae and three non-Naucleeae transcriptomes were used for cross-species transcriptome analysis. These transcriptomes were obtained from the following sources. Publicly available RNA - sequencing data was obtained from the European Nucleotide Archive (https://www.ebi.ac.uk/ena/browser/home). The following run was downloaded: *Coffea arabica* (SRR17345234). The *Catharanthus roseus* transcriptome was obtained from BioProject accession PRJNA847226. The *Cinchona pubescens* transcriptome was obtained from Lombe *et al.* 2024.[3]

The *Uncaria rhynchophylla* transcriptome was obtained from BioProject (https://ngdc.cncb.ac.cn/bioproject). The following runs were downloaded from project PRJNA792441: leaf-1 (SRX13561691) and leaf-2 (SRX13561691). Trinity v2.15.1 was used for the assembly on the Galaxy server, with the parameters "paired-end data" and "--no_normalize_reads" for the assembly.[4]
For the *Uncaria guianensis* transcriptome, libraries were prepared using the KAPA Stranded RNA-Seq Library Preparation Kit (KR0934) with NEBNext Multiplex Oligos for Illumina (E7335). Sequencing was performed on an Illumina HiSeq 2500 to 150 nt in paired end mode by the RTSF Genomics Core at Michigan State University. Raw reads were trimmed using Cutadapt v3.4[5] with the parameters, "--trim-n --quality-cutoff 20,20 --minimum-length 30 --times 3 -a AGATCGGAAGAGCACACGTCTGAACTCCAGTCACNNNNNNATCTCGTATGCCGTCTTCTGCTTG -A AGATCGGAAGAGCGTCGTGTAGGGAAAGAGTGTAGATCTCGGTGGTCGCCGTATCATT". Trimmed reads were used for transcriptome assembly using Trinity v2.3.2 with the parameters "--SS_lib_type RF" and "--no_normalize_reads" for the assembly.[4]

Liquid Chromatograph – Mass Spectrometry (LC-MS) data acquisition

Compound identification

For LC-MS data acquisition an UltiMate 3000 ultrahigh performance liquid chromatography system (UHPLC; *Thermo Fischer*) connected to an Impact II UHR-Q-ToF (Ultra-High Resolution Quadrupole-Time-of-Flight) mass spectrometer (*Bruker*) was used with Bruker Compass Hystar v6.2 software. Compound separation was achieved using reverse-phase liquid chromatography on a Phenomenex Kinetex XB-C18 (100 x 2.1 mm, 2.6 µm; 100 Å) column operated at 40 °C. Mobile phases: (A) water with 0.1 % formic acid; (B) acetonitrile; flow rate = 0.6 ml/min. 2 µL sample was injected in each run; authentic standards were prepared as methanol solutions in concentration ranges between 20-100 µM. Chromatography conditions: 10% B from 0 – 1 min, 10 – 30% B from 1 – 6 min, 30 – 100% B from 6 – 6.1 min, 100% B from 6.1 – 7.5 min, 100 – 10% B from 7.5 – 7.6 min, and 10% B from 7.5 – 10 min. Mass spectrometry conditions: mass spectrometry was performed in positive electrospray ionization mode (capillary voltage = 3500 V; end plate offset = 500 V; nebulizer pressure = 2.5 bar; drying gas: nitrogen at 250 °C and 11 L/min). Mass spectrometry data was recorded at 12 Hz ranging from 80 to 1000 m/z using data dependent MS2 and an active exclusion window of 0.2 min. Tandem mass spectrometry settings: fragmentation was triggered on an absolute threshold of 400 and restricted to a total cycle time range of 0.5 s; collision energy was deployed in a stepping option model (20- 50 eV). To calibrate MS spectrum recording each run was initiated with the direct source infusion of a sodium formate-isopropanol calibration solution (operated by an external syringe pump at 0.18 ml/min using a 5 mL syringe with an ID of 10.3 mm). The initial 1 min of the chromatographic gradient was directed to waste.

Compound purification using preparative HPLC

For preparative HPLC, an Agilent 1260 Infinity II system was used, equipped with a Phenomenex Kinetex XB-C18 column (250 x 10 mm, 5 µm; 100 Å) and coupled to a multiple wavelength detector and fraction collector. Mobile phases A (water + 0.1 % formic acid) and B (acetonitrile) were used. The flow rate was set to 9 mL/min with the following gradient: 10 – 30% B from 0 – 12 min, 30 – 40% B from 12 – 18 min, 40 – 95% B from 18 – 20 min, 95% B from 20 – 24 min, 95 – 10 % B from 24 – 27 min, and 10% B from 27 – 30 min. Samples were prepared in MeOH as concentrated solutions filtered through a 0.22 µm PTFE syringe filter and injected successively (injection volume: 300 µL). All fractions were assessed by UPLC-MS and fractions containing the desired product were concentrated *in vacuo* and dried using lyophilization.

Compound purification using semi-preparative HPLC

For semi-preparative HPLC, an Agilent 1260 Infinity II was used, equipped with a Phenomenex LC column (Luna® 5 µm C18 (2) 100A, 250 x 30 mm, AXIA™ Packed, Ea) and coupled to a multiple

wavelength detector and fraction collector. As mobile phases A (water + 0.1 % formic acid) and B (acetonitrile) were used. The flow rate was set to 9 mL/min with the following gradients:

DHC (13a)/DHM (14a) purification:

20% B from 0 – 6 min, 20 – 30% B from 6 – 14 min, 30 – 100% B from 14 – 16 min, 100% B from 16 – 18 min, 100 – 20 % B from 18 – 19 min, and 20% B from 19 – 22 min.

Rifat Kratom alkaloid purification:

20% B from 0 – 6 min, 20 – 30% B from 6 – 14 min, 30 – 35% B from 14 – 16 min, 35 – 100% B from 16 – 18 min, 100% B from 18 – 20 min, 100 – 20 % B from 20 – 22 min, and 20% B from 22 – 24 min.

Samples were prepared in MeOH as concentrated solutions filtered through a 0.22 μm PTFE syringe filter and injected successively (injection volume: 20 μL). All fractions were assessed by UPLC-MS and fractions containing the desired product were concentrated *in vacuo* and dried using lyophilization.

Purification of *Ms*DCR isoforms and mutants from *Escherichia coli*

The coding sequences for *Ms*DCR1 and *Ms*DCR2 (and mutants of *Ms*DCR1) were cloned into pOPINF and transformed into BL21 (DE3) *E. coli* cells for expression. These strains were grown for 16 h in 2 mL LB + 100 mg/mL carbenicillin (CARB) at 37 ˚C. 1 mL of these saturated cultures was used to inoculate 100 mL LB + CARB, which were grown for a further 3.5 h at 37 ˚C until OD_{600} = 0.6. The cultures were moved to 18 ˚C for 1 h, after which expression was induced via addition of 20 μL 1 M isopropyl ß-D-1-thiogalactopyranoside (IPTG, final concentration = 200 μM). Expression then continued at 18 ˚C for another 16 h.

Cells were harvested via centrifugation, and the supernatant removed. Cell pellets were frozen at –20 ˚C for 1 h. Upon thawing, cells were resuspended in 10 mL 50 mM Tris-HCl buffer pH = 7.5 + 1 mg/mL lysozyme, 0.1 mg/mL DNAse, and 1 mM $MgCl_2$ and incubated with shaking at 37 ˚C for 1 h. The lysed cells were moved to ice for 30 min and then sonicated on ice for 5 min (1 s on; 1 s off). The lysate was then clarified by centrifugation at 35,000 *g* for 15 min. The resulting supernatant was then poured over a 1 mL Ni-NTA column, washed with 20 mL 50 mM Tris-HCl buffer pH = 7.5 buffer containing 20 mM imidazole, and eluted with 2.5 mL of 50 mM Tris-HCl buffer pH = 7.5 buffer containing 250 mM imidazole. The eluted proteins were spin-concentrated/buffer-exchanged using centrifuge filtration with a 10 kDa cutoff (Amicon, *Millipore*) into 50 mM HEPES buffer pH = 7.5. The proteins were then snap-frozen at –70 ˚C. Protein concentration was estimated using the Bradford assay.

Purification of *Rgn*TDC variants and *Pf*TrpB^{2B9} H275E from *Escherichia coli*

The coding sequences for *Rgn*TDC variants (wt, L355A, L355M) and *Pf*TrpB^{2B9} H275E[6] were cloned into pET28b and transformed into BL21 (DE3) *E. coli* cells for expression. This strain was grown for 16

h in 2 mL LB + 100 mg/mL kanamycin (KAN) at 37 ˚C. 1 mL of this saturated culture was used to inoculate 100 mL 2x yeast extract broth (YEB) + KAN, which was grown for a further 3.5 h at 37 ˚C until OD_{600} > 1.0. The culture was moved to 18 ˚C for 1 h, after which expression was induced via addition of 100 µL 1 M isopropyl ß-D-1-thiogalactopyranoside (IPTG, final concentration = 1 mM). Expression then continued at 18 ˚C for another 16 h.

Cells were harvested via centrifugation, and the supernatant removed. Cell pellets were frozen at –20 ˚C for 1 h. Upon thawing, cells were resuspended in 10 mL 50 mM sodium phosphate buffer pH = 8.0 + 1 mg/mL lysozyme, 0.1 mg/mL DNAse, 1 mM $MgCl_2$, and 400 µM PLP and incubated with shaking at 37 ˚C for 1 h. The lysed cells were moved to ice for 30 min and then sonicated on ice for 5 min (1 s on; 1 s off). The lysate was then clarified by centrifugation at 35,000 *g* for 15 min. The resulting supernatant was then poured over a 2 mL Ni-NTA column, washed with 20 mL 50 mM sodium phosphate buffer pH = 8.0 containing 20 mM imidazole, and eluted with 4 mL of 50 mM sodium phosphate buffer pH = 8.0 buffer containing 250 mM imidazole. The eluted protein was spin-concentrated/buffer-exchanged using centrifuge filtration with a 10 kDa cutoff (Amicon, *Millipore*) into 50 mM sodium phosphate buffer pH = 8.0. The protein was then snap-frozen at –70 ˚C. Protein concentration was estimated using a Bradford assay.

Coding Sequences used for this study

***Cr*STR:**
atggcacaccatcaccaccatcacagcagcggtctggaagttctgtttcagggcccgtcgcctattctgaaaaagattttcattgagtctcctagttacgctc
ctaacgcgtttactttcgacagcactgataaagggttctatacaagcgttcaagacggacgtgtgatcaaatatgagggaccgaactctggttttacagattt
cgcttatgcaagtccattctggaacaaggctttctgtgaaaacagtactgacccagaaaaacgccctttgtgtgggcgtacgtacgacatttcatacgattat
aagaattctcagatgtacatcgtcgatggtcactaccatctttgtgtggtaggaaaggagggaggttatgctactcaactggcaacgtctgtccaaggtgta
ccgttcaagtggttatatgcagtcaccgttgatcaacgcactgggatcgtgtactttacggacgtctcgtctatccatgacgactcacctgagggggtcgag
gagattatgaatacgtctgatcgtacgggccgtcttatgaagtacgacccatctactaaggagactacgctgttacttaaagagttacacgttccgggcggg
gctgagatttcggcagatgggagctttgtggtagtagcggagtttctttctaaccgcatcgtcaaatactggttagaaggtcccaaaaaggggagtgcaga
gttcttggtgacgattccgaatcccggtaacattaagcgtaactcagacgggcatttttgggtgtcttcaagcgaggaactggatggaggacagcatggcc
gtgtggttagtcgcgggatcaaattcgacggttttggaaatatcttgcaagtcatccctttgcctcctccttatgaaggcgaacactttgagcagatccaaga
acatgacgggttactgtatattggcagcctgttccattcatcagtcggaatcttagtctacgatgaccatgacaacaaggggaactcttacgtgagcagcta
a

***Ms*STR1:**
atgctatcgaaaatcacacctaacatacacacttctgaaagtatggttgcgttaaccattttctttaccctgttcttgttccctctttcagttgttctatcttctgcgg
aatttttccagttcctcaagtcaccctacggccccaacgccttcgcttttaactccgctggtgaactctacgctgccgtcgaagatggcagaattgtcaagta
caaaggatcaagcaatcacgggttttcgacccacgctgttgcctccccattctggaacagaaaagtttgtgagaattataccgaacttcagctgaaaccttt
tgtgggaggacatatgaccttggatttcaccatgaaactcggcagttgtacattgctgattgctattacggtcttggggtagttggacctgaaggaggccgt
gccactcaagttgccaggagtgcagatggagtggacttcaagtggctctatgccttggccgtggaccaacaaactggctttgtttacctcactgatgttagc
acaaaatatgatgacagaggtgttcaagacatcatgaggataaatgatacaacaggaagattaattaaatatgatccctcaactaatgaagctagagttttga
tgaatgggctgaatgtaccaggtggcaccgaagttagcaaagatggctcatttcttgttgtcgctgaattcttgagccacagaattctcaagtattggttaaag
ggtcctaaggcaaatacttctgaggtattattgaaagtgaggggcccaggaaacataaaaaggaccaaagctggtgaattttgggtggcctctagtgaca
ataatggaattactgttacgcctagagctataaagtttgacgactttggcaacattttacaagtcgtgcctgtccctccaccatataaaggtgaacatttcgaac
aggctcaagagcataatggtgctctttacatcgggacactgttccacgactttgtgggcatattacacaataacgaaggttcatctgaacccaaggaaaata
atgcacatggggtcagtggatctttgaatggggtggcttcttctgtctga

***Cr*SGD:**
atgaacacttctgaaagtatggttgcgttaaccattttctttgctctgttcttgtcccctctttcagttgttttatcttctgcggaatttttccagttcctcaagtcaccc
tacggccccaacgccttcgcttttaactcagctggtgaactctacgctgccgtcgaagacggcagaattgtcaagtacaaaggatcaagcaatcacgggtt
ttccacccacgctgttgcctccccattctggaacagaaaagtttgtgagaattataccgaacttcagctgaaaccgttttgtgggaggacatatgaccttgga
tttcactatgaaactcagcagttgtacattgctgattgctattacggtcttggggtggttggacctgaaggaggccgtgccactcaagttgccaggagtgca
gatggagtggacttcaagtggctctatgccttggccgtggaccagcaaactggctttgtttacctcactgatgttagcataaaatatgatgacagaggtgttc
aagacatcctgaggataaatgatacaacaggcagattaattaaatatgatccgtcaactaatgaagctagagttttgatgaatgggttgaatgtaccaggtg
gtaccgaagttagcaaagatggctcatttcttgttgttgctgaattcttgagccacagaatactcaagtattggttaaagggtcctaaagcaaatacttctgag
gtattattgaaagtgaggggcccaggtaacataaaaaggaccaaagctggtgaattttgggtggcctctagtgacaataatggaattactgttacgcctaga
gctattaagttcgacgactttggcaacattttacaagtcgtgcctgtccctccaccatacaaaggtgaacatttcgaacaagctcaagagcataatggttctct
ttacatcgggacactgttccacgactttgtgggtatattacacaactacgagggttcatctgatcccaaagaaaataatgtagatggggtcgatggatctttg
aatggagtggcttcttctgtctga

***Ms*SGD:**
atggaagctcaaagaactgccactgttgtttccaacgatgcaagcaagatcaaccgcggtgattttgctgaggatttcatttttggagcagcttcatctgctta
tcagacggaaggcggtgcaagtgaaggtggtcgaggtcctagcatatgggacactttcacccagagacgaccaggtatgataaaggagggcggcaat
ggaaatatggctgttgattcatatcatcagtataaggaagatgtcaagattttgaagaacatggggctagatgcctatcggttctcaatatcatggtcgagagt
actgccaggtgggaatttaaatgctggcgtaaataaggaaggaatcaactattacaacaatctcattgatgagctcctagccaatggtatcgagccatatgt
aactctgtttcactgggatgttccccaagcattggaagataaatatggtggctttttaagtcctcaaattgtggacgacttccgcgagtacgtagagctttgctt
ttgggagtttggagatcgagtgaaacactggataacactgaatgaaccatggagctttagtgttggtggatatgtaaacggcacgtttgcaccgggccgag
gtgcctcttcatcagatcaagaaaacgaccatccagctgcactaccgagcagatgttctccatggcaatcacaaagtatttctagcaatggaaatccaggg
acagagccatatgtggtgactcacaatcagcttcttgctcatgcagctgctgtcgaattgtataagagcaactttcagaaatcacaaaatggcaagattggg

attacacttgtgtctcagtggatggaacctttggacgaaaacagtaaagctgatgtcgaagccgcaaagagagctcttgatttcatgcttggatggtttatgg
agcctttaacgaccggtgattatcccaaaactatgagaaaattagttggatctcgtctcccaaaattttcagccgagcaatctaagcaactcaagggatcata
tgattttcttggattaaattattacactgctgactatgtcacaagtgcatcaagctccactactggaggaaatttgagttacactacggattctcaagtgacccat
acaactgatcgaaatggagtgccaattggtccacagggtggctcagaatggttgcatatttatccagaagggattcgcaaactattggtttacgtaaagaag
acatacaatgttccgctcatttacataacagagaatggagttgatgaagtgaatgatacaagcttaacactttctgaagctcgagttgataacaccagaataa
agtatatccaagaccaccttttaaatattcgactagcaatcagtgatggagtaaatgttaagggctacttcgtttggtcattgttggataatttcgagtggagcg
aaggatacactgttcgttttgggtttattcacattgattacacaaacaattttgcaagatacccaaaagactcagccatatggtttatgaattcttttcacaagga
gtaccctaaaaaattcctcaagagaactctggaagatcacgaagatttcgtttcgaagaaaaggttgcgccagtag

***Ms*DCS1:**
atggcaggaaaatgtgcccaagaagagcacacagtgaaggcttttggatgggccgctagagaagcctccggcgctctatctccttacgggttctcaaga
agggcaacaggagagcgtgatgttcgggttaaaattttgtattgtggaatctgtagaacagacgcagaaatgatcagcgacaaattttgctttactaagtatc
ctcatgtgcctgggcatgagatcgtgggtgtggtatctgaagttggtaacaaggtgcaaaaattcaaggttggagctaaagtcggtgtgacaggcataatt
ggatgttgtcgaacttgttatagctgtaccaatggtcttgagagttactgcccaaatgttgcactaacagaagcaggtgaaggtggttgctctaacttcatagt
tttggatgaagactttgtgtttcgttggcctgagaaattacctcttgatcttggagctcctctcctgtgtgctggagccgcttcttacagccctttgaaaaattttg
gacttgataaacctggattgcatattggtatagctggtcttggtggcatgggccatgtagctgtaaaatttgctaaggcttttggggcaaaggtgacagtaatt
agtacatcagataacaaaaaggaggaagccattaaaaaatatggtgcagacgcatttttgaatagtagtaatcctgagcagatgcgggctgcagctggta
cactggctgccatcgttgatactatcccttcgcctcactctctagtgccattgctcgatttattgttgcctcatgggaaggttattgtattaggggcacccagtg
agccatttgtgttgccggttatgcccctgcttcaaggtggaagagtagtcgctgggagttccggtgcaagtttgaagcaaatccaagaaatgctcgattttg
ctgcagaacacaacatagtagctgatgctgaggttatcccaattgactatataaacactgcaataaagcgcattgagaagggcgatatcaaataccgatttg
tcgttgacatcgggaatacactgaaatcggcttaa

***Ms*DCS2:**
atggcagagaagagtcccgaggaagaacatccggttaaagcattcgggctcgcggccaaagatagctcaggcatcttatctccgtttaattttagtcgtcg
cgctacgggcgatcacgacgttcaactgcgtgttctgtactgcggattgtgctactacgacactaagatgattaagaataagagaggcgtgaccagatacc
cgtttgtattcggacacgaaatcgttggagaggttaccgaaataggccgtgaggttcagaaatttaaggtaggtgacaaggttggagtcggttgtatggttg
cttcctgccgtagttgcgagtcttgcgcgaataactgcgagaattattgtccgaacgtatccgtgacggacggtgcgttcttctttaaaacgggtgaggtgtt
gtacggcggctgctccgatattatggtcgcggacgagaacttcgttattcggtggccagagaatttcccacttgacgcaggagcgccattactgtgcgca
ggcattacaacgtattcgccattacgcaactttggcctggacaagccgggcatccacgtgggcatctacggactcggtggtctcggtcatgttgcggttca
gttcgcgaaagccttcggcgcgaaggttacagtcatatcttctagtgaccggaagcggatggaagcgatcgagaagctgggcgctgatagcttcctggt
gaatagcaacttagaagagatgcaggcagctatggggaccatgcacggaattatcgacaccgttccggcgaaccacagtttagtcccgttactggacct
gttaaaaccacagggtaaactgatagtggttggtggtcctgagaagccgttcgagctgccagtattcccgttactgcagggtggccgtctggttgctggaa
gcgccacaggtggcattaaacagacgcaggagatgatagacttcgccgcggaacacaatattctgccccacgtcgaggtggttagcgtcgactacgtca
atacggcaattgaacgaacggaacgtggagacgttaagtaccgttttgtaatcgatattggcaacaccctgtactga

***Cp*DCS:**
atggccggaaaatctcaagaagatgggcagacggtaaaggctctaggatgggccgctagggaagtttctggggcgatctctcctttcgatttctcaagaa
gggccccaggagagcgcgatgtgcaggttaaaatactatattgtggaatctgtagttttgacacagaaatgatcaataacaagtttggctttaccagatatcc
ctttgtactcgggcatgagattgtgggagtggtatctgaagttggtagaaaggtgcaaaaattcaagattggggataaagttggtgtaggaaccatgattgg
atcttgtcgcacttgttatagctgcactcacaatctcgaaaattactgcccaaaagttacattaacagaagcaacttctggtggttgttctaatcttgtgatagca
gatgaagactttgtgttccattggccggtgaatttgcctcttgatcttggagctcctctcctttgtgctgggattactgtttatagccctttgaaaaattttgaactt
gataagcctggattgcgtattggtgtggttggtcttggtggtattggccatatagctgtaaaatttgccaaggcttttggggctaaggtgacagtgattagttca
tcagaaagtaaaaaggttgaagccattgaaaaatatggtgcagattccttttttggttagcagtgatccagggcagatgctggcagctgccggaaccttgga
tggtgtcattgataccgtcccagcacctcactctattttgccattccttgatttactcttgcctcgtggaaagctaattatattaggtgcaccaatggagccatttg
tactgccaatctatcccctgcttcaaggtgggagagtagttgctgggagtgccactggaggattgaaacaaatccaagaaatgcttcattttgcagcagag
cacaacatagtagcagatggcgaggttatcccaatcgacgacattaacactgcgataaagcgcattgagaaaggcgatgtcaaatatcgatttgtggttga
cattggcaataccttaaaatctgcttga

***Ms*EnolMT:**
atgcaaccacagagagggagaaagagagagagagagagagatagagaagagatggaatccgtgcagagcaacagtagttcttctgatcaattcgcaa
tgaaaggtggagatgacgacttcagttacacaaagaattccacctggcagagagatgcaattcaagcaaccaaatttttcattcaagaatctattgctgaga
agcttgacgtcaataaattttgtggaaaggcattttgcgttgctgatttgggatgctcagttggacctaacactttgatagcaatgcagaacattgttgaagctg
tggagcttaaattcaaaaatagaaaaggattccattctcccactatccctgaatttcaagtcttctttaacgatcatacggtgaatgatttcaataccctctttaga
tctctcccaactggtcacgacaagcgctattacggcgttggggttccgggttccttttacggtcgattatttccttgtgactctattcacataatgcacacttcatt
ttctacaccgtttctttctcaagtaccaaaagaggtgattgacaaaaattcagctgcgtggaataaaggaaggattcatcacaattatgctaaagcagatgttt
tgaaggcttatgaagcacaacatgctgaggatatcgactgctttttgacggctagagctaaagaactggtccatggaggattattgatggatgtgacttcatt
ccgcccagatggggtccctcatacccatgtcttgactaacatagggatggaggtattgggttattgcctcatggacttggctggacttatcgatgaagaaaa
cgtggattcctacaacgttccagtttatcttcaatctcctgaagagttgaaacaagctgttcaacggaacaaatacttcagtatagaaaaaatggagagcgtg
cctatgatgatagattcagatgtttctgccaaagctcaacaatattcattgggaatgagggccgtaatgggggacgtgattagagagcaatttggagcgga
gatagtggataaactctttgatttgttcaagaagaaacttgaagagcatcctaactttgcaaaaggagttgtccttgacatgtttgttctccttaaacgcaatgc
agaggattga

***Ms*CO1:**
atgatcacaaaaatctgcaagattctattgctgatttcaattttctacttagtgatcccatcatcacattcatgcttgattcctcatagtttcatccattgcatttcacg
tacgtttccatcaaacgcttctatacttaatgtcctgtatctccctaacaattcttcttatccatatttattaaagtctaccattcacaatcttagattcttgacgccta
ccacccctaccccgcttgcaatagtcactcctttagaatactctcatgtccaagccactgttaaatgtagcaagctgaacggattaaacatcagaatccgaa
gtggtggccatgactatgaaggcatgtcatatacatctgaagttccgtttgtgatgcttgatctcagaaacctaacgtctatcagcattgacattaagactaata
gtgcatgggttgagactggtgcaacgttaggcgaattgtattattcaattgctaagacaagtcctattcatggctttccagcaggcctttgtccaactgttggtg
ttggtggacactttagtggtggcggtgtaggtaacctgatcagaaagtatggactagctgctgataatgtcatcaatgcgcgcattgttgatgttaatggtcg
aattttagatagaaaatcaatgggagctgatctttttgggctattagaggaggtggaggagcaagttttggagttatagttgcctggaaaatcaagcttgtgc
gtgttccacctgtagttactgttttcaatttaaccaagagttcaagtcaagaagccataggtcttattcacaaatggcaatatgcagcgcccaagttgagtgac
gatttgattgtttacattacaatatcatcgataaatgagaagggaggaggacttaccgcaacatttaattcattgttcctcggtagatctggtcagctcttgaaa
atgttggaggagagcttccccgaacttaacctcagaaaagaagattgtgttgagatgagttggattgagtcagtactccattttgcagcatatcaaaatgtgg
aaactatagaggccctaaagaaaagaattaactcgcaacctgataattacttcaaggctaagtcagacttggttcgcaagcctctaccatatgaagcattag
aagagttctggaaatggggttcagatatgaatgctccttacttttatgcagaattgcgtccttatggtggaagaatgaatgagatatcggaatcagaaactcc
atatccacacaggaaaaatgttctctatgaaattctctacgtggtgtcttggacgaaggataaagatgatggatcttccaaaaagaacatcaattggctaaga
ggattatacgagttcatgaccccttacgtgtcaaaagggccaagaggtgctgtttggaattgtagagatcttgatttaggtgctaatggtgcttcagaaacta
cttattctaaagccaaggcatggggatcaaggtatttcaggaacaattttaagaggctggcggttattaaaggtgaagttgatccaaataatttttcaactatg
agcaaagcattccccctcttgttttccatggacaaagtgtggaagagcagatgtgtgagttttcttga

N-His$_6$-*Ms*CO1:
atggcacaccatcaccaccatcacagcagcggtctggaagttctgtttcagatcacaaaaatctgcaagattctattgctgatttcaattttctacttagtgatc
ccatcatcacattcatgcttgattcctcatagtttcatccattgcatttcacgtacgtttccatcaaacgcttctatacttaatgtcctgtatctccctaacaattcttc
ttatccatatttattaaagtctaccattcacaatcttagattcttgacgcctaccacccctaccccgcttgcaatagtcactcctttagaatactctcatgtccaag
ccactgttaaatgtagcaagctgaacggattaaacatcagaatccgaagtggtggccatgactatgaaggcatgtcatatacatctgaagttccgtttgtgat
gcttgatctcagaaacctaacgtctatcagcattgacattaagactaatagtgcatgggttgagactggtgcaacgttaggcgaattgtattattcaattgcta
agacaagtcctattcatggctttccagcaggcctttgtccaactgttggtgttggtggacactttagtggtggcggtgtaggtaacctgatcagaaagtatgg
actagctgctgataatgtcatcaatgcgcgcattgttgatgttaatggtcgaattttagatagaaaatcaatgggagctgatctttttgggctattagaggagg
tggaggagcaagttttggagttatagttgcctggaaaatcaagcttgtgcgtgttccacctgtagttactgttttcaatttaaccaagagttcaagtcaagaag
ccataggtcttattcacaaatggcaatatgcagcgcccaagttgagtgacgatttgattgtttacattacaatatcatcgataaatgagaagggaggaggact
taccgcaacatttaattcattgttcctcggtagatctggtcagctcttgaaaatgttggaggagagcttccccgaacttaacctcagaaaagaagattgtgttg
agatgagttggattgagtcagtactccattttgcagcatatcaaaatgtggaaactatagaggccctaaagaaaagaattaactcgcaacctgataattactt
caaggctaagtcagacttggttcgcaagcctctaccatatgaagcattagaagagttctggaaatggggttcagatatgaatgctccttacttttatgcagaat
tgcgtccttatggtggaagaatgaatgagatatcggaatcagaaactccatatccacacaggaaaaatgttctctatgaaattctctacgtggtgtcttggac
gaaggataaagatgatggatcttccaaaaagaacatcaattggctaagaggattatacgagttcatgaccccttacgtgtcaaaagggccaagaggtgct
gtttggaattgtagagatcttgatttaggtgctaatggtgcttcagaaactacttattctaaagccaaggcatggggatcaaggtatttcaggaacaattttaag
aggctggcggttattaaaggtgaagttgatccaaataatttttcaactatgagcaaagcattccccctcttgttttccatggacaaagtgtggaagagcagat
gtgtgagttttcttag

***Ms*CO2:**
atgctcacaaaaatgtgtaaggtactattgctgatttcaattttctacttagtgatcccatcatcacattcaagcttgattcctcatagtttcatccattgcatttcag
gtacttttccatcaaacacttctatacttgatgtcctgtatctccctaacaattcttcttatccatatttattaaagtctaccattcacaatcttagattcttgacgccta
ccacccctaccccgcttgcaatagtcactcctttagaatactctcatgtccaagccactgttaaatgtagcaagctgaacggattaaacatcagaatccgaa
gtggtggccatgactatgaaggcatgtcatatacatccgaagttccgtttgtgatgcttgatctcagaaacctaacgtctatcagcattgacattaagactaat
agtgcatgggttgagactggtgcaacgttaggcgaattgtattattcaattgccaagacaagtcctattcatggctttccagcaggcctttgtccaactgttgg
tgttggtggacactttagcggtggcggcgtaggtaacctgatcagaaagtatggactagctgctgataatgtcatcaatgcgcgcattgttgatgttaatggt
cgaattttagatagagaaaatcaatgggagctgatctttttgggctattagaggaggtggaggagcaagtttcggagttatagttgcctggaaaatcaagcttg
tgcgtgttccacctgtagttactgttttcaatttaaccaagagttcaagtcaagaagccataggtcttattcacaaatggcaatatgcagcgcacaagctgagt
aacgatttgatggtttacattgcaatatcatccataaatgagaagggaggaggaattgccgcaacatttaattcattgttcctcggtagatctgatcaactcttg
aaaatgatggaggagagcttccctgaacttgacctgagaaaagaagattgtattgagatgagttggatcgagtcagtactccattttgcagcatatcaaaat
gtggaaactatagaggccctaaagaaaagaattaactcgctacctgatagttacttcaaggctaagtcagacttggttcacaagcctataccatatgaagca
ttagaagaattctggaaaaggtgttcagatatgaaagctccttacttttatgcagaattgcgtccttatggtggaagaatgaatgagacatcggaatcagaaa
ctccatatccacacaggaaaaatgtcctctacgaaattctctacatggtgttttggacgaaggataaagatgatggatcttcaaaaaagaacatcaattggct
aagaagattatacgagttcatgactccttatgtatcaaaagggccaagagctgctgttttgaattgcagagatcttgatttaggtgcaaatggtgcttcagaaa
ctacttattctaaagccaagacatggggatcaaggtatttcaggaacaattttaagaggttggcggttattaaaggtgaagttgatccaaataatttttcaact
atgagcaaagcattccacctctggttttccatggacgaagtgtcgaagagcaaatgtga

***Ms*DCR1:**
atggaagcaaaagcaagattttgataattgggggcacagggtacattggaaaattcgtagttgaagccagtgtaaaagaagggcacccaacttttgcatt
ggttagagaaagcgcagtctcagatcctaaaaaggctgcaattgtagaaagcttcaagagctcaggagtcacaattctttatggagatttaaacaatcatca
gcagttggttaatgcaatcaaacaagtggatattgtcatctctgctctgggtggagatttgacagtgatgcctgagcaagtgaagatcattgcggctattaaa
gaagctggaaccatcaaaagatttttacctgctgaattcgagattgatgtggatcgtatgcatgctgttgagcctgctgcaagctatttgaggtcgaaggcga
agattcgcaaaattgttgaggctgaaggaatacctcatacttacttggtatctaacggttttattggttatttgccccatttctttaatctctttgaagtccttagccc
cacaactcttcccagagacaaagttgttgttcttggtgatggaaatccaaaagttgttttcaataaggaagaggacatagctacgtataccatcaaagcagc
agatgacccaaggactctgaacaagagcgtgtacgttagacctcctgccaacactttgtccttcaatgaaataatctcattgtgggaaaagaaaattggcaa
gaccctcgaaaggatttatgttccagaggaagaacatcttaagaaaattcaagaggcttcaatgccattaaacgcgatcctagctatgggatactcggctat
cgtgaagggagatactgcaaactatgagattgtagctacttttggagcggaggcaactgagctttatcctgatgtgaaatataccacaactgacgagttcctt
gaccagtttgtacgagacatatcaagaaat

***Ms*DCR2:**
atggaagcaaaagcaagattttgataattgggggcacagggtacattggaaaattcatagttgaagccagtgtaaaagaagggcacccaacttttgcatt
ggttagagaaagcgcagtctcagatcctaaaaaggctgcaattgtagaaagcttcaagagctcaggcgtcacaattctttatggagatttaaacaatcatca
gcagttggttaatgcaatcaaacaagtggatattgtaatctctgctctgggggggagatttgaccgtggtgcctgagcaagtgaagatcattgcagctattaaa
gaagctggaaccatcaaaagatttttaccttctgaattcgagattgatgtggatcgtatgcatgctgttgagcctgctacaagctatttcaggtcgaaggcca
agattcgcagaattgttgaggctgaaggaatacctcatacttacttggtatctaactgttttatgggttatttgccccattgctttaatctctctgaagaccttagc
cccagacctcctcccagagacaaagttgttgttcttggtgatggaaatccaaaagttgttttcaataaggaagaggacatagctacatataccatcaaggca
gcagatgacccaaggactctgaacaagagcgtgtacgttagacctcctcccaacactttgtccttcaatgaaataatctcattgtgggaaaagaaaattggc
aagaccctcgaaaggatttatgttccagaggaagaacatcttaagaaaattcaagaggcttcaatgccattaaacgtgctcctagctatgggatactcggct
atcgtgaagggagataatgcaaactatgagattgtagctccttttggagcggaggcaactgagctttatcctgatgtgaaatacaccacaactgatgagttc
cttgaccagtttgca

***Ms*SAS (*Ms*3eCIS):**
atggcaagcttcccagcatttcaggaagaagcgaagagatggctctcgaaccattcagtggcagtctctttgcttcttccttcgttcatgatcttcattctatttc
tttccaaatggtggttcctcactaccagtaagaaaaagaaccttccaccttcacctccgagactgccaataattggaaaccttcatcaaattggttcttttgccc
accgctctttcaaatccttggccaaaaagcacggtccgataatgctacttcacgttggcaagcaaagactgttagttgtctcctccgccgacgctgctcgtg
aggctctcaaaacacatgatgctgtcttttgcgatagacctgattcagaggttacacgaagaattttctataaccataagaacatatctctctccacctatggtg
attattggaagctagtgaggagcatcgctgtgaatcaaattctaaatcagaagagggttcagtcgttcagaagtgtaagagaagaagaggtttcattactgg
tggaaagaatcaaagaatcttgctcttcttcctccgtaatatgcatgaatacgttgttgacaacgcttgtaaatgacatagtgtcaaggataaccattgggaag
aggtcctcaggaaaagatggaagcagatttgcagagtttttgtttgcatccgtcgacttattaggttcttttcttgcaggggacttcttcccagggctttggtgg

ctcggttacataactggattcgaagcaaagatcaagaaagtttccaaagatttggatcaatgtttggagaacttaattgaagaggaaataaacaggaacaaa
agaggagatgaccaaggtaaagacaatcagaatttcctccaggttttgcttgaaatccagagaaccgactcatctggccatgctttggatcgagaatccatt
aaggctgtcataatggacatggttgccggtgcatttgactcgtatacacttttggagtgggcaacgtcattgctagtaaaacatccagatgccatgaaaaaat
tgcaaaatgaggtgaaagaagttgctggatccaaatcattcatatcggaggatgatttaagtaaactgcaatacttgaaagcagtaataaaagaaactttcc
gattctgtcccggtgcaattcttgcaaggataccaatcaaggatgtcaaattaatgggctatgatattgcagcaggcactcaactcctcgtcaatacatgggc
aattggaagggatccaacgttgtgggaaaaacctgaggagtttcggccagaaaggttcttaaatagttcaatagatttgaaaggacatcattttgagctgctt
ccatttggcacaggaagaaggtcttgccccggtatgacatttgccttagtcatggatgagctcgtactagcaaatttggtgtgcaactttaattatgcattgcct
ggtggaacaagagtcgaggacttggacatgagtgaagttcctggaatcacgcctcgtaggagaacccctctgctgctcgttccatctctttgttaa

***Ms*10H:**
atggatgtcctcttgtactttctggccctgattttctctctattcttcattcaaaaccttctcagaaaacagaaagaaagactaccaccaagtccaccatccctac
ctattatcggccatctccacctcctaagaaatccgaaacacagagctctccaacatctctccaacaagtatggccccgtagtttaccttcgtttaggaacccg
tcaaacccttctcgtctcctctccatcaatcatagaggaatgcttcacaaagaatgacataattttcgcaaacagacccgattcacttatcagcaaatacttca
gctatcaaaataatgatcttacattcgccccatatggagatcgctggcgtaatctccgccggctggccaccatccatgtgttctcttcagccaatcttaaacg
gttctccgccattaggacggaagaagtttttgatgctgtgcaagaattgttgtccatctctcatattgaatcccacaaagtaaacctgagatctttgttttccaaa
atggtactcaatgtggctatgaagatgctttctggaaaaaaatttcgtggctctcctgaaatgactgaaatgttttcttcagatatgccgatgggcatttgtgatt
atcttccaatactgggatggcttggttttgggggttttgagaagagcttagtgaaatatcaaaagctgattgatgagttcctgcaagatctgcttgaggagag
ccgcaagattggggaaggttcagttgtgcaaaataaaaccattattcagtcattattgtctctgcaagaagctgaccccgagcgctatcatgatgatgttatc
aaaggaattatgatagatgttttcacagctgggacagatacagttgcatacaccatggaatggatcatgtccctcttactgaaccaccctgaggtgttgcag
aaaataaggagcgaaatagattttcatgtcatcccaggatgtttgattcaggattcagatctccctaaactgtcttatctacgttgtgtgatcaatgagacattaa
gactccttgccccccgtgccccttctgctgccccatttcagctccgaagactgcatagtaaatggattcaatgtgcctcgagggacaactttgttagtagatgtt
tggtccgttcatagagacccaaatgtgtgggaagagccattgaagtttaagcccgaaaggttcgaaggaatcagagtggaagatgaaaaaaagttcaagt
ttattccattcggagtggggagaagggcatgtccgggctcaggaatggctatgagattgatgggattggcattgggatccttgattcagtgctttgaatggg
agagggttgggactgagctagtggacttggaagagggagatgggctagttttgccgaaggttgaacctctagaagcactgtgcaagcctcgcccatcca
tggcccatctcatctctcatatt

BBE03:
atggtcatacaattctgtaagattctattgctaacttttattttctacttggtgatcccatcatcagattcaatcttgattcctcatagttttatcgattgcatttcacgtt
cgtttttatcaaacatttctattcttaatgtcctgtatctccccaacaattcttcttatccatttatctttctgtctaccattcacaatcttagattcttgacacataccag
ccccaccccctcttgcaataatcactcctttagattactctcatgtccaagctactgttaaatgtagcaaattgaatggattaaatatcagaatccgaagtggtgg
ccatgactatgaaggcatgtcttatacatctgaagttccatttgtcttgcttgacctcaataacctgaggtctattagcattgacattaagaaaaatagtgcatgg
gttgaatctggtgcaacaataggagaattgtattattcgattgctgaaagaagtccaattcatggctttccagcaggcctttgccccactattggcgtcggtgg
acactttagtgggggcggtgtaggtaacctgatcagaaagtatggactagctgctgataatgtcatcaatgcgtgcattgttgatgttaatggccgaattcta
gatagaaaatcaatgggaactgatctttttgggccataagaggaggtggtggagcaagtttcggagttatagttgcctggaaaatcaggcttgtgcgcgtt
ccacctgtagtaactgttttcgaattaactaagagttcgaatcaagaagccataggtcttattcacaaatggcaatatgtagcgcacaagctgagtgaagattt
gctgtttagaatcacaatatcatcgataaatgggaaggaaggaggaattgtcgcaacatttgattcattattcctcggtaaagctggtcaccttttgaaaatga
tggaggagagcctccccgaaattcacctgagaaaagaagattgtattgagatgagttggattgagtccgtcctccattttgcagcgtatcagaaaggggaa
aatacagatgccctaaagaatagaattagcccgctgcctaatgcttatttcaagggaaagtcagacttggttcacaagcctataccttatgaggcattagaa
gagttctggaaatggtgttcgcataaaaattctcctactcttcacatagaattgcatccttatggtggaagaatgaaagaaatatcggaatcagaaattccata
cccacacaggaaagatgtgctctacgaaatcctctacatggtgttatggatgaaggataaagatggtgaatcttcggaaaagaacatcaattggctaagag
gattatatgagttcatgactccttatgtgtcaaaagggccaagaggtgctatttggaatattagggatcttgatttaggtgcaaatggtgtttctaaaactactta
ctctaaagccaaggcatggggatcaaggtatttcaagaataattttaagaggctggcatttattaaaggtgaagttgatccaaataatttttcaactatgagca
aagcattccacctttggttttacatgcaaaa

BBE04:
atgagaaagctcagtatcattgtcgcttcattgctctcaaccttgcttatagtctcatttgcaacttctgattctgttcatgaggcatttgttcaatgtctagaacaa
cattcccaaaccaatatttcttcagtaatatacacccctaacaactcttcatttccatctgttttgcaagcttacattagaaacctacgattcaatgagtcctcaac
ccgaaaaccgttccttatcctcactgctttggatgtttctcatatacgggcagccgttatctgtgcaaaagcacatggcttgcagatgaaaatccgaagcgga
ggccatgactacgagggcgtttcctacgtctctgaagtccctttcttatcctagacttgttcaatcttcgatcgatcagtgtgaacatagccgaagagactgc
ttgggttcaggttggagcaacccttggtgaagtatactatagaattgctgagaaaagtaatgtccatggcttccctgcaggtgtttgtcccaccgtgggcgtt
ggtgggcattttggtggaggtggatatggtaacatgatgaggaaatatggtctttctgtggataacatcattgatgcacagatcattgatgggaatggtcgcg

tccttgatcgagcatcaatgggcgaagacttgttctgggccattactggaggtgggggttcgagttatggagttgtccttgcgtacaaaatcaacttagttcg
tgtcccacctcaagtaacagtgttccgggtggagaggacttacgaacaaaatgctacataccttgtacgccgttggcaagaaattgctgacaaattggata
atgatatcttcattaggatgatcattgatgtgattaataatactcgtaccgaagggaaaacaataagatctgcatatttcgcattattcctcggagattcggcaa
ggcttctttctctcatgaaccaaagttttcccgaattgggattgcagcaaaaagattgcattgagatgagttgggctgagtcagttgtctattatacaagctttc
cccttggaactcctgttgatgctcttcttagcagagttcctcaggtaatgactcatcttaaaaggaagtccgattatttgaaaaagcctatgccaatagaaggc
atagaattcatcttcaagaaaatgattgaattgcaaactccagtcttgactttcaatccatatgggggaaggatggctgaaattgcatcctcagcaaaacccctt
tccccatagagctgggaacattgcaaagatccagtatgcaacaaactgggatcaaaatggtgtcgagacggcagaacattacataaatttgacccgagct
ttatacaaatatatgactccttttgtctccaagtttccgagggaagcatttctaaactacagagatcttgacttgggaatcaaccataacggcaaggatagtttc
cttgaaggaactgtttatggaattaagtacttcaaggaaaattttaacagattggtaaaagttaagaccgcggttgatcctgataatttcttcagaaatgaacaa
agcatccccgtgtttccatccaagaaa

BBE05:
atggaaaacagctttcagtctccaaattacacgtctcttctacttcttcttcctcttttcagtttgaattttcagccctttgttgctgcctcagattcaatctatgaaaa
ttttgctagttgtctaacaaaaaatgaaatcccaagtgaccaaatcggtaagattctttatagcccatcaaacacttcattcaactctgttttagaagcttatgttc
gaaaccttaggctcaatacttccagtaccagaaaaccatcaataattgtcacacctttggaaatccaacatgttcaagcaacaattttatgcaccaaaggaac
agggctgcaactaaaaatcagaagcggcggtcatgattttgaaggtatctcatatgtttctgatgtcccttttatcattcttgacatgtttaatctgaggtccatc
agtgttaatatcccaagtgaaactgcttgggtacaagccggggcgacactcggggaactttattacagaatttgggaaaagagcaatgtatatggataccc
agctggtgtttgcccaactgtaggcgtcggtgggcatattagtgggggtggttatggtgcaatgttgcgaaaatttggcctcacagttgataatgttcttgatg
cacaaattgttgatgtaaaaggccaggttttggatagaaaagcaatgggggaagatctttttgggctattagaggcggtggtggtgccagttttggtgttgtt
ttggcgtacaaaattaagatagtccaagtgcctcaaacagtaactgtctttcgggttgaaaagactgaggcagagaatgcaacagatactcttgttcagtgg
caaaatgttgcagacaaaattgacaatgatcttttcataagagttcttgtccaaccaatcactgcaaaaagtggcaaaagtaagggtcagaagatcattaggt
taacattcattgcattatttcttggagattcaaataggcttatctctgtcatgaatgctggatttcctaaattggggttgaagaaaacggattgtcaagaaatgag
ttggatagagtccatgctatattgggcaaattttgacaacacgacaaaacctgaagctcttcttagcagacattacgataccaatttcttgaaaagaaaatcag
attatgtccagaccccaattcctaaggatgcactgaattcaatattcgcgaaaatggttcagcttggaaaaacaggttttgttttcaatccttatggaggaaga
atgagtgaaattcctgaaaacgaaacgccatttcctcatcgggctggtatcatttacaagcttcagtattctgtgaattgggatgatgcagacaccaacttag
caaaacaatatattgggcaagcaagggaactctacagtttcatgacccccctacgtatcaaagaatccaaggcaggcttttctcaactatagggatctagata
ttggtataactaataatggaaagaatagttataatgaaggaaaagtttatggactcaagtatttcaagggtaattatgatagattggttaaagtgaagactattg
tagatcctgaaaatttcttcaggaatgagcaaagtatcccacctctgacctccggggcggcctatcgtgggaggaggaag

BBE06:
atgaagcttcaaatttcttttgctcttcttctactaagcatttcttgtgcaatttcttcagctcagaccgataactccacagcttcttttctcagctgcatcttcagttc
caatgacacctcaatttcaagcatcgtttacaccccagaaaattcttctttcttatcaatcttggatttctacatacaaaattcacgattcctaaatccagaaacac
ccaagcctaaagtgattctgacaccagttaacgaatcacaaattcatttagccatatcttgcggtaacagttctggtttacaaatgagagtacgaagtggagg
ccatgattttgctggcagttcctatatttctgtagtcccatttttcatcctcgatatgttcaactttcgttcaatttctgtcgatgctgaaaatcgcaccgcatgggtt
ggagctggtgcaacccttggcgaaacatactacagcatttatgaaaagaacaggtctttgggtttcacagccggctattggcctactgttgctattggtggg
cacatcagcggaggaggttatggtgcattgaccaggcaatatggtctagctgctgacaatgtcattgatgctcgggtgataactgctaatggaacatatctt
gatcgagcctccatgggtgaggatctttttgggctattagaggtggggttggttcaaatttcgtagtcattcttgcgttccaactatctttagttgatgttccgga
aaatgttactgcattttctgtcactaggaccctggaacaaaacgcgattcaacttgtctacaagtggcaacatgtagcccccactttacctataaatcttaccat
ttccctccaatttactagcaacatttcgagtgaaacaggtaatgtaaccataaatgctgcatttatatctgtttaccgtggtggagttgatgaattactttcaataa
tgggagaatatttccctgaattgggtttaacaagagaggattgcagagaaatgctttggatccaatacttcccttttcatattaaccatccaatagataacgttat
agagtttttgaccaacagaactcctcctagcaaaccttatttcacaggcaaagctgattttgtcaaggaccctatcccagtagagggtcttgaaaagatacta
tacaagctttttgatgttcctccacttattggacagatggaatggactgtttttggaggagggataatggatgaaatccctgaatctgaaataccattcccacat
agagggaacctcttcattatgtttgaggtggtttattggtacgaaaacgatacgtctgccgtgattcaaaatcataccaattggctgagggaacttcatgaagt
tattggaaattatgttcctagcaaccccagggccgcatatgctgattatcgagatcttgacttgggggtgaacaacgttgaaggtgaaacaagcattgcaca
agcacgaatttggggtgctccatatttcaaggataactttgacaggttagtccaagtgaaaactgaggttgatcctgaaaactatttcaagaatgaacaaagt
tttccaccccttccatcttatgcctcatct

BBE07:
atgatgaagccaaaaactatgaagcttcaaattgcttttgcttttctacttgccttttcattaattgcatttgcatcaaagcctaatgagaaagcttttcttcaatgc
ctattgaaacattcagatgacaccacaatttcaagcatcatttacacacccaaaaactctactttcttatcagtcttggacttctacatccaaaattcacgctttct
aaatccggaaactcccaagcccaaagtcattctcacccccactcactgaatcacacattcaaactgcaatttactgtggcaagaagcataacatgcaaatga

gagttcgaagtggtggccatgactttgctggtagttcttacattgctgaggtacccttttttcgtacttgacatgttcaactttcgatccatctcagttgatgctaat
agccgcactgcttgggttggagctggtgccacccttggtgaaacatactacagcatttatgaaaagaatagctctcttggcttccctgctggttattggccta
ccgtttgcattggtgggcacatcagtggcggaggctacggtgcattgacaagggaatatggtcttgccgctgatcatgtcattgatgctcgcataattgatg
ccaccggggcaattcttgacagagcatccatgggtgaggatctcttttgggctatcagaggtggaattggagctaacttcgtagttattctttcctaccagctc
actctagttgacgttccagaaaaagttacagcattttctgtcccaagaaccctcgaacaagatgcaattcagctcgttcacaagtggcaacacgtcgccccc
aagttgccacaacagctcacgatttcagtccaatttacgagtgatgtttcagctgaaactggaaagagaactataattgccacatttatctctgtataccgtgg
tggggctgatcaacttctttcaataatgggagcacagttccctgagttgggcttgaccaaagctgattgcaaggaaatgctttggattcaatacttccccttcc
atattggccactcaatcgataacattaaagatttcttgaccagcagggtccctccaagtaagccttatttcacagccaaggctgattttgccaaggaccccat
ctcagtaaagggacttgaaaggatactgaacaatcttttcgatgtcggtccacttcttggacaaatggaatggaccattttcggaggaggtgtgatggataa
aattccggaatcggaaattccattcccacatagaggaagattgctgattatgtttcaggttgtttattggactgcaaatgatacctcatcagtgattcaatcacg
tatcgattggctaagaaagcttcacaaatctatcggaggctatgttcctaagaatccaagggctgcatatgctgattatcgtgaccttgacttgggtgtgaac
aatgttattggggaaacaagcatcgaacaagcaaagaaatggggtgctccatatttcgcgaacaattttgacaggttagtgcaagtgaaaactcaagttgat
cctgacaattacttcaagaatgaacaaagctttccaaccctcagtccttatacgacctcc

RED01:
atggcgaatcaagtccaactcgaactatggagtcatttggatggaaaatttgtgttggtcactggtgcatcttctgggcttggccgagaattctgcctagatct
cgccaaggctggttgtaaaattgttgcagcagctcgtcgagtggatcggctgaagtctctctgtgatgagatcaaccaattagtttcctcttcaagcacttctt
caatggctgatcattccgtccgagctatggctattgagctcgatgttacagcagatgctcgcagtatagaaatgtcggtccagagagcttgggatgcatttg
gacatattgatactttgattaataatgctggtgtaagaggtagaataagttcatctttggatcttcctgtagacgaatggaatgctaccgtaagaacaaacctaa
caggagcctggttggtgtcaaagtacgttggtttgcgcatgcgtgaagggggtagaggaggctctatcattaatatttcttccatttctgctttgaatcgagca
caaatgcgaggtggtattgcctacagttcttcgaaagcaggaatggactccatgacaaggataatggcattggaattgggacagtataaaattcgagtgaa
tacgataaatgctggactttttcgatctccaattactgagggtcttctagcaaaagatgggattgataatgtagtaaacagaacagttccattaagaactttagg
cacgacagacccggctttgacatcacttgtgcggttcttgattcatgactcctcaagctatataacgggcaatcatttcattgctgatgttggatattccctacc
aggatttcccattttctcttctctc

RED02:
atggcaaaagcagttgtagccacaagttcaagatggtctctatctggaaaaacagctcttgttacaggtggaactcgcggaataggccgtgcaattgtgga
ggagctagctcaaattggtgccactgttcacaccttttcaagaactgaagaagagcttaatcaactcttgcaagagtgggcctcaaagggatttaaagtcac
tggttcagtctgtgacgcatcatcaggagaacaaagaaagcaggtcatggaaaaggtttcttgcatcttcgatggcaagctcaacattcttgtaaacaatgtt
ggaacgtacgtagttaagccagcagctgagtttacacctgaagactattctttgcaaatgtctacaaatcttgattcttgttttcatttttctcaacttgcttatcca
ctcctgaaagcatctgggaatggaaatattgtgttcatttcatctattgcaggtctagtaagtgtacaatacgtatcagtttattcagcaacaaaaggtgcaata
aatcaacttacgaagaacttggcttgtgaatgggctaaagataacatccgggtaaattgtgttgcaccttggtatataagaaccccaccagttgaagattgg
ctgagtgttgaggagaacttgaagaaaatagaatcaagaactccaatgaggcgtgttggagaaccagaagaagtttcatccctggtggcttacctttgtttc
cctgctgcttcctatatcactggccagattatagctgttgatggaggaataactgtgaacggaatccagtgggac

RED03:
See MsDCR2 sequence above

RED04:
atgtctctaaaattgaacggcatcgatttgaactccgaggccgtctcaattgaagcaaatgaaaccccgacgtcgtctaatcctggtgtctccggtggagtg
gatgtgtttcctatggatgcggctagggttttagaggaattgaacgattcatccaatactagtgaattgggggaggagggtttgaaagcagcaacgaaggg
tgtaaatggggatggaagtttggaagtagaaatcgaggtaaataatagagtgtctgaatcaaaggagggtgatacacggagcgacaacagagaagttttt
gggcatgaaaatgcaaaaactaatactaaaatggatctggaagtaaaagggattgacaatgctgcggttgaagagaaggtaggggttgatcagaatcaa
gatacgggagtattggaatttccacagaatcacttcagaggggatgagataatgagtctctttgtggatctgaattcatgctataaagatgaaatttgtaatga
aggagctgagggcacagatagttttattgaggttaacttaccgttttctgttgattcagcggaaaacatgggtgcttcttataaagaaccggtgatgaatggtc
aatacaatgaagtggacggaaaggatgatgattctgatgctttgaaacctaaaattcgtgggatagagaatcaaaccgaaaaggaagacgaattttatgtg
tctgatttagtgtggggcaaggtgaggagccatccatggtggcctgggcagataattgagccttcggctgcatctgagaaagcaatgaaatattttaagaa
agatagctatttgatagcttattttggggacaaaacttttgcatggaatgaagcatcaaagataaagccattcagaatgtgtttctctcaaatgaagaatcaaa
gtaatgtcgaggctttctgccatgccgtggatgctgccctgattgaggtttccagaagggtagagtttgggttggcctgccgatgcttgcctgaggtggaaa
gttccaaggtgaagacccaggtggttgtaaatgctggtttccgagaagaaacaactagcatatatggtggagacagtttttcaattgcaagctcttttatacct
gcaaaactagtaaactttttggagacattagcagaatcgccacattctgacgttgactttttggaatttaccatagcaagatcgcagttattggcttttaatagat

ggaagggttattgccagctgcctgtacttcaagagcttaatggtttttgggaaatgatcctgatctccctgtaatatctggggagaaaaaccttgatgaggc
gattgaagatgatttgggttccaaggaagatgatcaaattgcatccagacaaggaatatctccttcacgcagtatttctcgcatgcgtaggcatcaatccatg
agtaatgagagcaagggaaaaaagagaaagcgcatgttggacctgatgtctggaagcagctcaagttttccaaatgtcaagaacgaatatgaaggaaaa
gtgagcagcgagaaggcctctttatctttcagaaataaacgcaatgctgtggattatgtatctggtgaatcaaggaaaacaagaaggaagggaatttccttg
caaagggctgggaatacaaatccactggttaaaaattacattgaagttgaagaaaatatacgcggttttgctgggaaaagaggtggaggggctccccgtc
ttagctctggcagtagaagttcacaacgagaagtagttaaccatggtgaaccgtctgtaaatgcttcttgttctggagctttgaccttgactgctgagaaatct
catagaggaagggcaactcctcgagagtatccttctacgaacgagatattttcaaagctatgtgctattgccgaaagtcctgctgaaggttacagttccttca
tatcagtagctggtttttctgtgatttccggaattcagtctgtctggaaaatcacaatcttgacaaaaaaacagggtcatcagggaaacaaagagtaaaaaa
gtcgtccgagttagaaactgcagaagcatatgaatttgaaggtatggaagactcttattggactgacagaatcatacaaagcaacttgaacgagcaaatac
tttttgaacctgataccccagctgagaaggatgatgttattgctgaacaagaaggtttagacagtacaaatgtggacttggataatcagcatgatatgaatcct
gtagttactgaagtggaaatggatcagaactccaatgaatactctccaactgctctgatcctaaactttaatgatttagaatctgttcctgcaataactaacctc
aacagtatattcaaccgctatggaccattagaggagtcgcagactaaaattttaagcaagagcaagcgcgcaaaagtggtattcaagaggcgttctgatg
ctgaaactgccttcagcagttctggtaaattcagcatttttggaccttcacttgtcagttaccgccttaaatatataccacctcgaaaagcttcttctgcctccaa
aactaaaagaagaaaaaatgcagcatcagcagaagacagtgcaatg

RED05:
atggcggtggtatcatcatccatgagattaagacttggtacgatgttatgggcatgtgtcttgtgtgaagtttgttggttatgctgctgctgctgcgttgcaccat
catcatcatcatcgtcttctttttcttcttcttcttcttcactgccgcctctgtgcccatccttgccttcttaccattccaaaattcctcctttccttttttactcttctttctc
agtgtcatctttcctcttaccatgccttccctcctttagagatgaatggaaactctcttgagatggcactaagttccaaatcaggggacgtgtatactgctgtgc
ttttctatgattcctggtgtccattttcatggaatgcccgttcaatgtttgaagtcttgggttccatgtatcccaaaattgatcatctagcagttgaacgatcttcag
ccatgccaagcttgttttcaaggtatggcatacatagcttaccagccatattaattgtgaatcaaacatctagaactcgatttcatggttctaaagatttggactc
tttcatgaagttttacaggaagactacaggacatgaaccagttcaatacattgctgtggatcagtctggcagtttaggaggtggtgggaagtctatagtggta
ccatgcattgggtcatcttcatttgaaatcttgacaaaagaaccctatttattgttctctatattattcctatgtttaagagtcatagtgtatatattcccaagaatcct
gttttattcaagaactctttgggtatcttttagtccccatctgaatttggagatttttggagagacaaggcaaaacttggggcgaatccttcacatgattgatgtc
cagagagcttggactaaggttagattgtgtaagagcaggaacttccaccgtggtgctaggaatgccagggtgttagcttcatctctggcttcagtctcattg
ggtgaaacatcaacctctagattg

RED06:
See MsDCR1 sequence above

RED07:
atggtaggcacggtgacggaagtgggaagcaaggtaaccaaatgtaaggttggagatacagttggaatgggatgcttgtgtggatcctgccgcacttgt
gagcaatgtaaaaatgatgtagagacatactgtgccgaactgaaaatgacctatctttccatctactatgatggaactcctactcaaggaggatactcaaatg
agatggttgcggatgagcacttcattattaactggccagagaactttcccctcgctgctggtgcccctctactttgcgctggtgccactgtttacagtccaatg
aaatactatggactggataaaccaggaacacacttgggaattgtaggtctaggtggactaggacatgtggcggtgaaatttgcaaaggctttcggtctaaa
agtgacagtcatcagtacctcgcctagcaaaagggatgaagctatcaatcaacttggagctgatgattttctggttagccgtgatccagatgcaatgaagg
cagccgttggaacaatggataacatttttgatgcagtatctgcagttcatcctcttatgccattgtttgatctattaaagcctcatggaaagcttgtcatggtcgg
tgcaccaactaagccttatgaaatagtggtcccgacactagtcatgtcaaggaaaatggtggggtttgctgctgctgcggggttgaaggagactcaggag
atgattaattttgcagctgagcacaatataactgcagatattgagcttgttcctatggactatgtgaacaaagccatagagcgtcttgccaagaatgatgtcag
atatcgattcgtgctcgatattgccaacacattgaaagaggag

RED08:
atggcaacaactcctgttaacaacatcccctctaaaatgaaggcctgggcttatgaccaatatggaaaaccggaggatgtcctgaaactggaatcagacg
tagctgttcctgacgtaaacgatgaccaggttttgatcaaagttgttgccgcggccctgaatccagtcgacccaaaacgcatggctggtctattcaaggcca
ccgattcccctctgcctacggttgcggggtatgatgttgcgggcgtggtagttaaagttggaagcaaagtgaaggaattcaaggtgggggatgaagtgta
tggagacattcatgagcatggtttggagaacccaaaacaatttgggtcactggcggagtatactgcagcggaggagaagctactagctttgaaacccaaa
aacttgagctttgctgaagccgctagtcttcctcttgccgttgaaacagcatatgaaggccttgaacgctctgggttttcggctggcaaatcaattcttgttctg
ggaggtgctggtggagttggatctttgataattcagctagcaaagcacgtctttggtgcatctagagtggcagcaacttctagctctggaaaactggagttc
cttaaaagcttaggtgctgatttggcaattgactacaaaaaggagaattatgaagatttgccagagaaatttgatgtggtatatgataccgtcggtgaaagtg
gtaggggtataaaggccgtaaaagaaggcggaagtgtggtgacaattatctggccagttgttccccctgcttccatattcgtgctgacttcaactggctctat

cttaaacaagctgaaacctttcatagaggaggggaaagtgaagccagtgattgaccctaaaagcccgtttcccttcacacagaccgtcgaagcgttttcct
atcttgaaactagcagggctaccggcaaagttgccatatatccaattccc

***Rgn*TDC-C-His$_6$** (protein sequence)**:**
MSQVIKKKRNTFMIGTEYILNSTQLEEAIKSFVHDFCAEKHEIHDQPVVVEAKEHQEDKIKQIKIP
EKGRPVNEVVSEMMNEVYRYRGDANHPRFFSFVPGPASSVSWLGDIMTSAYNIHAGGSKLAPM
VNCIEQEVLKWLAKQVGFTENPGGVFVSGGSMANITALTAARDNKLTDINLHLGTAYISDQTHS
SVAKGLRIIGITDSRIRRIPTNSHFQMDTTKLEEAIETDKKSGYIPFVVIGTAGTTNTGSIDPLTEISA
LCKKHDMWFHIDGAYGASVLLSPKYKSLLTGTGLADSISWDAHKWLFQTYGCAMVLVKDIRNL
FHSFHVNPEYLKDLENDIDNVNTWDIGMELTRPARGLKLWLTLQVLGSDLIGSAIEHGFQLAVW
AEEALNPKKDWEIVSPAQMAMINFRYAPKDLTKEEQDILNEKISHRILESGYAAIFTTVLNGKTV
LRICAIHPEATQEDMQHTIDLLDQYGREIYTEMKKALEKHHHHHH

***Rgn*TDC L355A-C-His$_6$** (protein sequence)**:**
MSQVIKKKRNTFMIGTEYILNSTQLEEAIKSFVHDFCAEKHEIHDQPVVVEAKEHQEDKIKQIKIP
EKGRPVNEVVSEMMNEVYRYRGDANHPRFFSFVPGPASSVSWLGDIMTSAYNIHAGGSKLAPM
VNCIEQEVLKWLAKQVGFTENPGGVFVSGGSMANITALTAARDNKLTDINLHLGTAYISDQTHS
SVAKGLRIIGITDSRIRRIPTNSHFQMDTTKLEEAIETDKKSGYIPFVVIGTAGTTNTGSIDPLTEISA
LCKKHDMWFHIDGAYGASVLLSPKYKSLLTGTGLADSISWDAHKWLFQTYGCAMVLVKDIRNL
FHSFHVNPEYLKDLENDIDNVNTWDIGME**A**TRPARGLKLWLTLQVLGSDLIGSAIEHGFQLAVW
AEEALNPKKDWEIVSPAQMAMINFRYAPKDLTKEEQDILNEKISHRILESGYAAIFTTVLNGKTV
LRICAIHPEATQEDMQHTIDLLDQYGREIYTEMKKALEKHHHHHH

***Rgn*TDC L355M-C-His$_6$** (protein sequence)**:**
MSQVIKKKRNTFMIGTEYILNSTQLEEAIKSFVHDFCAEKHEIHDQPVVVEAKEHQEDKIKQIKIP
EKGRPVNEVVSEMMNEVYRYRGDANHPRFFSFVPGPASSVSWLGDIMTSAYNIHAGGSKLAPM
VNCIEQEVLKWLAKQVGFTENPGGVFVSGGSMANITALTAARDNKLTDINLHLGTAYISDQTHS
SVAKGLRIIGITDSRIRRIPTNSHFQMDTTKLEEAIETDKKSGYIPFVVIGTAGTTNTGSIDPLTEISA
LCKKHDMWFHIDGAYGASVLLSPKYKSLLTGTGLADSISWDAHKWLFQTYGCAMVLVKDIRNL
FHSFHVNPEYLKDLENDIDNVNTWDIGME**M**TRPARGLKLWLTLQVLGSDLIGSAIEHGFQLAVW
AEEALNPKKDWEIVSPAQMAMINFRYAPKDLTKEEQDILNEKISHRILESGYAAIFTTVLNGKTV
LRICAIHPEATQEDMQHTIDLLDQYGREIYTEMKKALEKHHHHHH

***Pf*TrpB^{2B9} H275E-C-His$_6$** (protein sequence)**:**
MWFGEFGGQYVPETLVGPLKELEKAYKRFKDDEEFNRQLNYYLKTWAGRPTPLYYAKRLTEKI
GGAKVYLKREDLVHGGAHKTNNAIGQALLAKLMGKTRLIAETGAGQHGVATAMAGALLGMK
VDIYMGAEDVERQKMNVFRMKLLGANVIPVNSGSRTLKDAINEALRDWVATFEYTHYLIGSVV
GPHPYPTIVRDFQSVIGREAKAQILEAEGQLPDVIVACVGGGSNAMGIFYPFVNDKKVKLVGVEA
GGKGLESGKHSASLNAGQVGVSEGMLSYFLQDEEGQIKPSHSIAPGLDYPGVGPEHAYLKKIQR
AEYVAVTDEEALKAFHELSRTEGIIPALESAHAVAYAMKLAKEMSRDEIIIVNLSGRGDKDLDIV
LKASGNVLEKHHHHHH

***Cr*DPAS** (protein sequence)**:**
MAGKSAEEEHPIKAYGWAVKDRTTGILSPFKFSRRATGDDDVRIKILYCGICHTDLASIKNEYEFL
SYPLVPGMEIVGIATEVGKDVTKVKVGEKVALSAYLGCCGKCYSCVNELENYCPEVIIGYGTPY
HDGTICYGGLSNETVANQSFVLRFPERLSPAGGAPLLSAGITSFSAMRNSGIDKPGLHVGVVGLG
GLGHLAVKFAKAFGLKVTVISTTPSKKDDAINGLGADGFLLSRDDEQMKAAIGTLDAIIDTLAVV
HPIAPLLDLLRSQGKFLLLGAPSQSLELPPIPLLSGGKSIIGSAAGNVKQTQEMLDFAAEHDITANV
EIIPIEYINTAMERLDKGDVRYRFVVDIENTLTPPSEL

***Cr*THAS1** (protein sequence)**:**
MAMASKSPSEEVYPVKAFGLAAKDSSGLFSPFNFSRRATGEHDVQLKVLYCGTCQYDREMSKN
KFGFTSYPYVLGHEIVGEVTEVGSKVQKFKVGDKVGVASIIETCGKCEMCTNEVENYCPEAGSID
SNYGACSNIAVINENFVIRWPENLPLDSGVPLLCAGITAYSPMKRYGLDKPGKRIGIAGLGGLGH
VALRFAKAFGAKVTVISSSLKKKREAFEKFGADSFLVSSNPEEMQGAAGTLDGIIDTIPGNHSLEP
LLALLKPLGKLIILGAPEMPFEVPAPSLLMGGKVMAASTAGSMKEIQEMIEFAAEHNIVADVEVIS
IDYVNTAMERLDNSDVRYRFVIDIGNTLKSN*

***Ca*IFR** (protein sequence)**:**
MAVKSKILIVGATGYIGKHIVEASAKAGHPTFALVRESTISDPKRAAIIESFKSLGVTSLYGDLYN
HQQLVNAIKQVDIVISTVGGGTEVVAHQVKIIAAIKEAGNIKRFLPSEFGGDADRWHCVEPAASW
YRTKAEIRRAVEAAGIPYTYLVSNGFAGYLNCFLNLFGDFSSATPPKDKIGILGDGNSKVVFSKEE
DIAAYTIKAADDPRTLNKIVHLRPPANTLSCNEIVSLWEKKIGKTLEKIYLPEKEVLEKIQEATMPL
NLFLSVGYTIFVKGEMANFEIEASFGVEASELYPDVKYTTLDEYLNQFVSD

Synthetic Methods

d_5-tryptamine

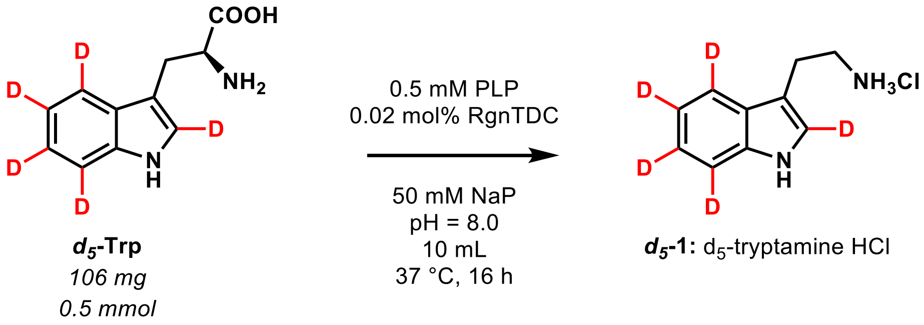

For the synthesis of d_5-tryptamine: to 10 mL of 50 mM sodium phosphate buffer pH = 8.0 was added 106 mg d_5-tryptophan (*Sigma-Aldrich*) (0.51 mmol, [final] = 51 mM), 250 µL 20 mM PLP ([final] = 0.51 mM), and 8.0 µM *Rgn*TDC (0.02 % mol catalyst).[6] The solution was then incubated for 16 h with shaking at 37 °C.

The reaction was quenched via addition of 30 mL ACN, followed by centrifugation to pellet any aggregated protein. The supernatant was removed and the pellet washed with 10 mL ACN, followed again by centrifugation. The combined supernatant fractions were pooled and the ACN evaporated. 0.5 mL 10 M NaOH was used to alkalize the resulting aqueous suspension, which was then extracted three times using 15 mL ethyl acetate (EtOAc). The combined organic layers were then concentrated to 20 mL via evaporation. The EtOAc layer was extracted three times with 15 mL 10 mM HCl in H_2O, the aqueous layers combined, frozen using liquid N_2, and dried using lyophilization. A tannish powder (92 mg) corresponding to d_5-tryptamine HCl was isolated in 90% yield.

tryptamine HCl (1): ^{1}H-NMR (400 MHz, D_2O) δ ppm: 7.60 (*brd, J*= 7.9 Hz, 1H), 7.46 (*brd, J*= 8.1 Hz, 1H), 7.22 (*s*, 1H), 7.20 (*brdd, J*= 7.9, 7.2 Hz, 1H), 7.11 (*brdd, J*= 8.1, 7.2 Hz, 1H), 3.27-3.21 (*m*, 2H), 3.11-3.05 (*m*, 2H). Corresponding spectra are shown in Supplementary Figure 45.

d_5-tryptamine (1- d_5): ^{1}H-NMR (400 MHz, D_2O) δ ppm: 3.20-3.14 (*m*, 2H), 3.05-2.99 (*m*, 2H). Corresponding spectra are shown in Supplementary Figure 46.
m/z $[M+H]^+$ – calculated: 166.1387; observed: 166.1393.

d_4-strictosidine/d$_4$-vincoside

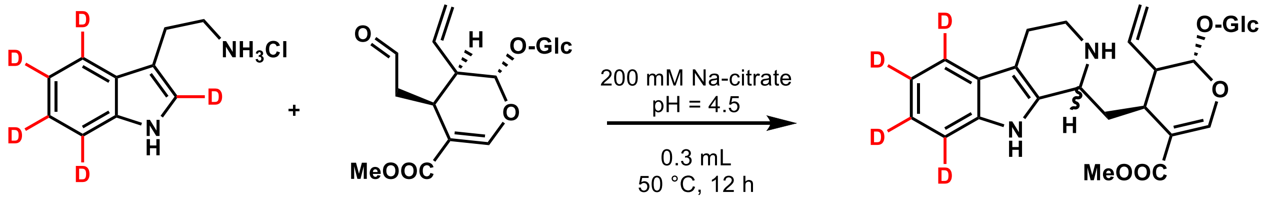

***d*$_5$-1:** d$_5$-tryptamine HCl *9.0 mg* *0.045 mmol*

secologanin *15.8 mg* *0.041 mmol*

***d*$_4$-2a/2b:** d$_4$-strictosidine/vincoside

For the synthesis of d_4-strictosidine/vincoside: to a 1.5 mL microcentrifuge tube was added 9.0 mg d_5-tryptamine HCl (0.045 mmol, [final] = 150 mM) and 15.8 mg secologanin (0.041 mmol, [final] = 136 mM). 0.3 mL 200 mM sodium citrate buffer pH = 4.5 was then added, and the reaction heated to 50 °C with shaking for 12 h. Full conversion of secologanin was observed. The mixture was injected onto preparative HPLC for purification. Fractions corresponding to d$_4$-strictosidine and d$_4$-vincoside were collected and pooled. These pooled fractions were evaporated to remove ACN, frozen, and lyophilized to remove solvent. 5.1 mg d_4-strictosidine (white powder, 23% yield), and 3.6 mg d_4-vincoside (white powder, 17% yield) were obtained.

d_4-strictosidine (d_4-2a): ^{1}H-NMR (500 MHz, MeOH-d_3) δ ppm: 7.78 (*s*, 1H), 5.85 (*ddd, J*= 17.4, 10.6, 7.7 Hz, 1H), 5.84 (*d, J*= 9.0 Hz, 1H), 5.34 (*ddd, J*= 17.4, 1.2, 1.2 Hz, 1H), 5.26 (*brd, J*= 10.6, 1.2, 1.2 Hz, 1H), 4.79 (*d, J*= 8.0 Hz, 1H), 4.53 (*brd*, *J*= 11.1, 1H), 3.97 (*dd, J*= 11.9, 1.8 Hz, 1H), 3.78 (*s*, 3H), 3.69-3.61 (*m*, 2H), 3.43-3.33 (*m*, 3H), 3.26-3.19 (*m*, 2H), 3.10-3.02 (*m*, 2H), 2.99 (*ddd, J*= 16.2, 4.9, 4.5 Hz, 1H), 2.73 (*m*, 1H), 2.28 (*ddd, J*= 14.8, 11.9, 2.9 Hz, 1H), 2.18 (*ddd, J*= 14.8, 11.4, 3.7 Hz, 1H). ^{13}C-NMR (126 MHz, $CDCl_3$) δ ppm: 171.0, 156.6, 138.2, 135.4, 131.0, 127.4, 122.7, 119.9, 119.6, 118.5, 111.8, 109.1, 107.2, 100.3, 97.2, 78.8, 78.1, 74.7, 71.8, 63.0, 52.9, 52.4, 45.4, 42.8, 35.0, 32.5, 19.9. The chemical shifts were in agreement with published data of strictosidine (Misa *et al*. 2022).[7] Corresponding spectra are shown in Supplementary Figure 47.
m/z $[M+H]^+$ – calculated: 535.2588; observed: 535.2611.

vincoside (2b): ^{1}H-NMR (400 MHz, MeOD) δ ppm: 7.52 (*s*, 1H), 7.44 (*brd, J*= 7.8 Hz, 1H), 7.34 (*brd, J*= 8.1 Hz, 1H), 7.12 (*brdd, J*= 8.1, 7.5 Hz, 1H), 7.03 (*brdd, J*= 7.8, 7.5 Hz, 1H), 5.98 (*ddd, J*= 17.4, 10.6, 9.0 Hz, 1H), 5.67 (*d, J*= 7.0 Hz, 1H), 5.40 (*brd, J*= 17.4 Hz, 1H), 5.35 (*brd, J*= 10.6 Hz, 1H), 4.75 (*d, J*= 7.9 Hz, 1H), 4.60 (*brs*, 1H), 3.98 (*dd, J*= 11.9, 2.0 Hz, 1H), 3.68 (*dd, J*= 11.9, 6.6 Hz, 1H), 3.65 (*m*, 1H), 3.64 (*s*, 3H), 3.43-3.34 (*m*, 3H), 3.29-3.20 (*m*, 2H), 3.12 (*m*, 1H), 3.07-2.91 (*m*, 2H), 2.81 (*m*, 1H), 2.40

(*m*, 1H), 2.14 (*m*, 1H). Corresponding spectra are shown in Supplementary Figure 48.
m/z $[M+H]^+$ – calculated: 531.2337; observed: 531.2351.

***d*$_4$-vincoside (*d*$_4$-2b):** ^{1}H-NMR (400 MHz, MeOD) δ ppm: 7.53 (*s*, 1H), 5.98 (*ddd, J*= 17.4, 10.5, 8.7 Hz, 1H), 5.67 (*d, J*= 7.1 Hz, 1H), 5.41 (*brd, J*= 17.4 Hz, 1H), 5.36 (*brd, J*= 10.5 Hz, 1H), 4.76 (*d, J*= 7.9 Hz, 1H), 4.65 (*brs*, 1H), 3.99 (*dd, J*= 11.8, 2.1 Hz, 1H), 3.68 (*dd, J*= 11.8, 6.6 Hz, 1H), 3.65 (*m*, 1H), 3.64 (*s*, 3H), 3.43-3.34 (*m*, 3H), 3.29-3.20 (*m*, 2H), 3.12 (*m*, 1H), 3.07-2.92 (*m*, 2H), 2.81 (*m*, 1H), 2.43 (*m*, 1H), 2.14 (*m*, 1H). Corresponding spectra are shown in Supplementary Figure 49.
m/z $[M+H]^+$ – calculated: 535.2588; observed: 535.2600.

4-methoxytryptamine

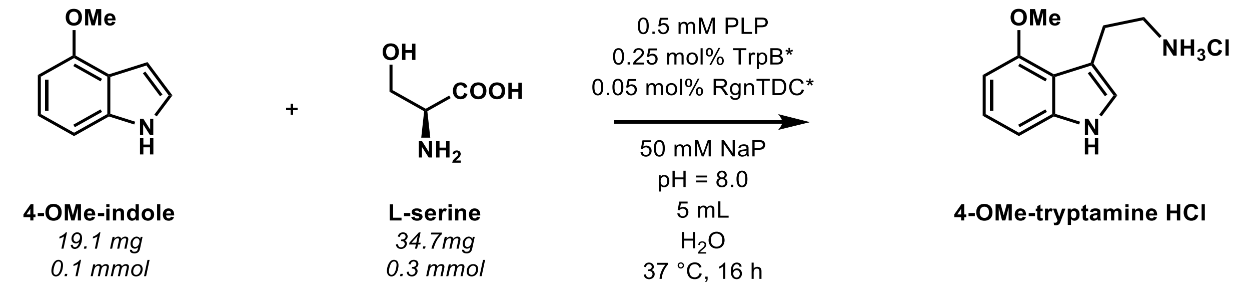

For the synthesis of 4-methoxytryptamine: to 5 mL of 50 mM sodium phosphate buffer pH = 8.0 was added 19.1 mg 4-methoxyindole (0.13 mmol, [final] = 26 mM), 34.7 mg serine (0.33 mmol, [final] = 66 mM), 163 µL 20 mM PLP ([final] = 0.65 mM), 65 µM *Pf*TrpB^{2B9} H275E (0.25 % mol catalyst), and 13 µM *Rgn*TDC L355M (0.05 % mol catalyst).[9] The mixed solution was incubated for 16 h with shaking at 37 °C.

The reaction was quenched via addition of 5 mL ACN, followed by centrifugation to pellet aggregated protein. The supernatant was removed and the pellet washed with 10 mL of 10 mM HCl in MeOH, followed again by centrifugation. The combined supernatant fractions were pooled and the ACN/MeOH evaporated. 0.5 mL 10 M NaOH was used to alkalize the resulting aqueous suspension, which was then extracted three times using 15 mL EtOAc. The combined organic layers were then concentrated to 10 mL via evaporation. The EtOAc layer was extracted three times with 5 mL 10 mM HCl in H_2O, the aqueous layers combined, frozen using liquid N_2, and dried using lyophilization. 10.7 mg of an off-white powder corresponding to 4-methoxy-tryptamine HCl was isolated in a 36% yield. ^{1}H-NMR analysis matched previous reports of 4-methoxytryptamine.[9]

4-methoxytryptamine HCl: ^{1}H-NMR (400 MHz, D_2O) δ ppm: 7.11-7.00 (*m*, 3H), 6.56 (*brd, J*= 7.5 Hz, 1H), 3.84 (*s*, 3H), 3.23-3.18 (*m*, 2H), 3.12-3.06 (*m*, 2H). Corresponding spectra are shown in Supplementary Figure 50.

m/z $[M+H]^+$ – calculated: 191.1179; observed: 191.1172.

d$_2$-4-methoxytryptamine

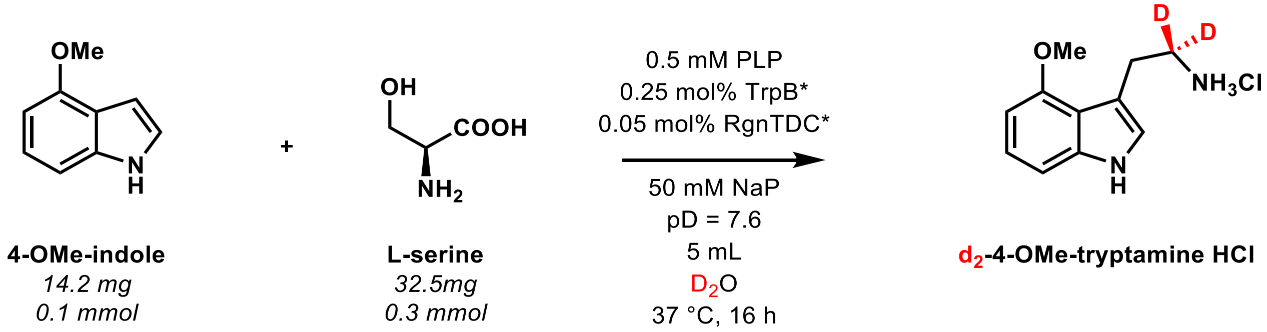

For the synthesis of d$_2$-4-methoxytryptamine: to 5 mL of 50 mM sodium phosphate buffer pD = 7.6 was added 14.2 mg 4-methoxyindole (0.10 mmol, [final] = 19 mM), 32.5 mg serine (0.31 mmol, [final] = 62 mM), 120 µL 20 mM PLP ([final] = 0.48 mM), 48 µM *Pf*TrpB^{2B9} H275E (0.25 % mol catalyst),[6] and 10 µM *Rgn*TDC L355A (0.05 % mol catalyst).[6] All solutions were prepared in D_2O, and enzyme solutions were buffer-exchanged into 50 mM sodium phosphate buffer pD = 7.6. The solution was then incubated for 16 h with shaking at 37 °C.

The reaction was quenched via addition of 5 mL ACN, followed by centrifugation to pellet aggregated protein. The supernatant was removed and the pellet washed with 10 mL of 10 mM HCl in MeOH, followed again by centrifugation. The combined supernatant fractions were pooled and the ACN/MeOH evaporated. 0.5 mL 10 M NaOH was used to alkalize the resulting aqueous suspension, which was then extracted three times using 15 mL EtOAc. The combined organic layers were then concentrated to 10 mL via evaporation. The EtOAc layer was extracted three times with 5 mL 10 mM HCl in H_2O, the aqueous layers combined, frozen using liquid N_2, and dried using lyophilization. 13.4 mg of an off-white powder corresponding to d$_2$-4-methoxy-tryptamine HCl was isolated in a 61% yield. ^{1}H-NMR analysis matched previous reports of 4-methoxytryptamine[9] and revealed a deuterium labeling ratio of 7:3 for d$_2$:d$_1$.

***d*$_2$-4-methoxytryptamine HCl:** ^{1}H-NMR (400 MHz, D_2O) δ ppm: 7.11-7.00 (*m*, 3H), 6.56 (*brd, J*= 7.5 Hz, 1H), 3.84 (*s*, 3H), 3.21-3.17 (*m*, 0.3H), 3.11-3.06 (*m*, 2H). Corresponding spectra are shown in Supplementary Figure 50.

m/z $[M+H]^+$ – calculated: 193.1304; observed: 193.1296.

9-methoxystrictosidine

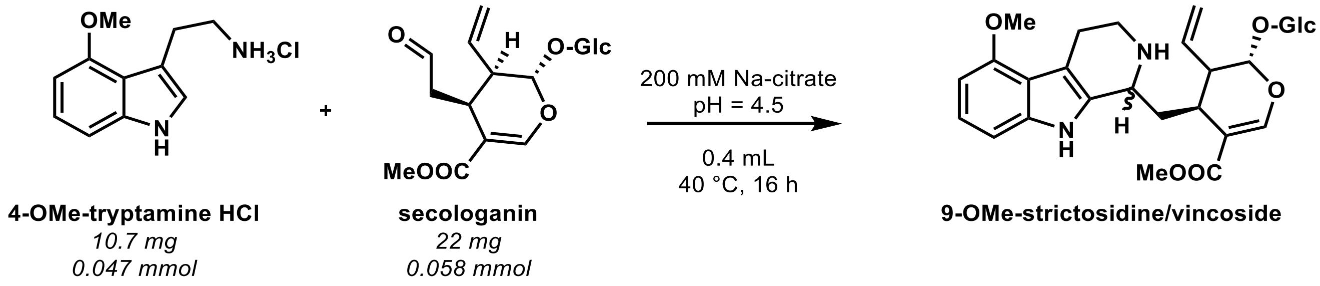

For the synthesis of 9-methoxystrictosidine: to a 1.5 mL microcentrifuge tube was added 10.7 mg 4-methoxy-tryptamine HCl (0.047 mmol, [final] = 120 mM) and 22 mg secologanin (0.058 mmol, [final] = 140 mM). 0.4 mL 200 mM sodium citrate buffer pH = 4.5 was then added, and the reaction heated to 40 °C with shaking for 16 h. Full consumption of tryptamine was was observed, and so the mixture was injected onto preparative HPLC for purification. Fractions corresponding to 9-methoxystrictosidine and 9-methoxyvincoside were collected and pooled. These pooled fractions were evaporated to remove ACN, frozen, and lyophilized to remove solvent. 3.0 mg 9-methoxystrictosidine (**9OS**, white powder, 21% yield), and 2.9 mg 9-methoxyvincoside (white powder, 24% yield).

9-methoxystrictosidine (9OS): ^{1}H-NMR (500 MHz, MeOH-d_3) δ ppm: 7.76 (*s*, 1H), 6.98 (*dd*, *J*= 8.1, 7.8 Hz, 1H), 6.88 (*d*, *J*= 8.1 Hz, 1H), 6.46 (*d*, *J*= 7.8 Hz, 1H), 5.84 (*ddd*, *J*= 17.2, 10.7, 7.7 Hz, 1H), 5.82 (*d*, *J*= 8.9 Hz, 1H), 5.33 (*ddd*, *J*= 17.2, 1.2, 1.2 Hz, 1H), 5.25 (*ddd*, *J*= 10.7, 1.2, 1.2 Hz, 1H), 4.78 (*d*, *J*= 7.9 Hz, 1H), 4.42 (*brd*, *J*= 11.6 Hz, 1H), 3.97 (*dd*, *J*= 11.8, 1.4 Hz, 1H), 3.85 (*s*, 3H), 3.78 (*s*, 3H), 3.64 (*dd*, *J*= 11.8, 6.8 Hz, 1H), 3.55 (*ddd*, *J*= 12.0, 5.0, 5.0 Hz, 1H), 3.40 (*dd*, *J*= 9.0, 8.7 Hz, 1H), 3.35 (*m*, 1H), 3.26 (*m*, 1H), 3.23 (*dd*, *J*= 9.2, 9.0 Hz, 1H), 3.22 (*dd*, *J*= 8.7, 7.9 Hz, 1H), 3.19 (*m*, 2H), 3.05 (*ddd*, *J*= 11.8, 4.5, 3.8 Hz, 1H), 2.72 (*m*, 1H), 2.22 (*ddd*, *J*= 14.5, 11.8, 2.7 Hz, 1H), 2.13 (*ddd*, *J*= 14.5, 11.6, 3.8 Hz, 1H). ^{13}C-NMR (126 MHz, MeOH-d_3) δ ppm: 170.8, 156.3, 155.6, 139.5, 135.5, 129.9, 123.8, 119.5, 117.7, 109.3, 107.3, 105.5, 100.3, 100.3, 97.3, 78.7, 78.1, 74.7, 71.8, 63.0, 55.4, 52.6, 52.3, 45.4, 43.0, 35.4, 32.5, 22.3. Corresponding spectra are shown in Supplementary Figure 51.
m/z $[M+H]^+$ – calculated: 561.2443; observed: 561.2427.

***d_2*-9-methoxystrictosidine**

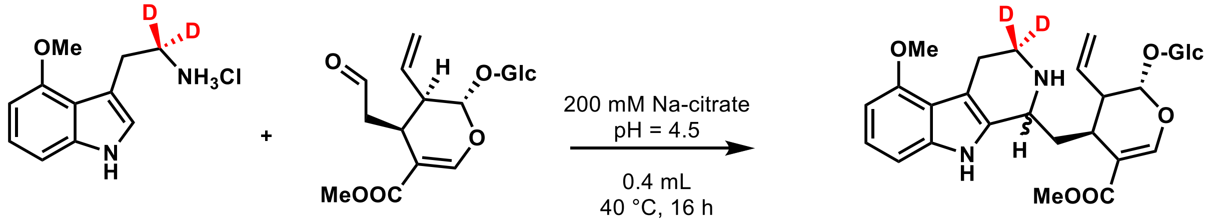

d_2-4-OMe-tryptamine HCl *8.3 mg* *0.036 mmol* — **secologanin** *22 mg* *0.057 mmol* — **d_2-9-OMe-strictosidine/vincoside**

For the synthesis of *d_2*-9-methoxystrictosidine: to a 1.5 mL microcentrifuge tube was added 8.3 mg *d_2*-4-methoxytryptamine HCl (0.036 mmol, [final] = 91 mM) and 22 mg secologanin (0.057 mmol, [final] = 140 mM). 0.4 mL 200 mM sodium citrate buffer pH = 4.5 was then added, and the reaction heated to 40 °C with shaking for 16 h. Full tryptamine conversion was observed, and so the mixture was injected onto preparative HPLC for purification. Fractions corresponding to unreacted secologanin, d_2-9-methoxystrictosidine, and d_2-9-methoxyvincoside were collected and pooled. These pooled fractions were evaporated to remove ACN, frozen, and lyophilized to remove solvent. 7.4 mg of secologanin was recovered (34% of original), along with 4.4 mg *d_2*-9-methoxystrictosidine (***d_2*-9OS**, white powder, 14% yield), and 4.9 mg *d_2*-9-methoxyvincoside (white powder, 13% yield).

***d_2*-9-methoxystrictosidine (*d_2*-9OS)**: ^{1}H-NMR (500 MHz, MeOH-*d_3*) δ ppm: 7.75 (*s*, 1H), 6.98 (*dd*, *J*= 8.1, 7.8 Hz, 1H), 6.87 (*d*, *J*= 8.1 Hz, 1H), 6.45 (*d*, *J*= 7.8 Hz, 1H), 5.84 (*ddd*, *J*= 17.3, 10.7, 7.7 Hz, 1H), 5.82 (*d*, *J*= 8.8 Hz, 1H), 5.33 (*ddd*, *J*= 17.3, 1.2, 1.2 Hz, 1H), 5.25 (*ddd*, *J*= 10.7, 1.2, 1.2 Hz, 1H), 4.78 (*d*, *J*= 7.9 Hz, 1H), 4.41 (*brd*, *J*= 11.2 Hz, 1H), 3.97 (*dd*, *J*= 11.8, 1.8 Hz, 1H), 3.85 (*s*, 3H), 3.78 (*s*, 3H), 3.64 (*dd*, *J*= 11.8, 6.9 Hz, 1H), 3.52 (*dd*, *J*= 5.0, 5.0 Hz, 0.3H), 3.39 (*dd*, *J*= 9.0, 8.7 Hz, 1H), 3.35 (*m*, 1H), 3.23 (*m*, 1H), 3.22 (*m*, 1H), 3.17 (*m*, 2H), 3.05 (*ddd*, *J*= 11.5, 4.3, 3.9 Hz, 1H), 2.71 (*ddd*, *J*= 8.8, 7.7, 4.3 Hz, 1H), 2.21 (*ddd*, *J*= 14.7, 11.5, 2.7 Hz, 1H), 2.13 (*ddd*, *J*= 14.7, 11.2, 3.9 Hz, 1H). ^{13}C-NMR (126 MHz, MeOH-*d_3*) δ ppm: 170.8, 156.3, 155.6, 139.5, 135.5, 129.9, 123.8, 119.5, 117.7, 109.4, 107.3, 105.5, 100.3, 100.3, 97.3, 78.7, 78.1, 74.7, 71.8, 63.0, 55.4, 52.6, 52.3, 45.4, 42.8, 35.4, 32.5, 22.1. Corresponding spectra are shown in Supplementary Figures 52-53.

m/z $[M+H]^+$ – calculated: 563.2568; observed: 563.2558.

Synthesis of 3-dehydromitragynine trifluoroacetate (14a).

3-Dehydromitragynine trifluoroacetate (**14a**) was prepared over two steps from mitragynine (**5a**) according to methods described previously.[8,9]

7-hydroxymitragynine (24a)

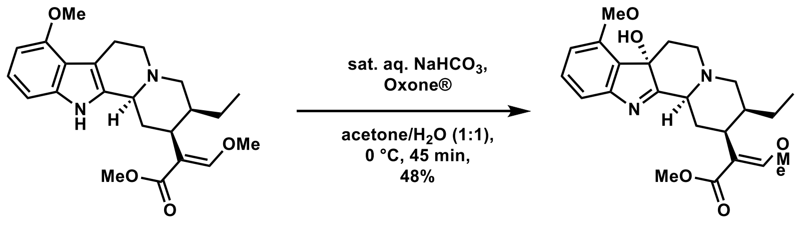

5a: mitragynine
4 mg
0.010 mmol

24a: 7-hydroxymitragynine

To a 1.5 mL vial containing (mitragynine (**5a**, 4 mg, 10 μmol) dissolved in anhydrous acetone (0.33 mL) was added sat. aq. $NaHCO_3$ (0.2 mL). The resulting amber suspension was cooled to 0 °C and then treated dropwise with a solution of Oxone® (6.16 mg, 10 μmol) in H_2O (0.1 mL). After stirring at 0 °C for 45 min, the mixture was diluted with H_2O (0.5 mL) and extracted with EtOAc (0.25 mL x 5). The combined organic layers were dried over anhydrous Na_2SO_4 and concentrated under a gentle stream of Ar to give an amber residue that was further purified by PTLC (2% [v/v] NEt_3 in EtOAc/hexane [2:3]) to afford title compound **24a** as a pale amber residue (2 mg, 48%): R_f 0.17 (2% [v/v] NEt_3 in EtOAc/hexane [2:3]).

7-hydroxymitragynine (24a): ^{1}H NMR (500 MHz, $CDCl_3$) δ 7.44 (s, 1H), 7.30 (t, J = 8.0 Hz, 1H), 7.21 (d, J = 7.6 Hz, 1H), 6.74 (d, J = 8.3 Hz, 1H), 3.87 (s, 3H), 3.81 (s, 3H), 3.70 (s, 3H), 3.13–3.09 (m, 1H), 3.05–3.00 (m, 2H), 2.82–2.77 (m, 2H), 2.64–2.62 (m, 2H), 2.48 (dd, J = 11.3, 2.3 Hz, 1H), 2.16 (s, 1H), 1.89 (d, J = 13.6 Hz, 1H), 1.74–1.65 (m, 2H), 1.42 (t, J = 7.3 Hz, 2H), 0.82 (t, J = 7.3 Hz, 3H). Spectral data agreed with those reported previously.[10] Detailed assignment shown in Supplementary Figure 63.

3-dehydromitragynine trifluoroacetate (14a)

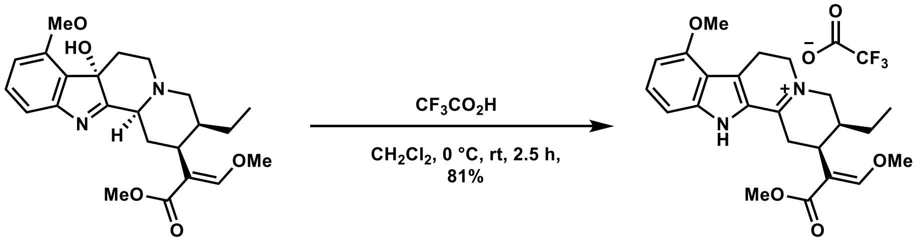

24a: 7-hydroxymitragynine
2 mg
0.005 mmol

14a: DHM-TFA

To a 1.5 mL vial containing 7-hydroxymitragynine (**24a**, 2 mg, 4.8 μmol) was added anhydrous CH_2Cl_2 (0.5 mL). Upon cooling to 0 °C, the solution was charged with trifluoroacetic acid (2.2 μL, 0.28 μmol) and allowed to stir for 2.5 h whilst slowly warming to rt. The reaction mass was concentrated under a gentle stream of argon to afford title compound **14a** as a bright yellow residue (2 mg, 81%).

3-dehydromitragynine trifluoroacetate (TFA) (14a) from synthesis: ^{1}H NMR (400 MHz, $CDCl_3$)* *δ* 11.61 (s, 1H), 7.52 (s, 1H), 7.30 (t, *J* = 8.1 Hz, 1H), 7.12 (d, *J* = 8.4 Hz, 1H), 6.43 (d, *J* = 7.7 Hz, 1H), 3.91 (s, 3H), 3.90–3.84 (m, 2H), 3.78 (s, 3H), 3.62 (s, 3H), 3.60–3.55 (m, 3H), 3.54–3.42 (m, 3H), 3.27 (t, *J* = 12.6 Hz, 1H), 2.15–2.12 (appbrs, 1H), 1.55–1.44 (m, 1H), 1.20–1.13 (m, 1H), 0.99 (t, *J* = 7.4 Hz, 3H). Spectral data agreed with those reported previously.[9] *Note: The presence of residual methylene chloride[11] and triethylammonium trifluoroacetate[12] was confirmed upon comparison of ^{1}H NMR obtained for **14a** with previously reported spectral data. Detailed assignment shown in Supplementary Figure 64. *m/z* $[M]^+$ – calculated: 397.2122; observed: 397.2120.

<u>(20*S*)-10-hydroxycorynantheidine (16a)</u>

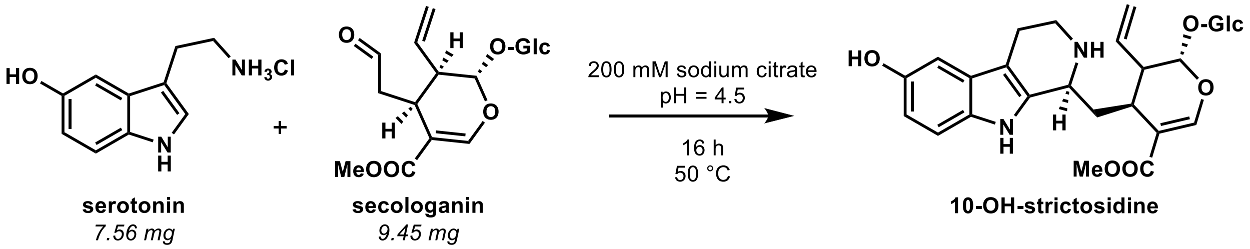

For the synthesis of (20*S*)-10-hydroxycorynantheidine: to a 1.5 mL microcentrifuge tube was added 7.56 mg serotonin HCl (0.036 mmol, [final] = 89 mM) and 9.45 mg secologanin (0.024 mmol, [final] = 61 mM). 0.4 mL 200 mM sodium citrate buffer pH = 4.5 was then added, and the reaction heated to 40 °C with shaking for 16 h. Full tryptamine conversion was observed, and so the mixture was injected onto preparative HPLC for purification. Fractions corresponding to 10-hydroxystrictosidine and 10-hydroxyvincoside were collected and pooled. These pooled fractions were evaporated to remove ACN, frozen, and lyophilized to remove solvent. This resulted in 4.1 mg of 10-hydroxystrictosidine (white powder, 31% yield), and 3.1 mg of 10-hydroxyvincoside (white powder, 24% yield). The 10-hydroxystrictosidine was immediately used for subsequent reaction.

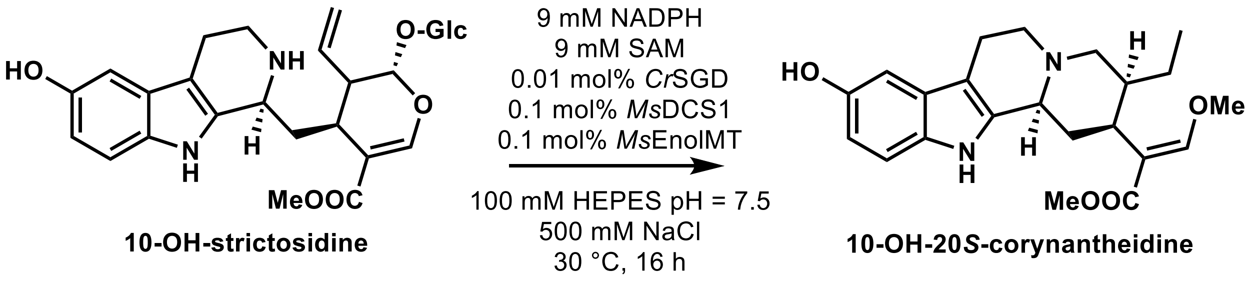

To a 5 mL vial was added 4.1 mg 10-hydroxystrictosidine (0.008 mmol, [final] = 8 mM), 7.5 mg β-Nicotinamide-adenine Dinucleotide Phosphate (NADPH), tetrasodium salt (0.009 mmol, [final] = 9 mM), 4.7 mg S-Adenosyl Methionine (SAM) iodide (0,009 mmol, [final] = 9 mM), 1 µM *Cr*SGD, 10 µM *Ms*DCS1, and 10 µM *Ms*EnolMT. The solution was diluted to 1 mL with 100 mM HEPES + 500 mM NaCl pH = 7.5 and allowed to incubate at 30 °C for 16 h.

The reaction was quenched via addition of 1 mL MeOH, followed by centrifugation to pellet any aggregated protein. The supernatant was removed and the pellet washed with 1 mL 1:1 MeOH : H_2O, followed again by centrifugation. The combined supernatant fractions were pooled, and the mixture was injected onto preparative HPLC for purification. Fractions corresponding to (20*S*)-10-hydroxycorynantheidine were collected and pooled. These pooled fractions were evaporated to remove ACN, frozen, and lyophilized to remove solvent. From this procedure, 0.31 mg of (20*S*)-10-hydroxycorynantheidine (yellow residue, 11% yield) was obtained.

(20*S*)-10-hydroxycorynantheidine (16a): ^{1}H-NMR (700 MHz, MeOH-d_3) δ ppm: 7.58 (*s*, 1H), 7.14 (*d*, *J*= 8.7 Hz, 1H), 6.80 (*d*, *J*= 2.3 Hz, 1H), 6.66 (*dd*, *J*= 8.7, 2.3 Hz, 1H), 4.03 (*m*, 1H), 3.82 (*s*, 3H), 3.70 (*s*, 3H), 3.50 (*m*, 1H), 3.46 (*brd*, *J*= 12.7 Hz, 1H), 3.26 (*m*, 1H), 3.16 (*m*, 1H), 3.13 (*m*, 1H), 3.06 (*brd*, *J*= 12.7 Hz, 1H), 2.83 (*m*, 1H), 2.64 (*ddd*, *J*= 13.4, 13.4, 13.0 Hz, 1H), 2.31 (*m*, 1H), 1.88 (*m*, 1H), 1.71 (*m*, 1H), 1.40 (*m*, 1H), 0.92 (*t*, *J*= 7.4 Hz, 3H). ^{13}C-NMR (126 MHz, $CDCl_3$) δ ppm: 170.0, 162.6, 112.42, 112.37, 103.1, 63.9, 62.1, 57.3, 54.6, 51.6, 40.6, 39.3, 28.9, 20.6, 19.6, 12.7. Due to low amount of the compound, only partial assignment was possible in ^{13}C. Corresponding spectra are shown in Supplementary Figures 73-74.
m/z $[M+H]^+$ – calculated: 385.2127; observed: 385.2119.

Structural Data

General Methods

NMR measurements were carried out on a 400 MHz Bruker Avance III HD spectrometer (Bruker Biospin GmbH, Rheinstetten, Germany), a 700 MHz Bruker Avance III HD spectrometer (Bruker Biospin GmbH, Rheinstetten, Germany) and a 500 MHz Bruker Avance III HD spectrometer (Bruker Biospin GmbH, Rheinstetten, Germany) using standard pulse sequences as implemented in Bruker Topspin ver. 3.6.1. 700 MHz and 500 MHz NMR were equipped with a TCI cryoprobe. Chemical shifts were referenced to the residual solvent signals of $CDCl_3$ (δ_H 7.26/δ_C 77.16), MeOD and MeOH-*d₃* (δ_H 3.31/δ_C 49.0), D_2O (δ_H 4.70) respectively. Chemical shift values (δ_H) are reported in parts per million (ppm) and coupling constants (*J*) are expressed in Hertz (Hz), in the following format; chemical shift value (multiplicity, coupling constant, integration). ^{1}H NMR spectral data are described, using the following abbreviations; s (singlet), d (doublet), t (triplet), dd (doublet of doublets), appbrs (apparent broad singlet), and m (multiplet). All spectra were recorded at 298 K.

NMR data

(20*S*)-corynantheidine (3a): ^{1}H-NMR (500 MHz, $CDCl_3$) δ ppm: 7.71 (*brs*, NH, 1H), 7.46 (*brd, J*= 7.8 Hz, 1H), 7.44 (*s*, 1H), 7.29 (*brd*, *J*= 7.8 Hz, 1H), 7.11 (*ddd*, *J*= 7.8, 7.2, 1.4 Hz, 1H), 7.07 (*ddd*, *J*= 7.8, 7.2, 1.2 Hz, 1H), 3.73 (*s*, 3H), 3.71 (*s*, 3H), 3.20 (*m*, 1H), 3.08-2.94 (*m*, 4H), 2.70 (*m*, 1H), 2.57 (*m*, 1H), 2.53 (*m*, 1H), 2.49 (*ddd, J*= 11.5, 3.1, 0.7 Hz, 1H), 1.84 (*m*, 1H), 1.78 (*m*, 1H), 1.64 (*m*, 1H), 1.21 (*m*, 1H), 0.87 (*t*, *J*= 7.5 Hz, 3H). ^{13}C-NMR (126 MHz, $CDCl_3$) δ ppm: 169.3, 160.7, 136.0, 135.8, 127.7, 121.3, 119.5, 118.3, 111.6, 110.8, 108.3, 61.7, 61.4, 57.9, 53.7, 51.5, 40.9, 40.1, 30.1, 22.1, 19.3, 13.0. The chemical shifts were in agreement with published data (Stærk *et al*. 2000, Wanner *et al*. 2011).[13,14] Corresponding spectra are shown in Supplementary Figure 54.
m/z $[M+H]^+$ – calculated: 369.2172; observed: 369.2169.

(20*R*)-corynantheidine (3b): ^{1}H-NMR (700 MHz, $CDCl_3$) δ ppm: 7.46 (*brd*, *J*= 7.6 Hz, 1H), 7.36 (*brs*, 1H), 7.27 (*d*, *J*= 7.8 Hz, 1H), 7.11 (*brdd*, *J*= 7.8, 7.4 Hz, 1H), 7.07 (*brdd*, *J*= 7.6, 7.4 Hz, 1H), 3.92-3.61 (*m*, 6H), 3.33 (*m*, 1H), 3.25-3.13 (*m*, 2H), 3.06 (*m*, 1H), 2.75 (*m,*1H), 2.70-2.61 (*m*, 2H), 2.34-1.87 (*m*, 4H), 1.43 (*m*, 1H), 1.06 (*m*, 1H), 0.88 (*t*, *J*= 7.0 Hz, 3H). ^{13}C-NMR (176 MHz, $CDCl_3$) δ ppm: 168.3, 160.1, 136.3, 127.6, 121.7, 119.6, 118.4, 108.1, 61.8, 60.9, 60.5, 53.2, 51.5, 40.1, 38.7, 33.8, 24.5, 21.6, 11.4. Due to low amount (0.09 mg) and broad signals, HSQC and HMBC were used for the assignment and only partial assignment was possible. The chemical shifts were in agreement with published data (Ren *et al*. 2024).[15] Corresponding spectra are shown in Supplementary Figure 55.
m/z $[M+H]^+$ – calculated: 369.2172; observed: 369.2171.

(20*S*)-isocorynantheidine (6a): ^{1}H-NMR (700 MHz, $CDCl_3$) δ ppm: 7.46 (*d*, *J*= 7.8 Hz, 1H), 7.45 (*s*, 1H), 7.32 (*d*, *J*= 7.6 Hz, 1H), 7.14-7.07 (*m*, 2H), 4.45 (*brs*, 1H), 3.81 (*s*, 3H), 3.67 (*s*, 3H), 3.36 (*m*, 2H), 3.15 (*m,*1H), 3.13 (*m,*1H), 2.74 (*m,*1H), 2.68 (*m,*1H), 2.07 (*m,*1H), 1.62 (*m*, 1H), 1.25 (*m*, 1H), 0.89 (*t*, *J*= 7.3 Hz, 3H). ^{13}C-NMR (176 MHz, $CDCl_3$) δ ppm: 169.3, 160.9, 136.2, 127.3, 121.9, 119.8, 118.1, 111.3, 110.7, 107.6, 61.9, 55.3, 52.4, 51.6, 38.9, 20.8, 12.5. Due to low amount (0.17 mg) and broad signals, HSQC and HMBC were used for the assignment and only partial assignment was possible. The chemical shifts were in agreement with published data (Lounasmaa *et al.* 1998).[16] Corresponding spectra are shown in Supplementary Figure 56.
m/z $[M+H]^+$ – calculated: 369.2172; observed: 369.2166.

hirsutine (6b): ^{1}H-NMR (500 MHz, $CDCl_3$) δ ppm: 8.91 (*brs*, NH, 1H), 7.48 (*brd, J*= 7.7 Hz, 1H), 7.44 (*d*, *J*= 8.2 Hz, 1H), 7.34 (*s*, 1H), 7.22 (*dd*, *J*= 8.2, 7.4 Hz, 1H), 7.15 (*dd*, *J*= 7.7, 7.4 Hz, 1H), 4.87 (*brs*, 1H), 3.79 (*brs*, 3H), 3.69 (*s*, 3H), 3.43 (*m*, 2H), 3.10 (*dd*, *J*= 11.4, 3.1 Hz, 1H), 3.01 (*m*, 1H), 2.76 (*m*, 1H), 2.69 (*m*, 1H), 2.61 (*dd*, *J*= 11.4, 11.4 Hz, 1H), 2.47 (*m*, 1H), 2.30 (*brdd, J*= 11.5, 11.5 Hz, 1H), 2.17 (*brd, J*= 13.4 Hz, 1H), 1.35 (*m*, 1H), 0.82 (*m*, 1H), 0.75 (*t*, *J*= 7.2 Hz, 3H). ^{13}C-NMR (126 MHz, $CDCl_3$) δ ppm: 168.6, 160.5, 136.8, 129.2, 127.0, 122.4, 120.0, 118.3, 111.8, 110.2, 106.3, 61.9, 54.3, 51.6, 50.2, 49.7, 37.0, 33.5, 30.3, 24.1, 16.4, 10.9. The chemical shifts were in agreement with published data (Ren *et al*. 2024).[15] Corresponding spectra are shown in Supplementary Figure 57.
m/z $[M+H]^+$ – calculated: 369.2172; observed: 369.2164.

mitragynine (5a): ^{1}H-NMR (500 MHz, $CDCl_3$) δ ppm: 7.68 (*brs*, NH, 1H), 7.43 (*s*, 1H), 6.99 (*dd*, *J*= 8.1, 7.7 Hz, 1H), 6.90 (*dd*, *J*= 8.1, 0.6 Hz, 1H), 6.45 (*brd*, *J*= 7.7 Hz, 1H), 3.87 (*s*, 3H), 3.73 (*s*, 3H), 3.71 (*s*, 3H), 3.16 (*brd*, *J*= 11.4 Hz, 1H), 3.12 (*m*, 1H), 3.06-2.89 (*m*, 4H), 2.53 (*ddd*, *J*= 11.5, 11.5, 4.2 Hz, 1H), 2.51 (*m*, 1H), 2.45 (*m*, 1H), 1.80 (*m*, 1H), 1.78 (*m*, 1H), 1.62 (*m*, 1H), 1.20 (*m*, 1H), 0.87 (*t*, *J*= 7.4 Hz, 3H). ^{13}C-NMR (126 MHz, $CDCl_3$) δ ppm: 169.4, 160.7, 154.7, 137.4, 133.8, 122.0, 117.8, 111.7, 108.1, 104.3, 99.9, 61.7, 61.4, 57.9, 55.5, 53.9, 51.5, 40.8, 40.1, 30.1, 24.1, 19.2, 13.0. The chemical shifts were in agreement with published data (Flores-Bocanegra *et al*. 2020).[17] Corresponding spectra are shown in Supplementary Figure 58.
m/z $[M+H]^+$ – calculated: 399.2278; observed: 399.2289.

speciogynine (5b): ^{1}H-NMR (700 MHz, $CDCl_3$) δ ppm: 7.37 (*brs*, 1H), 7.01 (*brdd*, *J*= 7.7, 7.5 Hz, 1H), 6.92 (*brs*, 1H), 6.43 (*brd*, *J*= 7.5 Hz, 1H), 3.94-3.55 (*m*, 9H), 3.55-3.08 (*m*, 6H), 2.66 (*m*, 1H), 2.27 (*m*, 2H), 2.03 (*m*, 1H), 1.41 (*m*, 1H), 1.04 (*m*, 1H), 0.88 (*t*, *J*= 6.9 Hz, 3H). ^{13}C-NMR (176 MHz, $CDCl_3$) δ ppm: 168.5, 160.4, 154.6, 137.9, 122.7, 117.2, 104.8, 99.9, 62.0, 60.6, 59.7, 55.4, 54.6, 39.1, 32.7, 24.1, 22.8, 14.2. Due to low amount (0.31 mg) and broad signals, HSQC and HMBC were used for the assignment and only partial assignment was possible. The chemical shifts were in agreement with

published data (Flores-Bocanegra *et al.* 2020).[17] Corresponding spectra are shown in Supplementary Figure 59.
m/z [M+H]$^+$ – calculated: 399.2278; observed: 399.2275.

speciociliatine (7a): ^{1}H-NMR (700 MHz, $CDCl_3$) δ ppm: 7.80 (*brs*, NH, 1H), 7.42 (*s*, 1H), 7.00 (*dd*, *J*= 8.1, 7.8 Hz, 1H), 6.91 (*d*, *J*= 8.1, 1H), 6.47 (*d*, *J*= 7.8 Hz, 1H), 4.13 (*brs*, 1H), 3.89 (*s*, 3H), 3.77 (*s*, 3H), 3.66 (*s*, 3H), 3.19 (*m,*1H), 3.12 (*dd*, *J*= 12.7, 5.8 Hz, 1H), 3.00 (*m*, 1H), 2.97 (*m,*1H), 2.90 (*m*, 2H), 2.77 (*dd, J*= 11.3, 5.8 Hz, 1H), 2.62 (*m*, 1H), 1.91 (*m*, 1H), 1.75 (*m*, 1H), 1.63 (*m*, 1H), 1.25 (*m*, 1H), 0.89 (*t*, *J*= 7.4 Hz, 3H). ^{13}C-NMR (176 MHz, $CDCl_3$) δ ppm: 169.6, 160.1, 154.5, 137.2, 132.1, 121.9, 118.0, 111.6, 108.1, 104.5, 99.8, 61.5, 55.4, 54.7, 52.7, 51.5, 50.3, 39.8, 33.8, 30.1, 20.9, 20.5, 12.7. Due to some very weak ^{13}C signals, HSQC and HMBC were used for the assignment. The chemical shifts were in agreement with published data (Flores-Bocanegra *et al.* 2020).[17] Corresponding spectra are shown in Supplementary Figure 60.
m/z [M+H]$^+$ – calculated: 399.2278; observed: 399.2277.

mitraciliatine (7b): ^{1}H-NMR (700 MHz, $CDCl_3$) δ ppm: 7.32 (*s*, 1H), 7.06 (*dd*, *J*= 8.1, 7.7 Hz, 1H), 7.01 (*d*, *J*= 8.1, 1H), 6.50 (*d*, *J*= 7.7 Hz, 1H), 4.61 (*brs*, 1H), 3.90 (*s*, 3H), 3.78 (*brs*, 3H), 3.69 (*s*, 3H), 3.34 (*m*, 2H), 3.21 (*m,*1H), 2.99 (*brd, J*= 16.4 Hz, 1H), 2.92 (*brd, J*= 10.5 Hz, 1H), 2.56 (*m*, 1H), 2.51 (*brdd, J*= 11.3, 11.3 Hz, 1H), 2.31 (*m*, 1H), 2.25 (*m*, 1H), 2.02 (*m*, 1H), 1.33 (*m*, 1H), 0.80 (*m*, 1H), 0.76 (*t*, *J*= 7.1 Hz, 3H). ^{13}C-NMR (176 MHz, $CDCl_3$) δ ppm: 169.1, 160.1, 154.5, 137.6, 129.3, 122.5, 117.8, 111.2, 107.3, 104.8, 99.8, 61.8, 55.4, 54.3, 51.5, 51.3, 50.2, 38.2, 34.4, 31.3, 24.3, 18.9, 11.2. The chemical shifts were in agreement with published data (Flores-Bocanegra *et al.* 2020).[17] Corresponding spectra are shown in Supplementary Figure 61.
m/z [M+H]$^+$ – calculated: 399.2278; observed: 399.2271.

3-dehydromitragynine – TFA salt (DHM, 14a) from isolation: ^{1}H-NMR (500 MHz, $CDCl_3$) δ ppm: 12.0 (*s*, 1H), 7.52 (*s*, 1H), 7.29 (*dd*, *J*= 8.3, 7.7 Hz, 1H), 7.13 (*d*, *J*= 8.3 Hz, 1H), 6.42 (*d*, *J*= 7.7 Hz, 1H), 3.95-3.80 (*m*, 2H), 3.91 (*s*, 3H), 3.78 (*s*, 3H), 3.62 (*s*, 3H), 3.60-3.53 (*m*, 4H), 3.53-3.38 (*m*, 2H), 3.32 (*dd*, *J*= 12.3, 11.4 Hz, 1H), 2.12 (*m*, 1H), 1.49 (*m*, 1H), 1.18 (*m*, 1H), 0.98 (*t*, *J*= 7.4 Hz, 3H). ^{13}C-NMR (126 MHz, $CDCl_3$) δ ppm: 168.5, 167.2, 162.7, 156.0, 143.5, 130.4, 125.4, 122.6, 107.8, 107.1, 100.2, 62.2, 55.5, 54.6, 53.1, 51.7, 38.6, 31.6, 28.1, 23.2, 21.5, 11.8. The chemical shifts were in agreement with published data (Chakraborty *et al.* 2021).[9] Corresponding spectra are shown in Supplementary Figure 62 Supplementary .
m/z [M]$^+$ – calculated: 397.2122; observed: 397.2123.

3-dehydrocorynantheidine (DHC, 13a): ^{1}H-NMR (500 MHz, $CDCl_3$) δ ppm: 7.80 (*brd*, *J*= 7.9 Hz, 1H), 7.54 (*d*, *J*= 8.1 Hz, 1H), 7.50 (*s*, 1H), 7.37 (*brdd*, *J*= 7.9, 7.6 Hz, 1H), 7.13 (*dd*, *J*= 8.1, 7.6 Hz, 1H), 3.98-3.82 (*m*, 3H), 3.77 (*s*, 3H), 3.74 (*m*, 1H), 3.61 (*s*, 3H), 3.65-3.54 (*m*, 2H), 3.37-3.26 (*m*, 3H), 2.10 (*m*, 1H), 1.49 (*m*, 1H), 1.17 (*m*, 1H), 0.99 (*t*, *J*= 7.3 Hz, 3H). ^{13}C-NMR (126 MHz, $CDCl_3$) δ ppm: 168.5, 168.5, 162.6, 142.6, 128.5, 126.7, 123.9, 121.7, 120.6, 115.5, 108.1, 62.2, 54.7, 53.2, 51.7, 38.8, 32.8, 28.1, 23.3, 20.2, 11.8. Detailed assignment shown in Supplementary Figures 65-68 and Supplementary Table 4.
m/z $[M]^+$ – calculated: 367.2016; observed: 367.2011.

isoajmalicine (11b): ^{1}H-NMR (500 MHz, $CDCl_3$) δ ppm: 8.38 (*brs*, NH, 1H), 7.50 (*d, J*= 1.6 Hz, 1H), 7.49 (*brd*, *J*= 8.0 Hz, 1H), 7.41 (*ddd*, *J*= 8.1, 0.9, 0.9 Hz, 1H), 7.19 (*ddd*, *J*= 8.1, 7.2, 0.9 Hz, 1H), 7.12 (*ddd*, *J*= 8.0, 7.2, 0.9 Hz, 1H), 4.65 (*m*, 1H), 4.35 (*qd*, *J*= 6.7, 4.2 Hz, 1H), 3.73 (*s*, 3H), 3.34 (*m*, 2H), 3.22 (*ddd*, *J*= 14.0, 2.6, 2.6 Hz, 1H), 3.01 (*dddd*, *J*= 16.2, 10.4, 8.4, 2.7 Hz, 1H), 2.76 (*dd*, *J*= 10.7, 3.0 Hz, 1H), 2.70 (*m*, 1H), 2.58 (*dd*, *J*= 11.1, 10.7 Hz, 1H), 2.11 (*m*, 1H), 2.00 (*m*, 1H), 1.73 (*ddd*, *J*= 14.0, 12.0, 5.0 Hz, 1H), 0.92 (*d*, *J*= 6.7 Hz, 3H). ^{13}C-NMR (126 MHz, $CDCl_3$) δ ppm: 167.5, 154.9, 136.1, 131.7, 127.7, 121.9, 119.7, 118.2, 111.5, 107.6, 106.6, 73.7, 54.2, 51.2, 50.6, 47.1, 40.8, 30.9, 26.0, 16.8, 15.1. The chemical shifts were in agreement with published data (Ren *et al*. 2024).[15] Corresponding spectra are shown in Supplementary Figure 69.
m/z $[M+H]^+$ – calculated: 353.1870; observed: 353.1859.

isomitraphylline (12b$_i$): ^{1}H-NMR (700 MHz, $CDCl_3$) δ ppm: 8.02 (*brs*, NH, 1H), 7.39 (*s,* 1H), 7.35 (*m*, 1H), 7.17 (*brdd*, *J*= 7.6, 7.2 Hz, 1H), 7.00 (*m*, 1H), 6.86 (*brd*, *J*= 7.6 Hz, 1H), 4.37 (*m*, 1H), 3.57 (*s*, 3H), 3.29 (*m*, 1H), 3.13 (*m*, 1H), 2.61 (*m*, 1H), 2.54 (*m*, 1H), 2.42 (*m*, 1H), 2.21 (*m*, 1H), 2.18 (*m*, 1H), 2.04 (*m*, 1H), 1.93 (*m*, 1H), 1.91 (*m*, 1H), 1.12 (*d*, *J*= 6.6 Hz, 3H), 0.61 (*m*, 1H). ^{13}C-NMR (126 MHz, $CDCl_3$) δ ppm: 181.3, 167.2, 154.1, 140.3, 134.0, 127.7, 125.1, 122.5, 109.7, 107.4, 74.2, 72.0, 56.5, 54.4, 53.5, 51.0, 41.0, 35.6, 30.2, 29.2, 15.0. The ^{13}C chemical shifts were in agreement with published data (Paradowska *et al*. 2008).[18] Corresponding spectra are shown in Supplementary Figure 70.
m/z $[M+H]^+$ – calculated: 369.1820; observed: 369.1794.

isocorynoxeine (8c$_i$): ^{1}H-NMR (700 MHz, $CDCl_3$) δ ppm: 7.77 (*brs*, NH, 1H), 7.45 (*brd*, *J*= 7.4 Hz, 1H), 7.17 (*ddd*, *J*= 7.7, 7.7, 1.2 Hz, 1H), 7.15 (*s,* 1H), 7.04 (*brdd*, *J*= 7.7, 7.4 Hz, 1H), 6.84 (*brd*, *J*= 7.7 Hz, 1H), 5.51 (*ddd*, *J*= 17.2, 10.3, 8.8 Hz, 1H), 4.95 (*brd*, *J*= 17.2 Hz, 1H), 4.90 (*brdd*, *J*= 10.3, 1.9 Hz, 1H), 3.69 (*s*, 3H), 3.57 (*s*, 3H), 3.30 (*ddd*, *J*= 8.6, 8.6, 2.1 Hz, 1H), 3.19 (*dd*, *J*= 10.9, 4.0 Hz, 1H), 2.83 (*m*, 1H), 2.54-2.47 (*m*, 3H), 2.39 (*ddd*, *J*= 13.1, 9.1, 2.1 Hz, 1H), 2.06 (*ddd*, *J*= 13.1, 8.6, 8.6 Hz, 1H), 1.99 (*dd*, *J*= 11.0, 10.9 Hz, 1H), 1.48 (*m*, 1H), 1.07 (*m*, 1H). ^{13}C-NMR (126 MHz, $CDCl_3$) δ ppm: 181.8, 168.4, 159.6, 140.1, 139.8, 134.1, 127.6, 125.4, 122.5, 115.5, 112.1, 109.3, 72.2, 61.4, 58.9, 56.9, 54.1, 51.1, 42.6, 37.8, 35.7, 29.7. The chemical shifts were in agreement with published data (Kitajima *et al*.

2001).[19] Corresponding spectra are shown in Supplementary Figure 71.

m/z $[M+H]^+$ – calculated: 383.1965; observed: 385.1965.

corynoxeine (8c$_{ii}$): See Supplementary Table 5 for detailed assignments. Corresponding spectra are shown in Supplementary Figure 72.

m/z $[M+H]^+$ – calculated: 383.1965; observed: 385.1960.

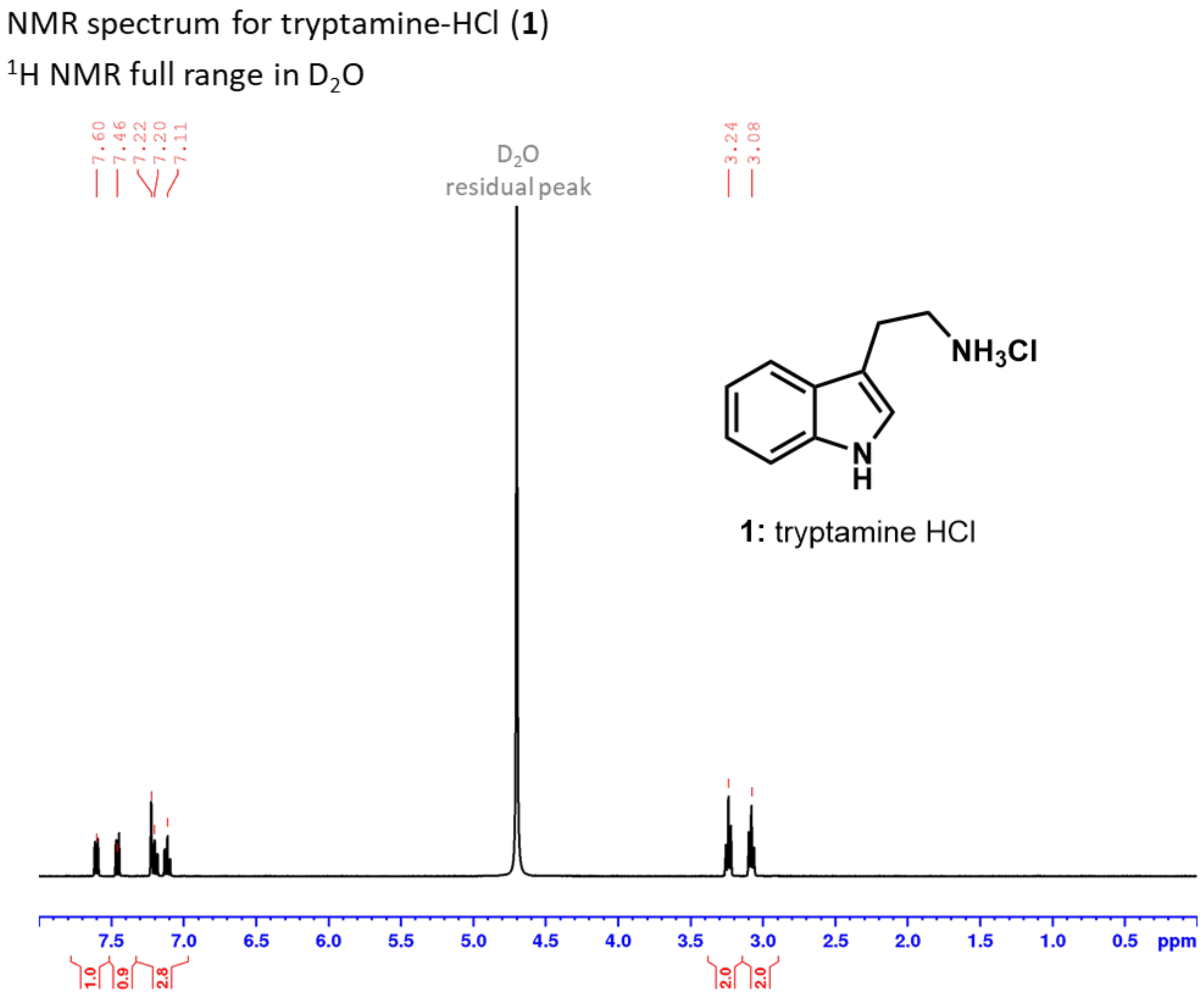

Supplementary Figure 45: NMR spectrum for **1:** tryptamine-HCl.

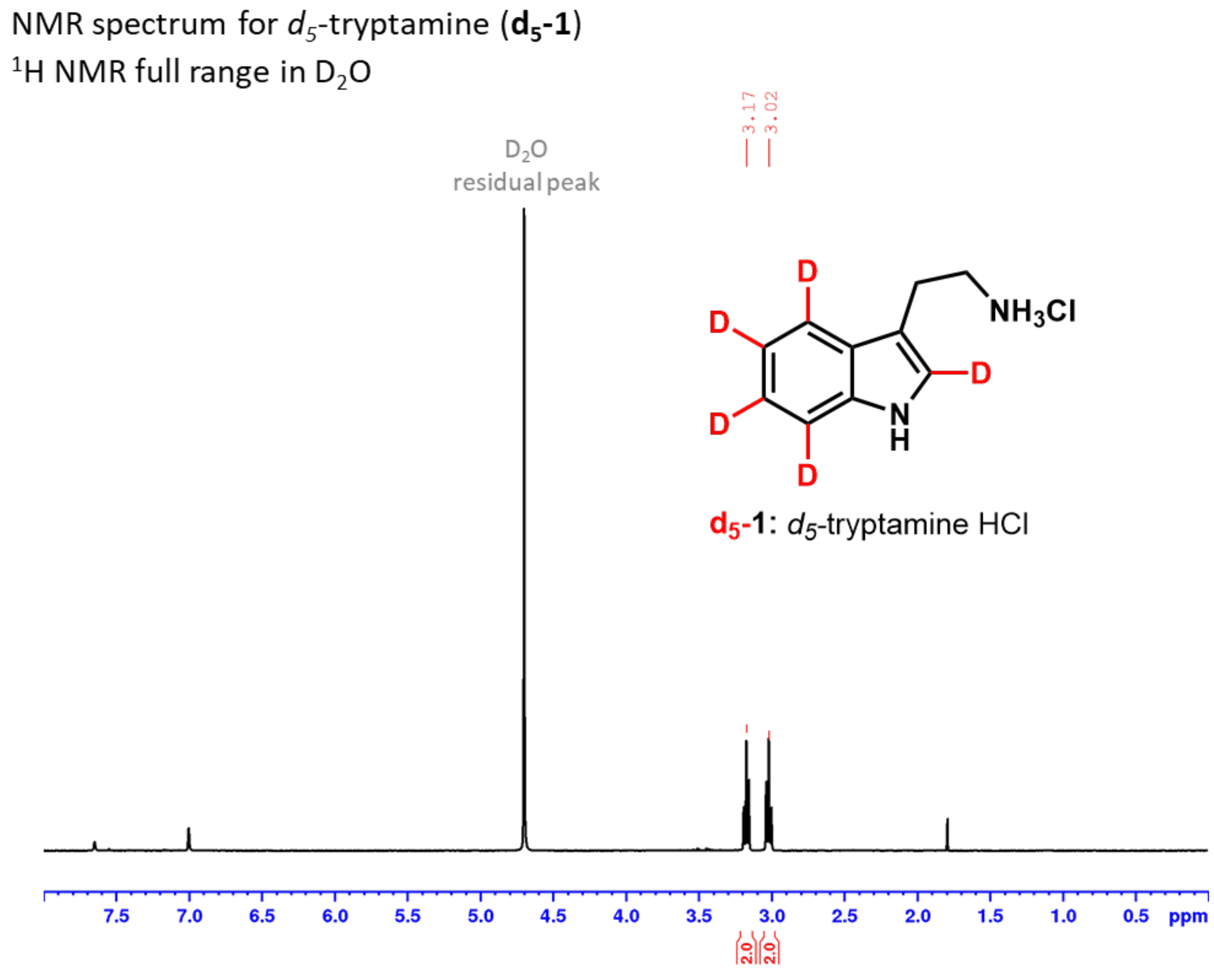

Supplementary Figure 46: NMR spectrum for ***d*$_5$-1:** *d*$_5$-tryptamine-HCl.

NMR spectra for d_4-strictosidine (**d_4-2a**)

^{1}H NMR with water presaturation full range in MeOH-d_3

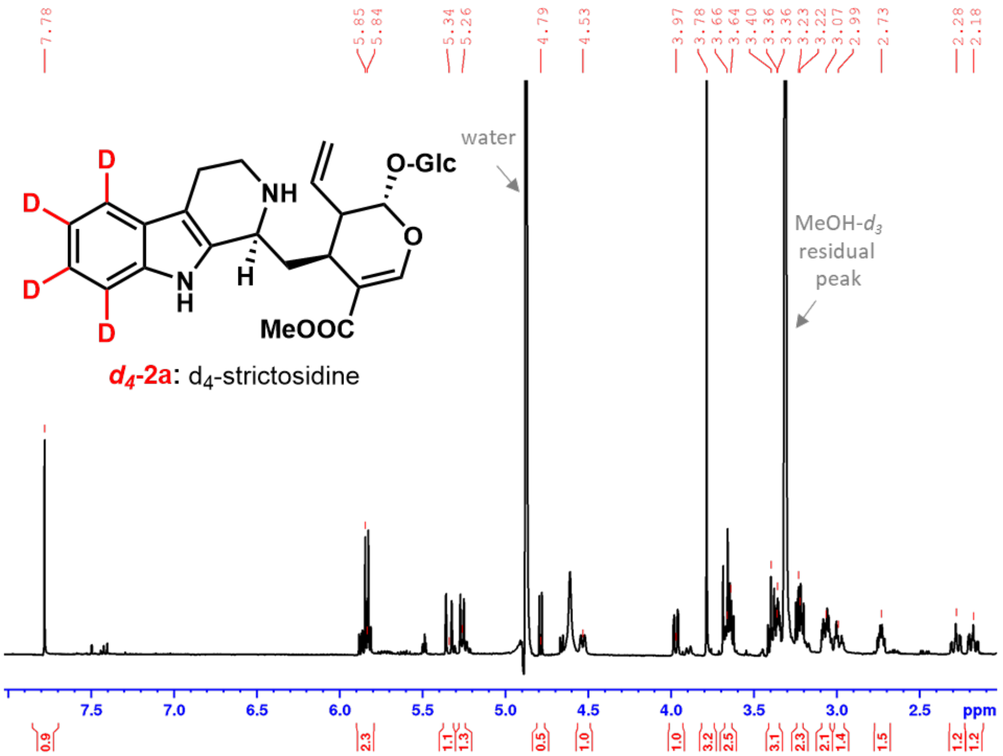

DEPTQ full range in MeOH-d_3

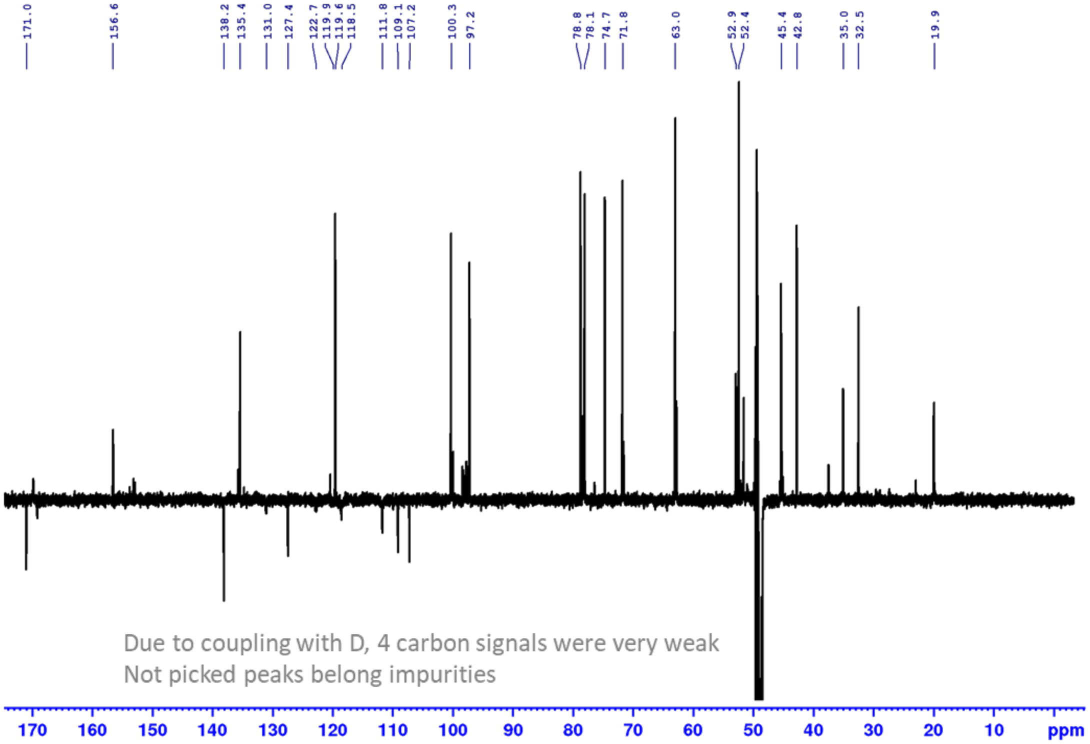

Supplementary Figure 47: NMR spectrum for *d_4*-strictosidine (***d_4*-2a**).

NMR spectrum for vincoside (**2b**)

^{1}H NMR full range in MeOD

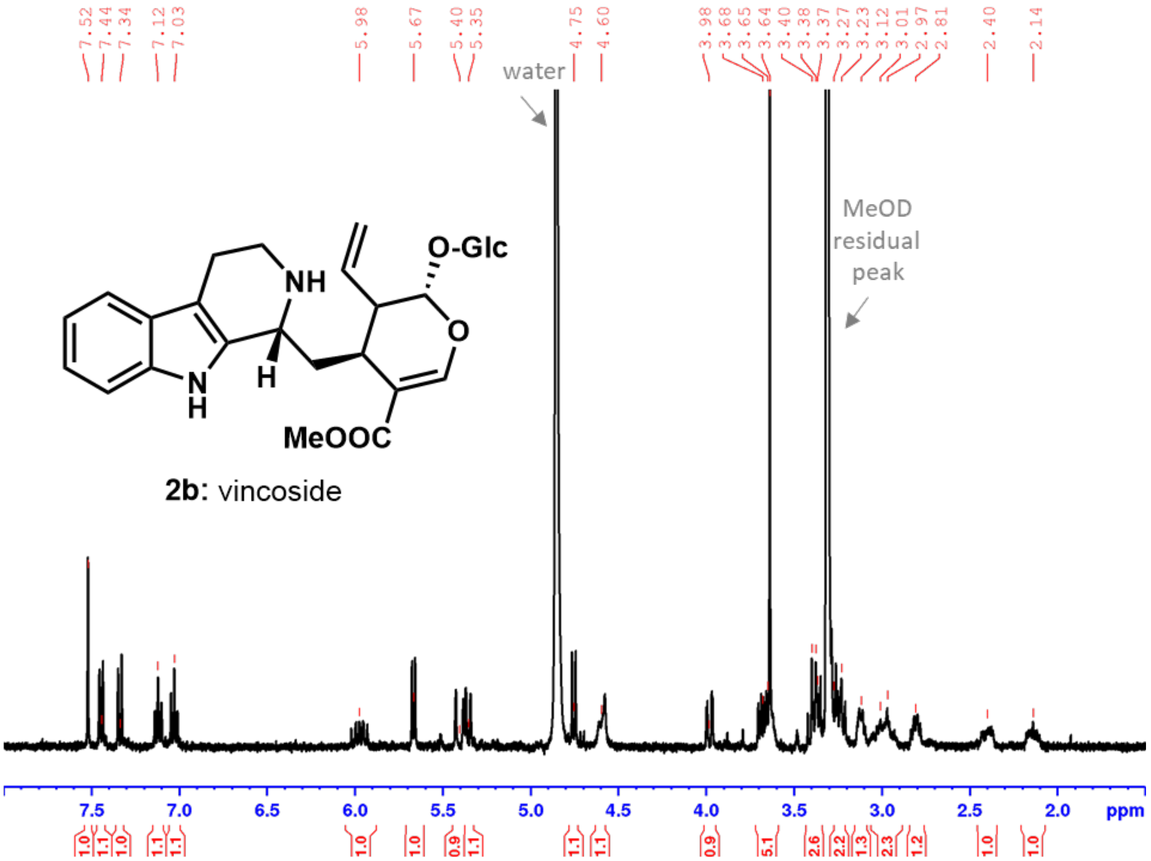

Supplementary Figure 48: NMR spectrum for vincoside (**2b**).

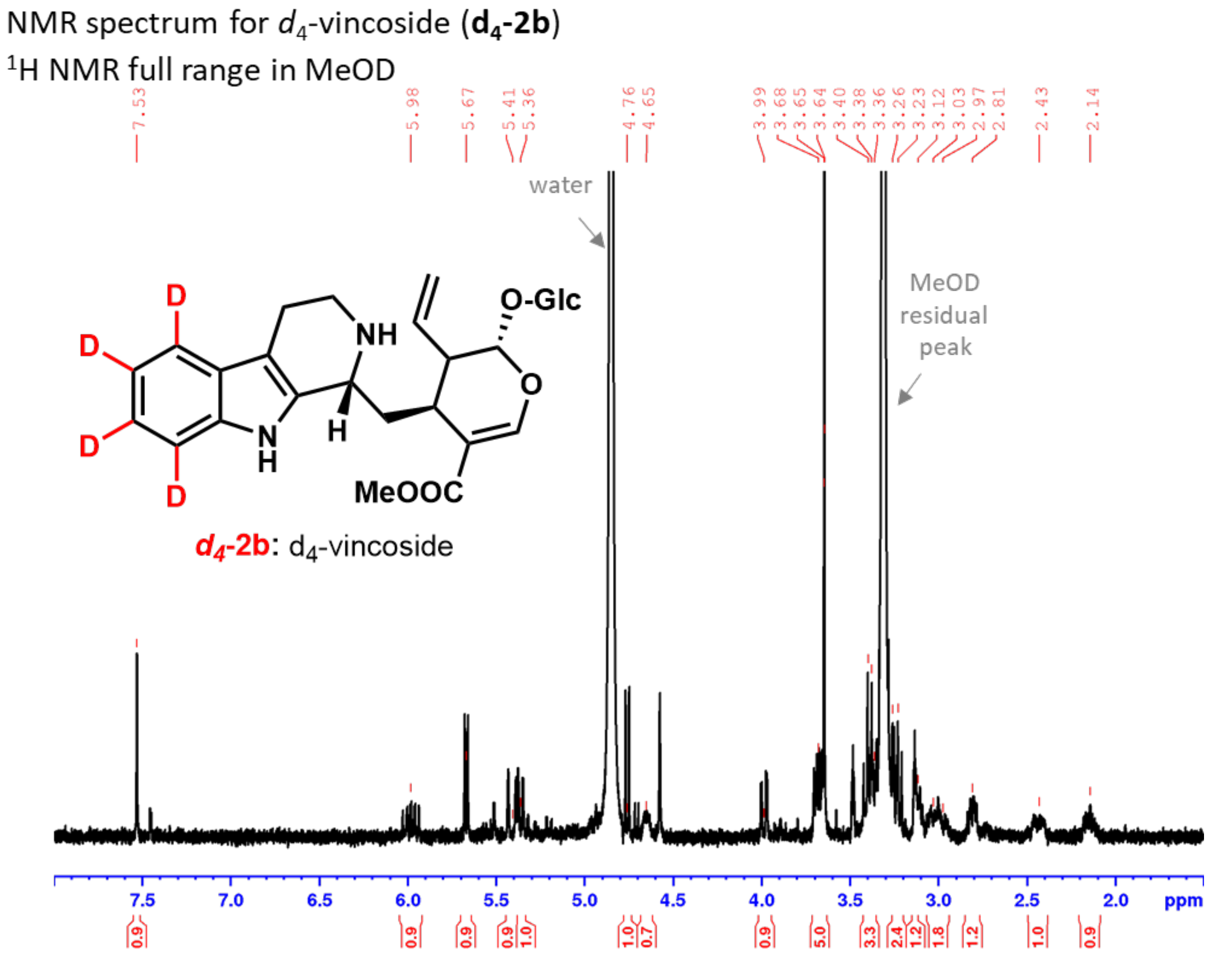

Supplementary Figure 49: NMR spectrum for d_4-vincoside (***d_4*-2b**).

NMR spectrum for 4-OMe-tryptamine HCl

^{1}H NMR full range in D_2O

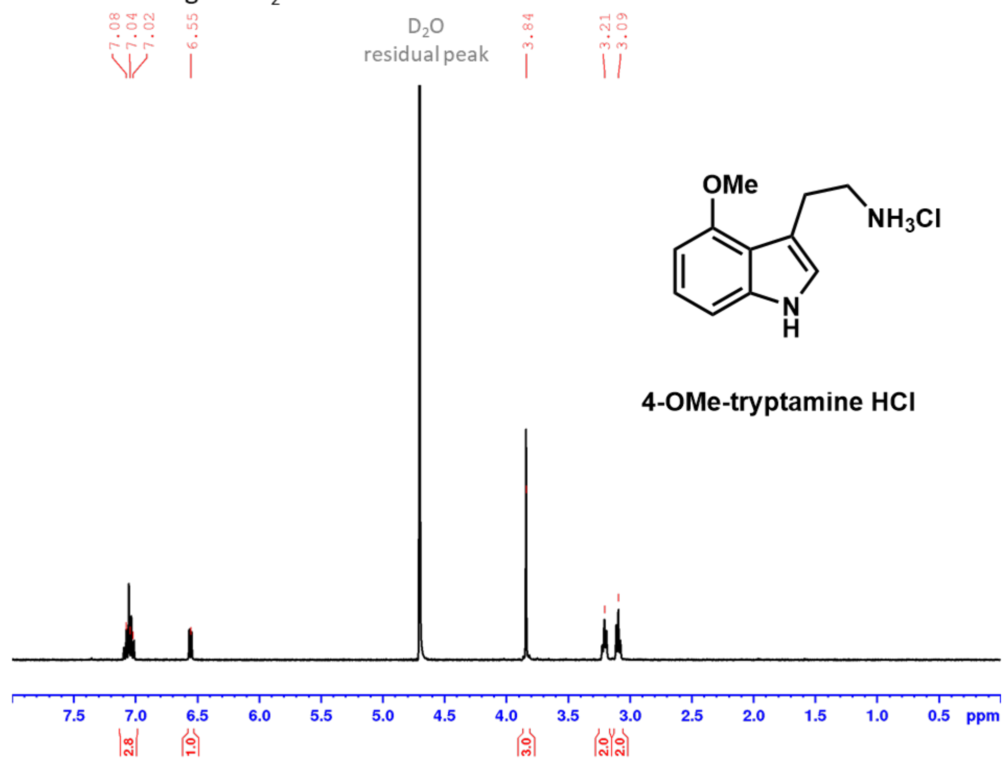

NMR spectrum for d_2-4-OMe-tryptamine HCl

^{1}H NMR full range in D_2O

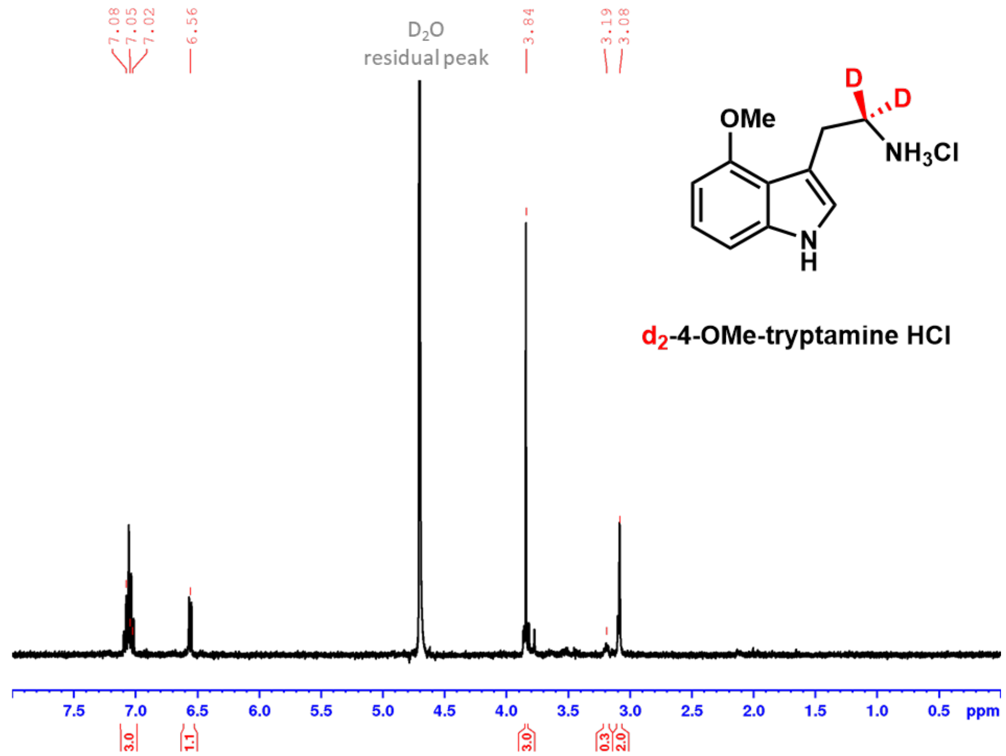

Supplementary Figure 50: NMR spectra for 4-methoxytryptamine-HCl and d_2-4-methoxytryptamine-HCl.

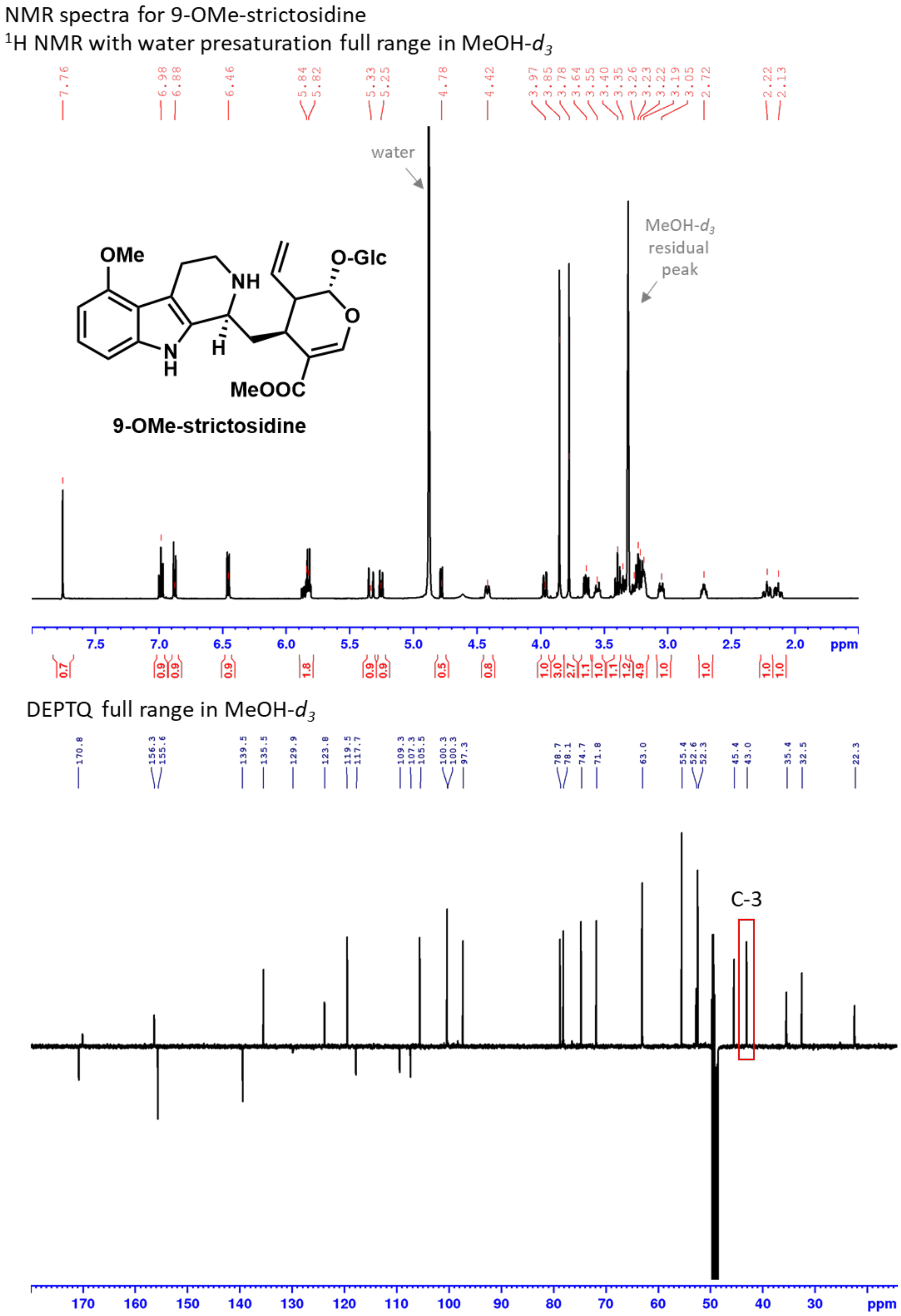

Supplementary Figure 51: NMR spectra for 9-methoxystrictosidine.

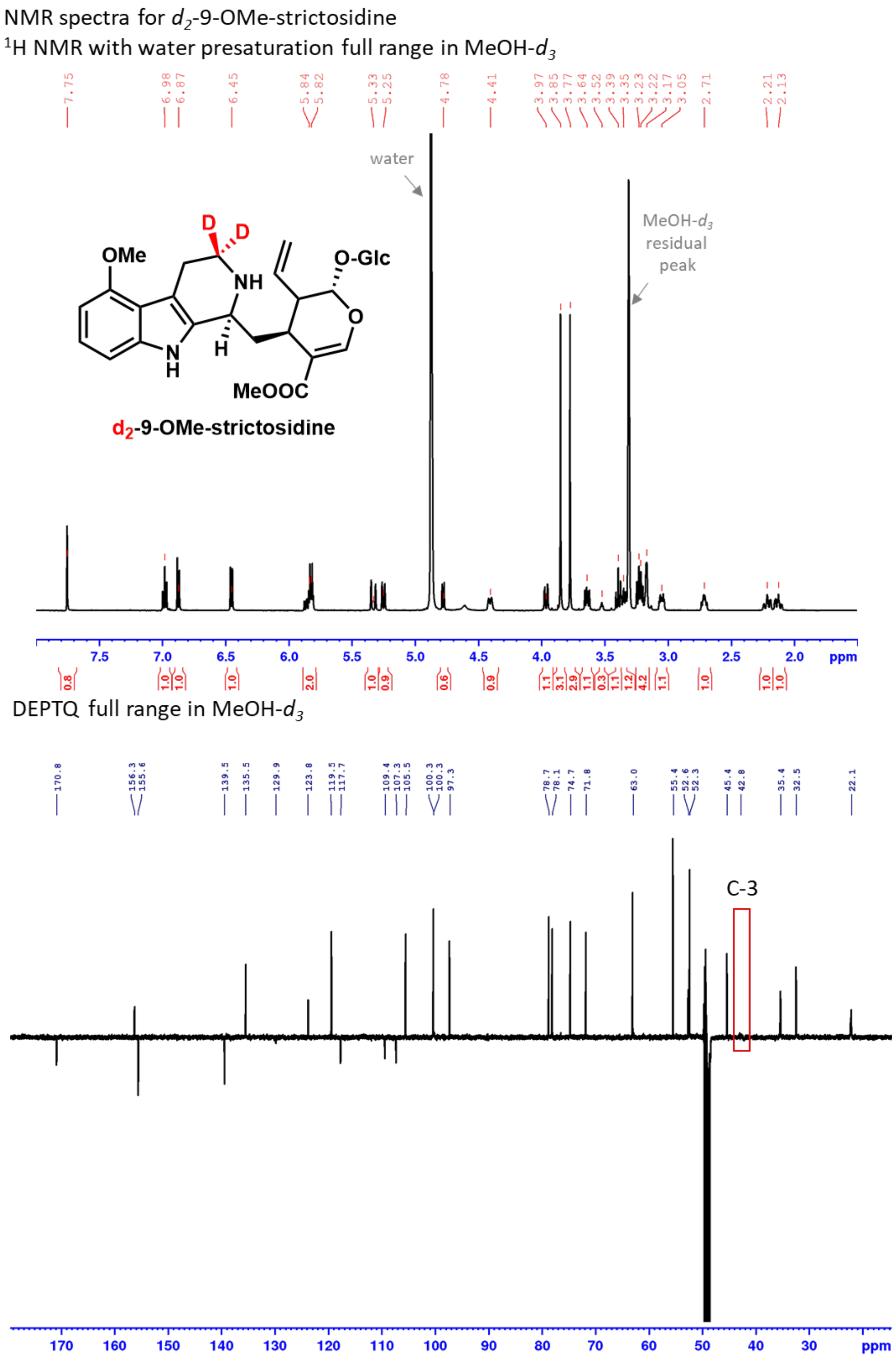

Supplementary Figure 52: NMR spectra for d_2-9-methoxystrictosidine.

^{1}H NMR with water presaturation of 9-OMe-strictosidine (3-4 ppm)

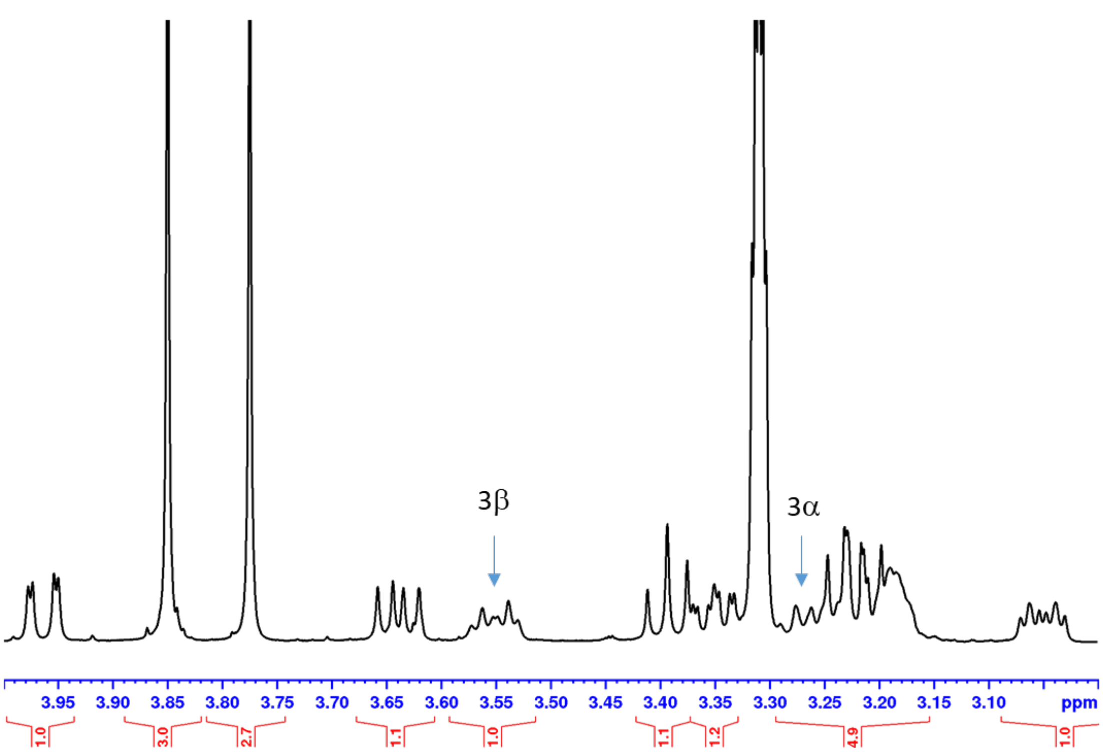

^{1}H NMR with water presaturation of d_2-9-OMe-strictosidine (3-4 ppm)

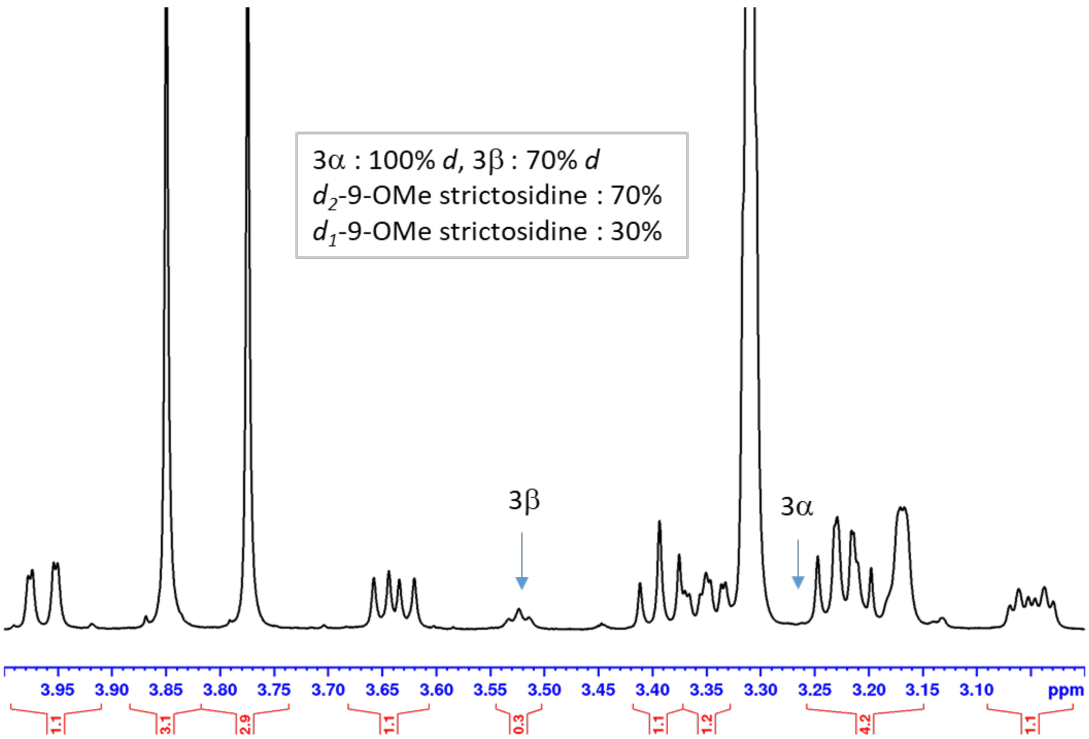

Supplementary Figure 53: NMR spectra for d_2-9-methoxystrictosidine.

NMR spectra for 20*S*-corynantheidine (**3a**)

^{1}H NMR full range in $CDCl_3$

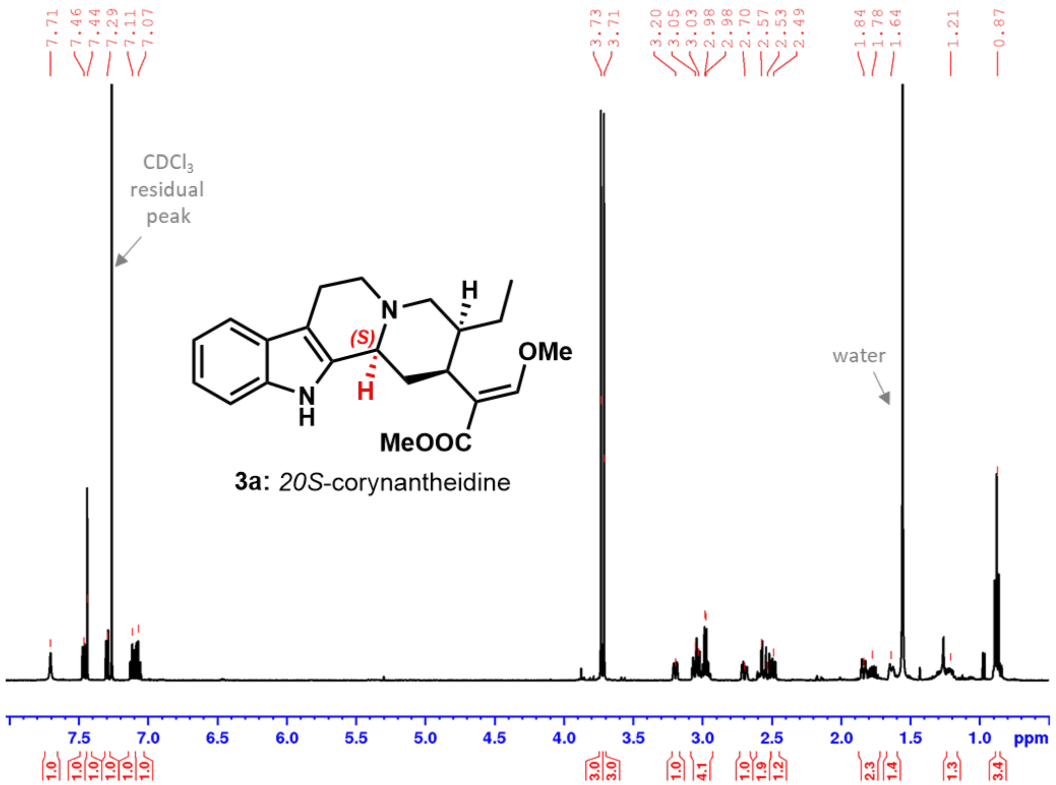

DEPTQ full range in $CDCl_3$

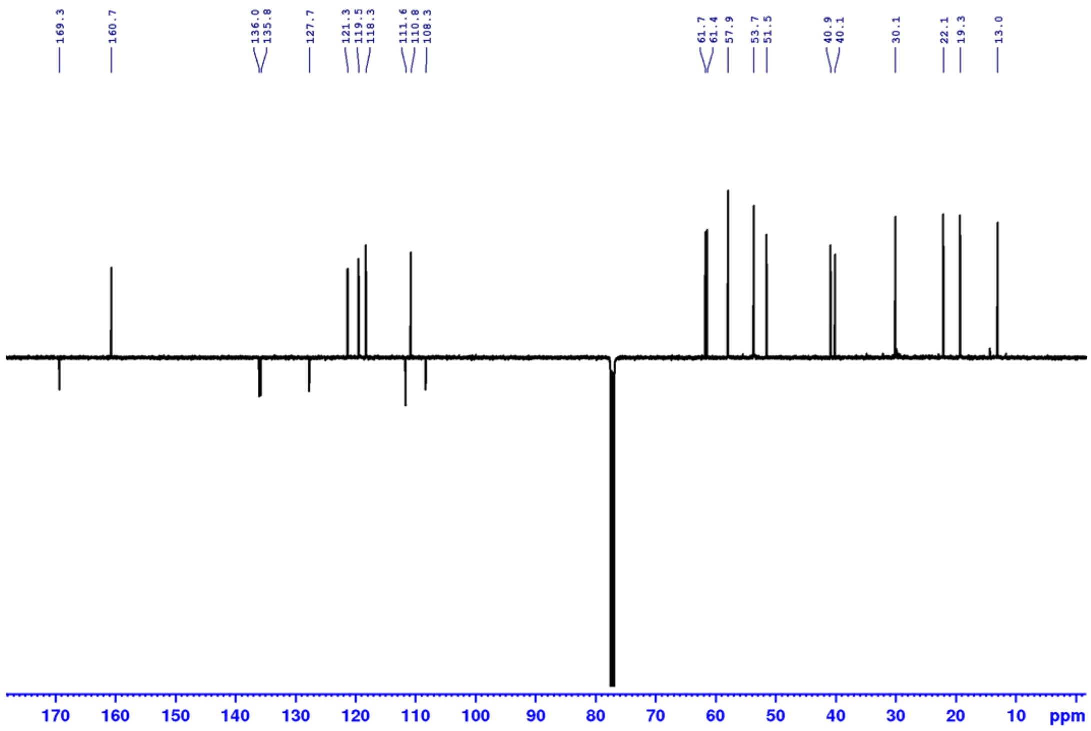

Supplementary Figure 54: NMR spectra for **3a:** (20*S*)-corynantheidine.

NMR spectra for 20*R*-corynantheidine (**3b**)

^{1}H NMR full range in $CDCl_3$

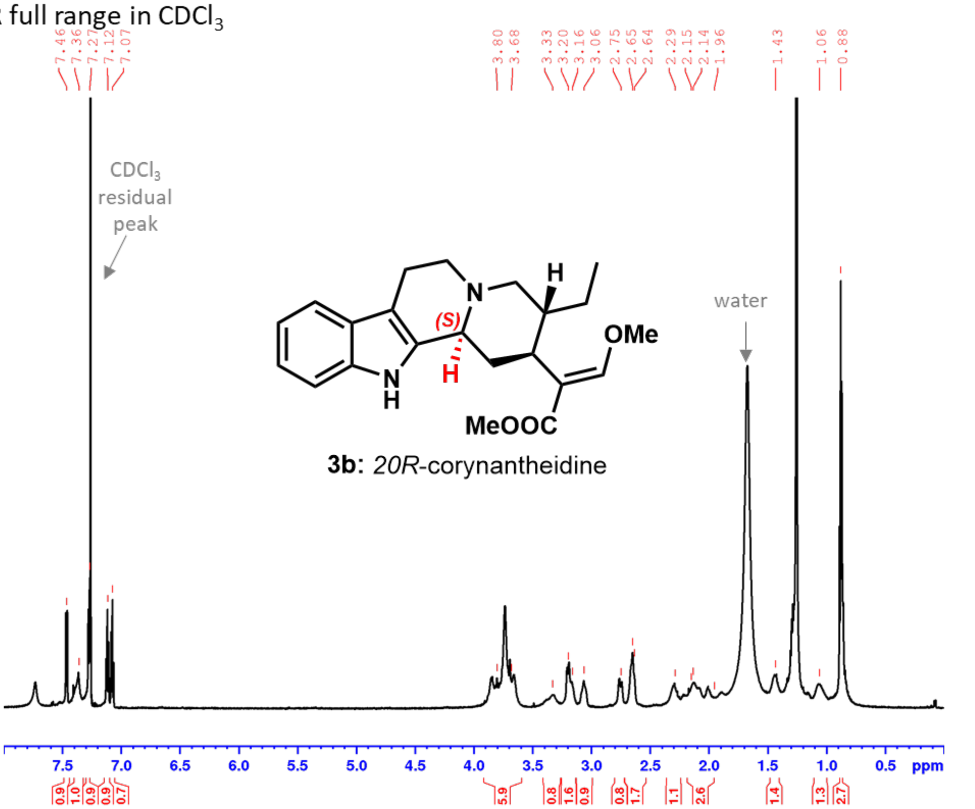

Due to low amount and broad signals only partial assignment was possible.

Phase sensitive HSQC (blue: CH, CH_3, green CH_2)/ HMBC (red) full range in $CDCl_3$

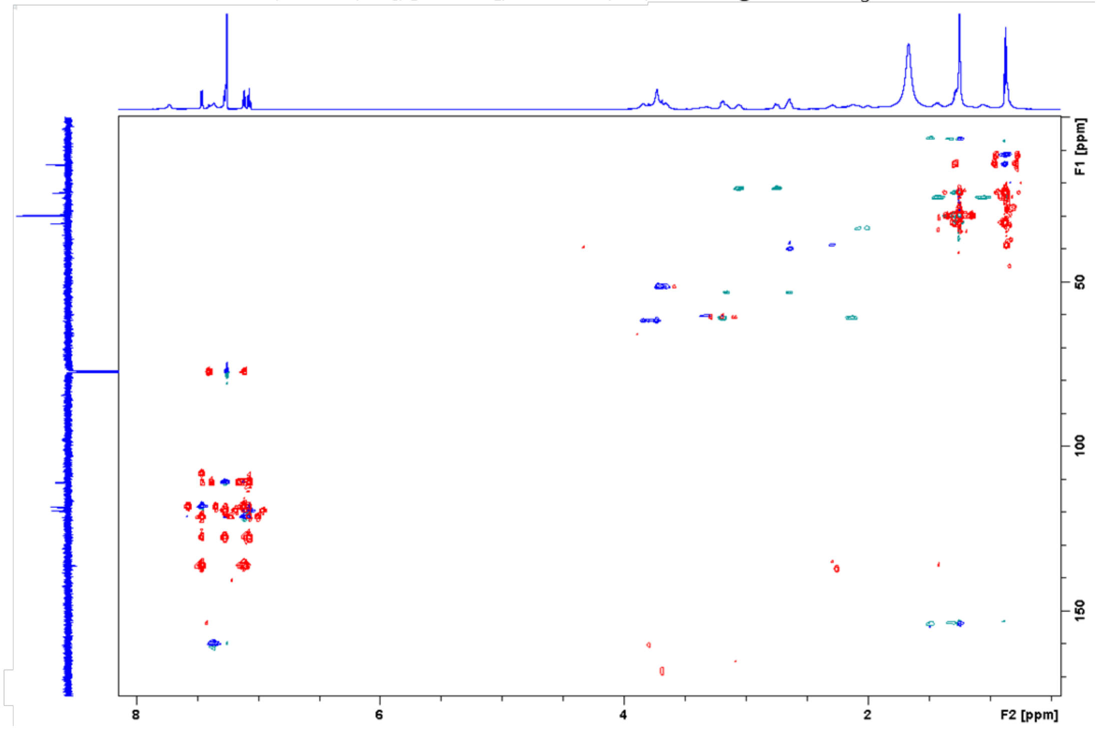

Supplementary Figure 55: NMR spectra for **3b: (**20*R*)-corynantheidine.

NMR spectra for 20*S*-isocorynantheidine (**6a**)

^{1}H NMR full range in $CDCl_3$

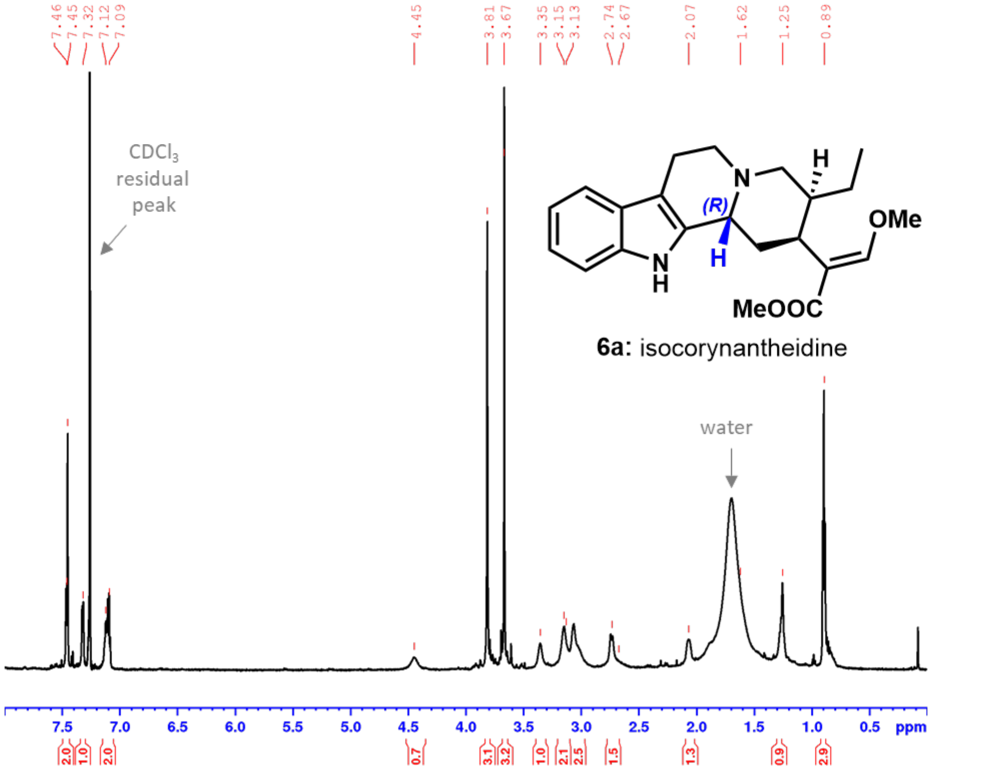

Phase sensitive HSQC (blue: CH, CH_3, green CH_2)/ HMBC (red) full range in $CDCl_3$

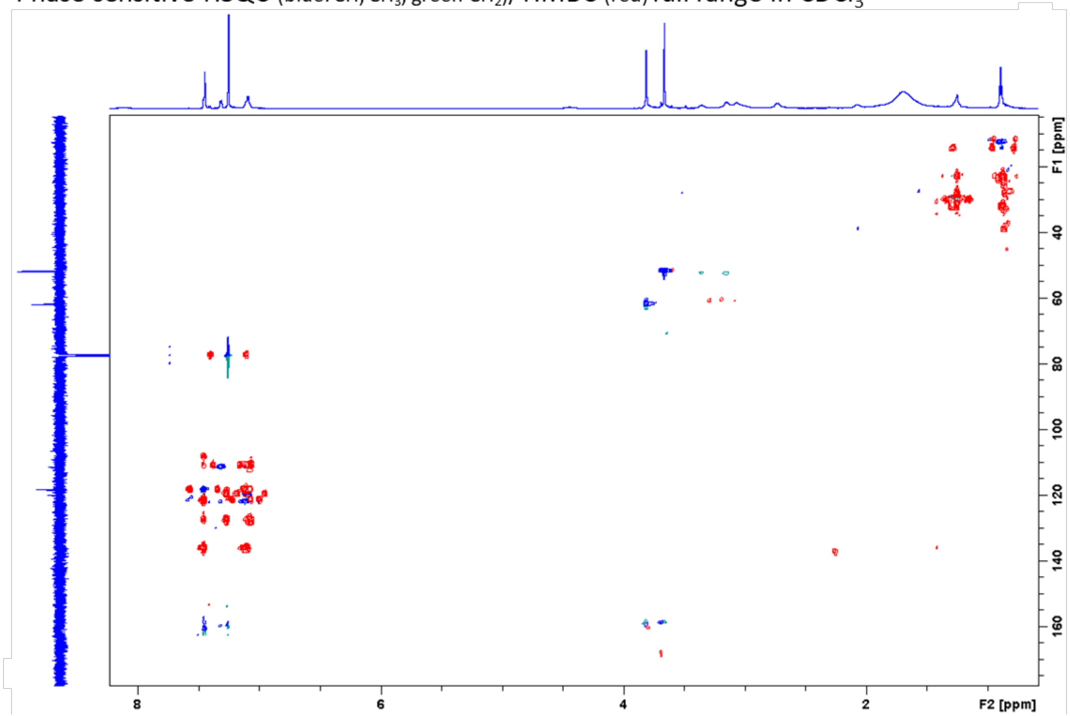

Supplementary Figure 56: NMR spectra for **6a:** (20*S*)-isocorynantheidine.

NMR spectra for hirsutine (**6b**)

^{1}H NMR full range in $CDCl_3$

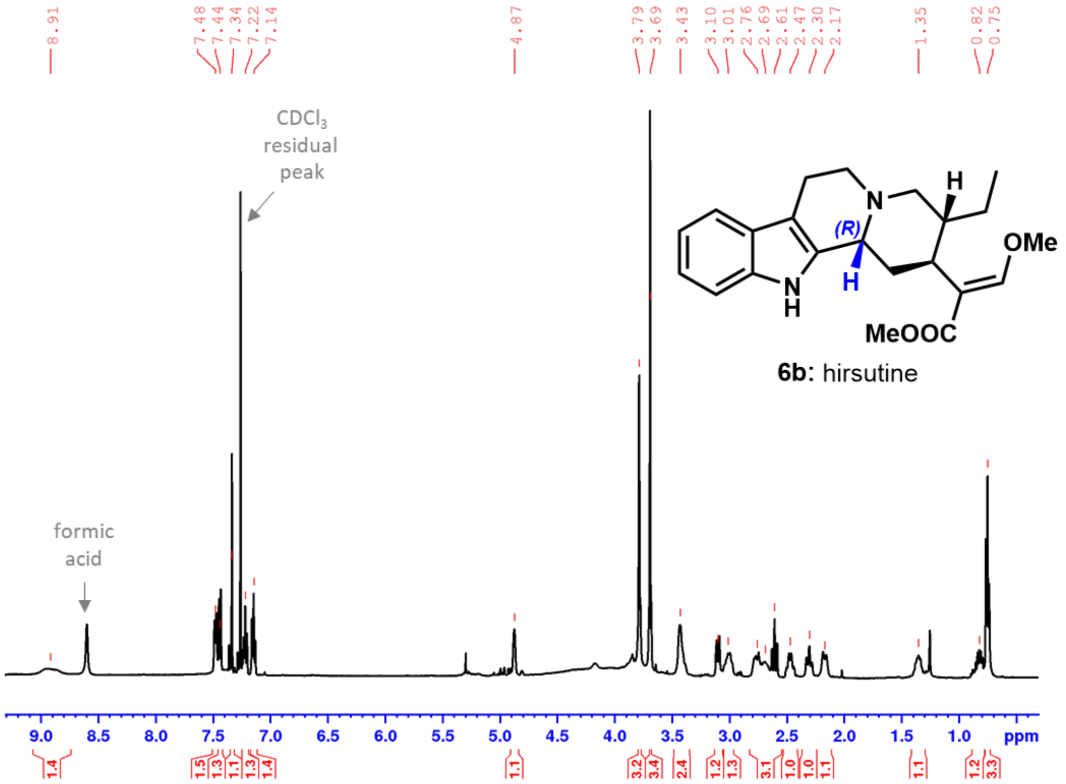

DEPTQ full range in $CDCl_3$

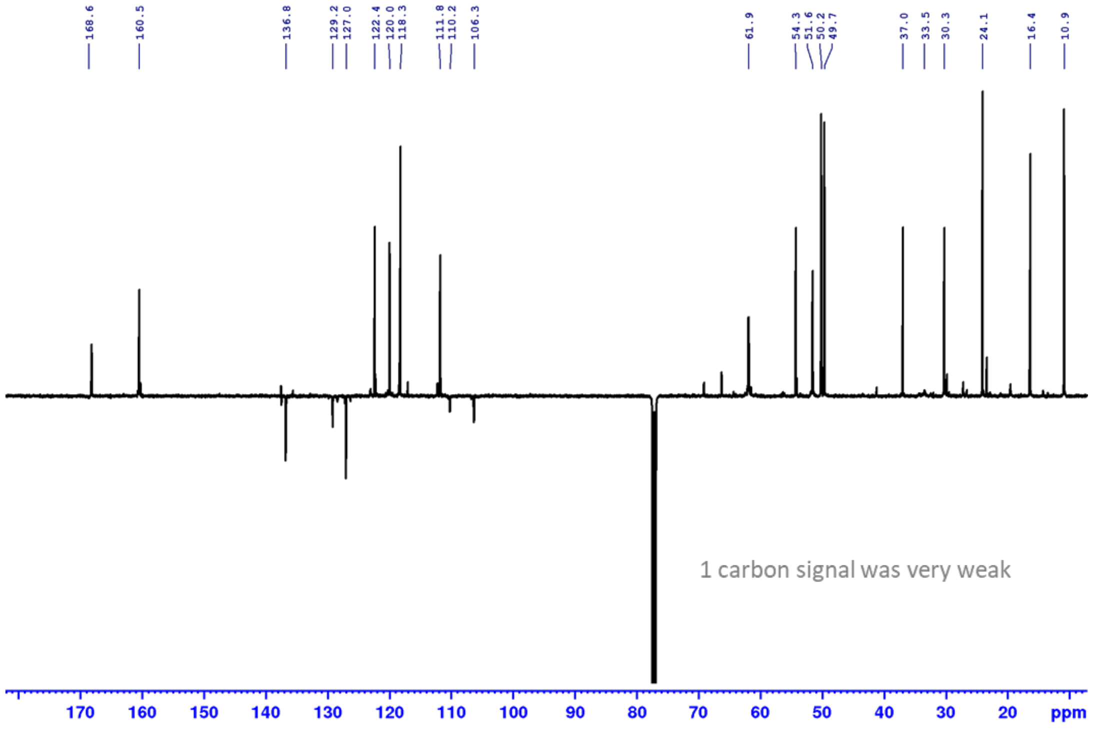

Supplementary Figure 57: NMR spectra for **6b:** hirsutine.

NMR spectra for mitragynine (**5a**)

^{1}H NMR full range in $CDCl_3$

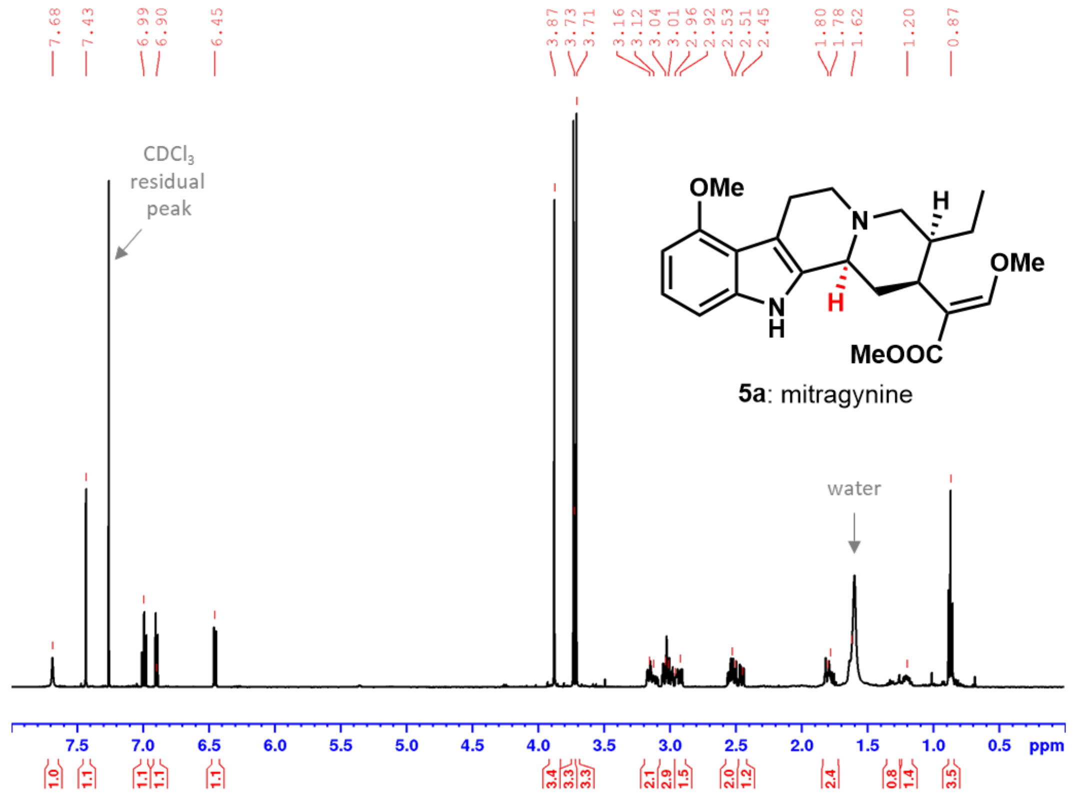

DEPTQ full range in $CDCl_3$

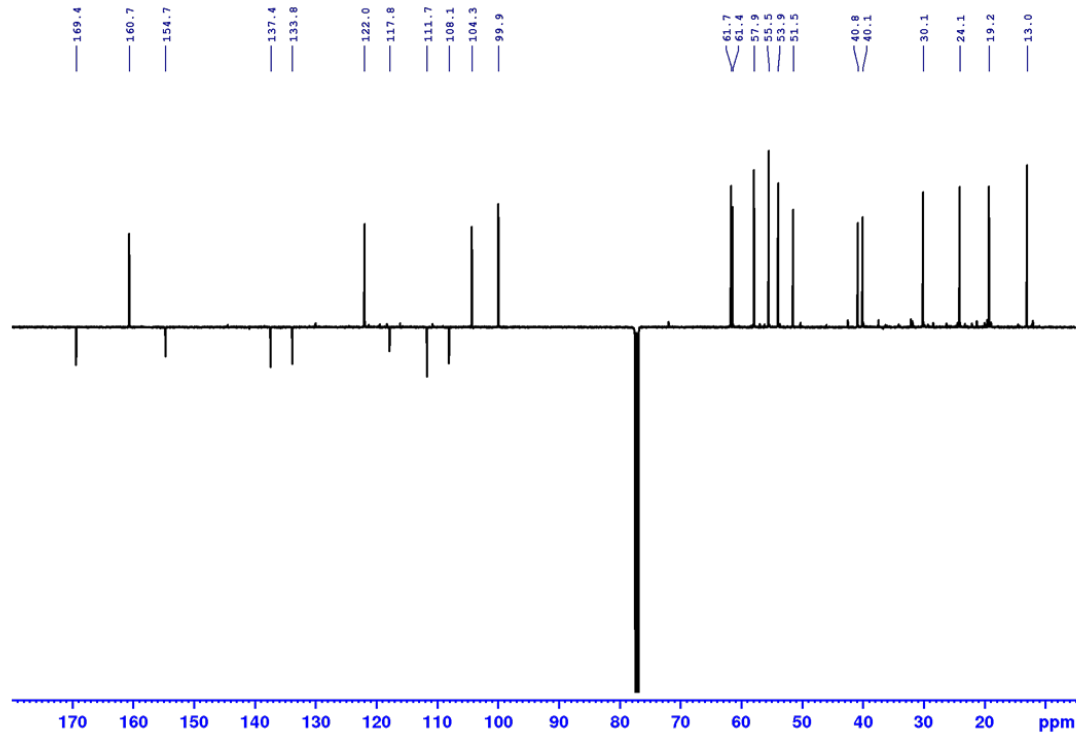

Supplementary Figure 58: NMR spectra for **5a:** mitragynine.

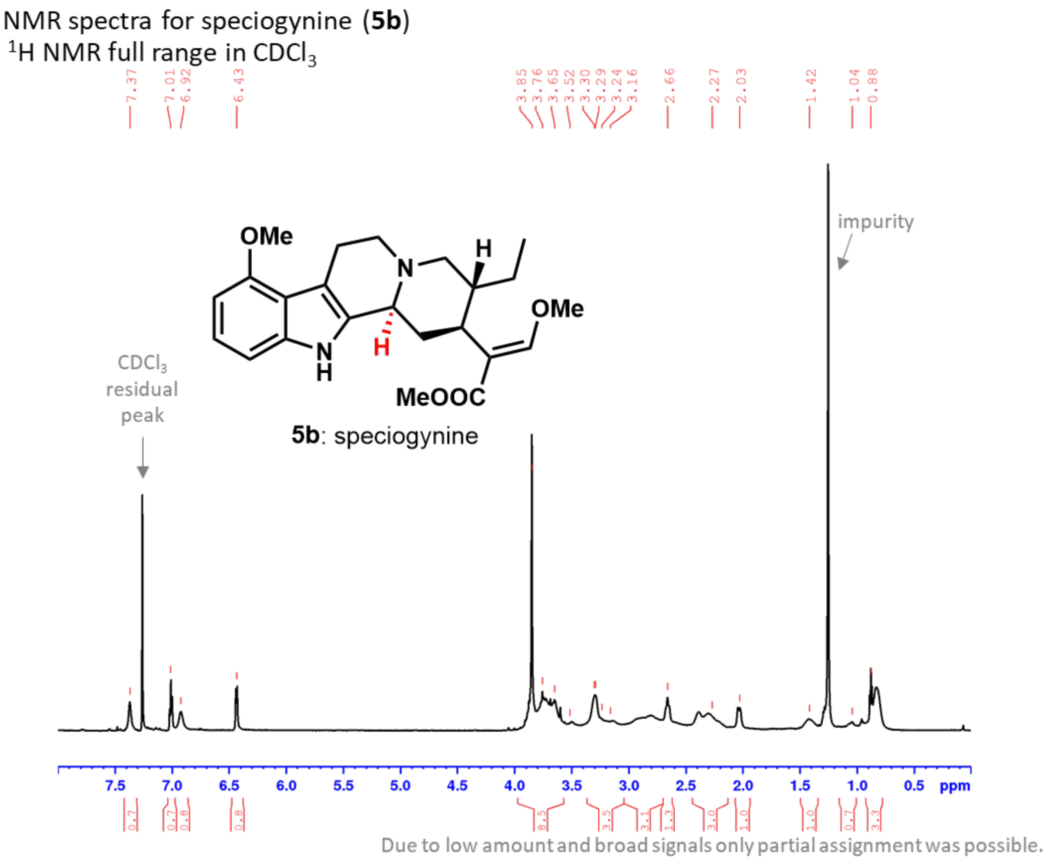

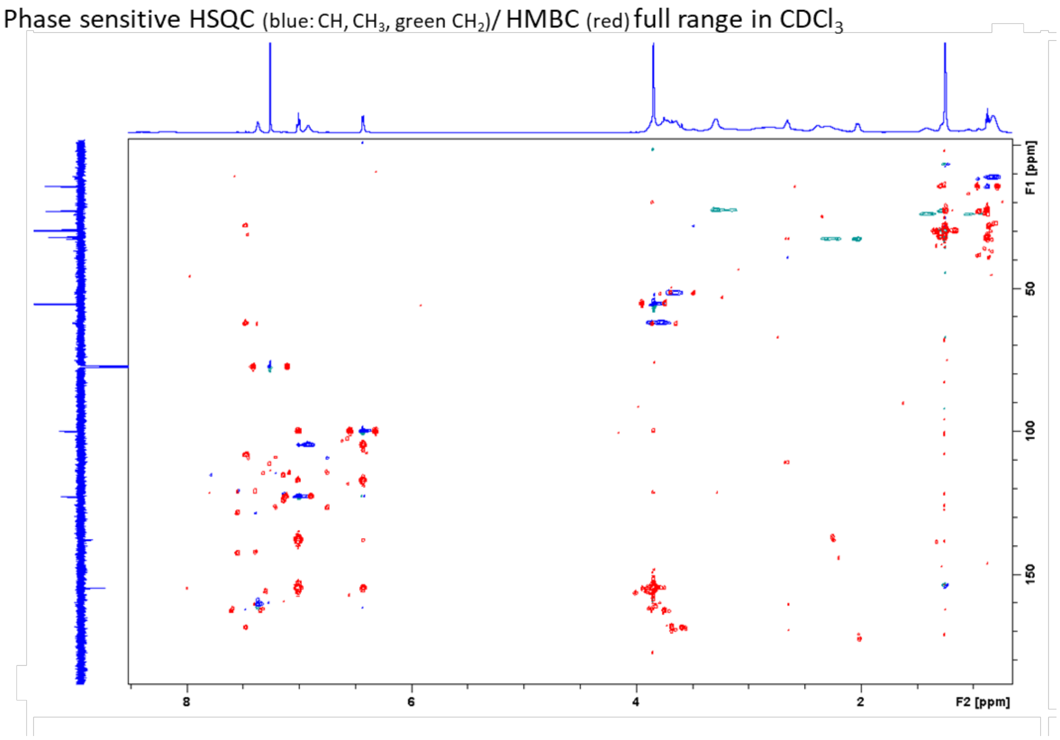

Supplementary Figure 59: NMR spectra for **5b:** speciogynine.

NMR spectra for speciociliatine (**7a**)

^{1}H NMR full range in $CDCl_3$

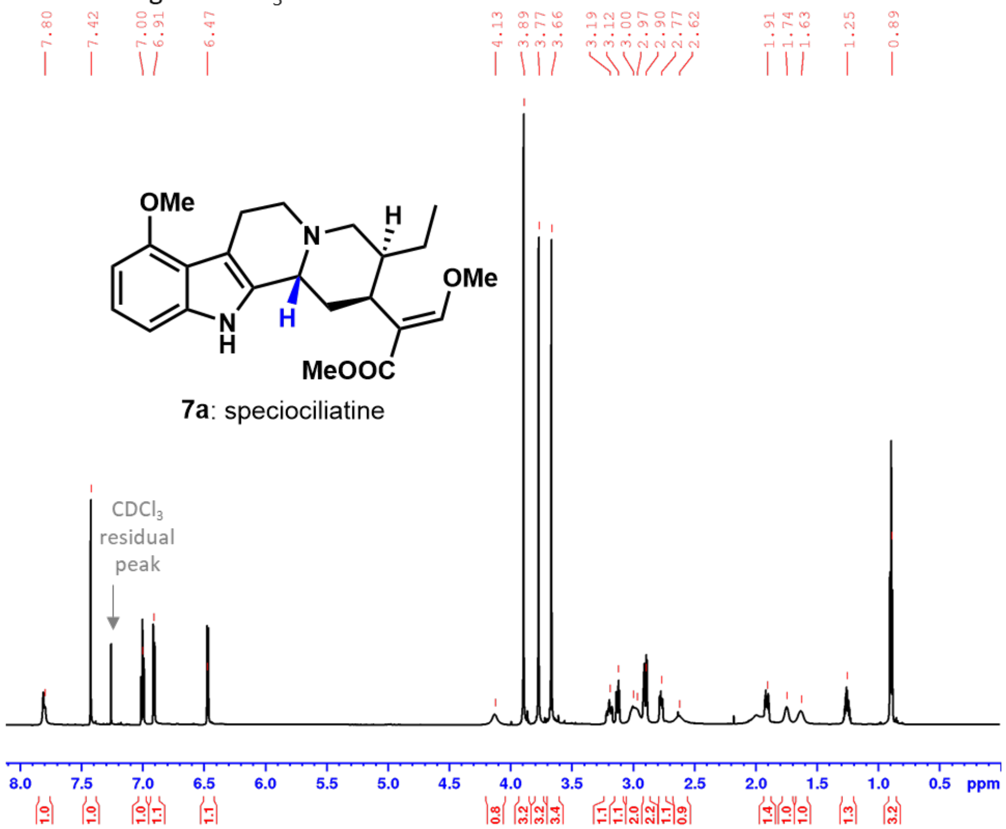

Phase sensitive HSQC (blue: CH, CH_3, green CH_2)/ HMBC (red) full range in $CDCl_3$

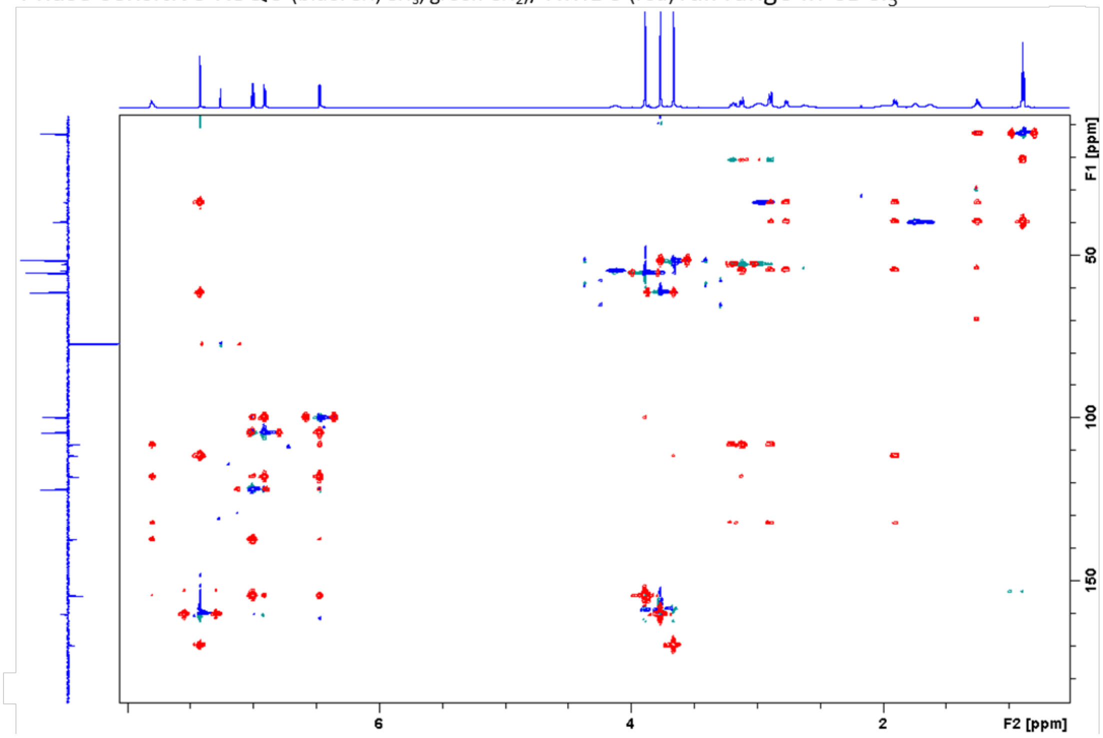

Supplementary Figure 60: NMR spectra for **7a:** speciociliatine.

NMR spectra for mitraciliatine (**7b**)

^{1}H NMR full range in $CDCl_3$

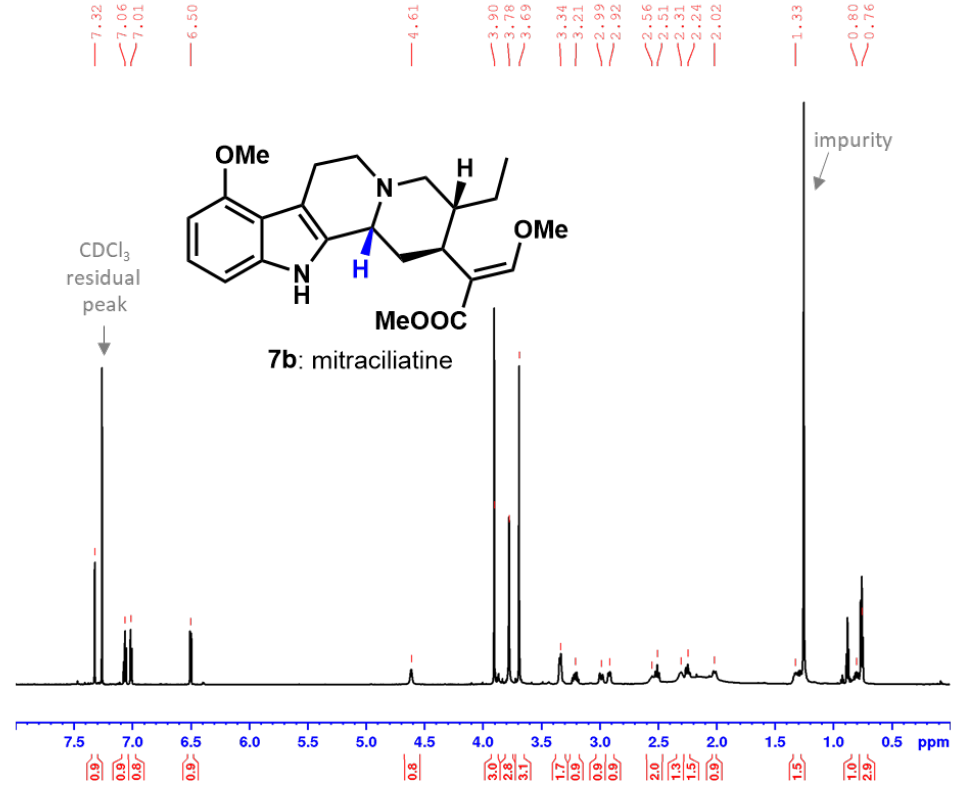

Phase sensitive HSQC (blue: CH, CH_3, green CH_2)/ HMBC (red) full range in $CDCl_3$

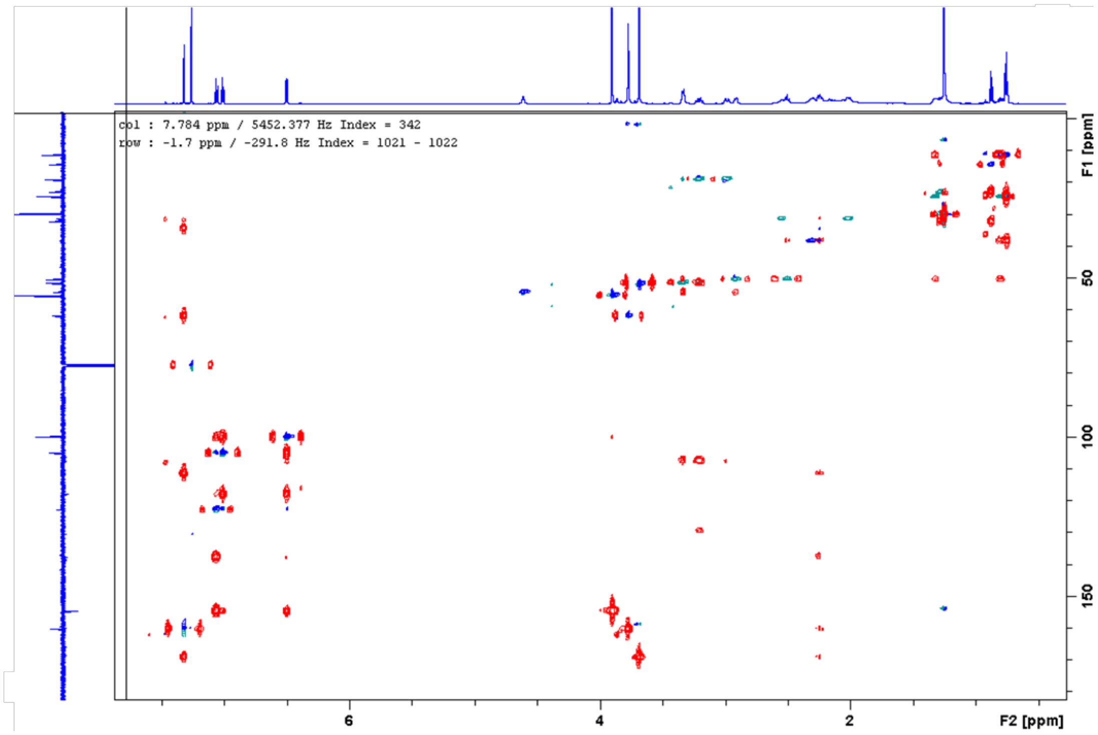

Supplementary Figure 61: NMR spectra for **7b:** mitraciliatine.

NMR spectra for dehydromitragynine, TFA salt (**14a**)

^{1}H NMR full range in $CDCl_3$

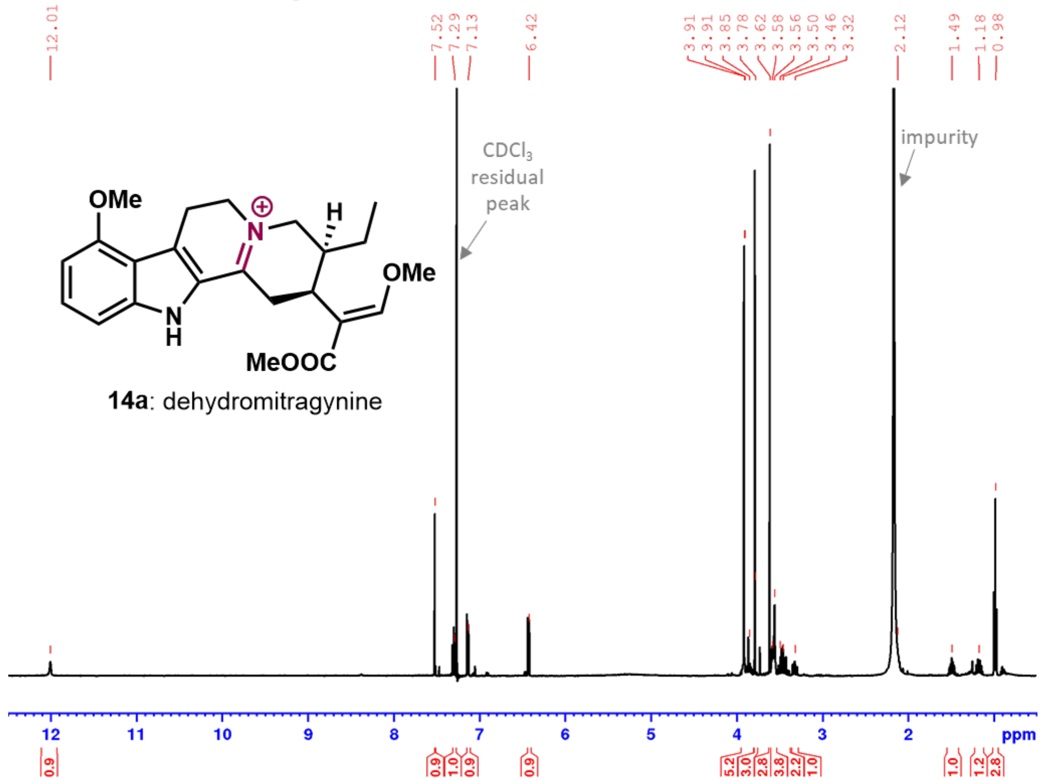

DEPTQ full range in $CDCl_3$

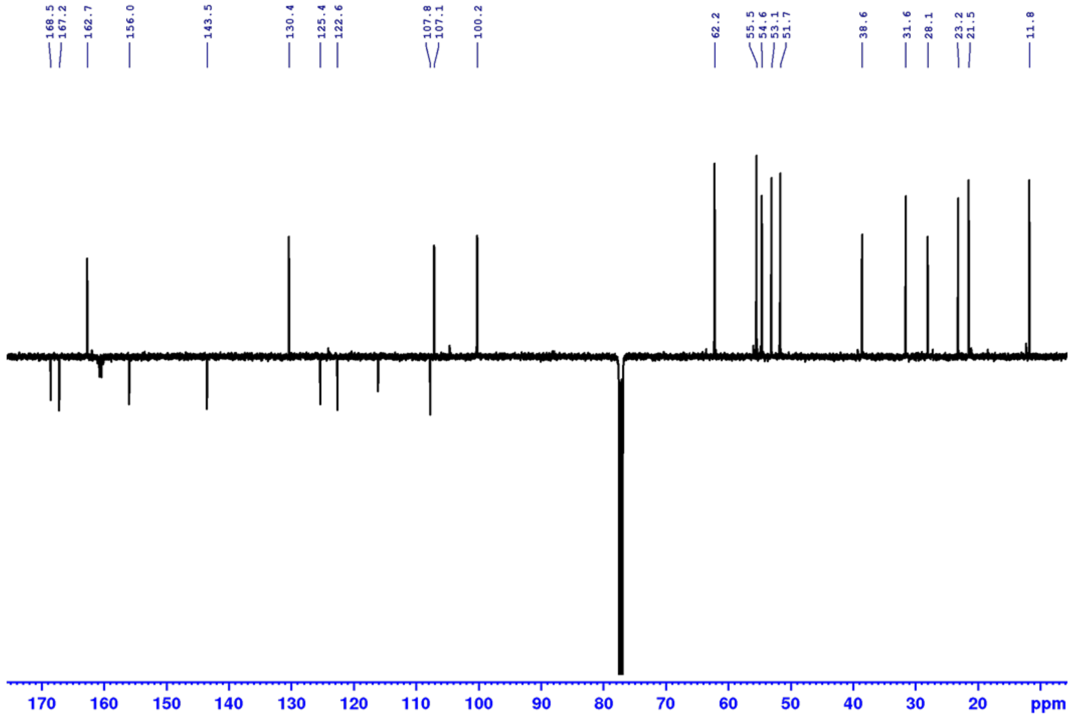

Supplementary Figure 62: NMR spectra for **14a:** 3-dehydromitragynine (DHM).

^{1}H NMR full range in $CDCl_3$

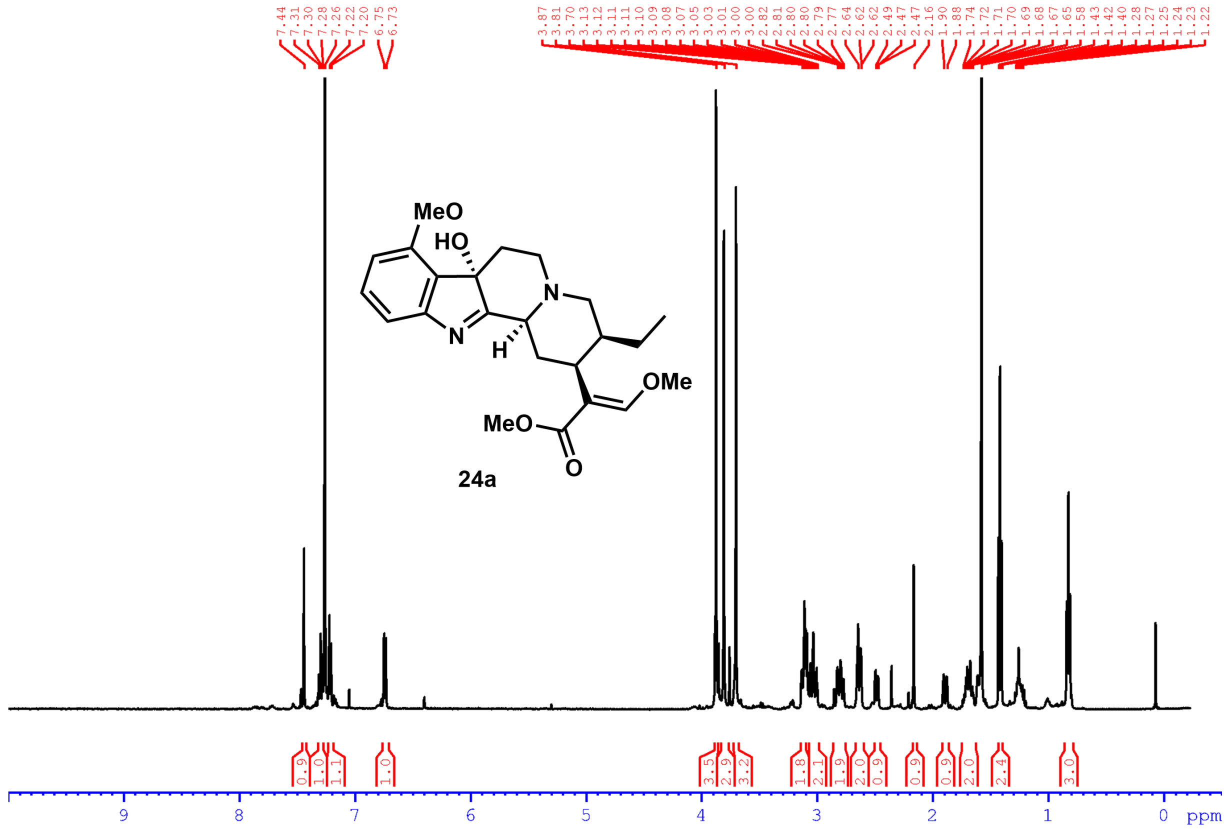

Supplementary Figure 63: ^{1}H NMR spectrum (500 MHz, $CDCl_3$) for 7-hydroxymitragynine (**24a**).

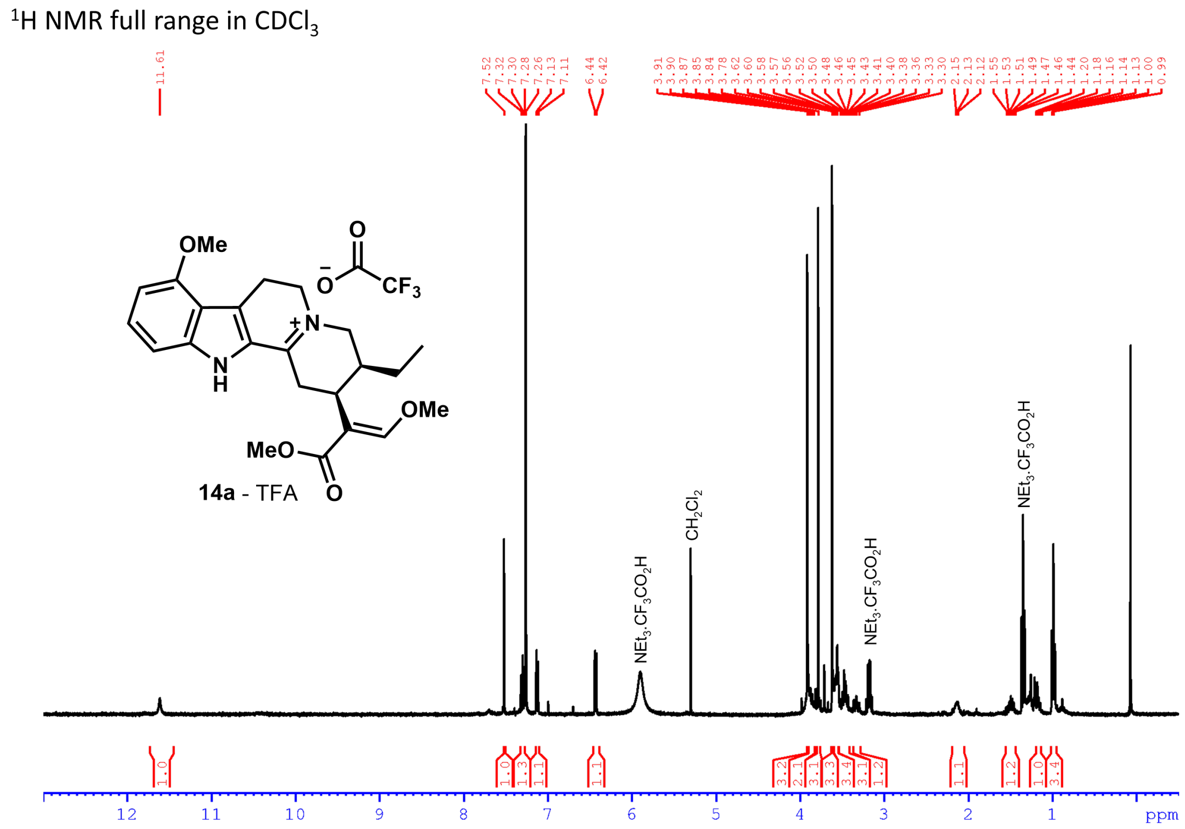

Supplementary Figure 64: ^{1}H NMR spectrum (400 MHz, $CDCl_3$) for DHM-TFA (**14a**).

NMR spectra for 20*S*-dehydrocorynantheidine (**13a**)

^1H NMR full range in $CDCl_3$

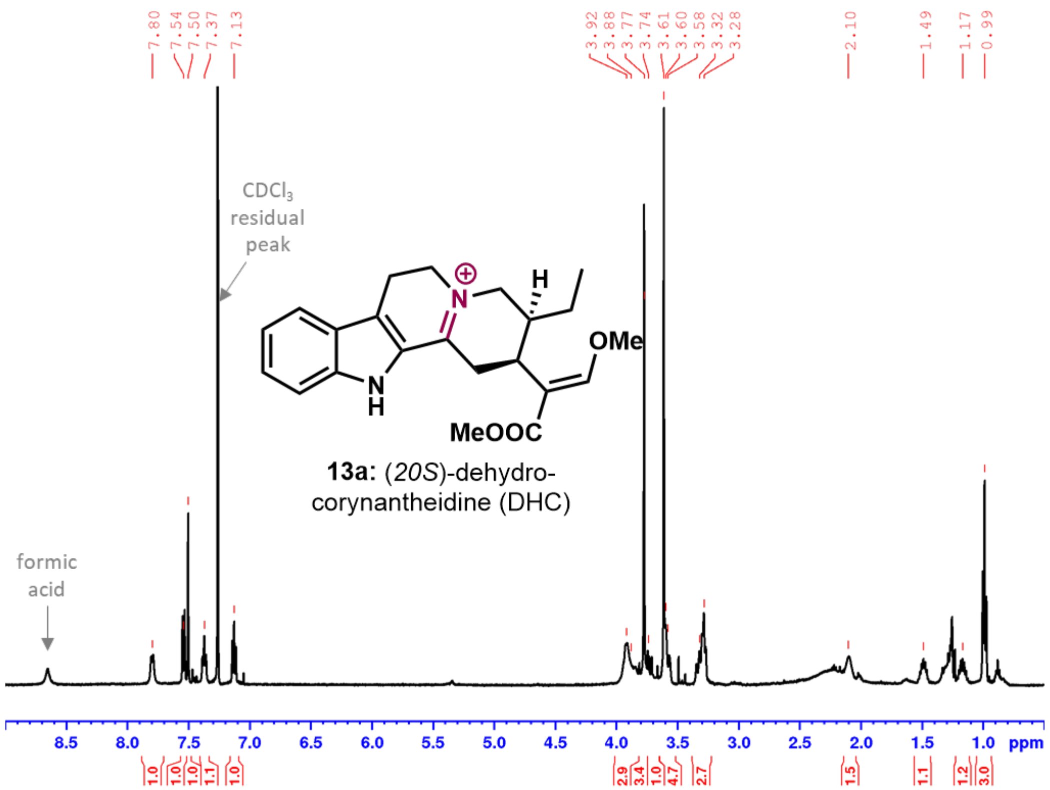

DEPTQ full range in $CDCl_3$

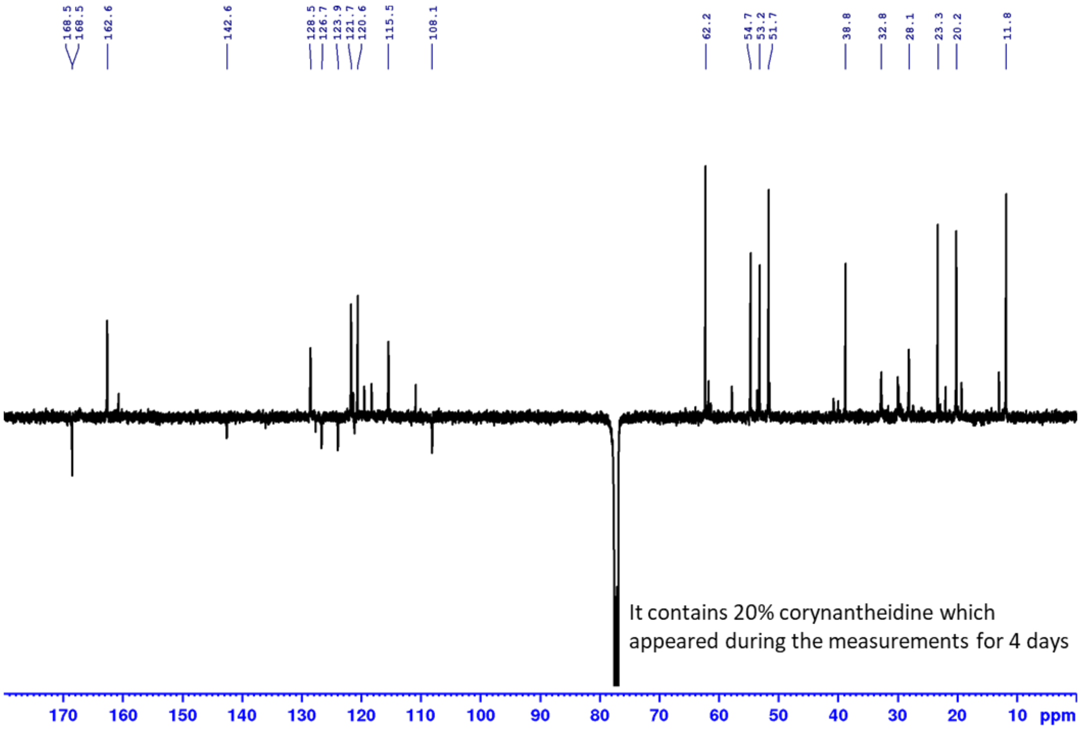

Supplementary Figure 65: NMR spectra for **13a:** (20*S*)-3-dehydrocorynantheidine (DHC).

NMR spectra for 20*S*-dehydrocorynantheidine (**13a**)

Phase sensitive HSQC (black: CH, CH_3, red: CH_2) full range

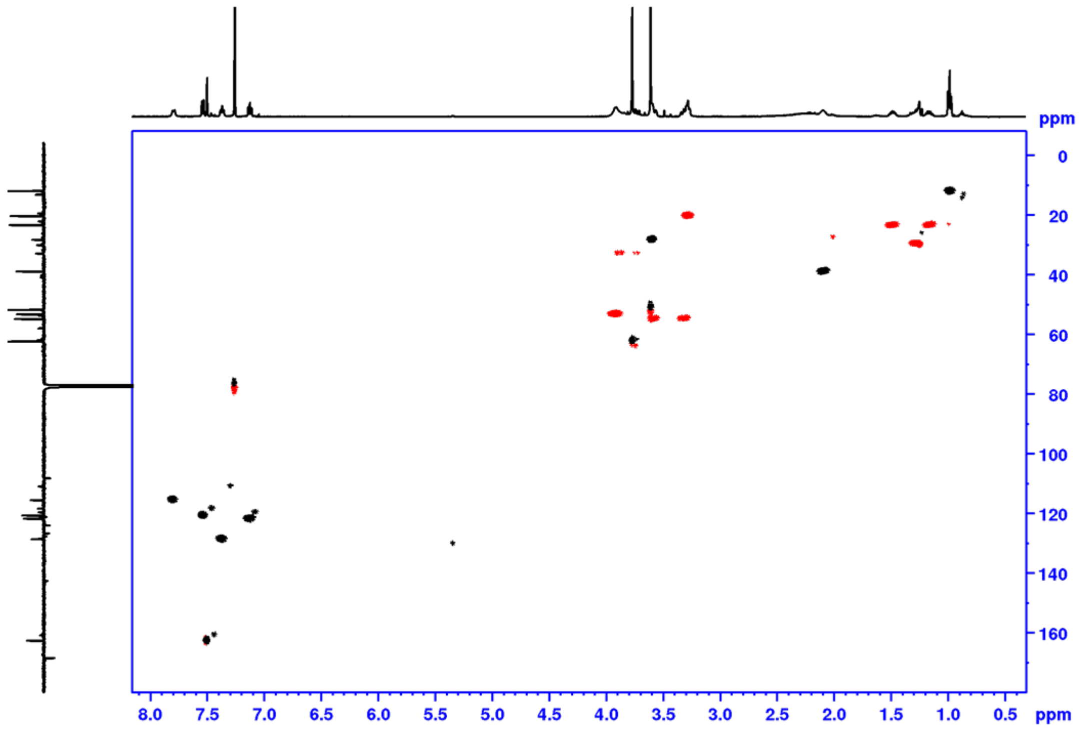

Phase sensitive HSQC (black: CH, CH_3, red: CH_2) aliphatic range

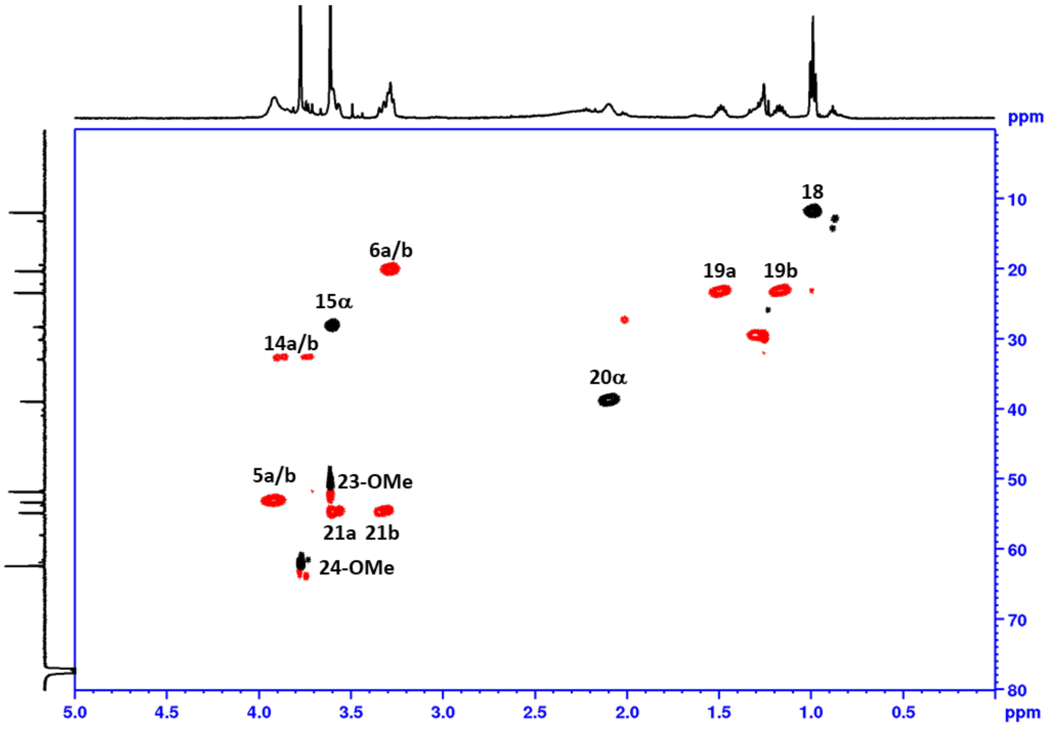

Supplementary Figure 66: NMR spectra for **13a:** (20*S*)-3-dehydrocorynantheidine (DHC).

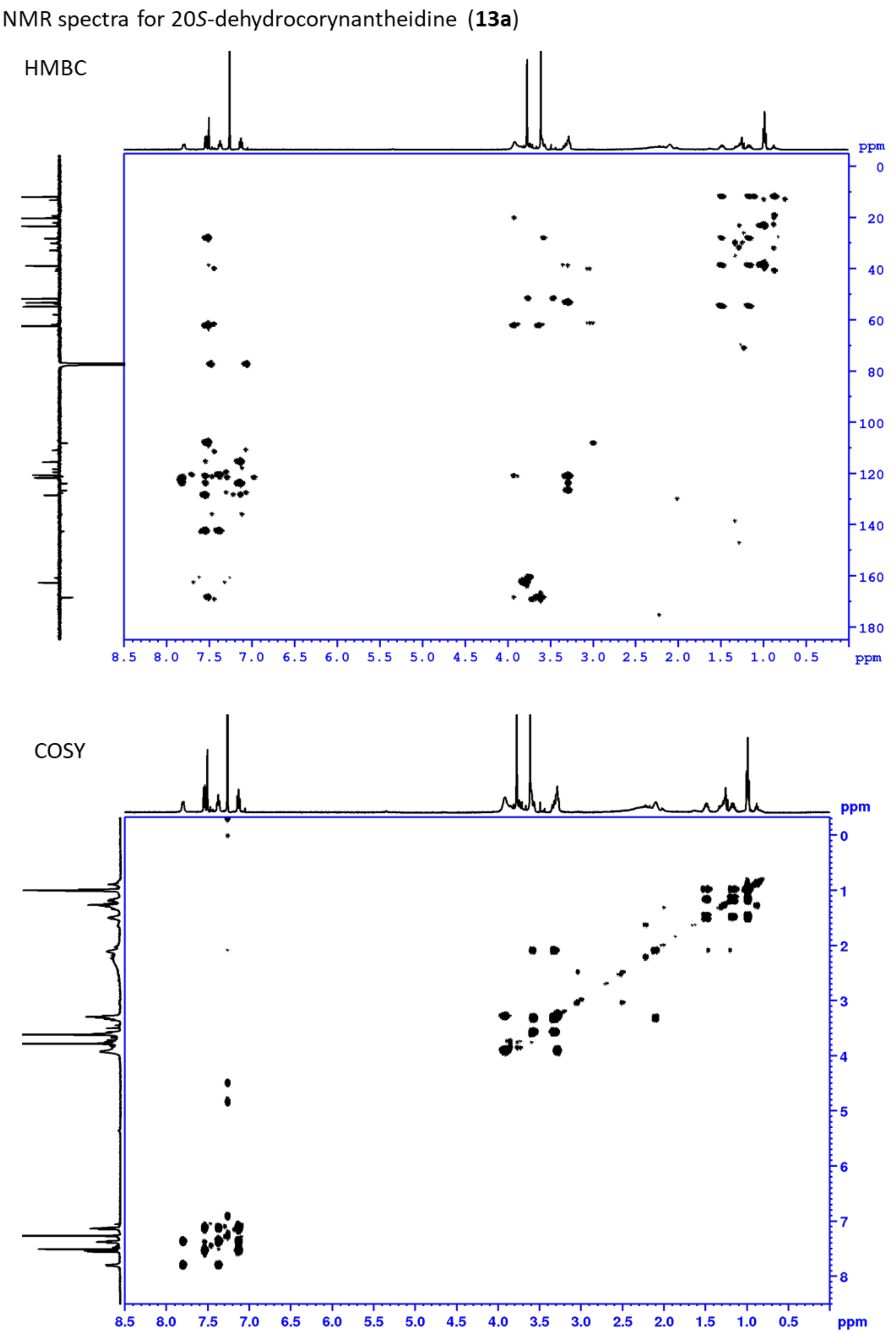

Supplementary Figure 67: NMR spectra for **13a:** (20*S*)-3-dehydrocorynantheidine (DHC).

^{1}H NMR

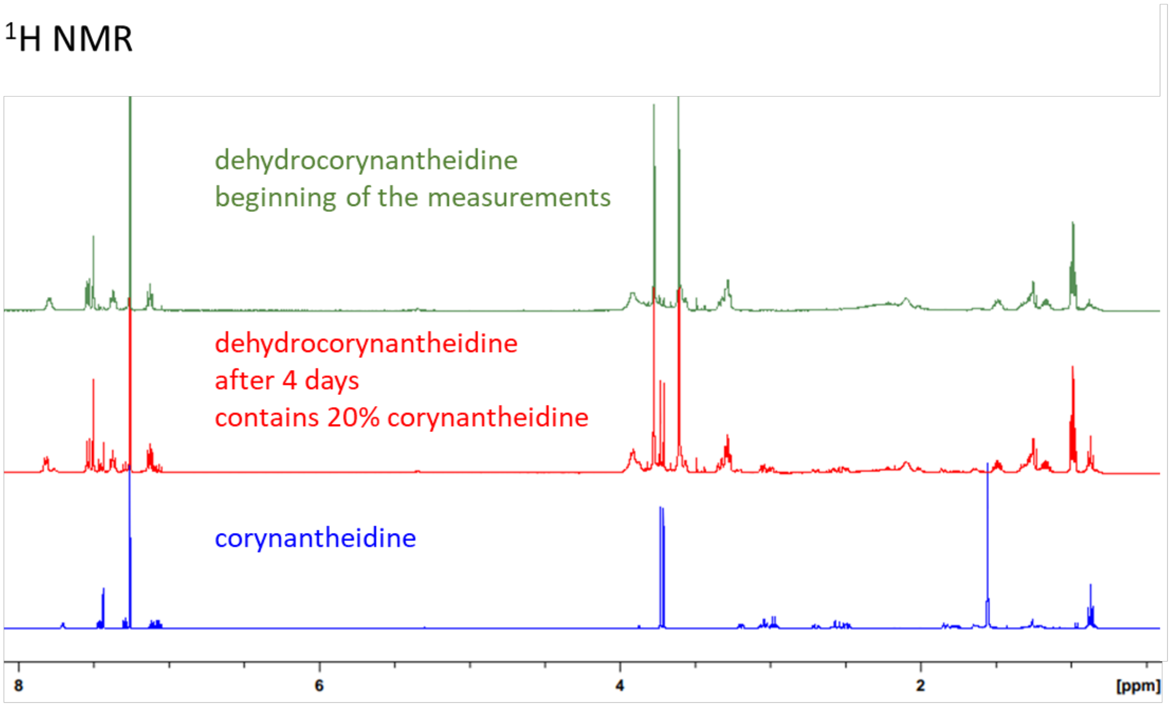

^{13}C NMR (DEPTQ)

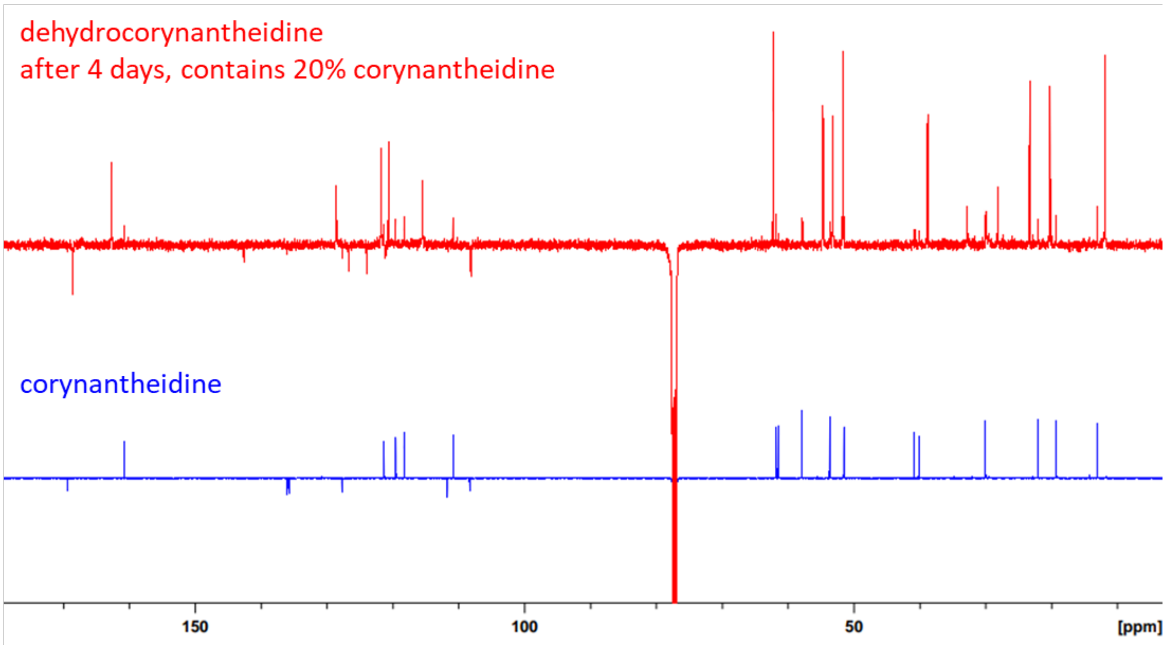

Supplementary Figure 68: NMR spectra for **13a:** (20*S*)-3-dehydrocorynantheidine (DHC) and **3a**: (20*S*)-corynantheidine. Some conversion of **13a** to **3a** was observed during NMR analysis.

NMR spectra for 3-iso-ajmalicine (**11b**)

^{1}H NMR full range in $CDCl_3$

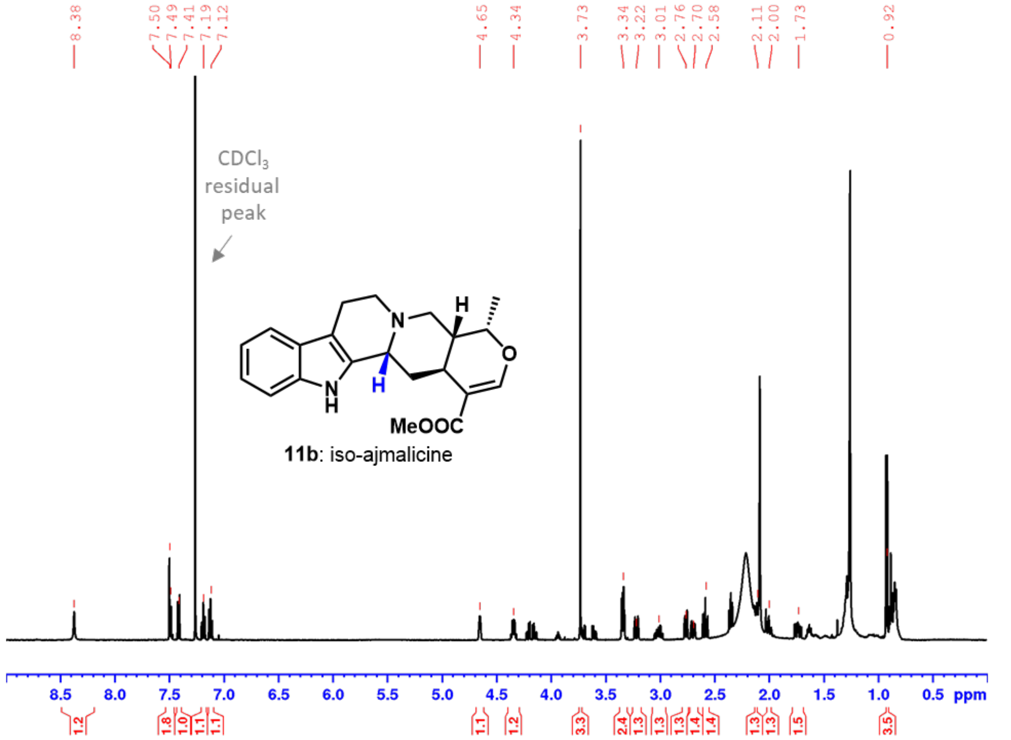

Not picked/integrated peaks correspond to impurities

DEPTQ full range in $CDCl_3$

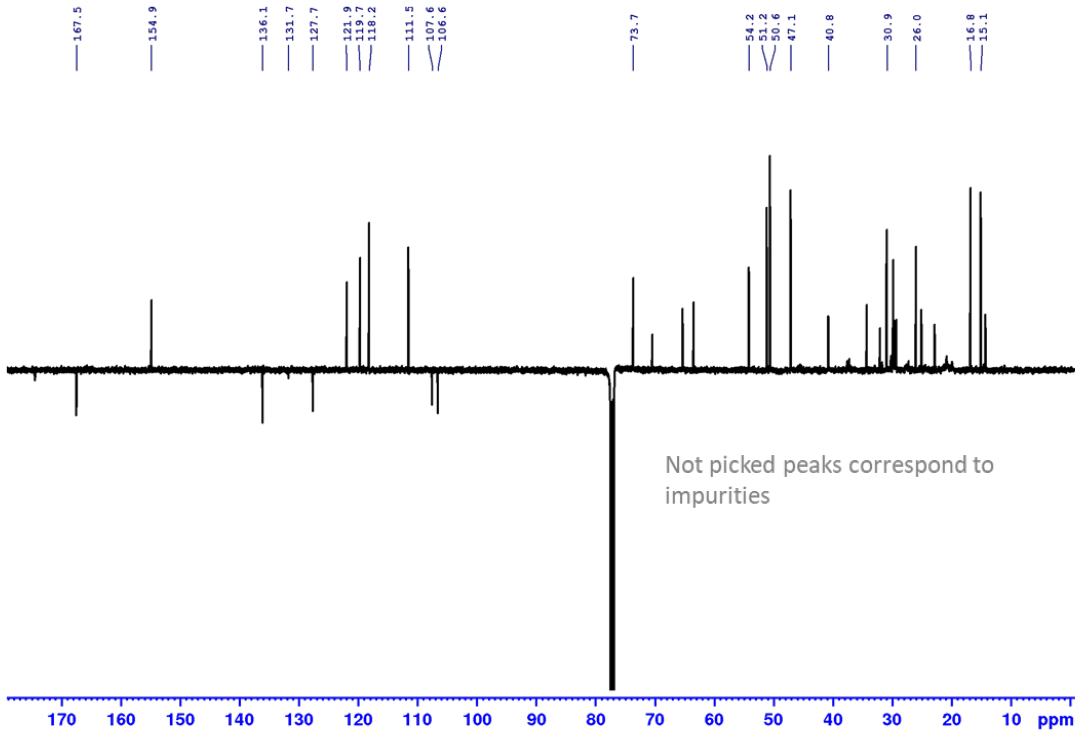

Supplementary Figure 69: NMR spectra for **11b:** isoajmalicine.

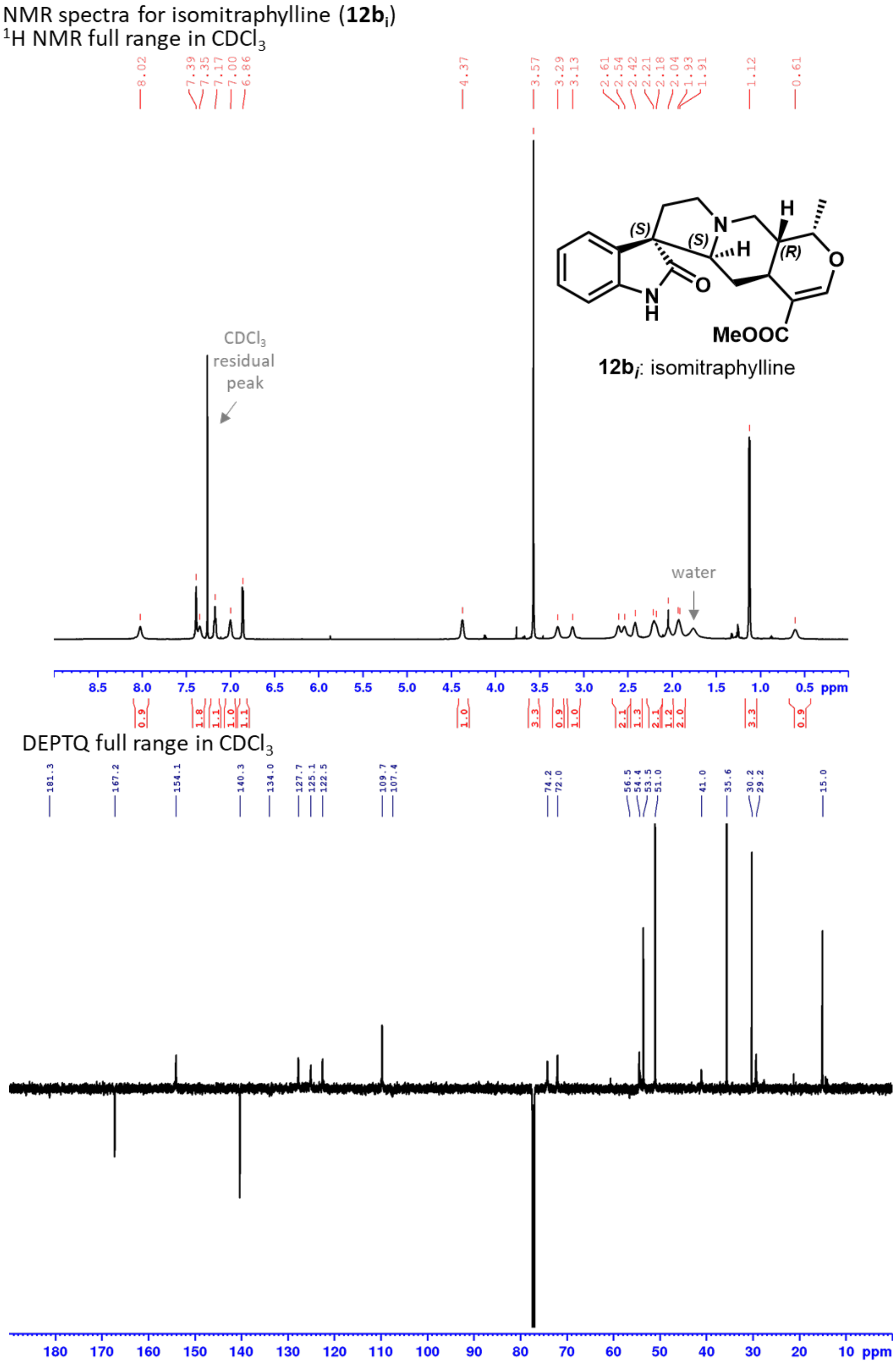

Supplementary Figure 70: NMR spectra for **12b$_i$:** isomitraphylline.

NMR spectra for isocorynoxeine (**8c$_i$**)

^1H NMR full range in $CDCl_3$

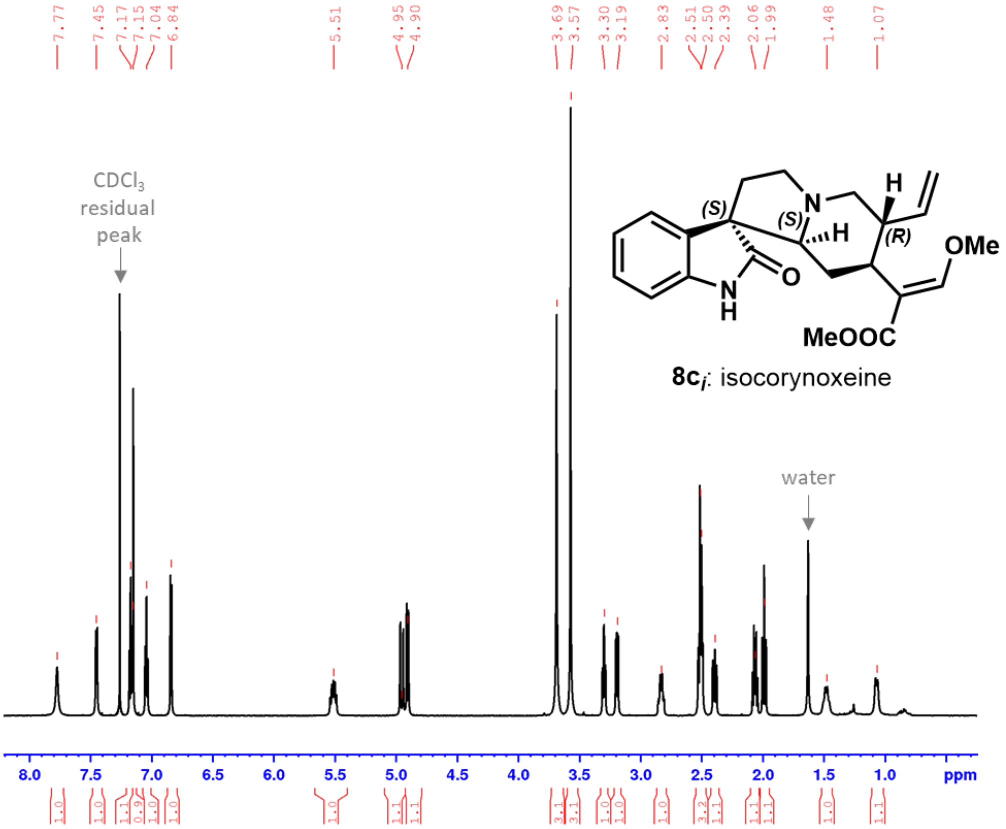

DEPTQ full range in $CDCl_3$

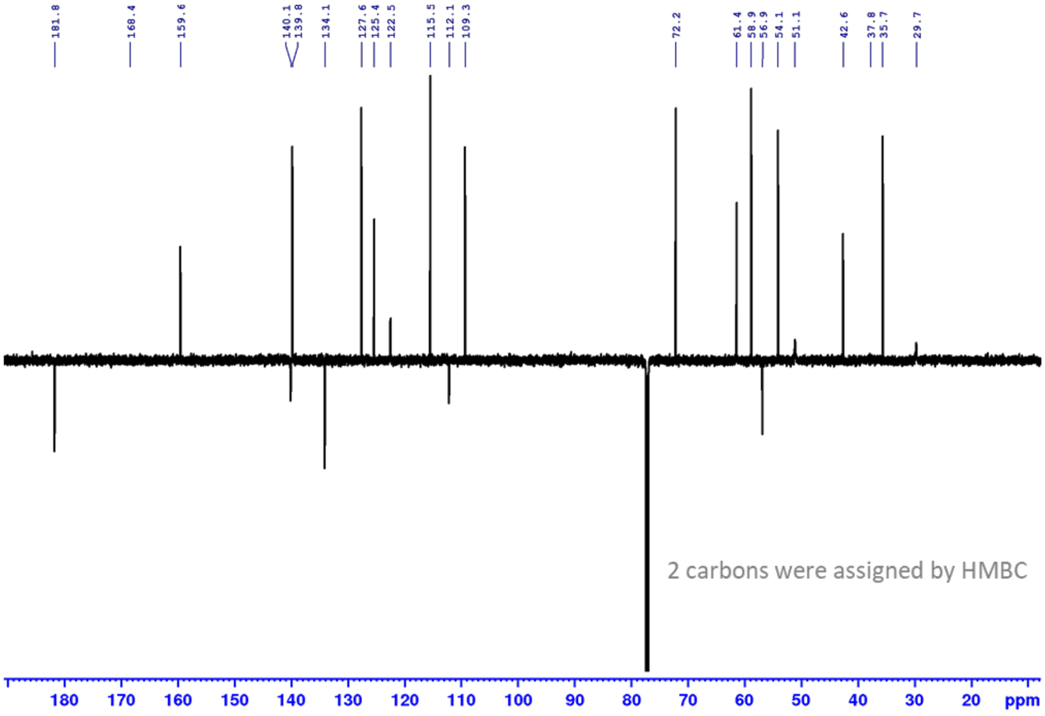

Supplementary Figure 71: NMR spectra for **8c$_i$:** isocorynoxeine.

NMR spectra for corynoxeine ($\mathbf{8c_{ii}}$)

^{1}H NMR full range in $CDCl_3$

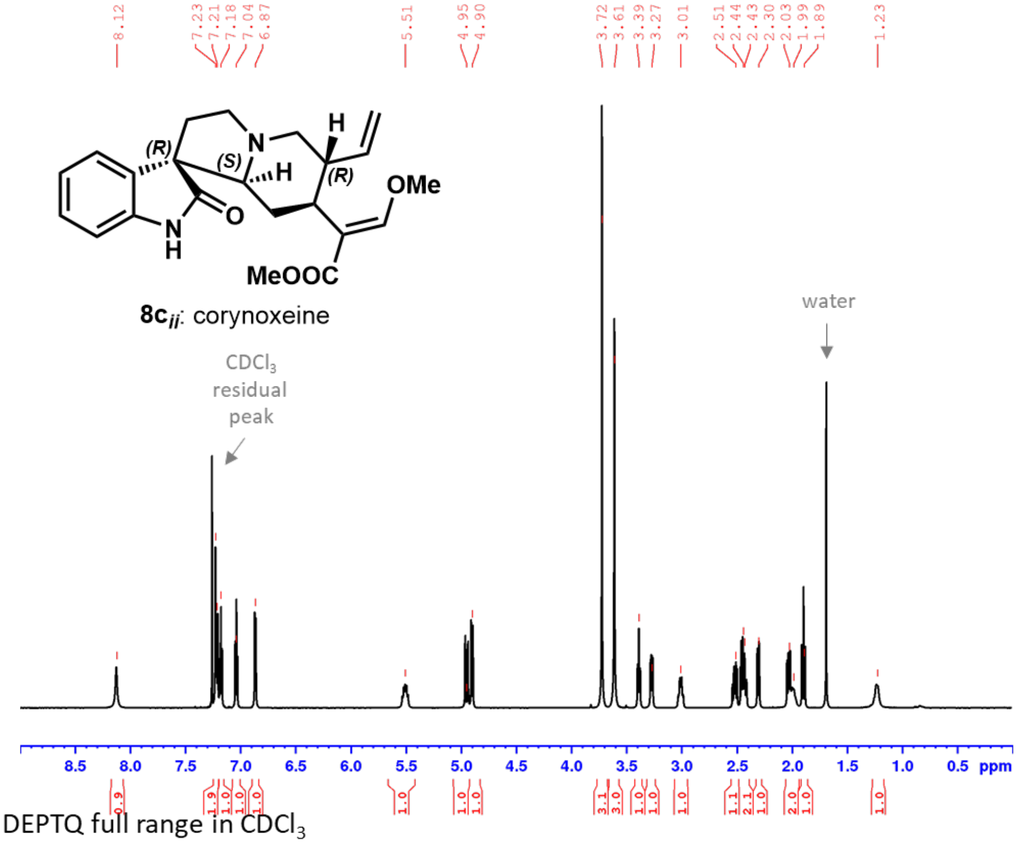

DEPTQ full range in $CDCl_3$

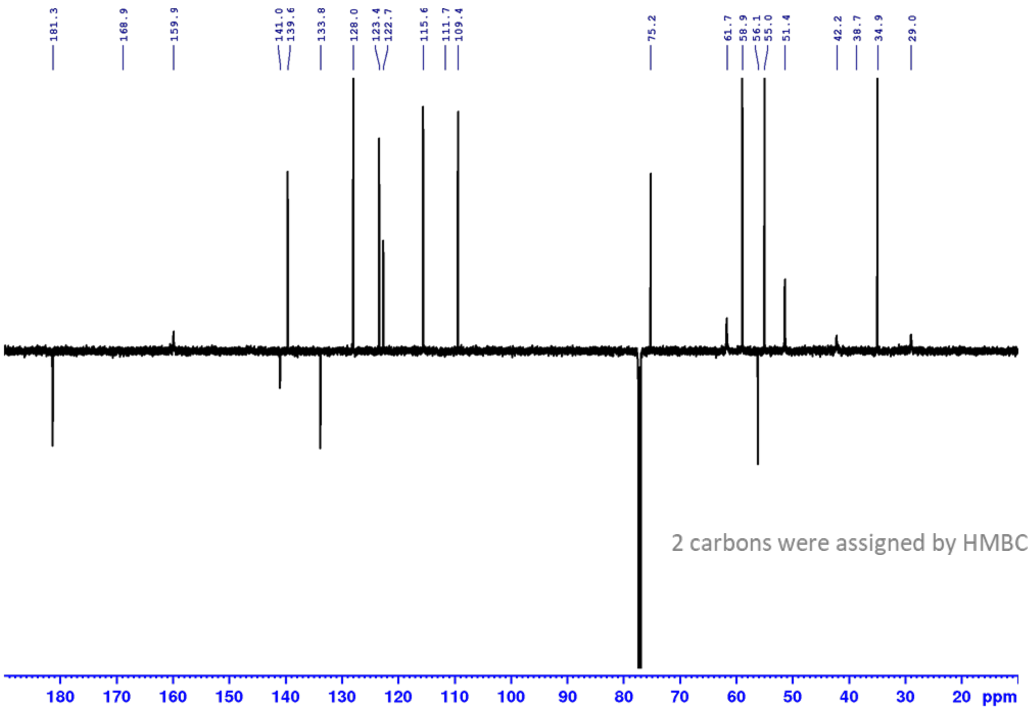

Supplementary Figure 72: NMR spectra for $\mathbf{8c_{ii}}$**:** corynoxeine.

NMR spectra for 20S-10-hydroxycorynantheidine (**16a**)

^{1}H NMR with water presaturation, full range in MeOH-d_3

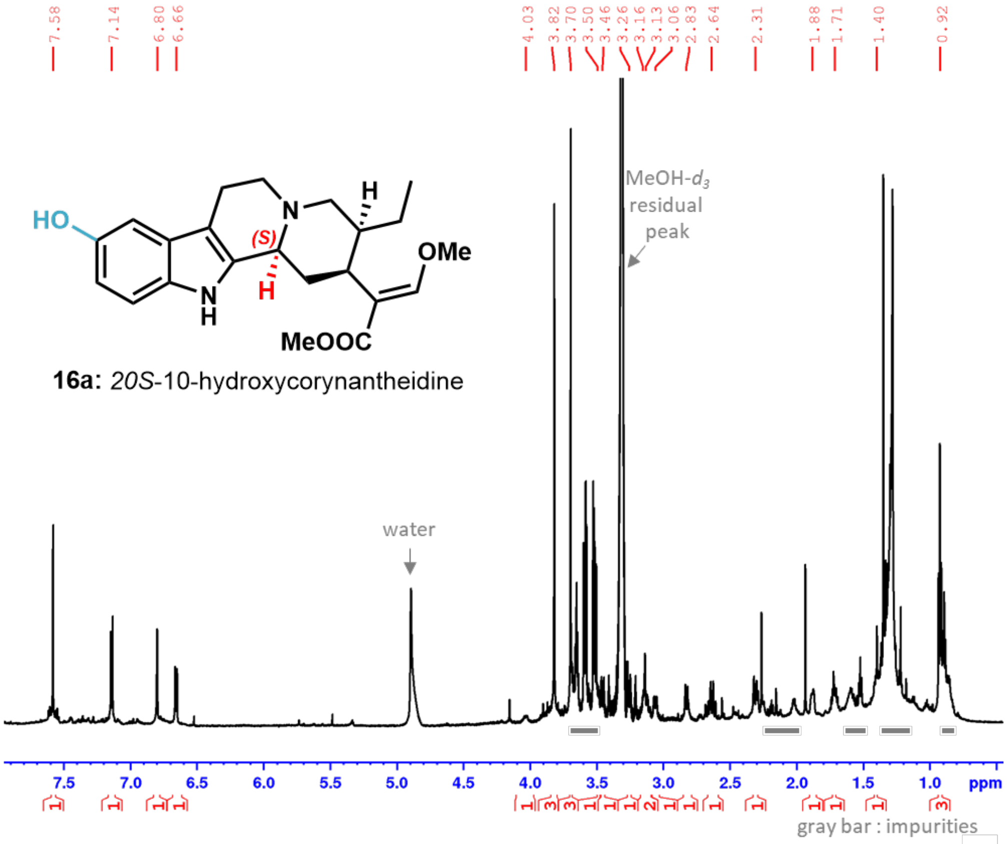

^{1}H NMR with water presaturation, aromatic range in MeOH-d_3

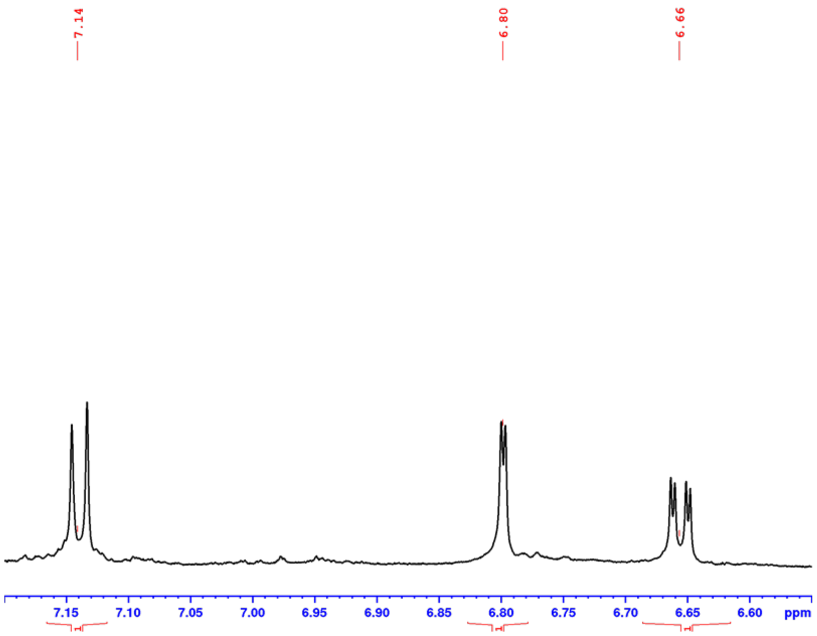

Supplementary Figure 73: NMR spectra for **16a:** (20S)-10-hydroxycorynantheidine.

NMR spectra for 20*S*-10-hydroxycorynantheidine (**16a**)

Water suppression HSQC, full range in MeOH-d_3

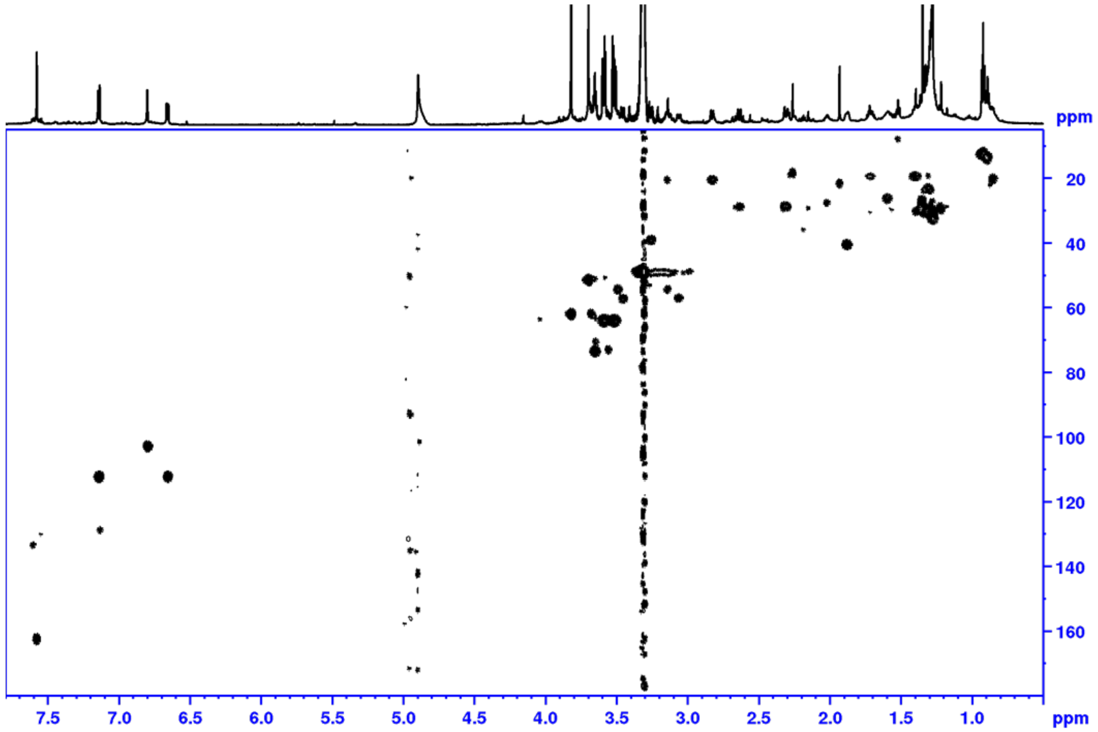

Water suppression COSY, full range in MeOH-d_3

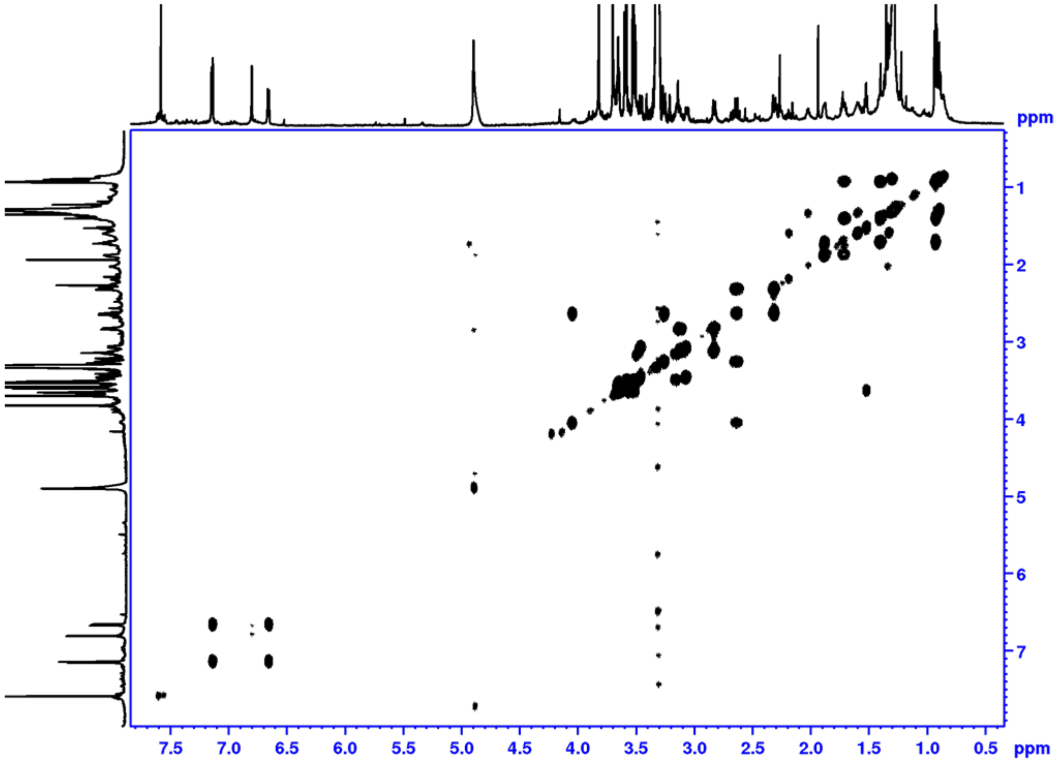

Supplementary Figure 74: NMR spectra for **16a:** (20*S*)-10-hydroxycorynantheidine.

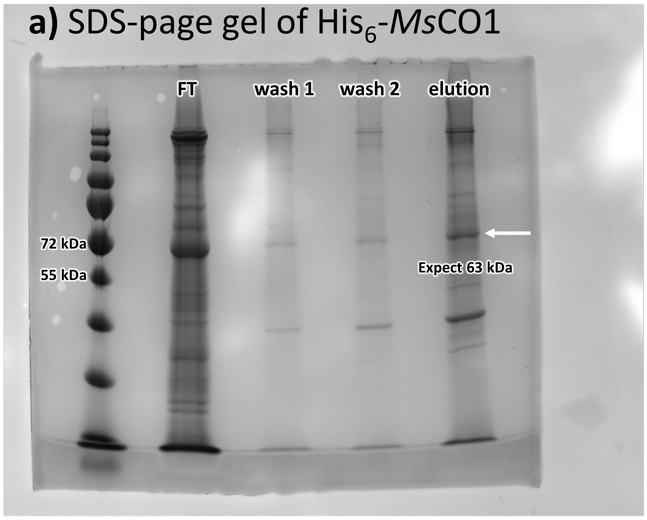

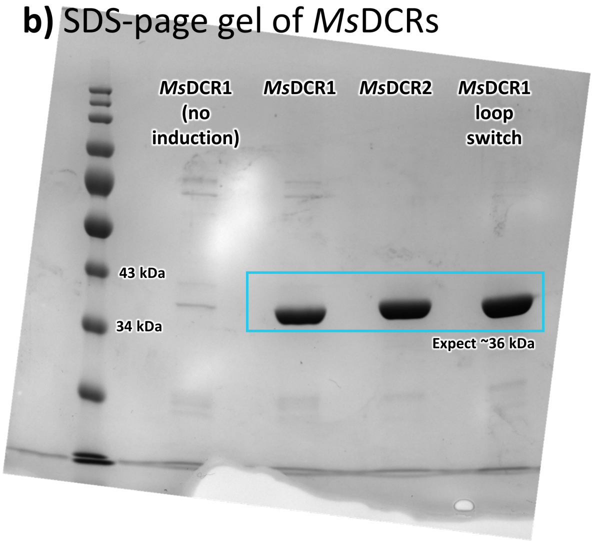

Supplemental Figure 75: Uncropped/unedited gel images pertaining to **a)** Supplementary Figure 15 **b)** Supplementary Figure 21.

Supplementary References

(1) Nguyen, T. M.; Grzech, D.; Chung, K.; Xia, Z.; Nguyen, T.; Dang, T. T. Discovery of a Cytochrome P450 Enzyme Catalyzing the Formation of Spirooxindole Alkaloid Scaffold. *Front. Plant Sci.* **2023**, No. February, 1–7. https://doi.org/10.3389/fpls.2023.1125158.

(2) Laus, G.; Brössner, D.; Senn, G.; Wurst, K. Analysis of the Kinetics of Isomerization of Spiro Oxindole Alkaloids. *J. Chem. Soc. Perkin Trans. 2* **1996**, *9*, 1931–1936. https://doi.org/10.1039/P29960001931.

(3) Lombe, B. K.; Zhou, T.; Caputi, L.; Ploss, K.; Connor, S. E. O. Biosynthetic Origin of the Methoxy Group in Quinine and Related Alkaloids Angewandte. *Angew. Chemie* **2024**, *e202418306*, 1–8. https://doi.org/10.1002/anie.202418306.

(4) Haas, B. J.; Papanicolaou, A.; Yassour, M.; Grabherr, M.; Blood, P. D.; Bowden, J.; Couger, M. B.; Eccles, D.; Li, B.; Lieber, M.; Macmanes, M. D.; Ott, M.; Orvis, J.; Pochet, N.; Strozzi, F.; Weeks, N.; Westerman, R.; William, T.; Dewey, C. N.; Henschel, R.; Leduc, R. D.; Friedman, N.; Regev, A. De Novo Transcript Sequence Reconstruction from RNA-Seq Using the Trinity Platform for Reference Generation and Analysis. *Nat. Protoc.* **2013**, *8* (8), 1494–1512. https://doi.org/10.1038/nprot.2013.084.

(5) Martin, M. Cutadapt Removes Adapter Sequences from High-Throughput Sequencing Reads. *EMBnet J.* **2011**, 5–7.

(6) McDonald, A. D.; Higgins, P. M.; Buller, A. R. Substrate Multiplexed Protein Engineering Facilitates Promiscuous Biocatalytic Synthesis. *Nat. Commun.* **2022**, *13*, 5242. https://doi.org/10.1038/s41467-022-32789-w.

(7) Misa, J.; Billingsley, J. M.; Niwa, K.; Yu, R. K.; Tang, Y. Engineered Production of Strictosidine and Analogues in Yeast. *ACS Synth. Biol.* **2022**, *11* (4), 1639–1649. https://doi.org/10.1021/acssynbio.2c00037.

(8) Kruegel, A. C.; Sames, D.; Javitch, J. A.; Majumdar, S. Deuterated Mitragynine Analogs as Safer Opioid Modulators in the Mitragynine Class. *Espacenet* **2020**, *WO20201602*.

(9) Chakraborty, S.; Uprety, R.; Slocum, S. T.; Irie, T.; Rouzic, V. Le; Li, X.; Wilson, L. L.; Scouller, B.; Alder, A. F.; Kruegel, A. C.; Ansono, M.; Varadi, A.; Eans, S. O.; Hunkele, A.; Allaoa, A.; Kalra, S.; Xu, J.; Pan, Y. X.; Pintar, J.; Kivell, B. M.; Pasternak, G. W.; Cameron, M. D.; Mclaughlin, J. P.; Sames, D. Oxidative Metabolism as a Modulator of Kratom's Biological Actions. *J. Med. Chem.* **2021**, *64*, 16553–16572. https://doi.org/10.1021/acs.jmedchem.1c01111.

(10) Kruegel, A. C.; Gassaway, M. M.; Kapoor, A.; Váradi, A.; Majumdar, S.; Filizola, M.; Javitch, J. A.; Sames, D. Synthetic and Receptor Signaling Explorations of the Mitragyna Alkaloids: Mitragynine as an Atypical Molecular Framework for Opioid Receptor Modulators. *J. Am. Chem. Soc.* **2016**, *138* (21), 6754–6764. https://doi.org/10.1021/jacs.6b00360.

(11) Fulmer, G. R.; Miller, A. J. M.; Sherden, N. H.; Gottlieb, H. E.; Nudelman, A.; Stoltz, B. M.; Bercaw, J. E.; Goldberg, K. I. NMR Chemical Shifts of Trace Impurities: Common Laboratory Solvents, Organics, and Gases in Deuterated Solvents Relevant to the Organometallic Chemist. *Organometallics* **2010**, *29* (9), 2176–2179. https://doi.org/10.1021/om100106e.

(12) Andreev, I. A.; Boichenko, M. A.; Ratmanova, N. K.; Ivanova, O. A.; Levina, I. I.; Khrustalev, V. N.; Sedov, I. A.; Trushkov, I. V. 4-(Dimethylamino)Pyridinium Azide in Protic Ionic Liquid Media as a Stable Equivalent of Hydrazoic Acid. *Adv. Synth. Catal.* **2022**, *364* (14), 2403–2415. https://doi.org/10.1002/adsc.202200486.

(13) Wanner, M. J.; Claveau, E.; Van Maarseveen, J. H.; Hiemstra, H. Enantioselective Syntheses of Corynanthe Alkaloids by Chiral Brønsted Acid and Palladium Catalysis. *Chem. - A Eur. J.* **2011**, *17* (49), 13680–13683. https://doi.org/10.1002/chem.201103150.

(14) Stærk, D.; Lemmich, E.; Christensen, J.; Kharazmi, A.; Olsen, C. E.; Jaroszewski, J. W. Leishmanicidal, Antiplasmodial and Cytotoxic Activity of Indole Alkaloids from Corynanthe

Pachyceras. *Planta Med* **2000**, *66*, 531–536.
(15) Ren, J.; Ding, S. H.; Li, X. N.; Zhao, Q. S. Unified Strategy Enables the Collective Syntheses of Structurally Diverse Indole Alkaloids. *J. Am. Chem. Soc.* **2024**, *146* (11), 7616–7627. https://doi.org/10.1021/jacs.3c13869.
(16) Lounasmaa, M., Jokela, R., Laine, C., & Hanhinen, P. Preparation of (+-)-Hirsutine and (+-)-3-Isocorynantheidine. *Heterocycles* **1998**, *49*, 445–450.
(17) Flores-bocanegra, L.; Raja, H. A.; Graf, T. N.; Augustinovic, M.; Wallace, E. D.; Hematian, S.; Kellogg, J. J.; Todd, D. A.; Cech, N. B.; Oberlies, N. H. The Chemistry of Kratom [Mitragyna Speciosa]: Updated Characterization Data and Methods to Elucidate Indole and Oxindole Alkaloids. *J. Nat. Prod.* **2020**, *83*, 2165–2177. https://doi.org/10.1021/acs.jnatprod.0c00257.
(18) Paradowska, K.; Wolniak, M.; Pisklak, M.; Gliński, J. A.; Davey, M. H.; Wawer, I. 13C, 15N CPMAS NMR and GIAO DFT Calculations of Stereoisomeric Oxindole Alkaloids from Cat's Claw (Uncaria Tomentosa). *Solid State Nucl. Magn. Reson.* **2008**, *34* (4), 202–209. https://doi.org/10.1016/j.ssnmr.2008.10.002.
(19) Kitajima, M.; Yokoya, M.; Takayama, H.; Aimi, N. Co-Occurrence of Harman and β-Carboline-Type Monoterpenoid Glucoindole Alkaloids in Uña de Gato (Uncaria Tomentosa). *Nat. Med.* **2001**, *55* (6), 308–310.
