# Supplementary material for: Enzymatic epimerization of monoterpene indole alkaloids in kratom
Source: Nat Chem Biol. 2025 Jul 16;22(2):229–38. doi: 10.1038/s41589-025-01970-9 (PMC12441186; doi:10.1038/s41589-025-01970-9)
Supplement: Supplementary file 2 — Reporting Summary [file 41589_2025_1970_MOESM2_ESM.pdf]

nature portfolio

Corresponding author(s): O'Connor, Sarah E.

Last updated by author(s): May 26, 2025

Reporting Summary

Nature Portfolio wishes to improve the reproducibility of the work that we publish. This form provides structure for consistency and transparency in reporting. For further information on Nature Portfolio policies, see our Editorial Policies and the Editorial Policy Checklist.

Statistics

For all statistical analyses, confirm that the following items are present in the figure legend, table legend, main text, or Methods section.

n/a	Confirmed	
☐	☒	The exact sample size (*n*) for each experimental group/condition, given as a discrete number and unit of measurement
☐	☒	A statement on whether measurements were taken from distinct samples or whether the same sample was measured repeatedly
☐	☒	The statistical test(s) used AND whether they are one- or two-sided<br>*Only common tests should be described solely by name; describe more complex techniques in the Methods section.*
☒	☐	A description of all covariates tested
☒	☐	A description of any assumptions or corrections, such as tests of normality and adjustment for multiple comparisons
☐	☒	A full description of the statistical parameters including central tendency (e.g. means) or other basic estimates (e.g. regression coefficient) AND variation (e.g. standard deviation) or associated estimates of uncertainty (e.g. confidence intervals)
☒	☐	For null hypothesis testing, the test statistic (e.g. *F*, *t*, *r*) with confidence intervals, effect sizes, degrees of freedom and *P* value noted<br>*Give P values as exact values whenever suitable.*
☒	☐	For Bayesian analysis, information on the choice of priors and Markov chain Monte Carlo settings
☒	☐	For hierarchical and complex designs, identification of the appropriate level for tests and full reporting of outcomes
☐	☒	Estimates of effect sizes (e.g. Cohen's *d*, Pearson's *r*), indicating how they were calculated

Our web collection on statistics for biologists contains articles on many of the points above.

Software and code

Policy information about availability of computer code

Data collection	Mass spectrometry data was collected on a Bruker UPLC-MS QTof Impact II system using Bruker Compass Hystar v6.2 software. NMR measurements were carried out on a 400 MHz Bruker Avance III HD spectrometer (Bruker Biospin GmbH, Rheinstetten, Germany), a 700 MHz Bruker Avance III HD spectrometer (Bruker Biospin GmbH, Rheinstetten, Germany) and a 500 MHz Bruker Avance III HD spectrometer (Bruker Biospin GmbH, Rheinstetten, Germany) using standard pulse sequences as implemented in Bruker Topspin ver. 3.6.1. 700 MHz and 500 MHz NMR were equipped with a TCI cryoprobe.
Data analysis	RNAseq reads related software are as follows (please see Methods section for specific details): Trinity (v2.15.1 and v2.3.2), Cutadapt (v3.4). Assay graphs were made in GraphPad Prism (8.2.0). Protein crystal structures/models were visualized in Pymol (2.3.2). Microsoft Powerpoint was used to generate figures and Microsoft Excel was used for data analysis. Nucleic acid and protein sequences were analyzed/designed in Geneous Prime (2019.1.1). Chemical structures were generated in Chendraw Professional (17.1). Protparam (ExPasy) online tool was used to calculate MW and extinction coefficients for determining protein concentrations. Transcripts and their homology were analyzed using Python (8.0) and BioPython. Docking was performed using Autodock Vina (4.2.6, Scripps). All calculations were performed using GaussView ver.6 (Semichem Inc., Shawnee, Kansas, USA) and Gaussian ver.16 (Gaussian Inc., Wallingford, Connecticut, USA).

For manuscripts utilizing custom algorithms or software that are central to the research but not yet described in published literature, software must be made available to editors and reviewers. We strongly encourage code deposition in a community repository (e.g. GitHub). See the Nature Portfolio guidelines for submitting code & software for further information.

Data

Policy information about availability of data

All manuscripts must include a data availability statement. This statement should provide the following information, where applicable:

- Accession codes, unique identifiers, or web links for publicly available datasets
- A description of any restrictions on data availability
- For clinical datasets or third party data, please ensure that the statement adheres to our policy

All studied gene/enzyme sequence data is available in the Supplemental Information. Kratom SRA data is deposited under Genbank BioProject PRJNA1244102. Uncaria guianensis SRA data is deposited under Genbank BioProject PRJNA1167310.

Research involving human participants, their data, or biological material

Policy information about studies with human participants or human data. See also policy information about sex, gender (identity/presentation), and sexual orientation and race, ethnicity and racism.

Reporting on sex and gender	NA
Reporting on race, ethnicity, or other socially relevant groupings	NA
Population characteristics	NA
Recruitment	NA
Ethics oversight	NA

Note that full information on the approval of the study protocol must also be provided in the manuscript.

Field-specific reporting

Please select the one below that is the best fit for your research. If you are not sure, read the appropriate sections before making your selection.

☒ Life sciences ☐ Behavioural & social sciences ☐ Ecological, evolutionary & environmental sciences

For a reference copy of the document with all sections, see nature.com/documents/nr-reporting-summary-flat.pdf

Life sciences study design

All studies must disclose on these points even when the disclosure is negative.

Sample size	Prior determination of sample size was not a consideration for our data. Sample size number was chosen with the following rationale: biological experiments were performed using three biological replicates; otherwise screening of gene candidates and enzyme mutant activity comparisons were performed in duplicate.
Data exclusions	No data was excluded.
Replication	Except for the purposes of comparing activity trends or gene screening (which were performed in duplicate), all experiments were performed in triplicate. For most characterization experiments, initial trials were performed first in singleton, followed by triplicate validation. We observed similar results from replicated experiments.
Randomization	Randomization as not relevant for our study because replicates were done independently and on different days. Replicates were analyzed in no particular order.
Blinding	Blinding was not relevant for our study because group allocation was not necessary to collect and process data.

Reporting for specific materials, systems and methods

We require information from authors about some types of materials, experimental systems and methods used in many studies. Here, indicate whether each material, system or method listed is relevant to your study. If you are not sure if a list item applies to your research, read the appropriate section before selecting a response.

Materials & experimental systems

n/a	Involved in the study
☒	☐ Antibodies
☒	☐ Eukaryotic cell lines
☒	☐ Palaeontology and archaeology
☒	☐ Animals and other organisms
☒	☐ Clinical data
☒	☐ Dual use research of concern
☐	☒ Plants

Methods

n/a	Involved in the study
☒	☐ ChIP-seq
☒	☐ Flow cytometry
☒	☐ MRI-based neuroimaging

Dual use research of concern

Policy information about dual use research of concern

Hazards

Could the accidental, deliberate or reckless misuse of agents or technologies generated in the work, or the application of information presented in the manuscript, pose a threat to:

No	Yes
☒	☐ Public health
☒	☐ National security
☒	☐ Crops and/or livestock
☒	☐ Ecosystems
☒	☐ Any other significant area

Experiments of concern

Does the work involve any of these experiments of concern:

No	Yes
☒	☐ Demonstrate how to render a vaccine ineffective
☒	☐ Confer resistance to therapeutically useful antibiotics or antiviral agents
☒	☐ Enhance the virulence of a pathogen or render a nonpathogen virulent
☒	☐ Increase transmissibility of a pathogen
☒	☐ Alter the host range of a pathogen
☒	☐ Enable evasion of diagnostic/detection modalities
☒	☐ Enable the weaponization of a biological agent or toxin
☒	☐ Any other potentially harmful combination of experiments and agents

Plants

Seed stocks	Kratom seeds were obtained from a collaborator at the University of Florida, as described in the Methods. The plants originated from Thailand. Nicotiana benthamiana plants were propogated from a long-standing stock (> 15 years).
Novel plant genotypes	Only wild-type plants were used.
Authentication	No genetic modification was performed on these plants. Kratom plants were characterized by RNAseq and metabolomic analysis as described in the manuscript.
